# Supplementary material for: Extra kinetic dimensions for label discrimination
Source: Nat Commun. 2022 Mar 18;13:1482. doi: 10.1038/s41467-022-29172-0 (PMC8933551; doi:10.1038/s41467-022-29172-0)
Supplement: Supplementary file 1 — Supplementary Information [file 41467_2022_29172_MOESM1_ESM.pdf]

Extra kinetic dimensions for multiplexed label discrimination

Raja Chouket,[1†] Agnès Pellissier-Tanon,[1†] Aliénor Lahlou,[1,2] Ruikang Zhang,[1]
Diana Kim,[1] Marie-Aude Plamont,[1] Mingshu Zhang,[3] Xi Zhang,[3]
Pingyong Xu,[3,4] Nicolas Desprat,[5,6] Dominique Bourgeois,[7] Agathe Espagne,[1]
Annie Lemarchand,[8*] Thomas Le Saux,[1*] Ludovic Jullien[1*]

[1]PASTEUR, Département de chimie, École normale supérieure,
PSL University, Sorbonne Université, CNRS, Paris, France
[2]Sony Computer Science Laboratories, Paris, France
[3]Key Laboratory of RNA Biology, Institute of Biophysics,
Chinese Academy of Sciences, Beijing, China
[4]College of Life Sciences, University of Chinese Academy of Sciences,
Beijing, China
[5]Laboratoire de Physique de L'ENS, École Normale Supérieure,
PSL University, CNRS, Sorbonne Université, Université de Paris, Paris, France
[6]Institut de Biologie de l'ENS (IBENS), École Normale Supérieure,
CNRS, INSERM, PSL University, Paris, France
[7]Univ. Grenoble Alpes, CNRS, CEA, IBS, F-38000 Grenoble, France
[8]LPTMC, Sorbonne Université, CNRS, Paris, France

[†]These authors contributed equally to this work.
[*]To whom correspondence should be addressed; E-mail:
Annie.Lemarchand@sorbonne-universite.fr,
Thomas.Lesaux@ens.psl.eu,
Ludovic.Jullien@ens.psl.eu.

December 13, 2021

Contents

1 Supplementary Methods **4**
1.1 Numbering and sequences of the RSFPs . . . 4
1.2 Characterization of the optical setup . . . 12
1.2.1 Configuration for acquiring the RSFP photoswitching information . . . 12
Temporal characterization of the illumination profiles . . . 12
Spatial characterization of the illumination profiles . . . 12
LEDs . . . 13
Lasers . . . 13
1.2.2 Configuration for acquiring the LIGHTNING images . . . 13
Spatial characterization of the illumination profiles . . . 13

1.2.3 Calibration of light intensities 16
LEDs 16
Lasers 16
1.3 Protocols of acquisition of the RSFP photochemical behavior for LIGHTNING implementation 17
1.4 Data processing for LIGHTNING implementation 19
1.5 Acquisition of the LIGHTNING kinetic fingerprints of the RSFPs 20
1.6 Processing of the LIGHTNING images of RSFP-labeled bacteria 20
1.6.1 Segmentation of bacteria 20
1.6.2 Registration of bacteria 21
1.6.3 Selection criteria 22
1.7 Definition of a set of distinguishable RSFPs 25
1.7.1 Sorting algorithms 25
Selection of the most distant RSFPs within sets of RSFPs 26
Selection of the most distant RSFPs within inclusive subsets of an RSFP set 26
1.7.2 Evaluation of the cutoff distance 28
1.8 Assigning an identity to an unknown RSFP 29

2 Supplementary Figures **30**
2.1 Absorption and fluorescence emission spectra of the RSFPs 30
2.2 Measurement of the rate constant k_{21}^{Δ} associated with the thermally driven relaxation of the photo-switched RSFPs 31
2.3 Photochemical characterization of the RSFPs 34
2.3.1 Preliminary illumination experiments on the RSFPs 34
2.3.2 Final illumination experiments 38
2.4 Photochemical characterization of the RSFP-labeled bacteria 60

3 Supplementary Tables **65**
3.1 Photophysical properties of the RSFPs 65
3.2 Photochemical properties of the RSFPs acquired in RSFP solutions 66

A LIGHTNING data collection **68**
A.1 Theoretical analysis of the time evolution of the RSF fluorescence signal upon constant illumination . 68
A.2 Design of the measurement cell of the photoswitchometer for liquid solutions 69
A.2.1 Fluorescence evolution in a homogeneous system 69
Illumination I 70
Illumination II 71
Illumination III 71
A.2.2 Impact of diffusion 72
Spherical illumination 72
Cylindrical illumination 76
Conclusion 77
A.3 Optimization of LIGHTNING acquisition 77
A.3.1 Discrimination power and number of discrimination dimensions 77

A.3.2 Optimization and time integration of the four illuminations . . . 77
A.3.3 Optimization of sampling . . . 78
A.3.4 Optimization of the photon budget and the acquisition duration . . . 79

B LIGHTNING data processing **81**
B.1 Spectrum of the characteristic times of RSF fluorescence evolution . . . 81
B.1.1 Generating a spectrum . . . 81
B.1.2 Application to RSFPs . . . 83
B.2 Monoexponential reduction of RSF fluorescence evolution . . . 84
B.2.1 A kinetic filter . . . 84
B.2.2 Application to RSFPs . . . 86
B.3 Comparison of the spectrum and the monoexponential reduction for extracting characteristic times . . . 86

C Kinetic models of fluorescence photoswitching in RSFPs **86**
C.1 An eight-state photoswitching mechanism for RSFPs relevant in the 1 μs–1 s time window . . . 87
C.2 Elimination of the singlet excited states – The five-state mechanism . . . 90
C.3 Reduced mechanisms valid for four different illuminations . . . 91
C.3.1 Illumination I_{low} . . . 91
C.3.2 Illumination I_{high} . . . 92
C.3.3 Illumination II_{low} . . . 92
C.3.4 Illumination II_{high} . . . 92
C.4 Non-redundancy of the characteristic times acquired under the four different illuminations . . . 93
C.5 Comparison of the experiments with the predictions of the reduced mechanisms of RSFPs . . . 94

D LIGHTNING discriminatory power **96**
D.1 On solutions of RSFPs . . . 96
D.1.1 LIGHTNING kinetic fingerprint of RSFPs . . . 96
D.1.2 LIGHTNING minimal distance among sets of RSFPs . . . 97
D.1.3 LIGHTNING cutoff distance d_c . . . 97
d_c evaluation . . . 97
Discussion . . . 100
D.2 On images of RSFP-labeled bacteria . . . 103
D.2.1 LIGHTNING kinetic fingerprint of RSFP-labeled bacteria . . . 103
D.2.2 LIGHTNING sorting of an optimized set of RSFP-labeled bacteria . . . 104
D.2.3 LIGHTNING discrimination of 9 RSFP-labeled bacteria . . . 105
D.3 The LIGHTNING robustness . . . 107
D.3.1 Results . . . 108
D.3.2 Discussion . . . 114
Dependence of the LIGHTNING kinetic fingerprint on the RSFP environment . . . 114
Dependence of the LIGHTNING kinetic fingerprint on the acquisition parameters . . . 115
Impact of the light intensities I_1 and I_2 . . . 116
Impact of the frequency and time window of fluorescence acquisition . . . 117
D.4 Colocalization of RSFPs . . . 119

1 Supplementary Methods

1.1 Numbering and sequences of the RSFPs

The numbering adopted for the RSFPs in this manuscript is provided below together with the sequences of the genes coding for these proteins:

- **1**: Dronpa.[1]
  ATGGTGAGTGTGATTAAACCAGACATGAAGATCAAGCTGCGTATGGAAGGCGCT
  GTAAATGGACACCCGTTCGCGATTGAAGGAGTTGGCCTTGGGAAGCCTTTCGAG
  GGAAAACAGAGTATGGACCTTAAAGTCAAAGAAGGCGGACCTCTGCCTTTCGCC
  TATGACATCTTGACAACTGTGTTCTGTTACGGCAACAGGGTATTCGCCAAATAC
  CCAGAAAATATAGTAGACTATTTCAAGCAGTCGTTTCCTGAGGGCTACTCTTGG
  GAACGAAGCATGAATTACGAAGACGGGGGCATTTGTAACGCGACAAACGACATA
  ACCCTGGATGGTGACTGTTATATCTATGAAATTCGATTTGATGGTGTGAACTTT
  CCTGCCAATGGTCCAGTTATGCAGAAGAGGACTGTGAAATGGGAGCCATCCACT
  GAGAAATTGTATGTGCGTGATGGAGTGCTGAAGGGTGATGTTAACATGGCTCTG
  TCGCTTGAAGGAGGTGGCCATTACCGATGTGACTTCAAAACTACTTATAAAGCT
  AAGAAGGTTGTCCAGTTGCCAGACTATCACTTTGTGGACCACCACATTGAGATT
  AAAAGCCACGACAAAGATTACAGTAATGTTAATCTGCATGAGCACGCCGAAGCG
  CATTCTGAGCTGCCGAGGCAGGCCAAG;

- **2**: Dronpa-2.[2]
  ATGGTGAGTGTGATTAAACCAGACATGAAGATCAAGCTGCGTATGGAAGGCGCT
  GTAAATGGACACCCGTTCGCGATTGAAGGAGTTGGCCTTGGGAAGCCTTTCGAG
  GGAAAACAGAGTATGGACCTTAAAGTCAAAGAAGGCGGACCTCTGCCTTTCGCC
  TATGACATCTTGACAACTGTGTTCTGTTACGGCAACAGGGTATTCGCCAAATAC
  CCAGAAAATATAGTAGACTATTTCAAGCAGTCGTTTCCTGAGGGCTACTCTTGG
  GAACGAAGCATGAATTACGAAGACGGGGGCATTTGTAACGCGACAAACGACATA
  ACCCTGGATGGTGACTGTTATATCTATGAAATTCGATTTGATGGTGTGAACTTT
  CCTGCCAATGGTCCAGTTATGCAGAAGAGGACTGTGAAATGGGAGCCATCCACT
  GAGAAATTGTATGTGCGTGATGGAGTGCTGAAGGGTGATGTTAACACGGCTCTG
  TCGCTTGAAGGAGGTGGCCATTACCGATGTGACTTCAAAACTACTTATAAAGCT
  AAGAAGGTTGTCCAGTTGCCAGACTATCACTTTGTGGACCACCACATTGAGATT
  AAAAGCCACGACAAAGATTACAGTAATGTTAATCTGCATGAGCACGCCGAAGCG
  CATTCTGAGCTGCCGAGGCAGGCCAAG;

- **3**: Dronpa-3.[3]
  ATGGTGAGTGTGATTAAACCAGACATGAAGATCAAGCTGCGTATGGAAGGCGCT
  GTAAATGGACACCCGTTCGCGATTGAAGGAGTTGGCCTTGGGAAGCCTTTCGAG
  GGAAAACAGAGTATGGACCTTAAAGTCAAAGAAGGCGGACCTCTGCCTTTCGCC
  TATGACATCTTGACAACTGTGTTCTGTTACGGCAACAGGGTATTCGCCAAATAC
  CCAGAAAATATAGTAGACTATTTCAAGCAGTCGTTTCCTGAGGGCTACTCTTGG
  GAACGAAGCATGAATTACGAAGACGGGGGCATTTGTAACGCGACAAACGACATA
  ACCCTGGATGGTGACTGTTATATCTATGAAATTCGATTTGATGGTGTGAACTTT

CCTGCCAATGGTCCAGTTATGCAGAAGAGGACTGTGAAATGGGAGCCATCCACT
GAGAAATTGTATGTGCGTGATGGAGTGCTGAAGGGTGATATTAACGCCGCTCTG
TCGCTTGAAGGAGGTGGCCATTACCGATGTGACTTCAAAACTACTTATAAAGCT
AAGAAGGTTGTCCAGTTGCCAGACTATCACTTTGTGGACCACCACATTGAGATT
AAAAGCCACGACAAAGATTACAGTAATGTTAATCTGCATGAGCACGCCGAAGCG
CATTCTGAGCTGCCGAGGCAGGCCAAGTAA;

- **4**: Kohinoor.[4]
  ATGAGTGTGATTAAACCAGACATGAAGATCAAGCTGCGTATGGAAGGCGCTGTA
  AATGGACACCCGTTCGCGATTGAAGGAGTTGGCCTTGGGAAGCCTTTCGAGGGA
  AAACAGAGTATGGACCTTAAAGTCAAAGAAGGCGGACCTCTGCCTTTCGCCTAT
  GACATCTTGACAATGGCCTTCTGTTACGGCAACAGGGTATTCGCCAAATACCCA
  GAAAATATAGTAGATTATTTCAAGCAGTCGTTTCCTGAGGGCTACTCTTGGGAA
  CGAAGCATGATTTACGAAGACGGGGGCATTTGTATCGCGACAAACGACATAACC
  CTGGATGGTGACTGTTATATCTATGAAATTCGATTTGATGGTGTGAACTTTCCT
  GCCAATGGTCCAGTTATGCAGAAGAGGACTGTGAAATGGGAGCCATCCACTGAG
  AAATTGTATGTGCGTGATGGAGTGCTGAAGAGCGATGGCAATTACGCTCTGTCG
  CTTGAAGGAGGTGGCCACTACCGATGTGACTCCAAAACTACTTATAAAGCTAAG
  AAGGTTGTCCAGTTGCCAGACTATCACGATGTGGTCCACCACATTGAGATTAAA
  AGCCACGACAGAGATTACAGTAATGTTAATCTGCATGAGCATGCCGAAGCGCAT
  TCTGGGCTGCCGAGGCAGGCCAAGTAA;

- **5**: Padron.[5]
  ATGAGTGTGATTAAACCAGACATGAAGATCAAGCTGCGTATGGAAGGCGCTGTA
  AATGGACACCCGTTCGCGATTGAAGGAGTTGGCCTTGGGAAGCCTTTCGAGGGA
  AAACAGAGTATGGACCTTAAAGTCAAAGAAGGCGGACCTCTGCCTTTCGCCTAT
  GACATCTTGACAATGGCGTTCTGTTACGGCAACAGGGTATTCGCCAAATACCCA
  GAAAATATAGTAGACTATTTCAAGCAGTCGTTTCCTGAGGGCTACTCTTGGGAA
  CGAAGCATGATTTACGAAGACGGGGGCATTTGTAACGCGACAAACGACATAACC
  CTGGATGGTGACTGTTATATCTATGAAATTCGATTTGATGGTGTGAACTTTCCT
  GCCAATGGTCCAGTTATGCAGAAGAGGACTGTGAAATGGGAGCTATCCACTGAG
  AAATTGTATGTGCGTGATGGAGTGCTGAAGTCTGATGGTAACTACGCTCTGTCG
  CTTGAAGGAGGTGGCCATTACCGATGTGACTTCAAAACTACTTATAAAGCTAAG
  AAGGTTGTCCAGTTGCCAGACTATCACTCTGTGGACCACCACATTGAGATTAAA
  AGCCACGACAAAGATTACAGTAATGTTAATCTGCATGAGCATGCCGAAGCGCAT
  TCTGAGCTGCCGAGGCAGGCCAAG;

- **6**: rsEGFP2.[6]
  ATGGTGAGCAAGGGCGAGGAGCTGTTCACCGGGGTGGTGCCCATCCTGGTCGAG
  CTGGACGGCGACGTAAACGGCCACAAGTTCAGCGTGTCCGGCGAGGGCGAGGGC
  GATGCCACCTACGGCAAGCTGACCCTGAAGTTCATCTGCACCACCGGCAAGCTG
  CCCGTGCCCTGGCCCACCCTCGTGACCACCCTGGCCTACGGCGTGCTGTGCTTC
  AGCCGCTACCCCGACCACATGAAGCAGCACGACTTCTTCAAGTCCGCCATGCCC
  GAAGGCTACGTCCAGGAGCGCACCATCTTCTTCAAGGACGACGGCAACTACAAG

ACCCGCGCCGAGGTGAAGTTCGAGGGCGACACCCTGGTGAACCGCATCGAGCTG
AAGGGCATCGACTTCAAGGAGGACGGCAACATCCTGGGGCACAAGCTGGAGTAC
AACTACAACAGCCACAACGTCTATATCATGGCCGACAAGCAGAAGAACGGCATC
AAGTCTAACTTCAAGATCCGCCACAACATCGAGGACGGCAGCGTGCAGCTCGCC
GACCACTACCAGCAGAACACCCCCATCGGCGACGGCCCCGTGCTGCTGCCCGAC
AACCACTACCTGAGCACCCAGTCCAAGCTGAGCAAAGACCCCAACGAGAAGCGC
GATCACATGGTCCTGCTGGAGTTCGTGACCGCCGCCGGGATCACTCTCGGCATG
GACGAGCTGTACAAG;

- **7**: rsFastLime.[2,5]
  ATGGTGAGTGTGATTAAACCAGACATGAAGATCAAGCTGCGTATGGAAGGCGCT
  GTAAATGGACACCCGTTCGCGATTGAAGGAGTTGGCCTTGGGAAGCCTTTCGAG
  GGAAAACAGAGTATGGACCTTAAAGTCAAAGAAGGCGGACCTCTGCCTTTCGCC
  TATGACATCTTGACAACTGTGTTCTGTTACGGCAACAGGGTATTCGCCAAATAC
  CCAGAAAATATAGTAGACTATTTCAAGCAGTCGTTTCCTGAGGGCTACTCTTGG
  GAACGAAGCATGAATTACGAAGACGGGGGCATTTGTAACGCGACAAACGACATA
  ACCCTGGATGGTGACTGTTATATCTATGAAATTCGATTTGATGGTGTGAACTTT
  CCTGCCAATGGTCCAGTTATGCAGAAGAGGACTGTGAAATGGGAGCCATCCACT
  GAGAAATTGTATGTGCGTGATGGAGTGCTGAAGGGTGATGGTAACATGGCTCTG
  TCGCTTGAAGGAGGTGGCCATTACCGATGTGACTTCAAAACTACTTATAAAGCT
  AAGAAGGTTGTCCAGTTGCCAGACTATCACTTTGTGGACCACCACATTGAGATT
  AAAAGCCACGACAAAGATTACAGTAATGTTAATCTGCATGAGCACGCCGAAGCG
  CATTCTGAGCTGCCGAGGCAGGCCAAG;

- **8**: Skylan-MVF.
  CATCATCATCATCATCATGGTATGGCTAGCATGACTGGTGGACAGCAAATGGGT
  CGGGATCTGTACGACGATGACGATAAGGATCGATGGGGATCCATGAGTGCGATT
  AAGCCAGACATGAAGATCAAACTCCGTATGGAAGGCAACGTAAACGGGCACCAC
  TTTGTGATCGACGGAGATGGTACAGGCAAGCCTTTTGAGGGAAAACAGAGTATG
  GATCTTGAAGTCAAAGAGGGCGGACCTCTGCCTTTTGCCTTTGATATCCTGACC
  ACTGCATTCATGTACGGCAACAGGGTATTCGCCAAATATCCAGACAACATACAA
  GACTATTTTAAGCAGTCGTTTCCTAAGGGGTATTCGTGGGAACGAAGCTTGACT
  TTCGAAGACGGGGGCATTTGCAACGCCAGAAACGACATAACAATGGAAGGGGAC
  ACTTTCTATAATAAAGTTCGATTTTATGGTACCAACTTTCCCGCCAATGGTCCA
  GTTATGCAGAAGAAGACGCTGAAATGGGAGCCCTCCACTGAGAAAATGTATGTG
  CGTGATGGAGTGCTGACGGGTGATGTAGAGATGGCTTTGTTGCTTGAAGGAAAT
  GCCCATTACCGATGTGACTTCAGAACTACTTACAAAGCTAAGGAGAAGGGTGTC
  AAGTTACCAGGCGCCCACTTTGTGGACCACTGCATTGAGATTTTAAGCCATGAC
  AAAGATTACAACAAGGTTAAGCTGTATGAGCATGCTGTTGCTCATTCTGGATTG
  CCTGACAATGCCAGACGATAA;

- **9**: Skylan-SVF.
  CATCATCATCATCATCATGGTATGGCTAGCATGACTGGTGGACAGCAAATGGGT
  CGGGATCTGTACGACGATGACGATAAGGATCGATGGGGATCCATGAGTGCGATT

AAGCCAGACATGAAGATCAAACTCCGTATGGAAGGCAACGTAAACGGGCACCAC
TTTGTGATCGACGGAGATGGTACAGGCAAGCCTTTTGAGGGAAAACAGAGTATG
GATCTTGAAGTCAAAGAGGGCGGACCTCTGCCTTTTGCCTTTGATATCCTGACC
ACTGCATTCTCCTACGGCAACAGGGTATTCGCCAAATATCCAGACAACATACAA
GACTATTTTAAGCAGTCGTTTCCTAAGGGGTATTCGTGGGAACGAAGCTTGACT
TTCGAAGACGGGGGCATTTGCAACGCCAGAAACGACATAACAATGGAAGGGGAC
ACTTTCTATAATAAAGTTCGATTTTATGGTACCAACTTTCCCGCCAATGGTCCA
GTTATGCAGAAGAAGACGCTGAAATGGGAGCCCTCCACTGAGAAAATGTATGTG
CGTGATGGAGTGCTGACGGGTGATGTAGAGATGGCTTTGTTGCTTGAAGGAAAT
GCCCATTACCGATGTGACTTCAGAACTACTTACAAAGCTAAGGAGAAGGGTGTC
AAGTTACCAGGCGCCCACTTTGTGGACCACTGCATTGAGATTTTAAGCCATGAC
AAAGATTACAACAAGGTTAAGCTGTATGAGCATGCTGTTGCTCATTCTGGATTG
CCTGACAATGCCAGACGATAA;

- **10**: Skylan-LVF.
  CATCATCATCATCATCATGGTATGGCTAGCATGACTGGTGGACAGCAAATGGGT
  CGGGATCTGTACGACGATGACGATAAGGATCGATGGGGATCCATGAGTGCGATT
  AAGCCAGACATGAAGATCAAACTCCGTATGGAAGGCAACGTAAACGGGCACCAC
  TTTGTGATCGACGGAGATGGTACAGGCAAGCCTTTTGAGGGAAAACAGAGTATG
  GATCTTGAAGTCAAAGAGGGCGGACCTCTGCCTTTTGCCTTTGATATCCTGACC
  ACTGCATTCCTGTACGGCAACAGGGTATTCGCCAAATATCCAGACAACATACAA
  GACTATTTTAAGCAGTCGTTTCCTAAGGGGTATTCGTGGGAACGAAGCTTGACT
  TTCGAAGACGGGGGCATTTGCAACGCCAGAAACGACATAACAATGGAAGGGGAC
  ACTTTCTATAATAAAGTTCGATTTTATGGTACCAACTTTCCCGCCAATGGTCCA
  GTTATGCAGAAGAAGACGCTGAAATGGGAGCCCTCCACTGAGAAAATGTATGTG
  CGTGATGGAGTGCTGACGGGTGATGTAGAGATGGCTTTGTTGCTTGAAGGAAAT
  GCCCATTACCGATGTGACTTCAGAACTACTTACAAAGCTAAGGAGAAGGGTGTC
  AAGTTACCAGGCGCCCACTTTGTGGACCACTGCATTGAGATTTTAAGCCATGAC
  AAAGATTACAACAAGGTTAAGCTGTATGAGCATGCTGTTGCTCATTCTGGATTG
  CCTGACAATGCCAGACGATAA;

- **11**: Skylan-EVF.
  CATCATCATCATCATCATGGTATGGCTAGCATGACTGGTGGACAGCAAATGGGT
  CGGGATCTGTACGACGATGACGATAAGGATCGATGGGGATCCATGAGTGCGATT
  AAGCCAGACATGAAGATCAAACTCCGTATGGAAGGCAACGTAAACGGGCACCAC
  TTTGTGATCGACGGAGATGGTACAGGCAAGCCTTTTGAGGGAAAACAGAGTATG
  GATCTTGAAGTCAAAGAGGGCGGACCTCTGCCTTTTGCCTTTGATATCCTGACC
  ACTGCATTCGAGTACGGCAACAGGGTATTCGCCAAATATCCAGACAACATACAA
  GACTATTTTAAGCAGTCGTTTCCTAAGGGGTATTCGTGGGAACGAAGCTTGACT
  TTCGAAGACGGGGGCATTTGCAACGCCAGAAACGACATAACAATGGAAGGGGAC
  ACTTTCTATAATAAAGTTCGATTTTATGGTACCAACTTTCCCGCCAATGGTCCA
  GTTATGCAGAAGAAGACGCTGAAATGGGAGCCCTCCACTGAGAAAATGTATGTG
  CGTGATGGAGTGCTGACGGGTGATGTAGAGATGGCTTTGTTGCTTGAAGGAAAT
  GCCCATTACCGATGTGACTTCAGAACTACTTACAAAGCTAAGGAGAAGGGTGTC

AAGTTACCAGGCGCCCACTTTGTGGACCACTGCATTGAGATTTTAAGCCATGAC
AAAGATTACAACAAGGTTAAGCTGTATGAGCATGCTGTTGCTCATTCTGGATTG
CCTGACAATGCCAGACGATAA;

- **12**: Skylan-VVF.
  CATCATCATCATCATCATGGTATGGCTAGCATGACTGGTGGACAGCAAATGGGT
  CGGGATCTGTACGACGATGACGATAAGGATCGATGGGGATCCATGAGTGCGATT
  AAGCCAGACATGAAGATCAAACTCCGTATGGAAGGCAACGTAAACGGGCACCAC
  TTTGTGATCGACGGAGATGGTACAGGCAAGCCTTTTGAGGGAAAACAGAGTATG
  GATCTTGAAGTCAAAGAGGGCGGACCTCTGCCTTTTGCCTTTGATATCCTGACC
  ACTGCATTCGTGTACGGCAACAGGGTATTCGCCAAATATCCAGACAACATACAA
  GACTATTTTAAGCAGTCGTTTCCTAAGGGGTATTCGTGGGAACGAAGCTTGACT
  TTCGAAGACGGGGGCATTTGCAACGCCAGAAACGACATAACAATGGAAGGGGAC
  ACTTTCTATAATAAAGTTCGATTTTATGGTACCAACTTTCCCGCCAATGGTCCA
  GTTATGCAGAAGAAGACGCTGAAATGGGAGCCCTCCACTGAGAAAATGTATGTG
  CGTGATGGAGTGCTGACGGGTGATGTAGAGATGGCTTTGTTGCTTGAAGGAAAT
  GCCCATTACCGATGTGACTTCAGAACTACTTACAAAGCTAAGGAGAAGGGTGTC
  AAGTTACCAGGCGCCCACTTTGTGGACCACTGCATTGAGATTTTAAGCCATGAC
  AAAGATTACAACAAGGTTAAGCTGTATGAGCATGCTGTTGCTCATTCTGGATTG
  CCTGACAATGCCAGACGATAA;
- **13**: Skylan-MIF.
  CATCATCATCATCATCATGGTATGGCTAGCATGACTGGTGGACAGCAAATGGGT
  CGGGATCTGTACGACGATGACGATAAGGATCGATGGGGATCCATGAGTGCGATT
  AAGCCAGACATGAAGATCAAACTCCGTATGGAAGGCAACGTAAACGGGCACCAC
  TTTGTGATCGACGGAGATGGTACAGGCAAGCCTTTTGAGGGAAAACAGAGTATG
  GATCTTGAAGTCAAAGAGGGCGGACCTCTGCCTTTTGCCTTTGATATCCTGACC
  ACTGCATTCATGTACGGCAACAGGGTATTCGCCAAATATCCAGACAACATACAA
  GACTATTTTAAGCAGTCGTTTCCTAAGGGGTATTCGTGGGAACGAAGCTTGACT
  TTCGAAGACGGGGGCATTTGCAACGCCAGAAACGACATAACAATGGAAGGGGAC
  ACTTTCTATAATAAAGTTCGATTTTATGGTACCAACTTTCCCGCCAATGGTCCA
  GTTATGCAGAAGAAGACGCTGAAATGGGAGCCCTCCACTGAGAAAATGTATGTG
  CGTGATGGAGTGCTGACGGGTGATATTGAGATGGCTTTGTTGCTTGAAGGAAAT
  GCCCATTACCGATGTGACTTCAGAACTACTTACAAAGCTAAGGAGAAGGGTGTC
  AAGTTACCAGGCGCCCACTTTGTGGACCACTGCATTGAGATTTTAAGCCATGAC
  AAAGATTACAACAAGGTTAAGCTGTATGAGCATGCTGTTGCTCATTCTGGATTG
  CCTGACAATGCCAGACGATAA;
- **14**: Skylan-SIF.
  CATCATCATCATCATCATGGTATGGCTAGCATGACTGGTGGACAGCAAATGGGT
  CGGGATCTGTACGACGATGACGATAAGGATCGATGGGGATCCATGAGTGCGATT
  AAGCCAGACATGAAGATCAAACTCCGTATGGAAGGCAACGTAAACGGGCACCAC
  TTTGTGATCGACGGAGATGGTACAGGCAAGCCTTTTGAGGGAAAACAGAGTATG
  GATCTTGAAGTCAAAGAGGGCGGACCTCTGCCTTTTGCCTTTGATATCCTGACC

ACTGCATTCTCCTACGGCAACAGGGTATTCGCCAAATATCCAGACAACATACAA
GACTATTTTAAGCAGTCGTTTCCTAAGGGGTATTCGTGGGAACGAAGCTTGACT
TTCGAAGACGGGGGCATTTGCAACGCCAGAAACGACATAACAATGGAAGGGGAC
ACTTTCTATAATAAAGTTCGATTTTATGGTACCAACTTTCCCGCCAATGGTCCA
GTTATGCAGAAGAAGACGCTGAAATGGGAGCCCTCCACTGAGAAAATGTATGTG
CGTGATGGAGTGCTGACGGGTGATATTGAGATGGCTTTGTTGCTTGAAGGAAAT
GCCCATTACCGATGTGACTTCAGAACTACTTACAAAGCTAAGGAGAAGGGTGTC
AAGTTACCAGGCGCCCACTTTGTGGACCACTGCATTGAGATTTTAAGCCATGAC
AAAGATTACAACAAGGTTAAGCTGTATGAGCATGCTGTTGCTCATTCTGGATTG
CCTGACAATGCCAGACGATAA;

- **15**: Skylan-MVS.
  CATCATCATCATCATCATGGTATGGCTAGCATGACTGGTGGACAGCAAATGGGT
  CGGGATCTGTACGACGATGACGATAAGGATCGATGGGGATCCATGAGTGCGATT
  AAGCCAGACATGAAGATCAAACTCCGTATGGAAGGCAACGTAAACGGGCACCAC
  TTTGTGATCGACGGAGATGGTACAGGCAAGCCTTTTGAGGGAAAACAGAGTATG
  GATCTTGAAGTCAAAGAGGGCGGACCTCTGCCTTTTGCCTTTGATATCCTGACC
  ACTGCATTCATGTACGGCAACAGGGTATTCGCCAAATATCCAGACAACATACAA
  GACTATTTTAAGCAGTCGTTTCCTAAGGGGTATTCGTGGGAACGAAGCTTGACT
  TTCGAAGACGGGGGCATTTGCAACGCCAGAAACGACATAACAATGGAAGGGGAC
  ACTTTCTATAATAAAGTTCGATTTTATGGTACCAACTTTCCCGCCAATGGTCCA
  GTTATGCAGAAGAAGACGCTGAAATGGGAGCCCTCCACTGAGAAAATGTATGTG
  CGTGATGGAGTGCTGACGGGTGATGTAGAGATGGCTTTGTTGCTTGAAGGAAAT
  GCCCATTACCGATGTGACTCCAGAACTACTTACAAAGCTAAGGAGAAGGGTGTC
  AAGTTACCAGGCGCCCACTTTGTGGACCACTGCATTGAGATTTTAAGCCATGAC
  AAAGATTACAACAAGGTTAAGCTGTATGAGCATGCTGTTGCTCATTCTGGATTG
  CCTGACAATGCCAGACGATAA;

- **16**: Skylan-SVS.
  CATCATCATCATCATCATGGTATGGCTAGCATGACTGGTGGACAGCAAATGGGT
  CGGGATCTGTACGACGATGACGATAAGGATCGATGGGGATCCATGAGTGCGATT
  AAGCCAGACATGAAGATCAAACTCCGTATGGAAGGCAACGTAAACGGGCACCAC
  TTTGTGATCGACGGAGATGGTACAGGCAAGCCTTTTGAGGGAAAACAGAGTATG
  GATCTTGAAGTCAAAGAGGGCGGACCTCTGCCTTTTGCCTTTGATATCCTGACC
  ACTGCATTCTCCTACGGCAACAGGGTATTCGCCAAATATCCAGACAACATACAA
  GACTATTTTAAGCAGTCGTTTCCTAAGGGGTATTCGTGGGAACGAAGCTTGACT
  TTCGAAGACGGGGGCATTTGCAACGCCAGAAACGACATAACAATGGAAGGGGAC
  ACTTTCTATAATAAAGTTCGATTTTATGGTACCAACTTTCCCGCCAATGGTCCA
  GTTATGCAGAAGAAGACGCTGAAATGGGAGCCCTCCACTGAGAAAATGTATGTG
  CGTGATGGAGTGCTGACGGGTGAtGTAGAGATGGCTTTGTTGCTTGAAGGAAAT
  GCCCATTACCGATGTGACTCCAGAACTACTTACAAAGCTAAGGAGAAGGGTGTC
  AAGTTACCAGGCGCCCACTTTGTGGACCACTGCATTGAGATTTTAAGCCATGAC
  AAAGATTACAACAAGGTTAAGCTGTATGAGCATGCTGTTGCTCATTCTGGATTG
  CCTGACAATGCCAGACGATAA;

- **17**: Skylan-LVS.

  CATCATCATCATCATCATGGTATGGCTAGCATGACTGGTGGACAGCAAATGGGT
  CGGGATCTGTACGACGATGACGATAAGGATCGATGGGGATCCATGAGTGCGATT
  AAGCCAGACATGAAGATCAAACTCCGTATGGAAGGCAACGTAAACGGGCACCAC
  TTTGTGATCGACGGAGATGGTACAGGCAAGCCTTTTGAGGGAAAACAGAGTATG
  GATCTTGAAGTCAAAGAGGGCGGACCTCTGCCTTTTGCCTTTGATATCCTGACC
  ACTGCATTCCTGTACGGCAACAGGGTATTCGCCAAATATCCAGACAACATACAA
  GACTATTTTAAGCAGTCGTTTCCTAAGGGGTATTCGTGGGAACGAAGCTTGACT
  TTCGAAGACGGGGGCATTTGCAACGCCAGAAACGACATAACAATGGAAGGGGAC
  ACTTTCTATAATAAAGTTCGATTTTATGGTACCAACTTTCCCGCCAATGGTCCA
  GTTATGCAGAAGAAGACGCTGAAATGGGAGCCCTCCACTGAGAAAATGTATGTG
  CGTGATGGAGTGCTGACGGGTGATGTAGAGATGGCTTTGTTGCTTGAAGGAAAT
  GCCCATTACCGATGTGACTCCAGAACTACTTACAAAGCTAAGGAGAAGGGTGTC
  AAGTTACCAGGCGCCCACTTTGTGGACCACTGCATTGAGATTTTAAGCCATGAC
  AAAGATTACAACAAGGTTAAGCTGTATGAGCATGCTGTTGCTCATTCTGGATTG
  CCTGACAATGCCAGACGATAA;

- **18**: Skylan-SLF.

  CATCATCATCATCATCATGGTATGGCTAGCATGACTGGTGGACAGCAAATGGGT
  CGGGATCTGTACGACGATGACGATAAGGATCGATGGGGATCCATGAGTGCGATT
  AAGCCAGACATGAAGATCAAACTCCGTATGGAAGGCAACGTAAACGGGCACCAC
  TTTGTGATCGACGGAGATGGTACAGGCAAGCCTTTTGAGGGAAAACAGAGTATG
  GATCTTGAAGTCAAAGAGGGCGGACCTCTGCCTTTTGCCTTTGATATCCTGACC
  ACTGCATTCTCCTACGGCAACAGGGTATTCGCCAAATATCCAGACAACATACAA
  GACTATTTTAAGCAGTCGTTTCCTAAGGGGTATTCGTGGGAACGAAGCTTGACT
  TTCGAAGACGGGGGCATTTGCAACGCCAGAAACGACATAACAATGGAAGGGGAC
  ACTTTCTATAATAAAGTTCGATTTTATGGTACCAACTTTCCCGCCAATGGTCCA
  GTTATGCAGAAGAAGACGCTGAAATGGGAGCCCTCCACTGAGAAAATGTATGTG
  CGTGATGGAGTGCTGACGGGTGATCTGGAGATGGCTTTGTTGCTTGAAGGAAAT
  GCCCATTACCGATGTGACTTCAGAACTACTTACAAAGCTAAGGAGAAGGGTGTC
  AAGTTACCAGGCGCCCACTTTGTGGACCACTGCATTGAGATTTTAAGCCATGAC
  AAAGATTACAACAAGGTTAAGCTGTATGAGCATGCTGTTGCTCATTCTGGATTG
  CCTGACAATGCCAGACGATAA;

- **19**: rsFolder.

  ATGGTAAGCAAGGGCGAGGAGCTGTTCACCGGCGTGGTGCCAATCCTGGTGGAG
  CTGGATGGCGATGTGAACGGCCACAAGTTTAGCGTGCGCGGCGAGGGCGAGGGC
  GATGCTACCAACGGCAAGCTGACCCTGAAGTTTATCTGCACCACCGGCAAGCTG
  CCGGTGCCGTGGCCAACCCTGGTGACCACCCTGGCCTACGGCGTGCTGTGCTTT
  TCCCGCTACCCGGATCACATGAAGCGCCACGACTTTTTCAAGAGCGCCaTGCCG
  GAGGGCTACGTGCAgGAGCGCACCATCaGCTTCAAGGATGACGGCACCTACAAG
  ACCCGCGCTGAGGTGAAGTTTGAGGGCGATACCCTGGTGAACCGCATCGAGCTG
  AAGGGCATCgACTTTAAGGAGGATGGCAACATCCTGGGCCACAAGCTGGAGTAC
  AACTTTAACAGCCaCAACGTGTACATCACCGCCGAcaACGAGAAGAACGGCATC

AAGAGCAACTTCAAgATCCGCCACaAaCGTGGAGGATGGCTCCgTgCaGCTGGC
CGAcCACTACCagcaGAACACCCCAATCGGcgATGGCCCGGtGCTGCTGcCAGA
CAACCACTACCTGAgCACCCaGaGcaAGCTgAGCaAgGACCCgaAACGAgAAgc
gCGACCaCaTGgTGCtGCtGGAgTTtGTGA;

- **20**: rsFolder2.
ATGGTAAGCAAGGGCGAGGAGCTGTTCACCGGCGTGGTGCCAATCCTGGTGGAG
CTGGATGGCGATGTGAACGGCCACAAGTTTAGCGTGCGCGGCGAGGGCGAGGGC
GATGCTACCAACGGCAAGCTGACCCTGAAGTTTATCTGCACCACCGGCAAGCTG
CCGGTGCCGTGGCCAACCCTGGTGACCACCCTGGCCTACGGCGTGCTGTGCTTT
TCCCGCTACCCGGATCACATGAAGCGCCACGACTTTTTCAAGAGCGCCATGCCG
GAGGGCTACGTGCAGGAGCGCACCATCAGCTTCAAGGATGACGGCACCTACAAG
ACCCGCGCTGAGGTGAAGTTTGAGGGCGATACCCTGGTGAACCGCATCGAGCTG
AAGGGCATCGACTTTAAGGAGGATGGCAACATCCTGGGCCACAAGCTGGAGTAC
AACTATAACAGCCACAACGTGTACATCACCGCCGACAAGCAGAAGAACGGCATC
AAGAGCAACTTCAAGATCCGCCACAACGTGGAGGAtGGCTCCGtGCaGCTGGCC
GAcCACTACCAGCAGAACACCCCAATCGGCGATGGCCCGGTGCTGCTGCCAGAC
AACCACTACCTGaGCACCCAGAgCAAGCTGAgCAaGGAcCCGAACGAGAAGcGC
GACCACATGGtGCTGCTGgAgTTTGTGACCGCTGCTGGCATCaCCCACgGcATG
GATGAgCTGTaCAAGGgATCCGGCTGCTaA;

- **21**: rsFolder2 V151A.
ATGGTAAGCAAGGGCGAGGAGCTGTTCACCGGCGTGGTGCCAATCCTGGTGGAG
CTGGATGGCGATGTGAACGGCCACAAGTTTAGCGTGCGCGGCGAGGGCGAGGGC
GATGCTACCAACGGCAAGCTGACCCTGAAGTTTATCTGCACCACCGGCAAGCTG
CCGGTGCCGTGGCCAACCCTGGTGACCACCCTGGCCTACGGCGTGCTGTGCTTT
TCCCGCTACCCGGATCACATGAAGCGCCACGACTTTTTCAAGAGCGCCATGCCG
GAGGGCTACGTGCAGGAGCGCACCATCAGCTTCAAGGATGACGGCACCTACAAG
ACCCGCGCTGAGGTGAAGTTTGAGGGCGATACCCTGGTGAACCGCATCGAGCTG
AAGGGCATCGACTTTAAGGAGGATGGCAACATCCTGGGCCACAAGCTGGAGTAC
AACTATAACAGCCACAACGCGTACATCACCGCCGACAAGCAGAAGAACGGCATC
AAGAGCAACTTCAAGATCCGCCACAACGTGGAGGATGGCTCCGTGCAGCTGGCC
GACCACTACCAGCAGAACACCCCAATCGGCGATGGCCCGgtgCTGCTGCCAGaC
AACCACTACCTGAGCACCcAGAGCAAGCTGAGCAAGGACCcGAACGAGAAGCGC
GACCACATGGTGCTGCTGGAGTTTGTGACCGCTGCTGGCATCACCCACGGCATG
GATGAGCTGTACAAGGGATCCGGCTGCTAA;

- **22**: rsEGFP2 V151L.
ATGGTGAGCAAGGGCGAGGagCTGTTCACCGGGGTGGTGCCCATCCTGGTCGAG
CTGGACGGCGACGTAAACGGCCACAAGTTCAGCGTGTCCGGCGAGGGCGAGGGC
GATGCCACCTACGGCAAGCTGACCCTGAAGTTCATCTGCACCACCGGCAAGCTG
CCCGTGCCCTGGCCCACCCTCGTGACCACCCTGGCCTACGGCGTGCTGTGCTTC
AGCCGCTACCCCGACCACATGAAGCAGCACGACTTCTTCAAGTCCGCCATGCCC
GAAGGCTACGTCCAGGAGCGCACCATCTTCTTCAAGGACGACGGCAACTACAAG

```
ACCCGCGCCGAGGTGAAGTTCGAGGGCGACACCCTGGTGAACCGCATCGAGCTG
AAGGGCATCGACTTCAAGGAGGACGGCAACATCCTGGGGCACAAGCTGGAGTAC
AACTACAACAGCCACAACCTCTATATCATGGCCGACAAGCAGAAGAACGGCATC
AAGTCTAACTTCAAGATCCGCCACAACATCGAGGACGGCAGCGTGCAGCTCGCC
GACCACTACCAGCAGAACACCCCCATCGGCGACGGCCCCGTGCTGCTGCCCGAC
AACCACTACCTGAGCACCCAGTCCAAGCTGAGCAAAGACCCCAACGAGAAGCGC
GATCACATGGTCCTGCTGGAGTTCGTGACCGCCGCcGGgATCACTCTCGGCATG
GACGAGCTGTACAAGTAA.
```

1.2 Characterization of the optical setup

Myriads of illuminations can be considered for LIGHTNING implementation. In order to minimize the acquisition time, it is necessary to recognize the minimal set of illuminations, which generate time series of the RSFs fluorescence signal under control of different steps of their photocycle over a given kinetic window. To address this concern with RSFPs, we designed and built a specific optical instrument , which we denominated "photoswitchometer". Equipped with synchronized detectors, it integrates two sets of light sources (Light Emitting Diodes – LEDs – and lasers for low and high regimes of light intensities respectively) at the wavelengths $\lambda_1 = 488$ nm and $\lambda_2 = 405$ nm, which have been spectrally tuned to photoswitch green RSFPs. Two optical configurations of the photoswitchometer have been designed and built during this work:

- The first configuration was devoted to acquire the kinetic information on the photoswitching properties of the RSFPs (see Supplementary Figure 1a). In this configuration, the photoswitchometer automatically records and analyzes in a few minutes the fluorescence response from a RSFP solution exposed to homogeneous illumination at a series of light intensities covering 5 orders of magnitude (up to a few hundreds of ein.m^{-2}.s^{-1} – 10^4 W/cm^2 range) with microseconds time resolution. This configuration was subsequently used to evaluate the LIGHTNING discrimination power on solutions of RSFPs by fixing the illuminations at tailored light intensities;
- The second configuration was optimized to provide images of RSFP-labeled bacteria with a 20 μm-diameter circular field of view at 1 kHz frequency of image acquisition by fixing the illuminations at tailored light intensities (see Supplementary Figure 1b).

1.2.1 Configuration for acquiring the RSFP photoswitching information

Temporal characterization of the illumination profiles To characterize the temporal resolution of the photoswitchometer, we recorded the time response of the signal detected by the photomultiplier upon switching on and off the light sources. Supplementary Figure 2a–d display the results. The time resolution of the photoswitchometer is limited by the time resolution of the photomultiplier when switching on and off the laser light sources at both λ_1= 488 nm and λ_2= 405 nm ($\simeq 1$ μs). In contrast, the time resolution of the photoswitchometer is limited by the light sources when using the LEDs. Switching on and off the light sources respectively generates signal changes at 9.6 and 1.7 μs time scales at λ_1= 488 nm (respectively 5.7 and 1.1 μs at λ_2= 405 nm).

Spatial characterization of the illumination profiles

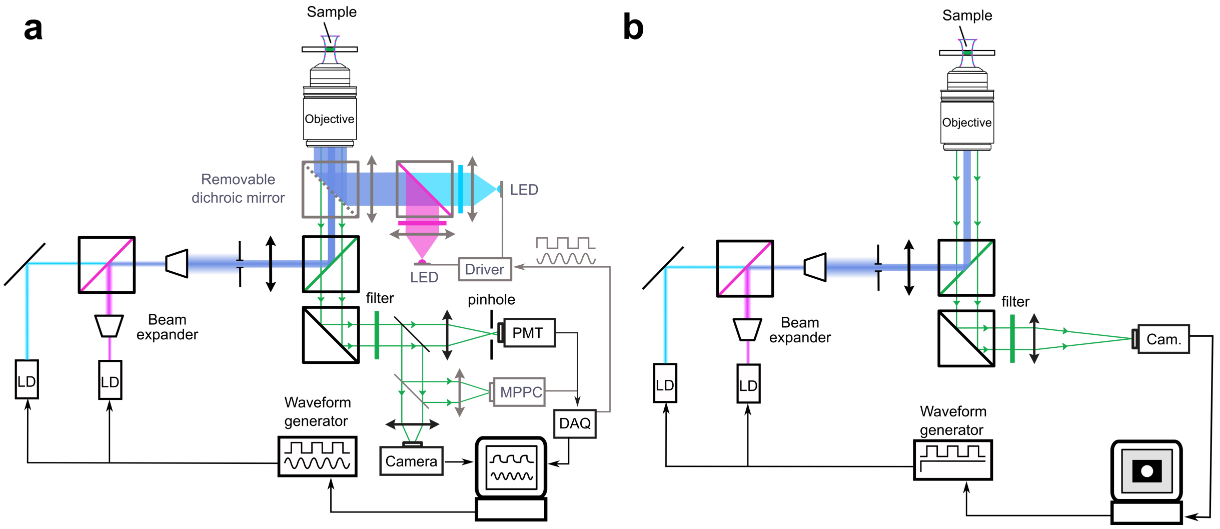

Supplementary Figure 1: Schemes of the photoswitchometer designs for automated acquisition of the photoswitching kinetic information of RSFPs in liquid solutions (**a**) and LIGHTNING imaging (**b**). Light-emitting diodes and lasers diode modules are controlled with a waveform generator or the analog outputs of a digital acquisition card (DAQ) and deliver sequences of modulated homogeneous illumination at the sample with 1–10^5 W/cm^2 intensities and 1–10^6 Hz frequencies at $\lambda_1 = 488$ nm and $\lambda_2 = 405$ nm. Fluorescence is retrieved with synchronized detectors (**a**) or with a synchronized sCMOS camera (**b**). The components involving LEDs and lasers for illuminations are displayed in gray and black respectively. DAQ: digital acquisition unit; MPPC: multipixel photon counter; PMT: photomultiplier tube; LD: Laser diode.

LEDs To characterize the illumination profiles from the LEDs, we imaged at $\lambda_1 = 488$ nm and λ_2= 405 nm with a 24 mm objective a 5 μM fluorescein solution in pH 7.4 PBS buffer embedded in a 3 mm-thick agarose gel. Supplementary Figure 3a,b show that the LED illumination profiles are homogeneous over 2 mm along the z axis of a 0.5 mm-radius cylinder.

Lasers In order to evaluate the homogeneity of the cylindrical illumination profiles from the lasers and determine their dimensions, we imaged a thin layer of 10 μM fluorescein solution in pH 7.4 PBS buffer (sandwiched between two glass plates; 5 μm of thickness) at different axial positions ranging from 0 to 125 μm by means of a microscope composed of a 20x objective, a tube lens, an emission filter, and a IDS camera. For each axial position, we measured the diameter of the illuminated area. For the setup displayed in Supplementary Figure 1a, Supplementary Figure 4a,b show that the illuminated area is rather homogeneous at 0 μm. Furthermore the 488 and 405 nm illumination profiles at 0 and 125 μm displayed in Supplementary Figure 4c,d suggest that laser illumination at $\lambda_1 = 488$ nm and $\lambda_2 = 405$ nm is homogeneous along a cylindrical profile of height $h = 125$ μm and radius $r_0 = 10$ μm.

1.2.2 Configuration for acquiring the LIGHTNING images

Spatial characterization of the illumination profiles In order to evaluate the homogeneity and the dimensions of the cylindrical illumination profiles from the lasers at the focal plane, we imaged a thin layer of 10 μM fluorescein solution in pH 7.4 PBS buffer sandwiched between two glass plates (5 μm of thickness, which is representative of the thickness of the bacteria samples). Supplementary Figure 5a,b show that the illuminated area is rather homogeneous under illumination at 488 and 405 nm: both light distributions exhibit spatial uniformity with a fluctuation of less than 10% over a disk of 20 μm diameter in the focal plane. Supplementary Figure 5c,d display the histograms of

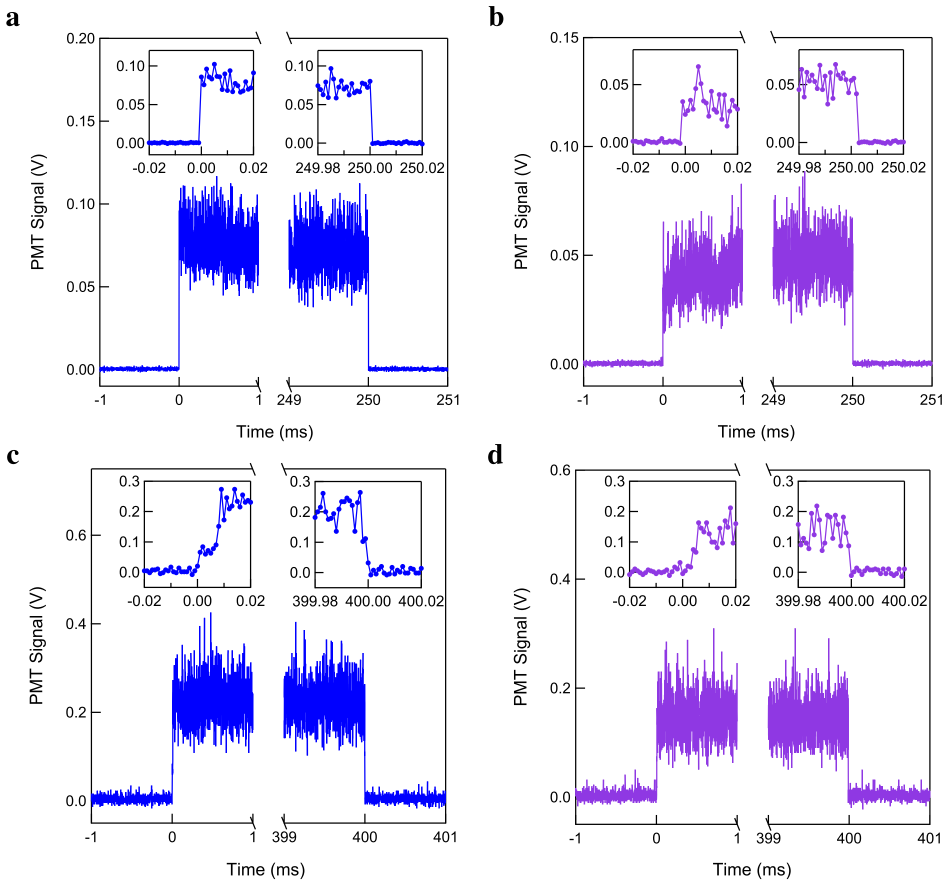

Supplementary Figure 2: *Characterization of the temporal resolution of the photoswitchometer.* Time response of the signal detected by the photomultiplier upon switching on and off the lasers (**a**,**b**) and the LEDs (**c**,**d**) at λ_1= 488 nm (**a**,**c**) and λ_2= 405 nm (**b**,**d**).

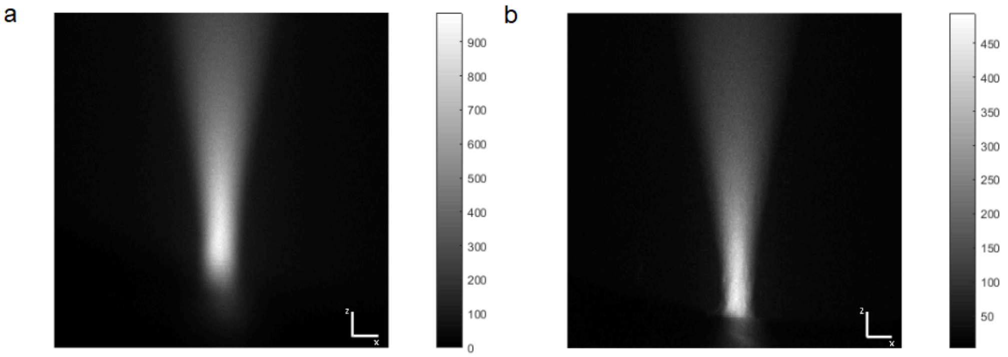

Supplementary Figure 3: *Characterization of the illumination profile from the LEDs in the photoswitchometer configuration for acquiring the RSFP photoswitching information.* **a**: λ_1= 488 nm; **b**: λ_2= 405 nm. Light intensity with linear grey scale in arbitrary units. Scaling bar: 1 mm.

the decimal logarithm of the light intensities at the image pixels over a region of interest of 10^4 pixels. The standard deviations associated to the distributions of the decimal logarithm of the light intensities at $\lambda_1 = 488$ nm and $\lambda_2 = 405$ nm are equal to 0.07 and 0.04 respectively.

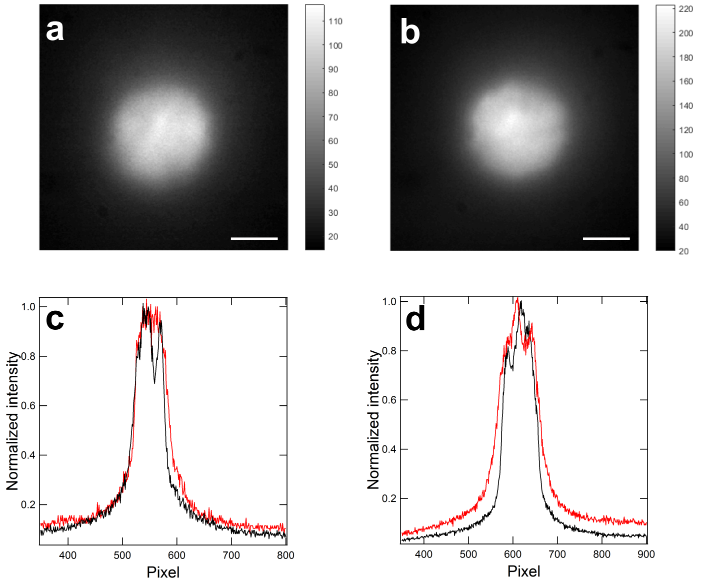

Supplementary Figure 4: *Characterization of the illumination profiles from the lasers in the photoswitchometer configuration for acquiring the RSFP photoswitching information*. **a**,**b**: Homogeneity of the laser illumination as evidenced by mapping the fluorescence emission from 5 μM fluorescein solution in pH 7.4 PBS buffer at $\lambda_2 = 405$ nm (**a**) and $\lambda_1 = 488$ nm (**b**) at $z = 0$ μm. Light intensity with linear grey scale in arbitrary units. Scaling bar: 10 μm; **c**,**d**: Laser illumination profile as evidenced from fluorescence emission from 5 μM fluorescein solution in pH 7.4 PBS buffer imaged at $\lambda_2 = 405$ nm (**c**) and $\lambda_1 = 488$ (**d**) for $z = 0$ (black line) or $z = 125$ (red line) μm.

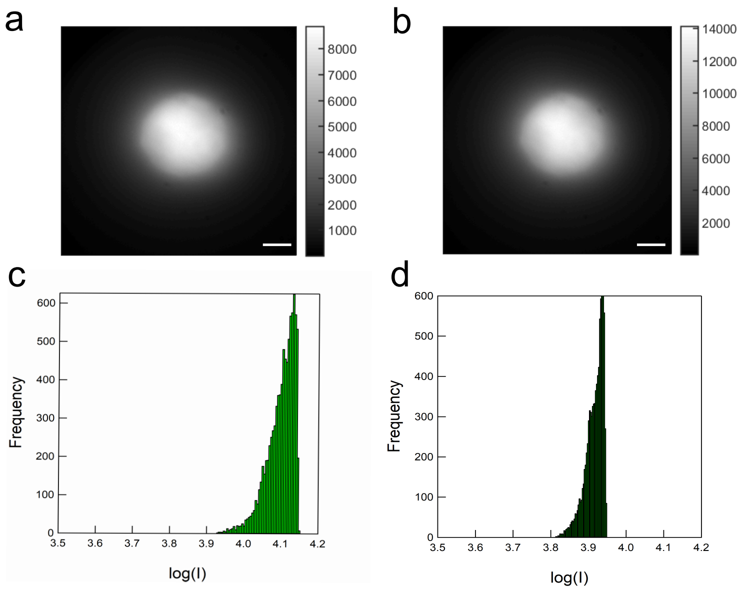

Supplementary Figure 5: *Characterization of the illumination profiles from the lasers in the focal plane of the photoswitchometer in its configuration for LIGHTNING imaging*. **a**,**b**: Homogeneity of the laser illumination as evidenced by mapping the fluorescence emission from a 5 μm-thick layer of 10 μM fluorescein solution in pH 7.4 PBS buffer imaged at $\lambda_1 = 488$ nm (**a**) and $\lambda_2 = 405$ nm (**b**) at the focal plane. Light intensity with linear grey scale in arbitrary units. Scaling bar: 10 μm; **c**,**d**: Distributions of the decimal logarithm of the light intensities at $\lambda_1 = 488$ nm (**c**) and $\lambda_2 = 405$ nm (**d**) at the pixels of the images recorded in **a** and **b** respectively.

1.2.3 Calibration of light intensities

LEDs We relied on the photoswitching kinetics of a photochemically well-characterized RSFP (Dronpa-2 at pH 7.4) for measuring light intensities at which the kinetics is well-accounted with a two-state model (see section A.2). We could show that such an actinometric measurement is photochemically reliable up to 10 ein.m^{-2}.s^{-1} light intensities at the wavelengths $\lambda_1 = 488$ nm and $\lambda_2 = 405$ nm respectively.

The calibration of the light intensities exploits light jumps on Dronpa-2 samples (typically a 10 μM Dronpa-2 solution in pH 7.4 PBS buffer):

- As a preliminary step of the calibration protocol, the Dronpa-2 solution is illuminated with the 405 nm LED ($I_2 = 0.01$ ein.m^{-2}.s^{-1} for 2 min) to secure that Dronpa-2 is initially in its thermodynamically stable state C_1;

- Then the sample is illuminated at constant light intensity I_1 at $\lambda_1 = 488$ nm (this first illumination is associated with the exponent I) and the evolution of the fluorescence signal is collected. The decay is adjusted by:

$$I_F^{\mathrm{I}} = I_F^{\mathrm{I}}(0) + A^{\mathrm{I}}\left[1 - \exp\left(-\frac{t}{\tau^{\mathrm{I}}}\right)\right] \tag{1}$$

  The time τ^{I} is used as a fitting parameter and the intensity I_1 is retrieved according to (see Eq.(43))

$$I_1 = \frac{\frac{1}{\tau^{\mathrm{I}}} - k_{21}^{\Delta}}{\Sigma_1} \tag{2}$$

  where the reference values of the rate constant for thermal return after reversible fluorescence photoswitching ($k_{21}^{\Delta} = 0.014$ s^{-1}) and the sum of the photoswitching cross sections ($\Sigma_1 = 196$ m^{2}mol^{-1}) at the wavelength λ_1 are given in reference;[7]

- In a second step, while maintaining constant illumination I_1 at the wavelength $\lambda_1 = 488$ nm, the sample is submitted to a light jump of intensity I_2 at the wavelength $\lambda_2 = 405$ nm (this second illumination protocol is associated with the exponent II). The exponential fluorescence recovery is recorded and adjusted using

$$I_F^{\mathrm{II}} = I_F^{\mathrm{II}}(0) + A^{\mathrm{II}}\left[1 - \exp\left(-\frac{t}{\tau^{\mathrm{II}}}\right)\right] \tag{3}$$

  where the time τ^{II} is used as a fitting parameter and the intensity I_2 can be retrieved from (see Eq.(52))

$$I_2^{\mathrm{II}} = \frac{\frac{1}{\tau^{\mathrm{II}}} - \Sigma_1 I_1 - k_{21}^{\Delta}}{\Sigma_2} \tag{4}$$

  where the reference value of the sum of the photoswitching cross sections ($\Sigma_2 = 413$ m^{2}mol^{-1}) at the wavelength λ_2 is given in reference.[7]

Lasers The calibrations of the light from the lasers were performed with a power meter (PM100A, Thorlabs, NJ, US) by measuring the dependence of the laser output power on the applied input (tension voltage – in V – for the 488 nm laser and input power – in mW – for the 405 nm laser). We observed a linear dependence (i) with the 488 nm laser source between 0.12 and 4.6 V (see Supplementary Figure 6a) and (ii) with the 405 nm laser diode between 2 mW and 100 mW (see Supplementary Figure 6b) and adopted the corresponding ranges to perform all the subsequent experiments.

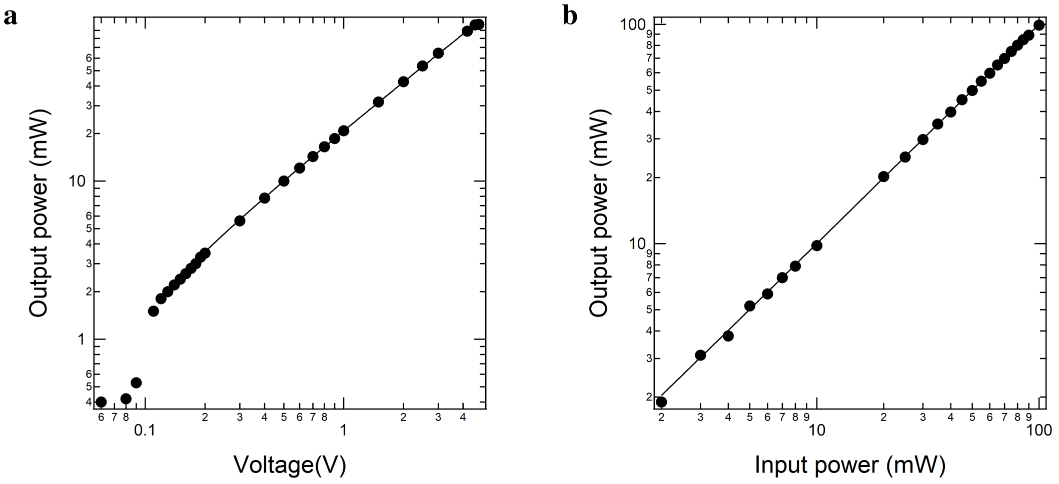

Supplementary Figure 6: *Calibration of the output power of the lasers*. Dependence of the laser output power on the applied input (tension voltage – in V – for the 488 nm laser – **a** – and input power – **b**, in mW – for the 405 nm laser).

In particular, these calibrations have been used to validate the continuity of the illumination when switching from the LEDs to the lasers for illumination since the maximum of LED intensity matches the lower laser intensity once the later is decreased with an optical absorptive density (NE10A-A Thorlabs, Newton, NJ, OD=1).

1.3 Protocols of acquisition of the RSFP photochemical behavior for LIGHTNING implementation

The protocol of acquisition of the RSFP photochemical behavior in RSFP solutions includes: (i) Recording the time series of the RSFP fluorescence signal under several illumination conditions at different light intensities; (ii) Extracting a single characteristic time from each time evolution of the RSFP fluorescence signal; (iii) Analyzing the dependence of the inverse of the characteristic time in each illumination condition on the associated light intensities.

Two illuminations denoted I and II have been used to characterize the photoswitching kinetics of the RSFPs. The photoswitchometer applies constant light at the wavelength $\lambda_1 = 488$ nm and periodic square-wave light at the wavelength $\lambda_2 = 405$ nm during three periods (see Supplementary Figure 7). The light at $\lambda_2 = 405$ nm is turned off during the first half periods, which generates the time windows of illumination I involving constant light at $\lambda_1 = 488$ nm. In contrast, it is turned on during the second half periods, which generates the time windows of illumination II involving constant lights at both $\lambda_1 = 488$ nm and $\lambda_2 = 405$ nm. The fluorescence evolution is recorded at 525 nm. The photoswitchometer has been automated to enable the fast screening of the green RSFPs. Data acquisition is automated with LabVIEW and includes the external control of the outputs (square wave, cycle duration, average voltage, duty cycle,...) of the two waveform generators.

Data acquisition has proceeded in two steps:

- In a preliminary step, we determined the time window over which the two-color photoactivation of the RSFPs is fully reversible. Illuminations I and II were applied using strong LED lights (Supplementary Figure 17–Supplementary Figure 20). In order to roughly delimit two kinetic regimes involving fast and slow variations of the fluorescence signal, the fluorescence evolution I_F^{i} observed for the two illuminations $i = \mathrm{I}, \mathrm{II}$ has been analyzed using an unsupervised biexponential fitting function over the whole time window

$$I_F^{\mathrm{i}}(t) \quad = \quad A^{\mathrm{i}}_{fast} \exp(-t/\tau^{\mathrm{i}}_{fast}) + A^{\mathrm{i}}_{slow} \exp(-t/\tau^{\mathrm{i}}_{slow}) + I_F^{\mathrm{i}}(\infty) \tag{5}$$

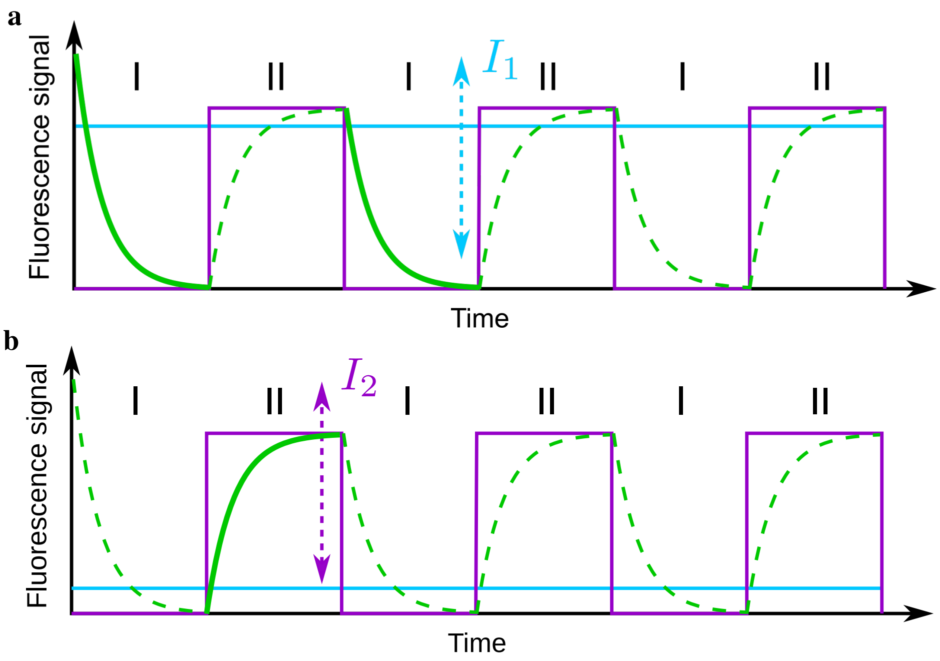

Supplementary Figure 7: *Illumination experiments devoted to the photochemical characterization of the RSFPs*. The photoswitchometer records the fluorescence evolution upon applying three periods of illumination, which involves constant light at the wavelength $\lambda_1 = 488$ nm (blue) and square-wave light at the wavelength $\lambda_2 = 405$ nm (purple) by using either LEDs or lasers. The first (resp. second) half period corresponds to illumination I (resp. II). Two series of photochemical experiments have been performed (see Supplementary Figure 21–Supplementary Figure 42a,b): **a**: Variable light intensity I_1 at the wavelength $\lambda_1 = 488$ nm and fixed light intensity at the wavelength $\lambda_2 = 405$ nm. Among the three periods of fluorescence acquisition (dashed green line), the first (for LIGHTNING imaging) or third (for acquisition of the photoswitching kinetic information of RSFPs) half periods (solid green line) under illumination I have been analyzed; **b**: Fixed light intensity at the wavelength $\lambda_1 = 488$ nm and variable light intensity I_2 at the wavelength $\lambda_2 = 405$ nm. Among the three periods of fluorescence acquisition (dashed green line), the second half period (solid green line) under illumination II is analyzed.

The values of the fast and slow relaxation times, τ^{i}_{fast} and τ^{i}_{slow}, have been extracted. They are in line with fast reversible photoswitching and slow long-term decay of fluorescence,[8,9] leading to an estimate of the associated cross sections (see Eq.(43) for i = I, Eq.(52) for i = II; compare the cross sections $\Sigma_{1,fast}$ and $\Sigma_{2,fast}$ in Supplementary Table 2 and Σ_1 and Σ_2 in Supplementary Table 3). For all the RFSPs and both illuminations, the ratios $\Sigma_{j,fast}/\Sigma_{j,slow}$ were found greater than 10^3. Hence we could define a wide time window in which reversible photoswitching is not perturbed by long-term decay of fluorescence;

- In a second step, the two illuminations I and II involving LED and laser lights were used to establish the RSFP photoswitching signature for several tens of light intensities in a broad 10^{-3}–10^2 ein.m^{-2}.s^{-1} range (see Supplementary Figure 21–Supplementary Figure 42a,b). At all investigated LED intensities, the time window acquisition is both larger than a few times the order of magnitude of the fast relaxation time τ^{i}_{fast} deduced in the preliminary step and narrow enough to neglect long-term decay of fluorescence. The value of the half-period was scaled proportionally to the inverse of light intensity upon laser illumination for diffusion to remain negligible in the time evolution of the fluorescence signal (see section A.2.2) (see Supplementary Figure 21–Supplementary Figure 42c–f). In the course of our experiments, we noticed that the fluorescence decay observed for illumination I during the first half period exhibited a larger amplitude for high I_1 intensities than during the following odd half periods. The detailed interpretation of this behavior is left for another publication. In the present report,

we processed the data from the first and the third half periods for three RSFPs (**1**, **2**, and **6** exhibiting distinct photochemical behavior). Supplementary Figure 8a–c show that despite the difference of their amplitudes, the fluorescence decays observed during the first and the third half periods of application of illumination I exhibit similar characteristic times so as to be equally used to establish the kinetic fingerprint of the RSFPs. Whereas the third half period has been used for data processing during our subsequent experiments to be at steady-state, the first half period was used for LIGHTNING imaging of RSFPs in order to get a better signal-to-noise-ratio.

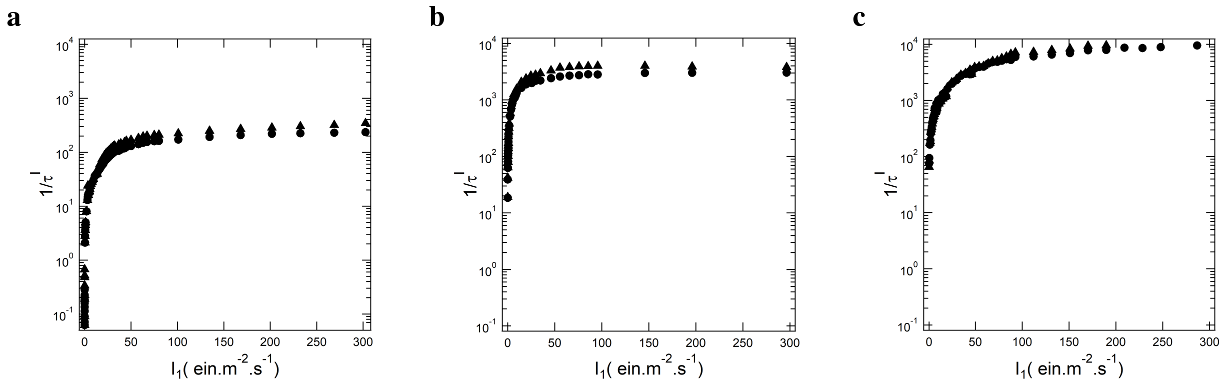

Supplementary Figure 8: Dependence of the inverse of the characteristic times $1/\tau^I$ of **1** (**a**), **2** (**b**), and **6** (**c**) obtained from analyzing the fluorescence decays during the first (triangle) and the third (disk) half periods of application of illumination I.

1.4 Data processing for LIGHTNING implementation

The analysis of the fluorescence evolution (see Section A.1) used a robust protocol to determine the characteristic times. The monoexponential reduction of fluorescence evolution regardless of the complexity of kinetics is described in Section B.2 and data analysis was automated using Igor Pro.

The fluorescence evolution under illumination I was monotonous for all RSFPs. We first used the unsupervised monoexponential fitting function (given in Eq.(40) for $\mathrm{I_{low}}$) over the whole acquisition window to extract a first guess of the characteristic time τ^{I}. We then restricted the time window to [0, $5\tau^{\mathrm{I}}$] in order to extract the final τ^{I} values using the same monoexponential fitting function. For illumination II, we used the same protocol as for illumination I when the fluorescence evolution was monotonous. When the fluorescence evolution displayed an extremum, we first restricted the lower and upper limits of the analyzed time window to the times respectively associated with the initial and extremum values of the fluorescence signal. Then we proceeded by applying the monoexponential fitting function (given in Eq.(49) for $\mathrm{II_{low}}$) over the delimited time window in order to extract the value of τ^{II}. Data processing ended up with plotting the inverse of the photoswitching relaxation times $1/\tau^{\mathrm{I}}$ versus the light intensity I_1 at the wavelength $\lambda_1 = 488$ nm and $1/\tau^{\mathrm{II}}$ versus the light intensity I_2 at the wavelength $\lambda_2 = 405$ nm (see Supplementary Figure 21–Supplementary Figure 42c–f).

In the regime of low light intensities, the fluorescence evolution is satisfactorily accounted by a two-state kinetic model (see section A.2). The reaction rate being limited by the photochemical step, $1/\tau^{\mathrm{i}}_{low}$ for $i = \mathrm{I}, \mathrm{II}$ linearly depends on light intensity (see Eqs.(43,52)). The slope of $1/\tau^{\mathrm{I}}_{low}$ versus I_1 provides the cross section Σ_1 and the slope of $1/\tau^{\mathrm{II}}_{low}$ versus I_2 provides the cross section Σ_2. In a high intensity regime where thermal steps impact the reaction rate, the inverse of the relaxation times $1/\tau^{\mathrm{I}}_{high}$ and $1/\tau^{\mathrm{II}}_{high}$ exhibit no or a weaker dependence on the light intensities. The cutoff intensity I_1^c (resp. I_2^c) delimiting the two regimes was defined as the intensity for which the inverse of the characteristic time $1/\tau^{\mathrm{I}}$ (resp. $1/\tau^{\mathrm{II}}$) departs from 33% (resp. 20%) from the prediction of the two-state model $\Sigma_1 I_1^c$

(resp. $\Sigma_1 I_1^c + \Sigma_2 I_2^c$). The overall results reporting on the photochemical behavior of the 22 investigated RSFPs for LIGHTNING implementation are provided in Supplementary Table 3.

1.5 Acquisition of the LIGHTNING kinetic fingerprints of the RSFPs

Due to their different light intensity dependence (see section C), the characteristic times acquired under illuminations I and II in the regimes of low- and high-light intensity are non-redundant for kinetic discrimination. As a consequence any set of four illuminations I and II in their respective regimes of low- and high-light intensity is relevant to generate a discriminative set of characteristic times for LIGHTNING application. We here retained four illuminations $\{\mathrm{I}_{\mathrm{low}}, \mathrm{II}_{\mathrm{low}}, \mathrm{I}_{\mathrm{high}}, \mathrm{II}_{\mathrm{high}}\}$ associated with light intensity enabling us to achieve each regime for all RSFPs while minimizing the acquisition duration and defined the RSFP LIGHTNING kinetic fingerprint as the set of four characteristic times $\{\tau^{\mathrm{I}}_{\mathrm{low}}, \tau^{\mathrm{II}}_{\mathrm{low}}, \tau^{\mathrm{I}}_{\mathrm{high}}, \tau^{\mathrm{II}}_{\mathrm{high}}\}$ obtained for the ordered sequence $\{\mathrm{I}_{\mathrm{low}}, \mathrm{II}_{\mathrm{low}}, \mathrm{I}_{\mathrm{high}}, \mathrm{II}_{\mathrm{high}}\}$:

- For the solutions of the 22 investigated RSFPs with the photoswitchometer in the configuration displayed in Supplementary Figure 1a, we adopted the values of light intensities $I_1 = 2$ ein.m^{-2}.s^{-1} for the regime I_{low}, $I_1 = 0.1$ ein.m^{-2}.s^{-1} and $I_2 = 0.1$ ein.m^{-2}.s^{-1} for the regime II_{low}, $I_1 = 200$ ein.m^{-2}.s^{-1} for the regime I_{high}, and $I_1 = 2$ ein.m^{-2}.s^{-1} and $I_2 = 90$ ein.m^{-2}.s^{-1} for the regime II_{high}. Supplementary Table 7 sums up the results;

- For imaging the RSFP-labeled *Escherichia coli* with the photoswitchometer in the imaging configuration displayed in Supplementary Figure 1b. First, a high quality movie used for image segmentation was recorded at 0.1 kHz upon low-light illumination. Then two binned movies have been recorded under low- and high-light illuminations. For the low light movie, we applied sequences of illuminations I and II with the values of light intensities: $I_1 = 2$ ein.m^{-2}.s^{-1} for the illumination I_{low}, $I_1 = 2$ ein.m^{-2}.s^{-1} and $I_2 = 0.2$ ein.m^{-2}.s^{-1} for the illumination II_{low}. For the high light movie, we applied sequences of illuminations I and II with the values of light intensities: $I_1 = 50$ ein.m^{-2}.s^{-1} for the illumination I_{high}, and $I_1 = 50$ ein.m^{-2}.s^{-1} and I_2 =20 ein.m^{-2}.s^{-1} for the illumination II_{high}. Supplementary Table 13 sums up the results.

1.6 Processing of the LIGHTNING images of RSFP-labeled bacteria

All the movies were processed and analysed with in-house routines written in Python 3 (the codes are available on Github[1]) using the scikit-image library.[10] We used the high quality video as a reference to perform the segmentation of the bacteria, and the binned movies to extract the time evolution of the fluorescence from the RSFP-labeled bacteria under the different illuminations.

1.6.1 Segmentation of bacteria

For all acquired movies, we assumed that the bacteria positions did not vary throughout the recording. Hence we first selected and summed frames with high dynamics and contrast from the high-quality movie to generate an information-rich single reference image F_r (Supplementary Figure 9a). More precisely, we computed $S_i = \sum_{x_i,y_i} p_{x_i,y_i}$ in the image i for a movie of n frames ($i \in I = [1, ..., n]$) where x_i, y_i designate the coordinates of the pixels with signal level p_{x_i,y_i}. After identifying $S_{max} = \max\{S_i\}$, we extracted the set $M = \{i, S_i > 0.8 \times S_{max}\}$. The reference image r, $p_{x_r,y_r} = \sum_{i \in M} p_{x_i,y_i}$, is the sum of the images of the M set.

[1]The codes for processing the images are available at https://github.com/Alienor134/image_segmentation. See also https://doi.org/10.5281/zenodo.5684342.

The first step of the segmentation was to process the reference image F_r by applying operations based on mathematical morphology:[11,2] (i) the *rank.enhance_contrast* filter on the one hand. We obtained a first image with enhanced contrast of the bacteria against the background (Supplementary Figure 9b); (ii) the *rank.autolevel* filter on the other hand.[3] We retrieved a second image with sharpened frontiers of the bacteria against the background (Supplementary Figure 9c). Both grey-level images were binarised by using the local threshold method of Otsu (Supplementary Figure 9d,e).[12] The intersection of the generated two masks was used to generate a final mask sharpening the distinction between the background and the bacteria (Supplementary Figure 9f): this mask is white on the pixels where bacteria are present and black on the background pixels. The new reference image for the segmentation (Supplementary Figure 9g) was obtained from hiding the reference image F_r behind the final mask in Supplementary Figure 9f.

When the bacteria were isolated in the reference image F_r, the white areas in Supplementary Figure 9f corresponded to single bacteria with a single maximum (brightest point of the segmented area, displayed in Supplementary Figure 10a). In contrast, when bacteria were grouped (e.g. two bacteria emerging from division of a mother bacteria), the white areas corresponded to multiple cells with multiple maxima. To further split these white areas, we applied the Watershed Segmentation method[11] on Supplementary Figure 9g,[4] which resulted in the image displaying segmented individual bacteria in Supplementary Figure 10b.

The parameters of the segmentation algorithm are the size of the kernels and the thresholds selected for binarization. We tested several sets of parameters and selected a common set that segmented most objects in all of the experiments, without selecting parts of the background, and with a granularity of segmentation corresponding to the bacteria size. The segmentation can miss bacteria whose fluorescence expression level is low in each set of bacteria. We detected an overall low expression level for bacteria expressing protein **2**, **16** and **21**, which resulted in poor segmentation.

1.6.2 Registration of bacteria

The image processing of the high-quality video allowed to segment each bacteria individually on the high-quality movie. They had to be subsequently identified in the binned movies acquired at high frame-rate under the regimes of low- and high-light illuminations I and II. To spatially associate the bacteria to their position in each binned video, we first cross-correlated a down-scaled version of F_r (obtained by binning 4 pixels in the F_r image; see Supplementary Figure 11a) and the first frame of the binned movie (Supplementary Figure 11b). Then the segmented pixels in the high-quality image were attributed to the binned pixels (see Supplementary Figure 11c).[5] To avoid signal mixing, we subsequently discarded binned pixels corresponding to areas where two bacteria were present in the high definition image (see Supplementary Figure 11d).

[2]Morphological image processing relies on the use of kernels - small patches of pixels - to modify the features in the image. One way to visualize morphological image processing is to consider the image as a 3D landscape where the value of a pixel represents its elevation in the third dimension. Morphological operations would consist in modifying the elevation locally with small tools like shovels and pickaxes. For all the operations we used (see Supplementary Figure 9), the parameters were the size and shape of the kernel – a disk of radius varying between 2 pixels and 7 pixels in our case.

[3]The auto-leveling consists in stretching the histogram of the pixels covered by the kernel to locally increase the dynamics.

[4]The Watershed Segmentation method consists in flooding the negative of the image Supplementary Figure 9g from the position of the local maxima.

[5]We observed slightly different shifts in the two movies recorded under low- and high-light illuminations. Therefore, a bacterium segmented in the high-quality movie might be represented by $k_{N,low}$ pixels in the low-light movie and by $k_{N,high}$ pixels in the high-light movie, with $k_{N,low} \neq k_{N,high}$.

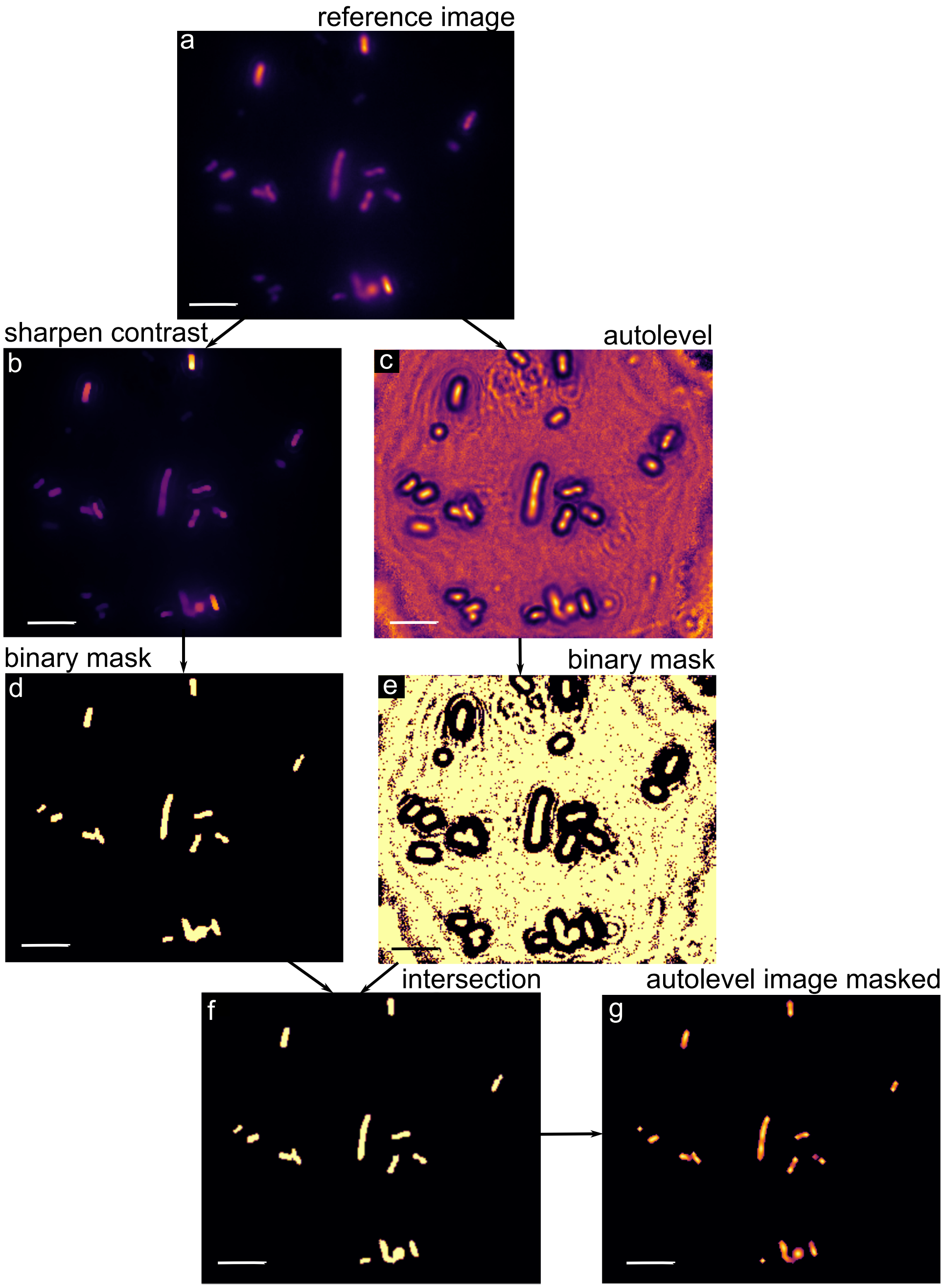

Supplementary Figure 9: **a**: Reference image F_r; **b**, **d**: Application of the *rank.enhance_contrast* filter on the reference image F_r to yield an image with enhanced contrast (**b**), which is subsequently binarized (**d**); **c**, **e**: Application of the *rank.autolevel* filter on the reference image F_r to yield an image with sharpened frontiers of the bacteria (**c**), which is subsequently binarized (**e**); **f**: Intersection of the images **d** and **e**; **g**: Image obtained by masking the image **c** with the intersection mask **f**. Scaling bar = 5 μm.

1.6.3 Selection criteria

The segmentation allowed us to collect the fluorescence value at each pixel belonging to a bacteria in each frame. For each bacterium numbered N that was identified on k_N pixels, we exploited the time response of the fluorescence signal to generate a table of k_N columns (one for each pixel) with as many lines as recorded frames. In each column, we identified the phases corresponding to illumination j = I and j = II and fitted the time course of the fluorescence

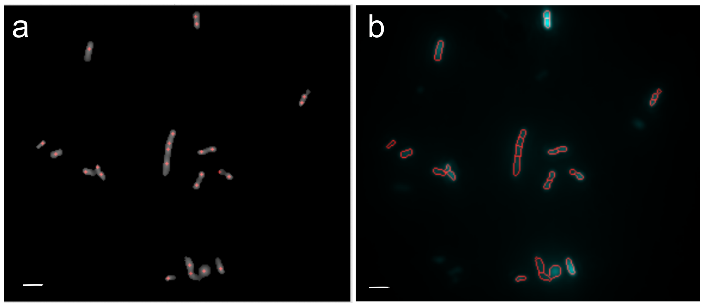

Supplementary Figure 10: **a**: Image exhibiting the positions of the local maxima, source of flooding by the Watershed Segmentation method; **b**: Resulting segmented contours of the items. Scaling bar = 5 μm.

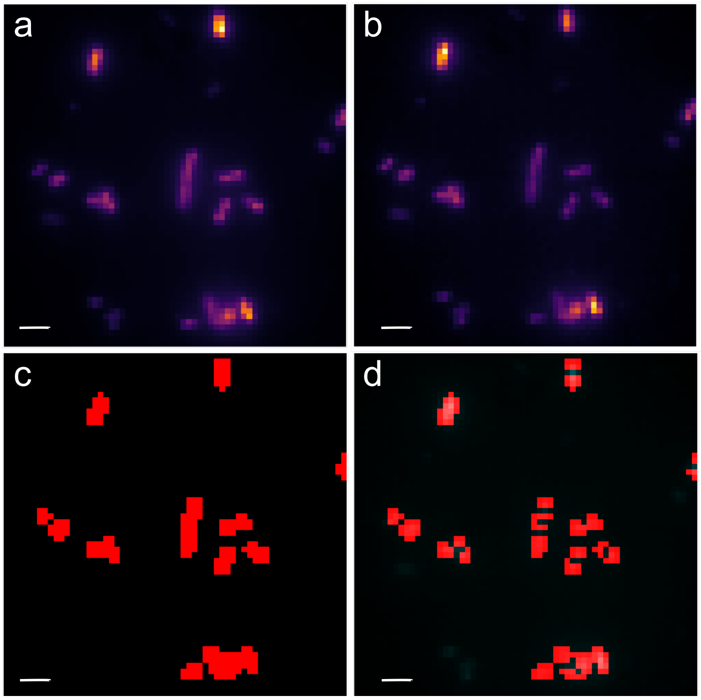

Supplementary Figure 11: **a**: Downscaled version of the reference image F_r after 4×4 binning; **b**: First image of a binned video acquired at high frame-rate; **c**: Segmented pixels on the binned image; **d**: Segmented pixels without bacteria overlaps. Scaling bar = 5 μm.

signal $I^{\mathrm{j}}_{F,N,i}$ ($i \in [1; k_N]$), with a 3-parameter exponential function given in Eq.(6)

$$I^{\mathrm{j}}_{F,N,i}(t) \quad = \quad A^{\mathrm{j}}_{N,i} exp(-t/\tau^{j}_{N,i}) + I^{\mathrm{j}}_{F,N,i}(\infty) \tag{6}$$

to retrieve a first estimate of $\tau^{j}_{N,i}$. Fitting has been first applied over the whole time window. In a second step, the time window for fitting has been adjusted to five times the value of $\tau^{j}_{N,i}$, and fitting with Eq.(6) has been reproduced to extract the final value of $\tau^{j}_{N,i}$.

Then we computed the mean τ^{j}_{N} and the normalized range $range^{\mathrm{j}}_{N,norm}$ of the values of $\tau^{\mathrm{j}}_{N,i}$ retrieved from the

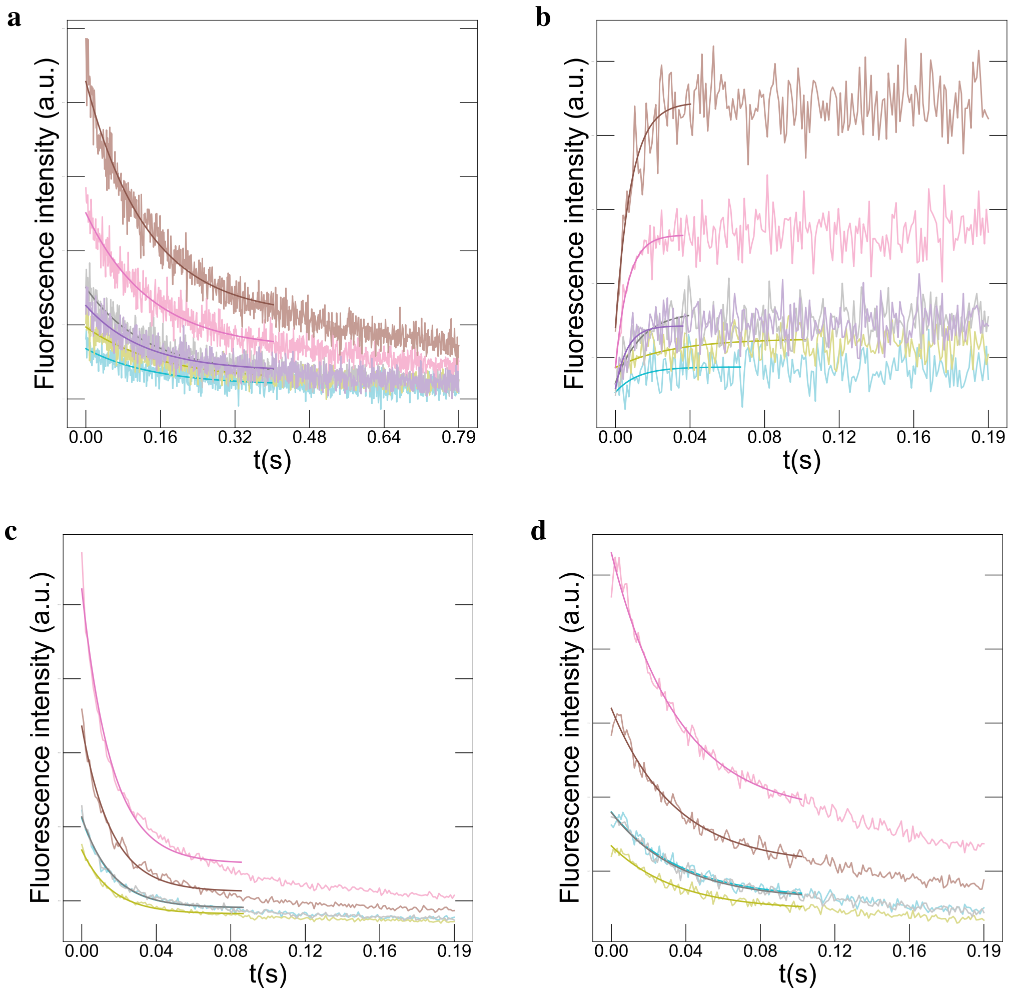

Supplementary Figure 12: Kinetic analysis of the pixels of a bacterium labeled with **9** submitted to the four illuminations I_{low} (**a**; $I_1 = 2$ ein.m^{-2}.s^{-1}), II_{low} (**b**; $I_1 = 2$ ein.m^{-2}.s^{-1} and $I_2 = 0.2$ ein.m^{-2}.s^{-1}), I_{high} (**c**; $I_1 = 50$ ein.m^{-2}.s^{-1}), and II_{high} (**d**; $I_1 = 50$ ein.m^{-2}.s^{-1} and $I_2 =$20 ein.m^{-2}.s^{-1}). T = 298 K.

pixels of each bacterium N

$$\tau_N^{\mathrm{j}} = \frac{1}{k_N}\sum_{i=1}^{k_N}\tau_{N,i}^{\mathrm{j}} \tag{7}$$

$$range_{N,norm}^{\mathrm{j}} = \frac{\max_i log_{10}\left(\tau_{N,i}^{\mathrm{j}}\right) - \min_i log_{10}\left(\tau_{N,i}^{\mathrm{j}}\right)}{log_{10}\left(\tau_N^{\mathrm{j}}\right)} \tag{8}$$

and applied a series of selection criteria common to all RSFPs, which ensured recognition of bacteria by their size (pixel size is 127 nm, which gives 0.5 μm after 4×4 binning) and homogeneity among the $\tau_{N,i}^{\mathrm{j}}$ values.

For the extraction of the histograms of the characteristic times of RSFP-labeled bacteria, we adopted:

$$k_N > 3 \tag{9}$$

$$\log_{10}(\tau_N^{\mathrm{j}}) < 0 \quad \forall i \tag{10}$$

$$range_{N,norm}^{\mathrm{j}} < 0.3 \quad \forall i \tag{11}$$

In particular, this series of criteria led to discard background objects that were misidentified as bacteria because of their

size and bacteria, which were exhibiting a low signal-to-noise ratio (such as **2**-, **16**- and **21**-labeled bacteria) or dividing (resulting in highly dispersed values among their pixels and abnormal mean value of the characteristic times) because of the range criterion. Eventually, the characteristic times of the selected RSFP-labeled bacteria, τ_N^{j}, were used to plot their distribution and extract their mean, τ^{j}, and standard deviation, σ^{j} (at least 50 bacteria were analyzed to build these plots, which is sufficient to reliably retrieve the LIGHTNING kinetic fingerprint). Supplementary Figure 43–Supplementary Figure 46 display the extracted histograms of the characteristic times and Supplementary Table 13 sums up the results, which have been obtained for the investigated RSFP-labeled bacteria.

To build Figure 4c in the Main Text, we first selected randomly a single experiment for each protein type. We applied the segmentation code to each video and extracted the values of the characteristic time for each illumination $\mathrm{I_{low}}$, $\mathrm{I_{high}}$, and $\mathrm{II_{high}}$. Then we applied the following selection criteria on the characteristic times for each bacterium segmented:

$$k_N > 2 \tag{12}$$

$$\log_{10}(\tau_N^{\mathrm{j}}) < 0 \quad \forall i \tag{13}$$

$$range_{N,norm}^{\mathrm{j}} < 1 \quad \forall i \tag{14}$$

These criteria are less restrictive than the ones described for retrieving the histograms of the characteristic times of RSFP-labeled bacteria displayed in Supplementary Figure 43–Supplementary Figure 46. Hence we discard less bacteria and the distributions are wider on the images. The bacteria that were removed by the selection criteria do not appear in the final image.

To build the confusion matrix displayed in Figure 4d in the Main Text, we performed the following operations on the set of experiment samples that had allowed to build the histograms from Supplementary Figure 43–Supplementary Figure 46, focusing only on the 9 RSFPs of interest :

- Image segmentation with the soft threshold used in Eqs. (12–14). Since we used a less discriminatory threshold than to produce the histograms and the covariance data, we collected around 6000 bacteria (instead of 3600 bacteria);
- Computation of the probability P_{ij} defined in Eq. (20) for each of the 9 RSFPs;
- Assigning the class of highest probability to each bacterium;
- Comparison to the ground truth and production of the confusion matrix.

1.7 Definition of a set of distinguishable RSFPs

1.7.1 Sorting algorithms

Optimizing the discriminating power of LIGHTNING requires choosing the most distant RSFPs. We have implemented two algorithms to rank N RSFPs according to their distance between pairs. The distances d_{ij} between all pairs (i, j) of RSFPs

$$d_{ij} = \sqrt{\Sigma_{k=1}^{n} (l_{ki} - l_{kj})^2} \tag{15}$$

are computed in the space of n discriminative dimensions, where l_{ki} and l_{kj} are the decimal logarithms of the kth characteristic time of RSFP i and RSFP j, respectively.

Selection of the most distant RSFPs within sets of RSFPs We first determined optimized subsets $s'(m)$ of m RSFPs for $m = 2, ..., N$. The minimum distances $d_{\min}$ between pairs are computed for all subsets of m RSFPs included in $s(N)$. The subset $s'(m)$ with the maximum value of $d_{\min}$ is selected.

The Matlab script is provided below.

```
l1 % decimal logarithm of the characteristic times along dimension 1
l2 % decimal logarithm of the characteristic times along dimension 2
l3 % decimal logarithm of the characteristic times along dimension 3
l4 % decimal logarithm of the characteristic times along dimension 4

list = 1:1:N; % list of RSFPs to consider
N = length(list);

for k = 2:N
combs = combnk(list,k); % combinaisons from list with cardinal k
ncombs = length(combs(:,1));
dmin = 0;
for i = 1:ncombs % loop on the combinations
pairs = combnk(combs(i,:),2); % creation of the pairs
d = sqrt((l1(pairs(:,1)) - l1(pairs(:,2))).^2 + (l2(pairs(:,1)) - l2(pairs(:,2))).^2 + (l3(pairs(:,1)) - l3(pairs(:,2))).^2 + (l4(pairs(:,1)) - l4(pairs(:,2))).^2);
if max(isnan(d)) > 0
dmin(i) = NaN;
else dmin(i) = min(d); % minimum distance between pairs
end
end
[dminmax(k),id]=max(dmin); % maximum on combinations of minimum distances between pairs
listopt(k,1:k) = combs(id,:); % associated list
end

listopt % optimized subsets of RSFPs
dmin % associated minimum distance
```

Selection of the most distant RSFPs within inclusive subsets of an RSFP set As shown in Supplementary Table 8 and Supplementary Table 15, the relation $s'(m) \subset s'(m+1)$ is not necessarily observed for $s'(m)$ and $s'(m+1)$. This absence may be a drawback for end-users who would like to use a same set of RSFPs in order to discriminate various numbers of its members. Hence we have also implemented the following efficient algorithm including three-body interactions to rank N RSFPs according to their distance between pairs.

The procedure followed to generate an ordered list of RSFPs begins with sorting pairs by increasing distances and sequentially eliminating the RSFP which is the closest to two other RSFPs. Specifically, the pair associated with the smallest distance in the set $s(N)$ of N RSFPs is denoted (i, j). The closest RSFP to i different from j is denoted k

and the closest RSFP to j different from i is denoted l. If the distance d_{ik} is smaller than d_{jl}, the RSFP i is the closest RSFP to two RSFPs. The rank assigned to the RSFP i in the ordered list is equal to the number N of RSFPs in the set $s(N)$. Then the RSFP i is removed from the set $s(N)$ leading to the set $s(N-1)$ of $N-1$ RSFPs. The procedure is repeated until the set $s(2)$ has been built. Rankings 1 and 2 are assigned to the two RSFPs of the remaining pair $s(2)$.

The Matlab script is provided below.

```
l1 % decimal logarithm of the characteristic times along dimension 1
l2 % decimal logarithm of the characteristic times along dimension 2
l3 % decimal logarithm of the characteristic times along dimension 3
l4 % decimal logarithm of the characteristic times along dimension 4

list = 1:1:N; % list of RSFPs to consider
N = length(list);

pairs = combnk(list,2); % creation of the pairs
d = sqrt((l1(pairs(:,1)) - l1(pairs(:,2))).^2 + (l2(pairs(:,1)) - l2(pairs(:,2))).^2 + (l3(pairs(:,1)) - l3(pairs(:,2))).^2 + (l4(pairs(:,1)) - l4(pairs(:,2))).^2);
[d,idx] = sort(d); % sorting of distances
sortedpairsa = pairs(idx,1); % sorting of the first pair numbers
sortedpairsb = pairs(idx,2); % sorting of the second pair numbers

for k = N:-1:3 % loop on the sizes
dmin(k) = d(1); % dmin
a = sortedpairsa(1); % RSFPs of the pair associated with dmin
b = sortedpairsb(1);
idaa = find(a == sortedpairsa); % position of RSFPs equal to a
idab = find(a == sortedpairsb);
idbb = find(b == sortedpairsb); % position of RSFPs equal to b
idba = find(b == sortedpairsa);
if length(idaa) < 2 % 2nd position of RSFPs equal to a
ida = idab(1);
elseif length(idab) < 1
ida = idaa(2);
else
ida = min(idaa(2),idab(1));
end
if length(idbb) < 2 % 2nd position of RSFPs equal to b
idb = idba(1);
elseif length(idba) < 1
idb = idbb(2);
else
```

idb = min(idbb(2),idba(1));
end

if ida < idb % which of a or b has the strongest three-body interaction
sortedlist(k) = a;
else
sortedlist(k) = b;
end

idx = vertcat(find(sortedpairsa == sortedlist(k)),find(sortedpairsb == sortedlist(k))); % position of the RSFP to be removed
sortedpairsa(idx) = []; % RSFP removed from the pairs
sortedpairsb(idx) = [];
d(idx) = []; % RSF removed from the distance
end
dmin(2) = d; % minimum distance of the remaining pair
sortedlist(1) = sortedpairsa; % RSFPs of the remaining pair
sortedlist(2) = sortedpairsb;

sortedlist % list of sorted RSFPs from the closest to the most remote
dmin % associated minimum distance

1.7.2 Evaluation of the cutoff distance

Discrimination between two RSFPs is possible if their distance is larger than the cutoff distance d_c imposed by the experimental accuracy Δd_{ij} on the distance d_{ij} induced by the uncertainty Δl_{ki} on the logarithm of the characteristic times l_{ki}. Differentiating Eq.(15), we find

$$\Delta d_{ij} = \frac{1}{d_{ij}} \Sigma_{k=1}^{n} \mid l_{ki} - l_{kj} \mid \Delta \mid l_{ki} - l_{kj} \mid . \tag{16}$$

Introducing the maximum uncertainty $M = \max_{k,i} (\Delta l_{ki})$ on the determination of the kinetic fingerprint $\{l_{ki}\}$, we have $\Delta \mid l_{ki} - l_{kj} \mid \leq 2M$. Hence Eq.(16) reads

$$\Delta d_{ij} \leq 2MX \tag{17}$$

with $X^2 = 1 + 2\Sigma_{k=1}^{n} \Sigma_{k'=k+1}^{n} \mid l_{ki} - l_{kj} \mid\mid l_{k'i} - l_{k'j} \mid / d_{ij}^2$

Regardless of the value of $\mid l_{ki} - l_{kj} \mid$, the inequality

$$2\Sigma_{k=1}^{n} \Sigma_{k'=k+1}^{n} \mid l_{ki} - l_{kj} \mid\mid l_{k'i} - l_{k'j} \mid \leq (n-1) \Sigma_{k=1}^{n} (l_{ki} - l_{kj})^2 \tag{18}$$

is observed, leading to $X \leq \sqrt{n}$. Using Eq.(17) we find $\Delta d_{ij} \leq d_c$ where the cutoff distance obeys

$$d_c = 2M\sqrt{n} \tag{19}$$

The cutoff distance between two RSFPs depends on two parameters, the number n of discriminating dimensions and the uncertainty M on the determination of the logarithm of the characteristic times. The protocol used to evaluate M is described in sections (D.1.3) and (D.2.2).

1.8 Assigning an identity to an unknown RSFP

We consider a mixture of N noncolocalized RSFPs. The kinetic fingerprint $(l_{1j}, l_{2j}, ..., l_{nj})$ of each RSFP j in a space of n characteristic times is supposed to be known. The kinetic fingerprint $(l_{1i}, l_{2i}, ..., l_{ni})$ of a given pixel occupied by an unknown RSFP i can be used to assign an identity to i.

For sufficiently close uncertainties on the determination of the different characteristic times, the distance d_{ij} given in Eq. (15) allows us to conclude. If the distance d_{ij} between the kinetic fingerprints $(l_{1i}, l_{2i}, ..., l_{ni})$ and $(l_{1j}, l_{2j}, ..., l_{nj})$ is smaller than the cutoff distance d_c, the unknown RSFP i is considered to be the RSFP j.

A more refined method is necessary in the case of different uncertainties on the characteristic times. For each RSFP j, we introduce the vector $\Lambda_j = (l_{1j}, l_{2j}, ..., l_{nj})$ considered as a random variable of mean value $\langle\Lambda_j\rangle$. The kinetic fingerprint $\Lambda_i = (l_{1i}, l_{2i}, ..., l_{ni})$ of an unknown RSFP i is compared to Λ_j using the probability

$$P_{ij} = \exp\left(-\frac{1}{2}\left(\Lambda_i - \langle\Lambda_j\rangle\right)\left(\Sigma^j\right)^{-1}\left(\Lambda_i - \langle\Lambda_j\rangle\right)^T\right) \tag{20}$$

where Σ^j is the covariance matrix associated with RSFP j of elements

$$\Sigma^j_{kk'} = \langle\left(l_{kj} - \langle l_{kj}\rangle\right)\left(l_{k'j} - \langle l_{k'j}\rangle\right)\rangle \tag{21}$$

with $k, k' = 1, ..., n$. The value of P_{ij} tends to 1 when the two kinetic fingerprints Λ_i and Λ_j are close. The two RSFPs i and j have then a high probability to be identical.

2 Supplementary Figures

2.1 Absorption and fluorescence emission spectra of the RSFPs

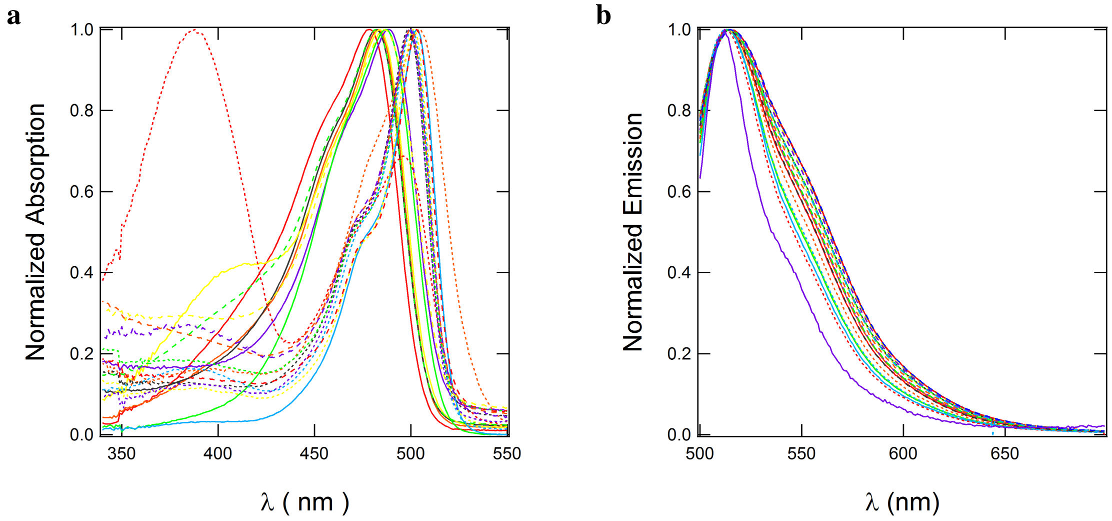

Supplementary Figure 13: Normalized absorption (**a**) and emission (**b**; λ_{exc} = 488 nm) spectra of the 22 RSFPs, which have been investigated in this study. See also Fig. 3**a**,**b** in the Main Text. RSFP solutions: 5 μM in pH 7.4 PBS (50 mM sodium phosphate, 150 mM NaCl); T = 298 K.

2.2 Measurement of the rate constant k_{21}^{Δ} associated with the thermally driven relaxation of the photoswitched RSFPs

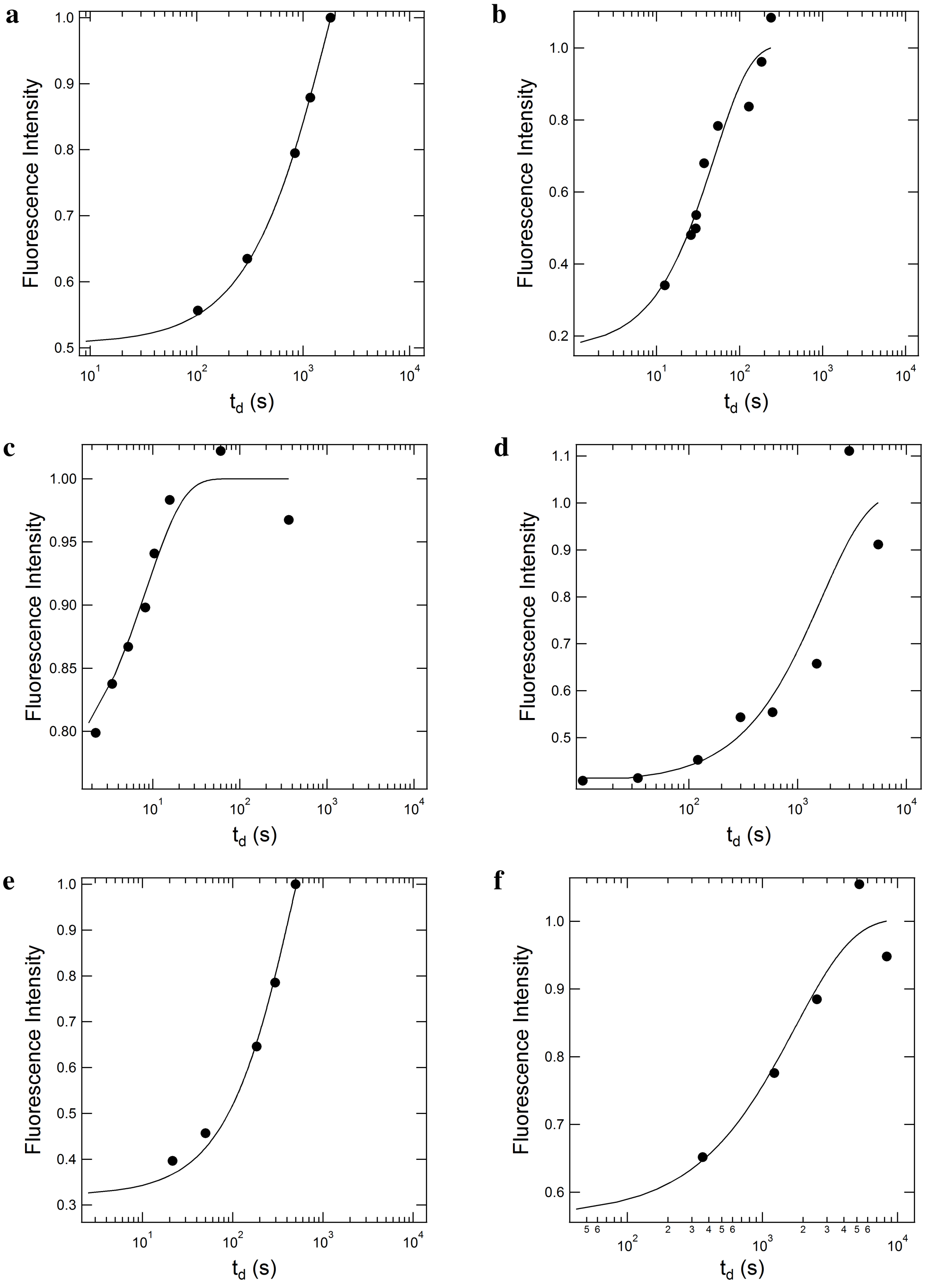

Supplementary Figure 14: Dependence of the normalized fluorescence emission at the initial time of each light pulse on the delay t_d between two pulses at 480 nm (in a regime of low light intensity in which a two-state model is relevant to account for the photoswitching behavior). **a**: **1**; **b**: **2**; **c**: **3**; **d**: **6**; **e**: **7**; **f**: **8**. RSFP solutions: 8 μM in pH 7.4 PBS (50 mM sodium phosphate, 150 mM NaCl); T = 298 K.

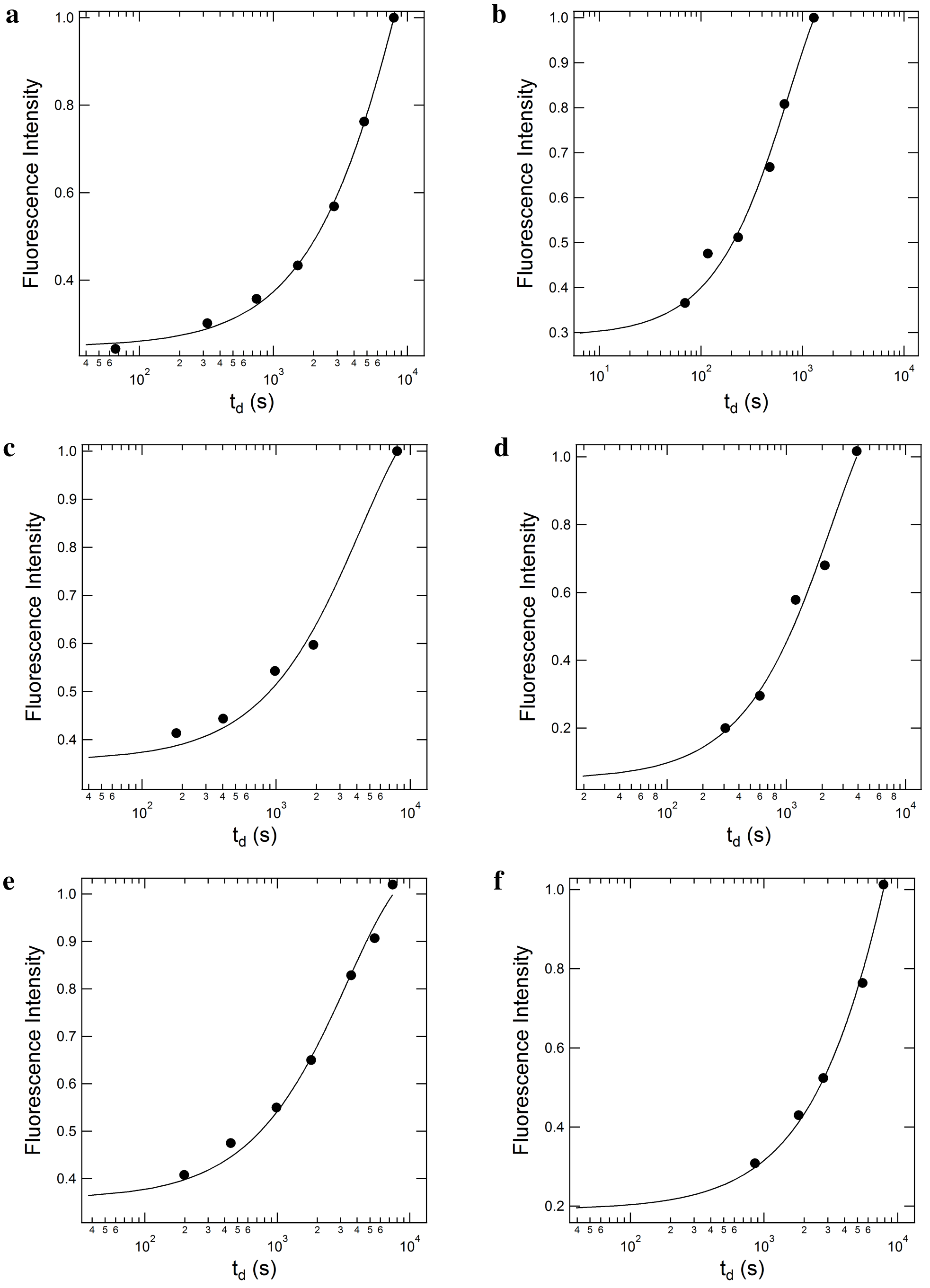

Supplementary Figure 15: Dependence of the normalized fluorescence emission at the initial time of each light pulse on the delay t_d between two pulses at 480 nm (in a regime of low light intensity in which a two-state model is relevant to account for the photoswitching behavior). **a**: **9**; **b**: **10**; **c**: **11**; **d**: **12**; **e**: **13**; **f**: **14**. RSFP solutions: 8 μM in pH 7.4 PBS (50 mM sodium phosphate, 150 mM NaCl); T = 298 K.

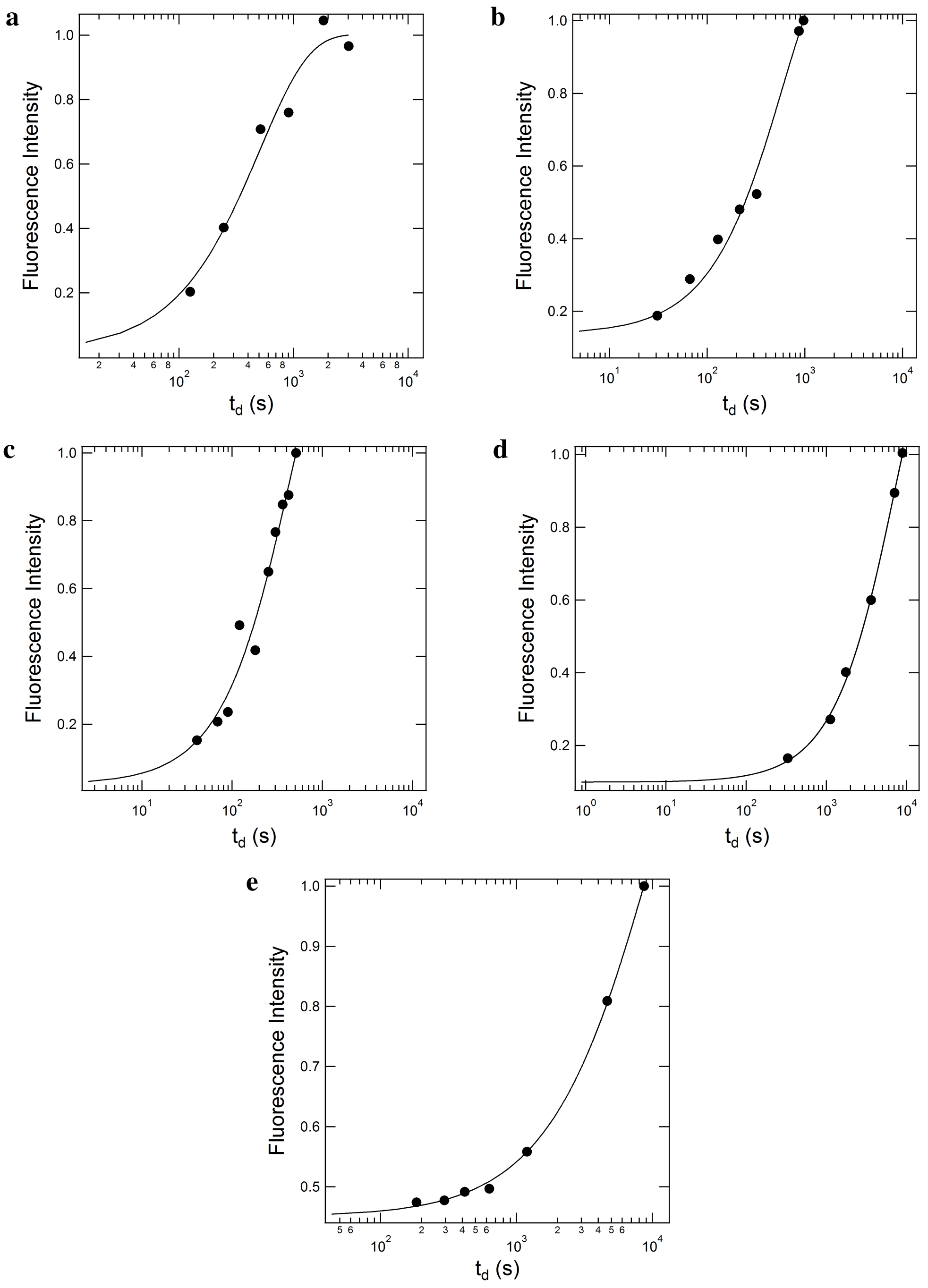

Supplementary Figure 16: Dependence of the normalized fluorescence emission at the initial time of each light pulse on the delay t_d between two pulses at 480 nm (in a regime of low light intensity in which a two-state model is relevant to account for the photoswitching behavior). **a**: **15**; **b**: **16**; **c**: **17**; **d**: **18**; **e**: **21**. RSFP solutions: 8 μM in pH 7.4 PBS (50 mM sodium phosphate, 150 mM NaCl); T = 298 K.

2.3 Photochemical characterization of the RSFPs

2.3.1 Preliminary illumination experiments on the RSFPs

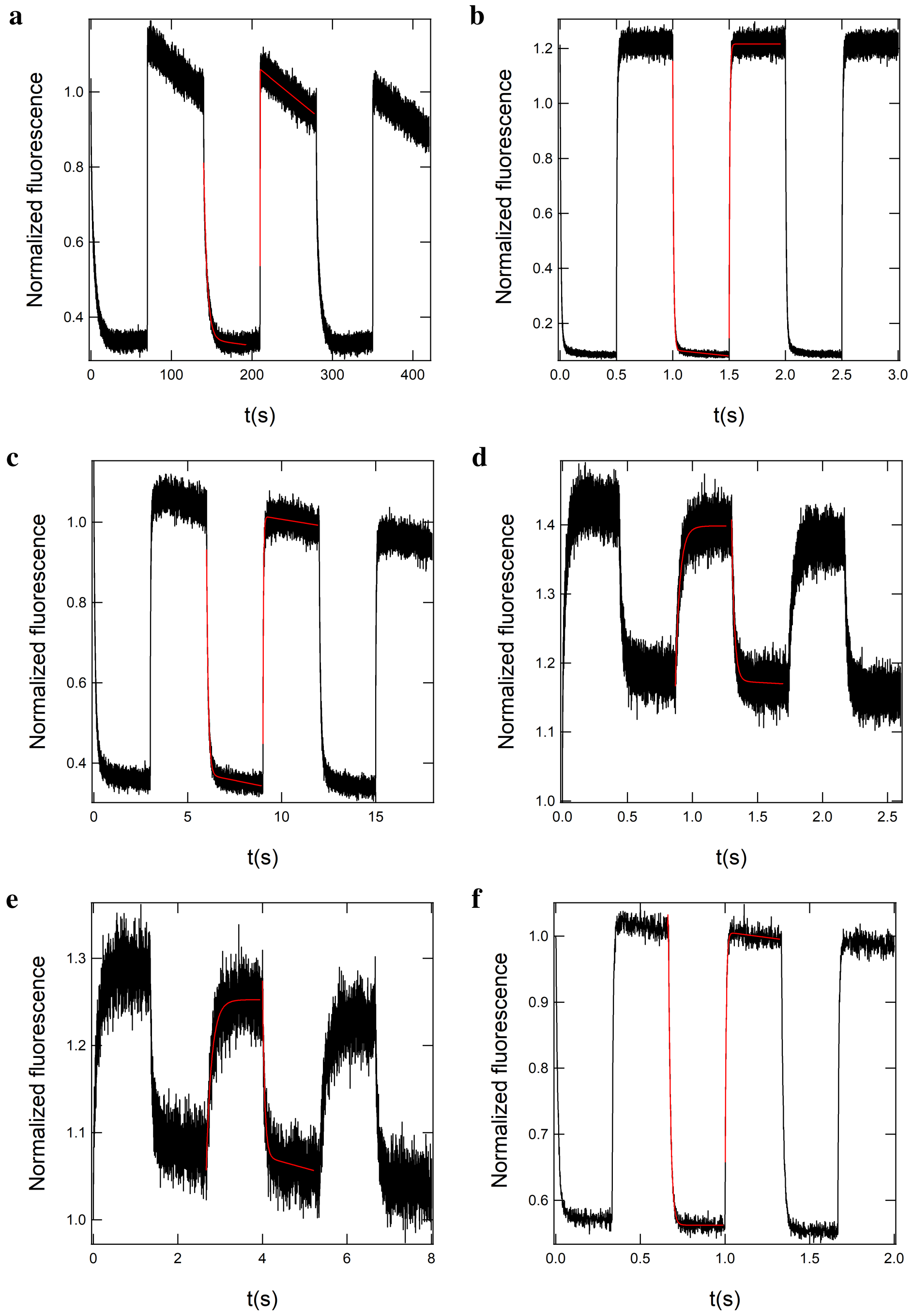

Supplementary Figure 17: Preliminary analysis of fluorescence photoswitching for **1** (**a**), **2** (**b**), **3** (**c**), **4** (**d**), **5** (**e**), **6** (**f**). Evolution of the fluorescence intensity scaled by the initial value upon continuous illumination of intensity I_1 at $\lambda_1 = 488$ nm and square-wave illumination of intensity I_2 at $\lambda_2 = 405$ nm yielding two different illumination regimes I ($I_1 = 0.6$ ein.m^{-2}.s^{-1}) and II ($I_1 = 0.6$ ein.m^{-2}.s^{-1} and $I_2 = 0.1$ ein.m^{-2}.s^{-1}). Solid black line: Experimental data; solid red line: Biexponentional fitting function with Eq.(5). RSFP solutions: 20 μM in pH 7.4 PBS (50 mM sodium phosphate, 150 mM NaCl); T = 298 K.

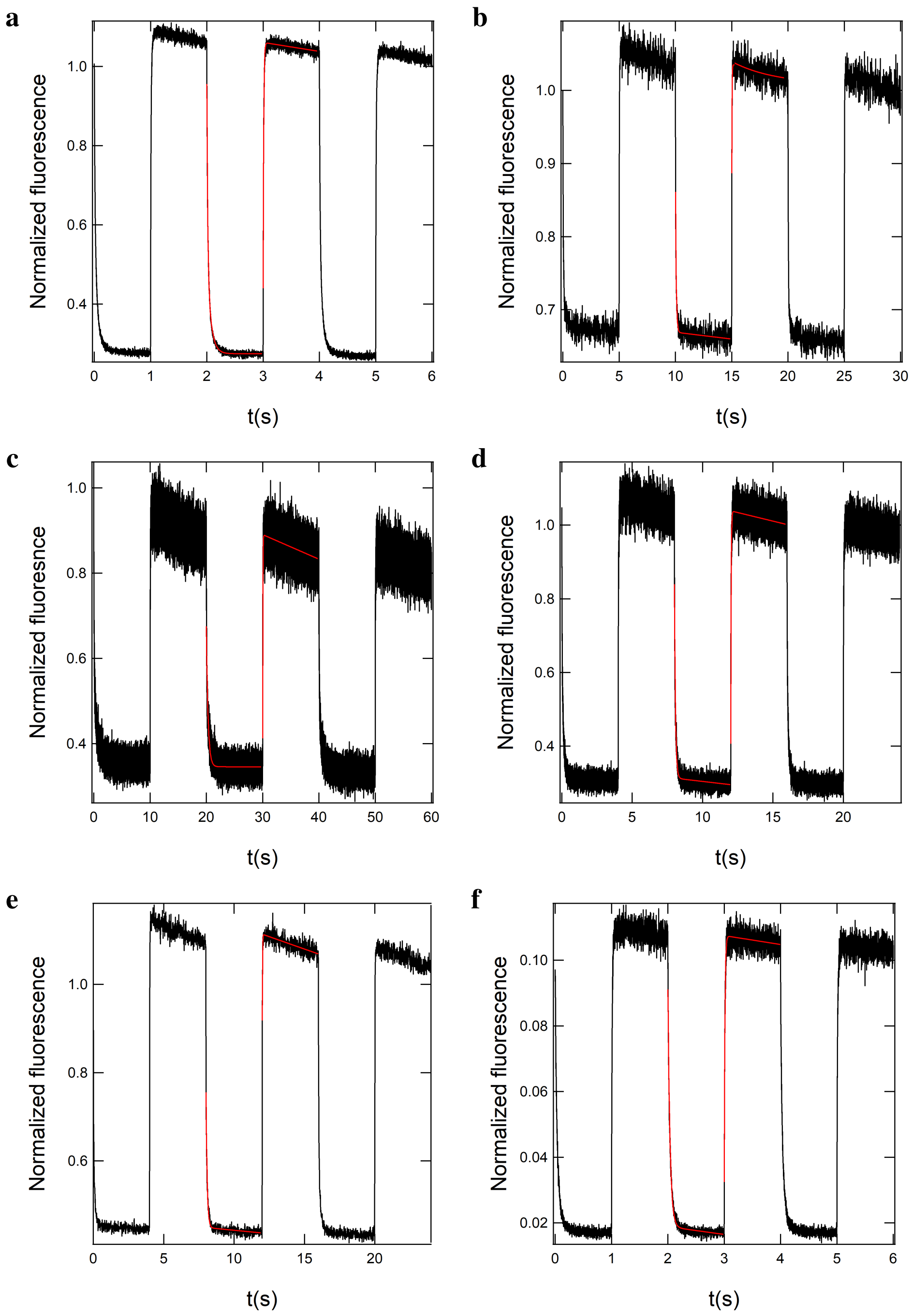

Supplementary Figure 18: Preliminary analysis of fluorescence photoswitching for **7** (**a**), **8** (**b**), **9** (**c**), **10** (**d**), **11** (**e**), **12** (**f**). Evolution of the fluorescence intensity scaled by the initial value upon continuous illumination of intensity I_1 at $\lambda_1 = 488$ nm and square-wave illumination of intensity I_2 at $\lambda_2 = 405$ nm yielding two different illumination regimes I ($I_1 = 0.6$ ein.m^{-2}.s^{-1} – except for **8** where it is 0.4 ein.m^{-2}.s^{-1}) and II ($I_1 = 0.6$ ein.m^{-2}.s^{-1} – except for **8** where it is 0.4 ein.m^{-2}.s^{-1} – and $I_2 = 0.1$ ein.m^{-2}.s^{-1}). Solid black line: Experimental data; solid red line: Biexponentional fitting function with Eq.(5). RSFP solutions: 20 μM in pH 7.4 PBS (50 mM sodium phosphate, 150 mM NaCl); T = 298 K.

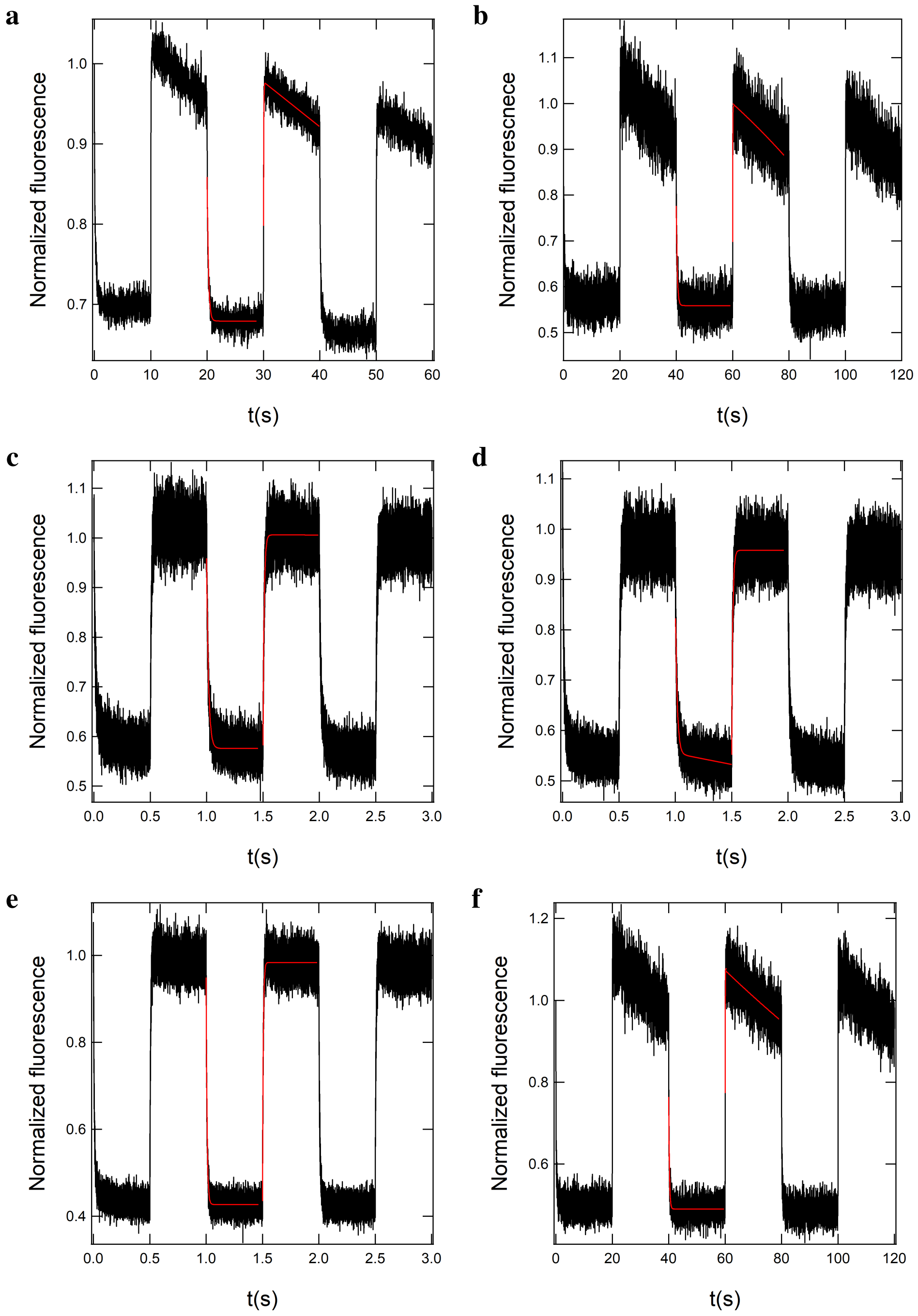

Supplementary Figure 19: Preliminary analysis of fluorescence photoswitching for **13** (**a**), **14** (**b**), **15** (**c**), **16** (**d**), **17** (**e**), **18** (**f**). Evolution of the fluorescence intensity scaled by the initial value upon continuous illumination of intensity I_1 at $\lambda_1 = 488$ nm and square-wave illumination of intensity I_2 at $\lambda_2 = 405$ nm yielding two different illumination regimes I ($I_1 = 0.6$ ein.m^{-2}.s^{-1} – except for **13** where it is 0.5 ein.m^{-2}.s^{-1}) and II ($I_1 = 0.6$ ein.m^{-2}.s^{-1} – except for **13** where it is 0.5 ein.m^{-2}.s^{-1} – and $I_2 = 0.1$ ein.m^{-2}.s^{-1}). Solid black line: Experimental data; solid red line: Biexponentional fitting function with Eq.(5). RSFP solutions: 20 μM in pH 7.4 PBS (50 mM sodium phosphate, 150 mM NaCl); T = 298 K.

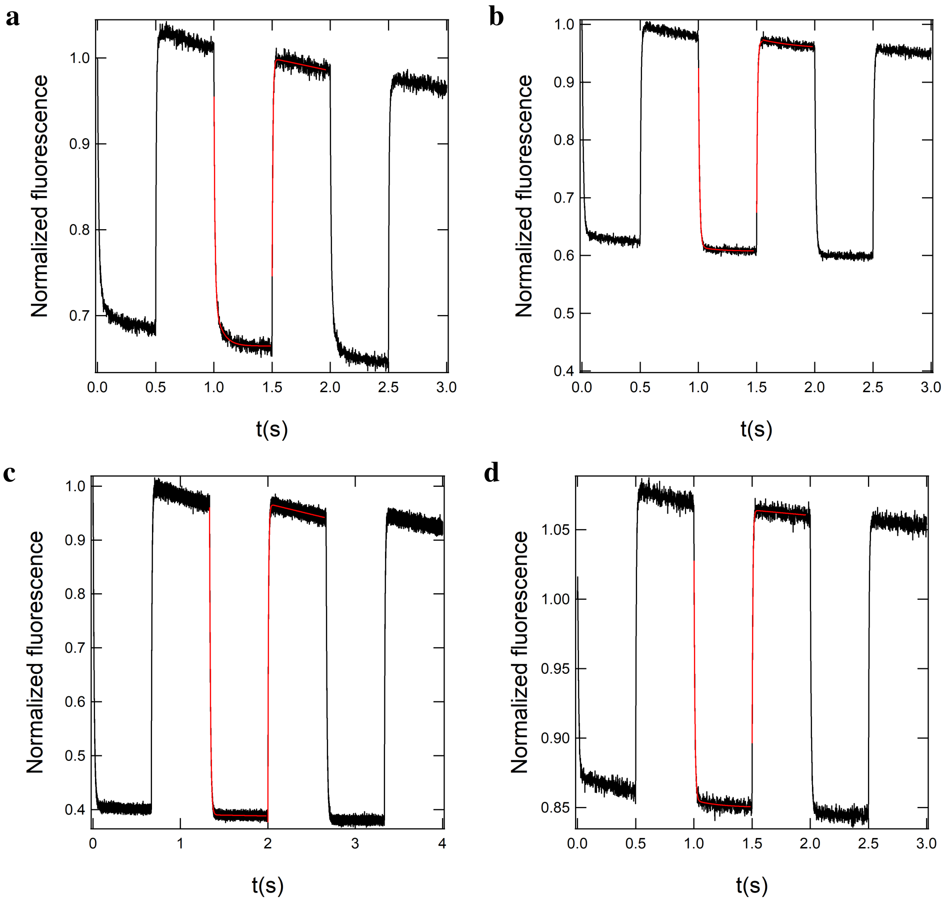

Supplementary Figure 20: Preliminary analysis of fluorescence photoswitching for **19** (**a**), **20** (**b**), **21** (**c**), **22** (**d**). Evolution of the fluorescence intensity scaled by the initial value upon continuous illumination of intensity I_1 at $\lambda_1 = 488$ nm and square-wave illumination of intensity I_2 at $\lambda_2 = 405$ nm yielding two different illumination regimes I ($I_1 = 0.6$ ein.m^{-2}.s^{-1}) and II ($I_1 = 0.6$ ein.m^{-2}.s^{-1} and $I_2 = 0.1$ ein.m^{-2}.s^{-1} – except for **20** where it is 0.08 ein.m^{-2}.s^{-1}). Solid black line: Experimental data; solid red line: Biexponentional fitting function with Eq.(5). RSFP solutions: 20 μM in pH 7.4 PBS (50 mM sodium phosphate, 150 mM NaCl); T = 298 K.

2.3.2 Final illumination experiments

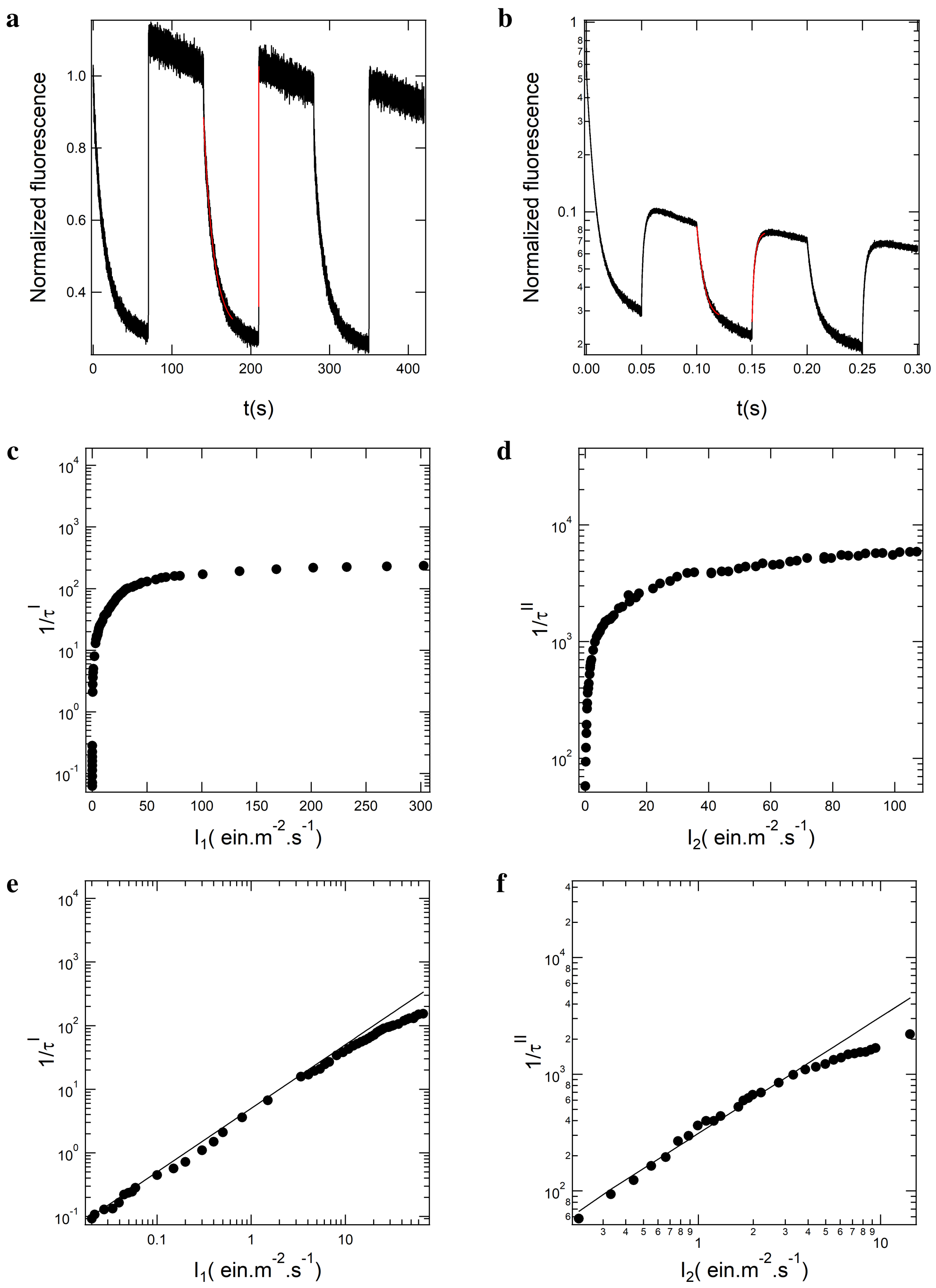

Supplementary Figure 21: Kinetic analysis of **1** photoswitching at 20 μM in pH 7.4 PBS (50 mM sodium phosphate, 150 mM NaCl); T = 298 K. **a**, **b**: Evolution of the fluorescence intensity scaled by the initial value upon continuous illumination of intensity I_1 at $\lambda_1 = 488$ nm and square-wave illumination of intensity I_2 at $\lambda_2 = 405$ nm (**a**: Illumination I $I_1 = 0.05$ ein.m^{-2}.s^{-1}, $I_2 = 0.1$ ein.m^{-2}.s^{-1}; **b**: $I_1 = 200$ ein.m^{-2}.s^{-1}, $I_2 = 3$ ein.m^{-2}.s^{-1}). Black line: experimental data; red line: fitting functions according to Eq.(40) and Eq.(49); **c**: Illumination I: Inverse of the relaxation time $1/\tau^{\mathrm{I}}$ (disks) versus light intensity I_1 at $\lambda_1 = 488$ nm, **d**: Illumination II: Inverse of the relaxation time $1/\tau^{\mathrm{II}}$ (disks) versus light intensity I_2 at $\lambda_2 = 405$ nm with $I_1 = 0.2$ ein.m^{-2}.s^{-1}; **e**: Magnification of **c** at low light intensity. Solid line: linear fitting function yielding the photoswitching cross section Σ_1, **f**: Magnification of **d** at low light intensity. Solid line: linear fitting function yielding the photoswitching cross section Σ_2.

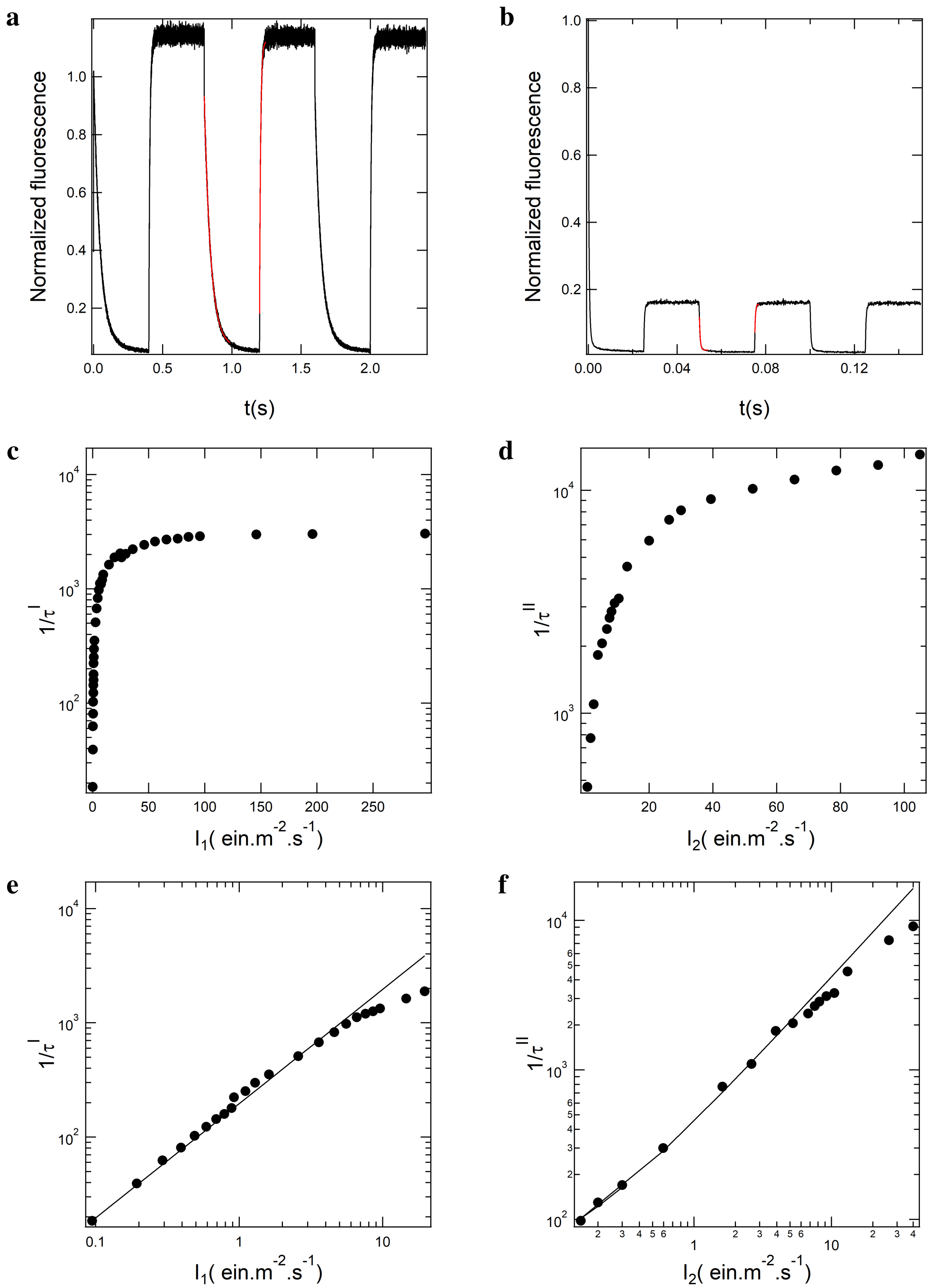

Supplementary Figure 22: Kinetic analysis of **2** photoswitching at 20 μM in pH 7.4 PBS (50 mM sodium phosphate, 150 mM NaCl); T = 298 K. **a**, **b**: Evolution of the fluorescence intensity scaled by the initial value upon continuous illumination of intensity I_1 at $\lambda_1 = 488$ nm and square-wave illumination of intensity I_2 at $\lambda_2 = 405$ nm (**a**: $I_1 = 0.05$ ein.m^{-2}.s^{-1}, $I_2 = 0.1$ ein.m^{-2}.s^{-1}; **b**: $I_1 = 200$ ein.m^{-2}.s^{-1}, $I_2 = 3$ ein.m^{-2}.s^{-1}). Black line: experimental data; red line: fitting functions according to Eq.(40) and Eq.(49); **c**: Illumination I: Inverse of the relaxation time $1/\tau^{\mathrm{I}}$ (disks) versus light intensity I_1 at $\lambda_1 = 488$ nm, **d**: Illumination II: Inverse of the relaxation time $1/\tau^{\mathrm{II}}$ (disks) versus light intensity I_2 at $\lambda_2 = 405$ nm with $I_1 = 0.2$ ein.m^{-2}.s^{-1}; **e**: Magnification of **c** at low light intensity. Solid line: linear fitting function yielding the photoswitching cross section Σ_1, **f**: Magnification of **d** at low light intensity. Solid line: linear fitting function yielding the photoswitching cross section Σ_2.

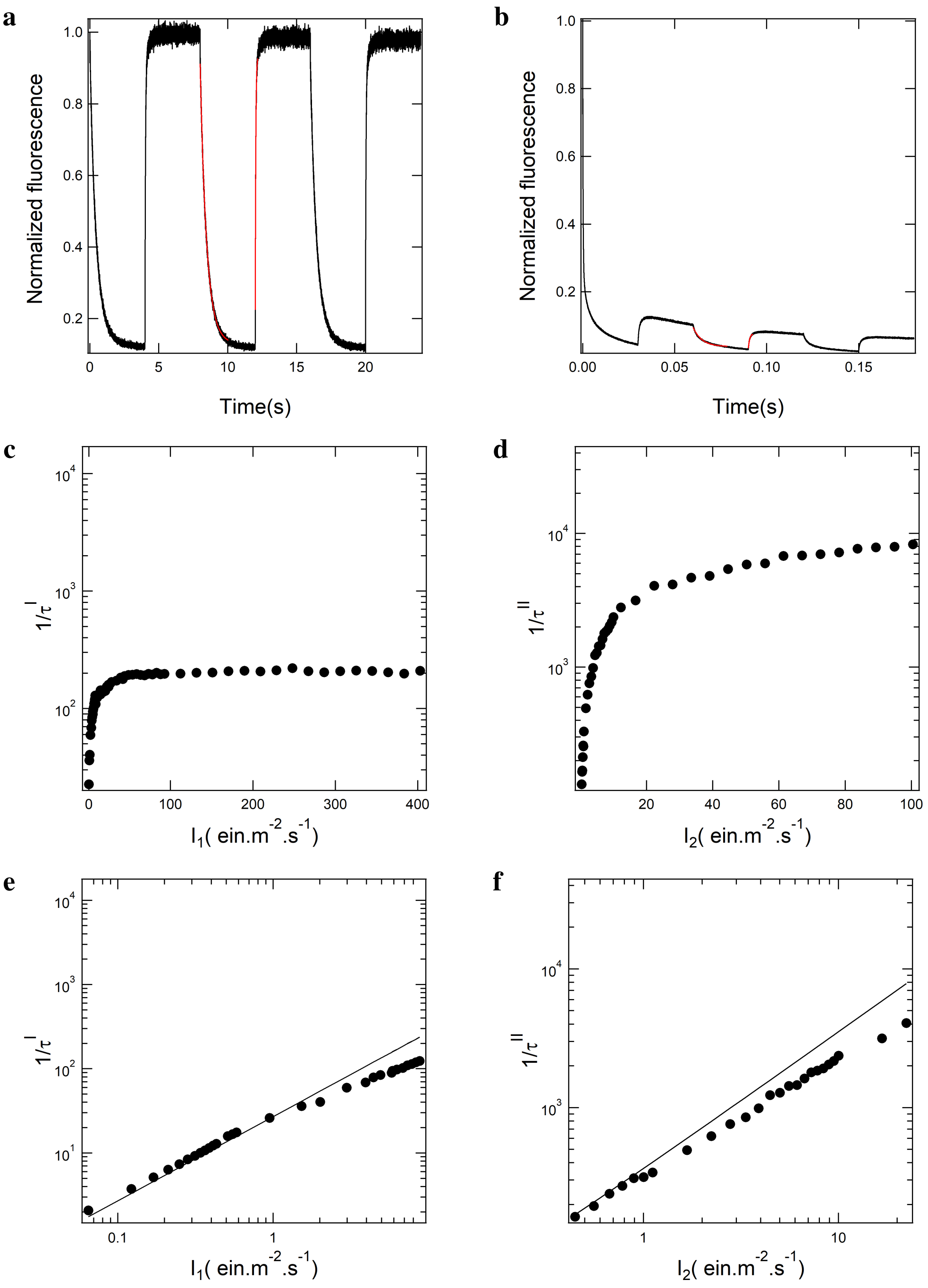

Supplementary Figure 23: Kinetic analysis of **3** photoswitching at 20 μM in pH 7.4 PBS (50 mM sodium phosphate, 150 mM NaCl); T = 298 K. **a**, **b**: Evolution of the fluorescence intensity scaled by the initial value upon continuous illumination of intensity I_1 at $\lambda_1 = 488$ nm and square-wave illumination of intensity I_2 at $\lambda_2 = 405$ nm (**a**: $I_1 = 0.05$ ein.m^{-2}.s^{-1}, $I_2 = 0.1$ ein.m^{-2}.s^{-1}; **b**: $I_1 = 200$ ein.m^{-2}.s^{-1}, $I_2 = 3$ ein.m^{-2}.s^{-1}). Black line: experimental data; red line: fitting functions according to Eq.(40) and Eq.(49); **c**: Illumination I: Inverse of the relaxation time $1/\tau^{\mathrm{I}}$ (disks) versus light intensity I_1 at $\lambda_1 = 488$ nm, **d**: Illumination II: Inverse of the relaxation time $1/\tau^{\mathrm{II}}$ (disks) versus light intensity I_2 at $\lambda_2 = 405$ nm with $I_1 = 0.2$ ein.m^{-2}.s^{-1}; **e**: Magnification of **c** at low light intensity. Solid line: linear fitting function yielding the photoswitching cross section Σ_1, **f**: Magnification of **d** at low light intensity. Solid line: linear fitting function yielding the photoswitching cross section Σ_2.

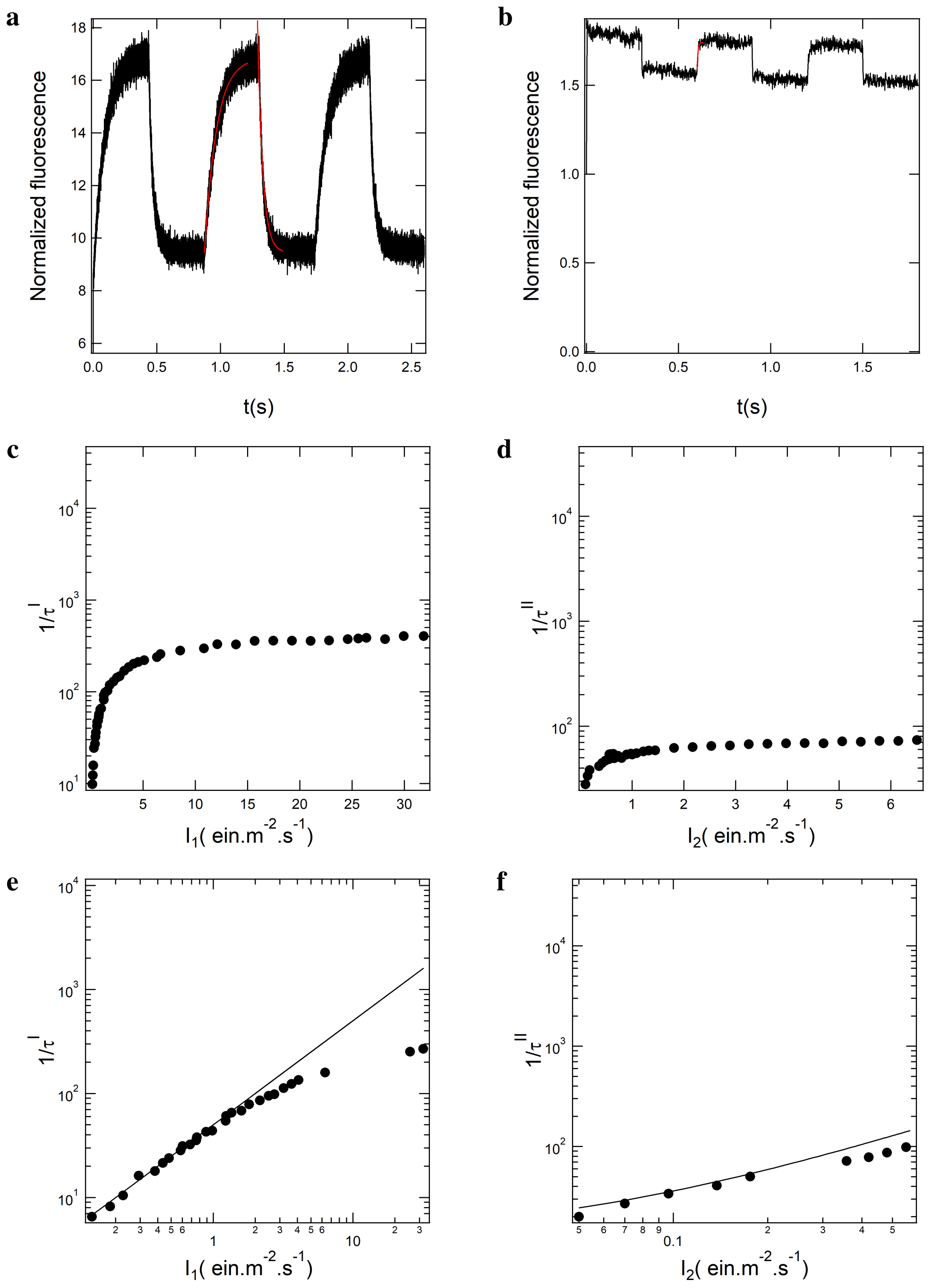

Supplementary Figure 24: Kinetic analysis of **4** photoswitching at 20 μM in pH 7.4 PBS (50 mM sodium phosphate, 150 mM NaCl); T = 298 K. **a**, **b**: Evolution of the fluorescence intensity scaled by the initial value upon continuous illumination of intensity I_1 at $\lambda_1 = 488$ nm and square-wave illumination of intensity I_2 at $\lambda_2 = 405$ nm (**a**: $I_1 = 0.05$ ein.m^{-2}.s^{-1}, $I_2 = 0.1$ ein.m^{-2}.s^{-1}; **b**: $I_1 = 200$ ein.m^{-2}.s^{-1}, $I_2 = 3$ ein.m^{-2}.s^{-1}). Black line: experimental data; red line: fitting functions according to Eq.(40) and Eq.(49); **c**: Illumination I: Inverse of the relaxation time $1/\tau^{\mathrm{I}}$ (disks) versus light intensity I_1 at $\lambda_1 = 488$ nm, **d**: Illumination II: Inverse of the relaxation time $1/\tau^{\mathrm{II}}$ (disks) versus light intensity I_2 at $\lambda_2 = 405$ nm with $I_1 = 0.2$ ein.m^{-2}.s^{-1}; **e**: Magnification of **c** at low light intensity. Solid line: linear fitting function yielding the photoswitching cross section Σ_1, **f**: Magnification of **d** at low light intensity. Solid line: linear fitting function yielding the photoswitching cross section Σ_2.

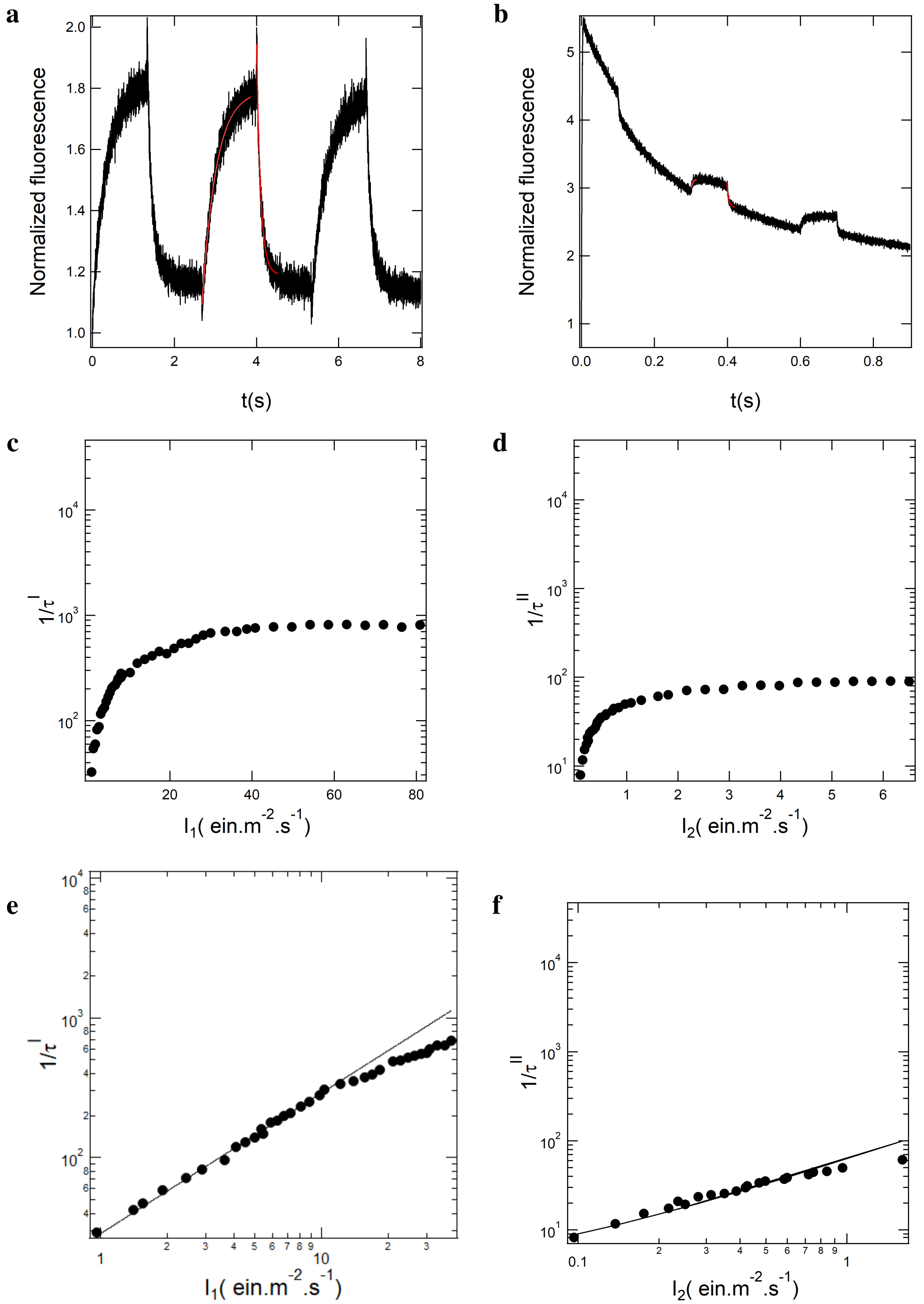

Supplementary Figure 25: Kinetic analysis of **5** photoswitching at 20 μM in pH 7.4 PBS (50 mM sodium phosphate, 150 mM NaCl); T = 298 K. **a**, **b**: Evolution of the fluorescence intensity scaled by the initial value upon continuous illumination of intensity I_1 at $\lambda_1 = 488$ nm and square-wave illumination of intensity I_2 at $\lambda_2 = 405$ nm (**a**: $I_1 = 0.05$ ein.m^{-2}.s^{-1}, $I_2 = 0.1$ ein.m^{-2}.s^{-1}; **b**: $I_1 = 200$ ein.m^{-2}.s^{-1}, $I_2 = 3$ ein.m^{-2}.s^{-1}). Black line: experimental data; red line: fitting functions according to Eq.(40) and Eq.(49); **c**: Illumination I: Inverse of the relaxation time $1/\tau^{\mathrm{I}}$ (disks) versus light intensity I_1 at $\lambda_1 = 488$ nm, **d**: Illumination II: Inverse of the relaxation time $1/\tau^{\mathrm{II}}$ (disks) versus light intensity I_2 at $\lambda_2 = 405$ nm with $I_1 = 0.2$ ein.m^{-2}.s^{-1}; **e**: Magnification of **c** at low light intensity. Solid line: linear fitting function yielding the photoswitching cross section Σ_1, **f**: Magnification of **d** at low light intensity. Solid line: linear fitting function yielding the photoswitching cross section Σ_2.

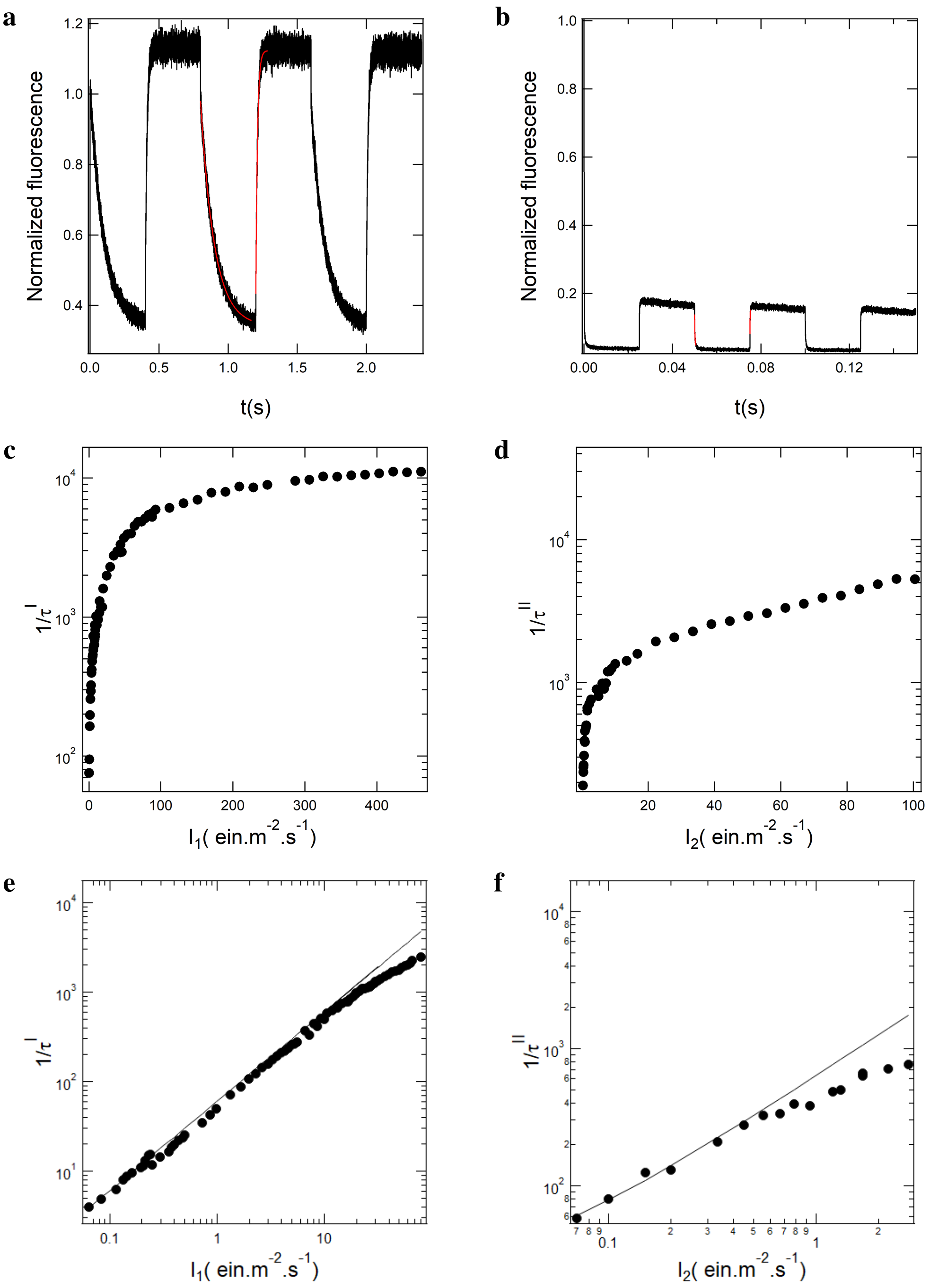

Supplementary Figure 26: Kinetic analysis of **6** photoswitching at 20 μM in pH 7.4 PBS (50 mM sodium phosphate, 150 mM NaCl); T = 298 K. **a**, **b**: Evolution of the fluorescence intensity scaled by the initial value upon continuous illumination of intensity I_1 at $\lambda_1 = 488$ nm and square-wave illumination of intensity I_2 at $\lambda_2 = 405$ nm (**a**: $I_1 = 0.05$ ein.m^{-2}.s^{-1}, $I_2 = 0.1$ ein.m^{-2}.s^{-1}; **b**: $I_1 = 200$ ein.m^{-2}.s^{-1}, $I_2 = 3$ ein.m^{-2}.s^{-1}). Black line: experimental data; red line: fitting functions according to Eq.(40) and Eq.(49); **c**: Illumination I: Inverse of the relaxation time $1/\tau^{\mathrm{I}}$ (disks) versus light intensity I_1 at $\lambda_1 = 488$ nm, **d**: Illumination II: Inverse of the relaxation time $1/\tau^{\mathrm{II}}$ (disks) versus light intensity I_2 at $\lambda_2 = 405$ nm with $I_1 = 0.2$ ein.m^{-2}.s^{-1}; **e**: Magnification of **c** at low light intensity. Solid line: linear fitting function yielding the photoswitching cross section Σ_1, **f**: Magnification of **d** at low light intensity. Solid line: linear fitting function yielding the photoswitching cross section Σ_2.

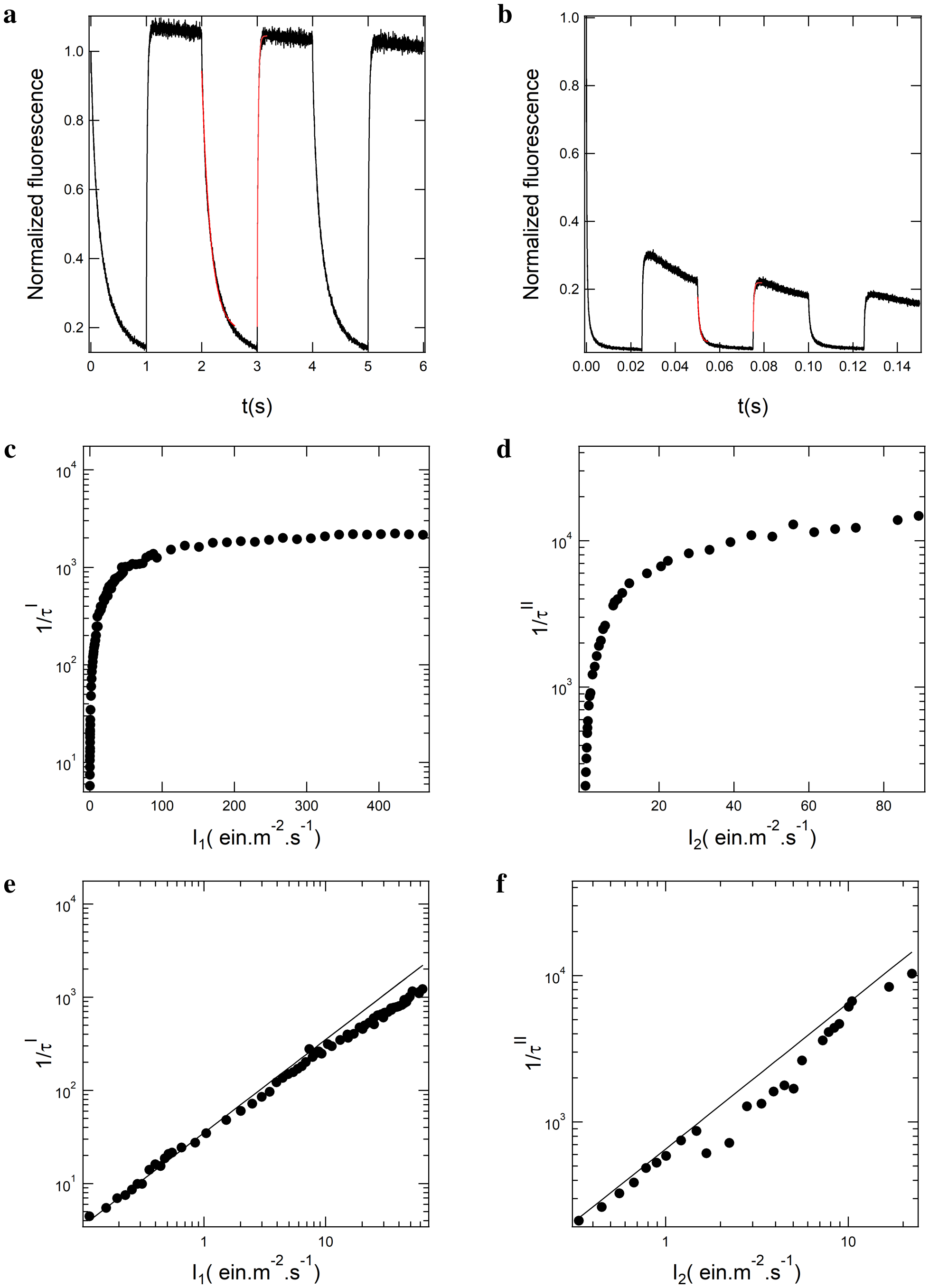

Supplementary Figure 27: Kinetic analysis of **7** photoswitching at 20 μM in pH 7.4 PBS (50 mM sodium phosphate, 150 mM NaCl); T = 298 K. **a**, **b**: Evolution of the fluorescence intensity scaled by the initial value upon continuous illumination of intensity I_1 at $\lambda_1 = 488$ nm and square-wave illumination of intensity I_2 at $\lambda_2 = 405$ nm (**a**: $I_1 = 0.05$ ein.m^{-2}.s^{-1}, $I_2 = 0.1$ ein.m^{-2}.s^{-1}; **b**: $I_1 = 200$ ein.m^{-2}.s^{-1}, $I_2 = 3$ ein.m^{-2}.s^{-1}). Black line: experimental data; red line: fitting functions according to Eq.(40) and Eq.(49); **c**: Illumination I: Inverse of the relaxation time $1/\tau^{\mathrm{I}}$ (disks) versus light intensity I_1 at $\lambda_1 = 488$ nm, **d**: Illumination II: Inverse of the relaxation time $1/\tau^{\mathrm{II}}$ (disks) versus light intensity I_2 at $\lambda_2 = 405$ nm with $I_1 = 0.2$ ein.m^{-2}.s^{-1}; **e**: Magnification of **c** at low light intensity. Solid line: linear fitting function yielding the photoswitching cross section Σ_1, **f**: Magnification of **d** at low light intensity. Solid line: linear fitting function yielding the photoswitching cross section Σ_2.

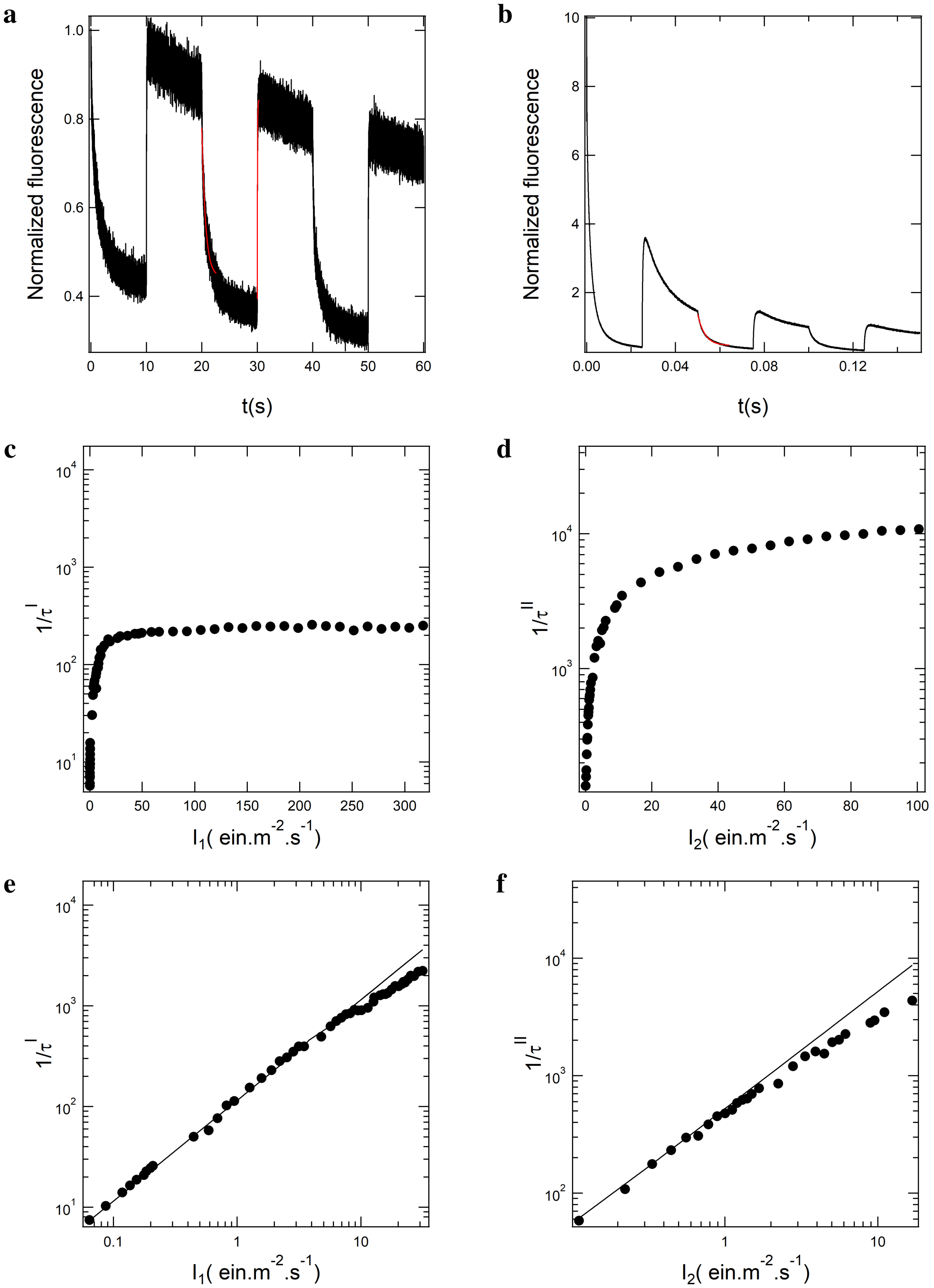

Supplementary Figure 28: Kinetic analysis of **8** photoswitching at 20 μM in pH 7.4 PBS (50 mM sodium phosphate, 150 mM NaCl); T = 298 K. **a**, **b**: Evolution of the fluorescence intensity scaled by the initial value upon continuous illumination of intensity I_1 at $\lambda_1 = 488$ nm and square-wave illumination of intensity I_2 at $\lambda_2 = 405$ nm (**a**: $I_1 = 0.05$ ein.m^{-2}.s^{-1}, $I_2 = 0.1$ ein.m^{-2}.s^{-1}; **b**: $I_1 = 200$ ein.m^{-2}.s^{-1}, $I_2 = 3$ ein.m^{-2}.s^{-1}). Black line: experimental data; red line: fitting functions according to Eq.(40) and Eq.(49); **c**: Illumination I: Inverse of the relaxation time $1/\tau^{\mathrm{I}}$ (disks) versus light intensity I_1 at $\lambda_1 = 488$ nm, **d**: Illumination II: Inverse of the relaxation time $1/\tau^{\mathrm{II}}$ (disks) versus light intensity I_2 at $\lambda_2 = 405$ nm with $I_1 = 0.2$ ein.m^{-2}.s^{-1}; **e**: Magnification of **c** at low light intensity. Solid line: linear fitting function yielding the photoswitching cross section Σ_1, **f**: Magnification of **d** at low light intensity. Solid line: linear fitting function yielding the photoswitching cross section Σ_2.

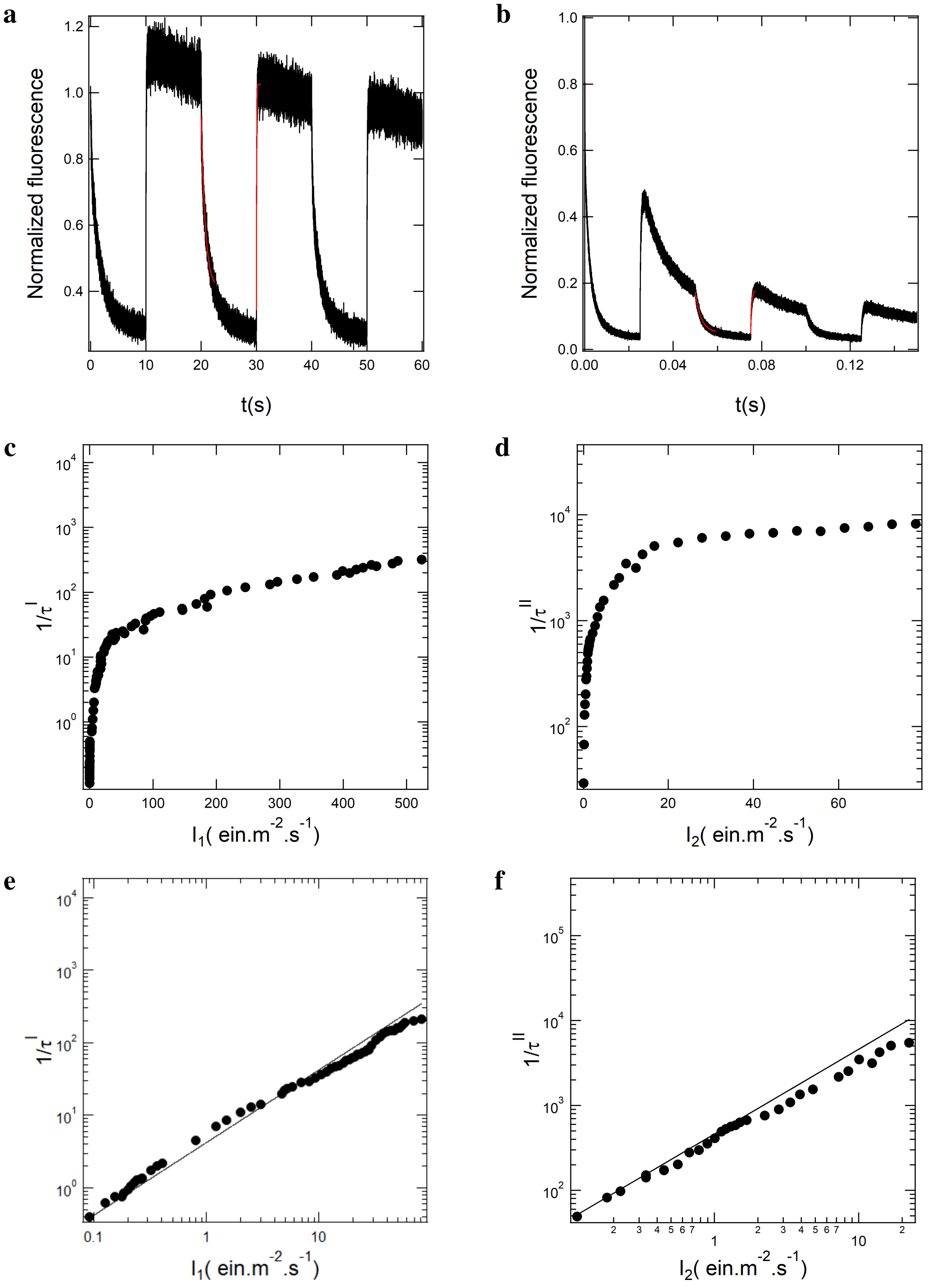

Supplementary Figure 29: Kinetic analysis of **9** photoswitching at 20 μM in pH 7.4 PBS (50 mM sodium phosphate, 150 mM NaCl); T = 298 K. **a**, **b**: Evolution of the fluorescence intensity scaled by the initial value upon continuous illumination of intensity I_1 at $\lambda_1 = 488$ nm and square-wave illumination of intensity I_2 at $\lambda_2 = 405$ nm (**a**: $I_1 = 0.05$ ein.m^{-2}.s^{-1}, $I_2 = 0.1$ ein.m^{-2}.s^{-1}; **b**: $I_1 = 200$ ein.m^{-2}.s^{-1}, $I_2 = 3$ ein.m^{-2}.s^{-1}). Black line: experimental data; red line: fitting functions according to Eq.(40) and Eq.(49); **c**: Illumination I: Inverse of the relaxation time $1/\tau^{\mathrm{I}}$ (disks) versus light intensity I_1 at $\lambda_1 = 488$ nm, **d**: Illumination II: Inverse of the relaxation time $1/\tau^{\mathrm{II}}$ (disks) versus light intensity I_2 at $\lambda_2 = 405$ nm with $I_1 = 0.2$ ein.m^{-2}.s^{-1}; **e**: Magnification of **c** at low light intensity. Solid line: linear fitting function yielding the photoswitching cross section Σ_1, **f**: Magnification of **d** at low light intensity. Solid line: linear fitting function yielding the photoswitching cross section Σ_2.

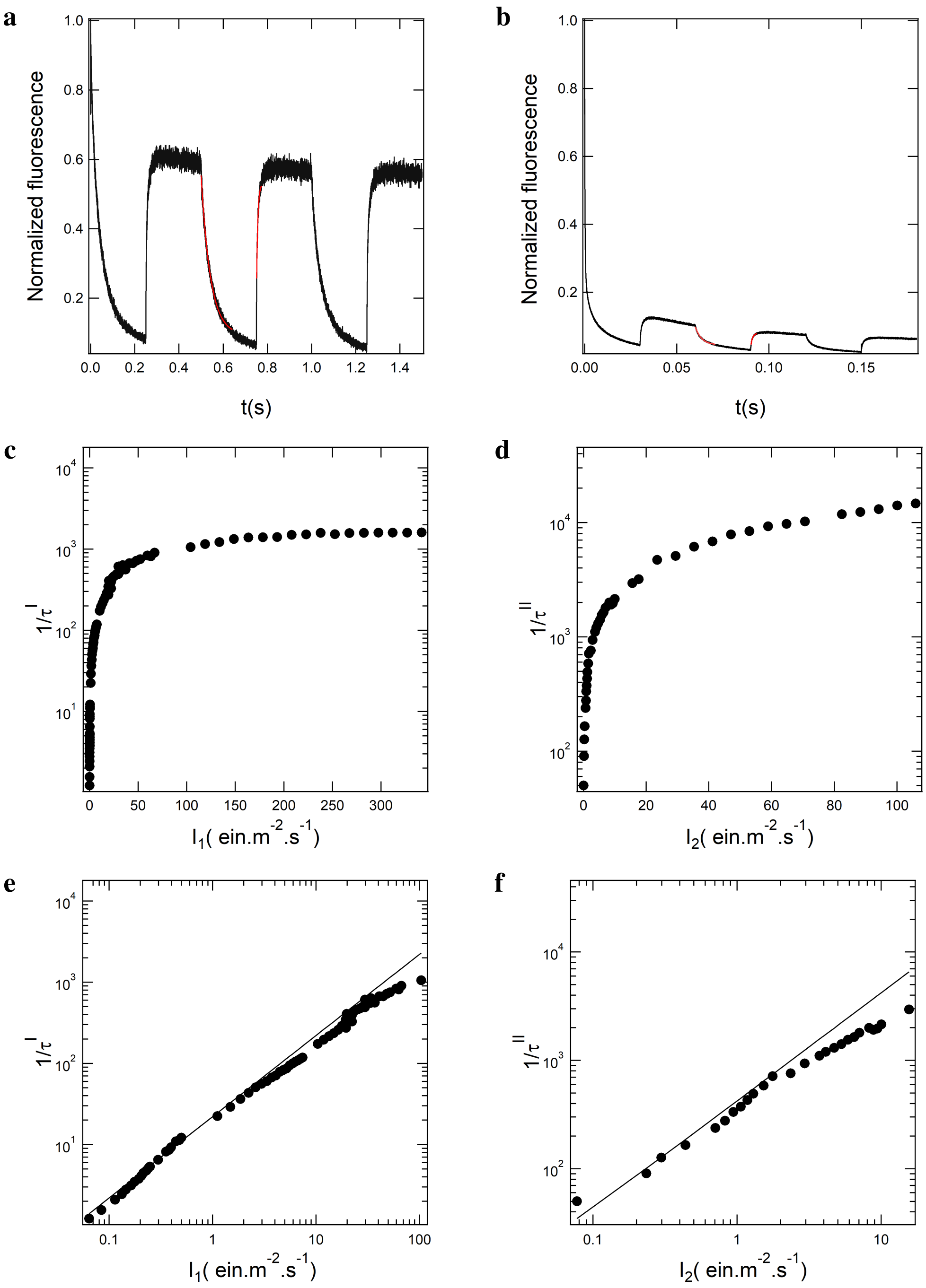

Supplementary Figure 30: Kinetic analysis of **10** photoswitching at 20 μM in pH 7.4 PBS (50 mM sodium phosphate, 150 mM NaCl); T = 298 K. **a**, **b**: Evolution of the fluorescence intensity scaled by the initial value upon continuous illumination of intensity I_1 at $\lambda_1 = 488$ nm and square-wave illumination of intensity I_2 at $\lambda_2 = 405$ nm (**a**: $I_1 = 0.05$ ein.m^{-2}.s^{-1}, $I_2 = 0.1$ ein.m^{-2}.s^{-1}; **b**: $I_1 = 200$ ein.m^{-2}.s^{-1}, $I_2 = 3$ ein.m^{-2}.s^{-1}). Black line: experimental data; red line: fitting functions according to Eq.(40) and Eq.(49); **c**: Illumination I: Inverse of the relaxation time $1/\tau^{\mathrm{I}}$ (disks) versus light intensity I_1 at $\lambda_1 = 488$ nm, **d**: Illumination II: Inverse of the relaxation time $1/\tau^{\mathrm{II}}$ (disks) versus light intensity I_2 at $\lambda_2 = 405$ nm with $I_1 = 0.2$ ein.m^{-2}.s^{-1}; **e**: Magnification of **c** at low light intensity. Solid line: linear fitting function yielding the photoswitching cross section Σ_1, **f**: Magnification of **d** at low light intensity. Solid line: linear fitting function yielding the photoswitching cross section Σ_2.

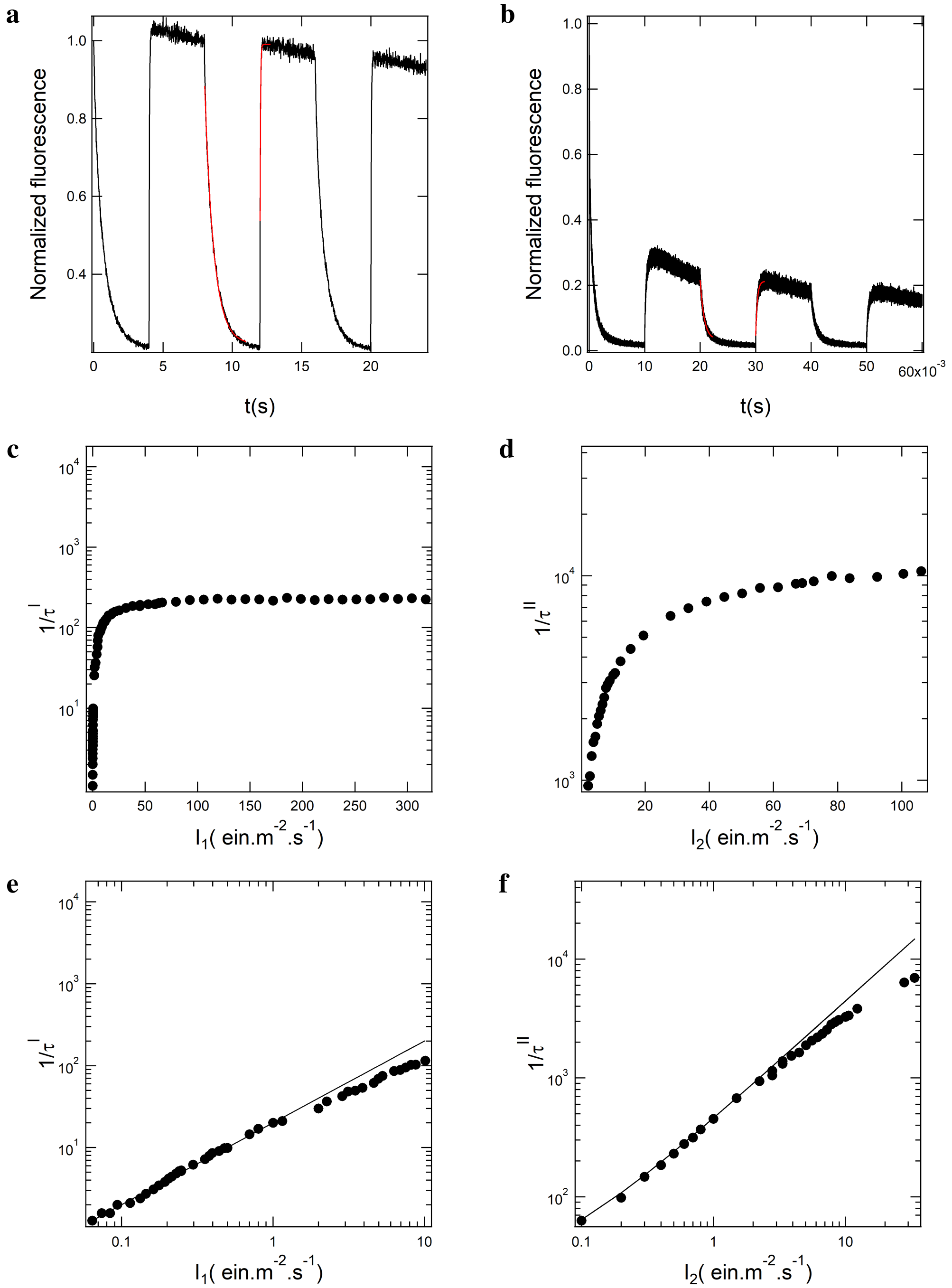

Supplementary Figure 31: Kinetic analysis of **11** photoswitching at 20 μM in pH 7.4 PBS (50 mM sodium phosphate, 150 mM NaCl); T = 298 K. **a**, **b**: Evolution of the fluorescence intensity scaled by the initial value upon continuous illumination of intensity I_1 at $\lambda_1 = 488$ nm and square-wave illumination of intensity I_2 at $\lambda_2 = 405$ nm (**a**: $I_1 = 0.05$ ein.m^{-2}.s^{-1}, $I_2 = 0.1$ ein.m^{-2}.s^{-1}; **b**: $I_1 = 200$ ein.m^{-2}.s^{-1}, $I_2 = 3$ ein.m^{-2}.s^{-1}). Black line: experimental data; red line: fitting functions according to Eq.(40) and Eq.(49); **c**: Illumination I: Inverse of the relaxation time $1/\tau^{\mathrm{I}}$ (disks) versus light intensity I_1 at $\lambda_1 = 488$ nm, **d**: Illumination II: Inverse of the relaxation time $1/\tau^{\mathrm{II}}$ (disks) versus light intensity I_2 at $\lambda_2 = 405$ nm with $I_1 = 0.2$ ein.m^{-2}.s^{-1}; **e**: Magnification of **c** at low light intensity. Solid line: linear fitting function yielding the photoswitching cross section Σ_1, **f**: Magnification of **d** at low light intensity. Solid line: linear fitting function yielding the photoswitching cross section Σ_2.

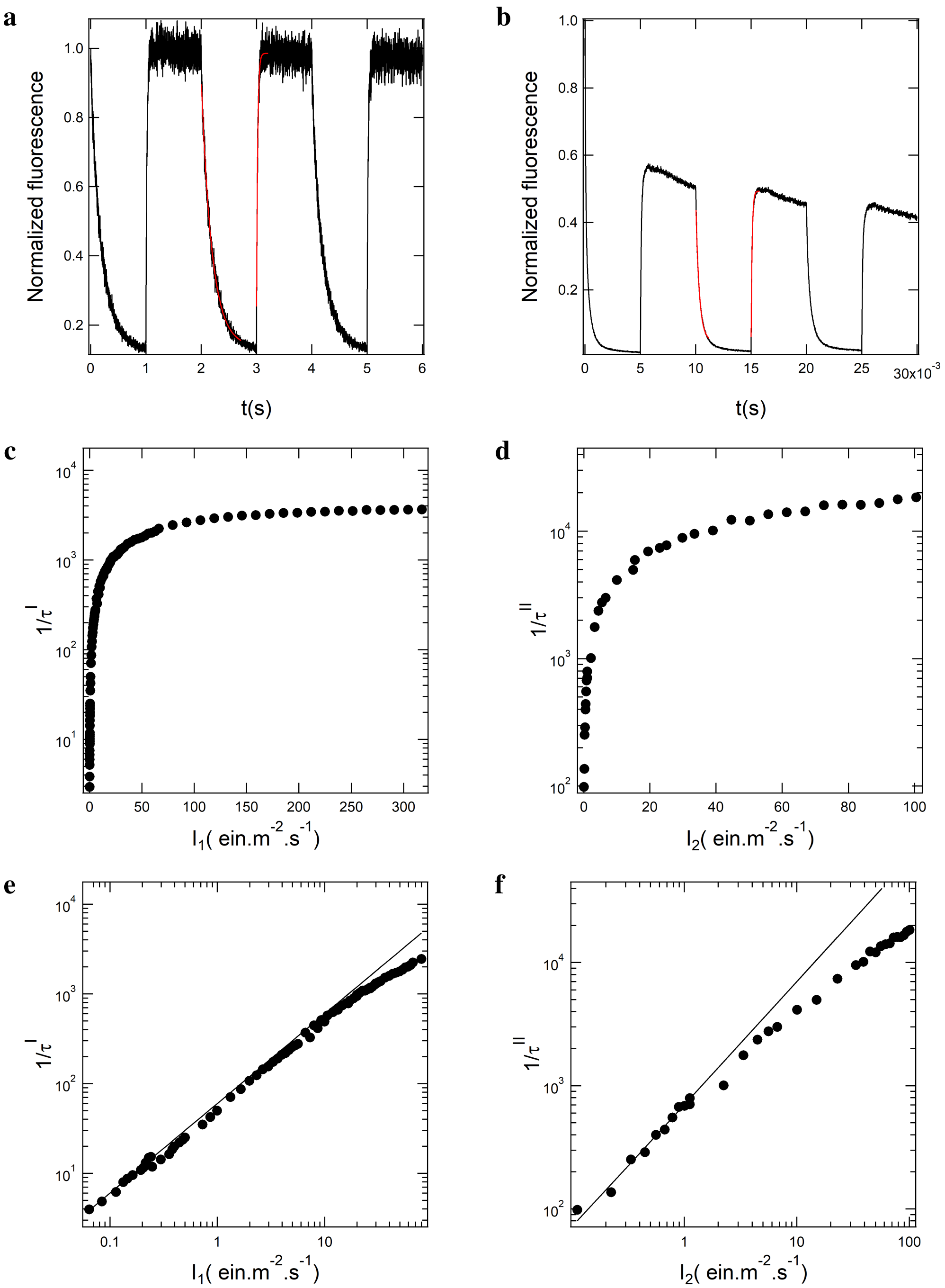

Supplementary Figure 32: Kinetic analysis of **12** photoswitching at 20 μM in pH 7.4 PBS (50 mM sodium phosphate, 150 mM NaCl); T = 298 K. **a**, **b**: Evolution of the fluorescence intensity scaled by the initial value upon continuous illumination of intensity I_1 at $\lambda_1 = 488$ nm and square-wave illumination of intensity I_2 at $\lambda_2 = 405$ nm (**a**: $I_1 = 0.05$ ein.m^{-2}.s^{-1}, $I_2 = 0.1$ ein.m^{-2}.s^{-1}; **b**: $I_1 = 200$ ein.m^{-2}.s^{-1}, $I_2 = 3$ ein.m^{-2}.s^{-1}). Black line: experimental data; red line: fitting functions according to Eq.(40) and Eq.(49); **c**: Illumination I: Inverse of the relaxation time $1/\tau^{\mathrm{I}}$ (disks) versus light intensity I_1 at $\lambda_1 = 488$ nm, **d**: Illumination II: Inverse of the relaxation time $1/\tau^{\mathrm{II}}$ (disks) versus light intensity I_2 at $\lambda_2 = 405$ nm with $I_1 = 0.2$ ein.m^{-2}.s^{-1}; **e**: Magnification of **c** at low light intensity. Solid line: linear fitting function yielding the photoswitching cross section Σ_1, **f**: Magnification of **d** at low light intensity. Solid line: linear fitting function yielding the photoswitching cross section Σ_2.

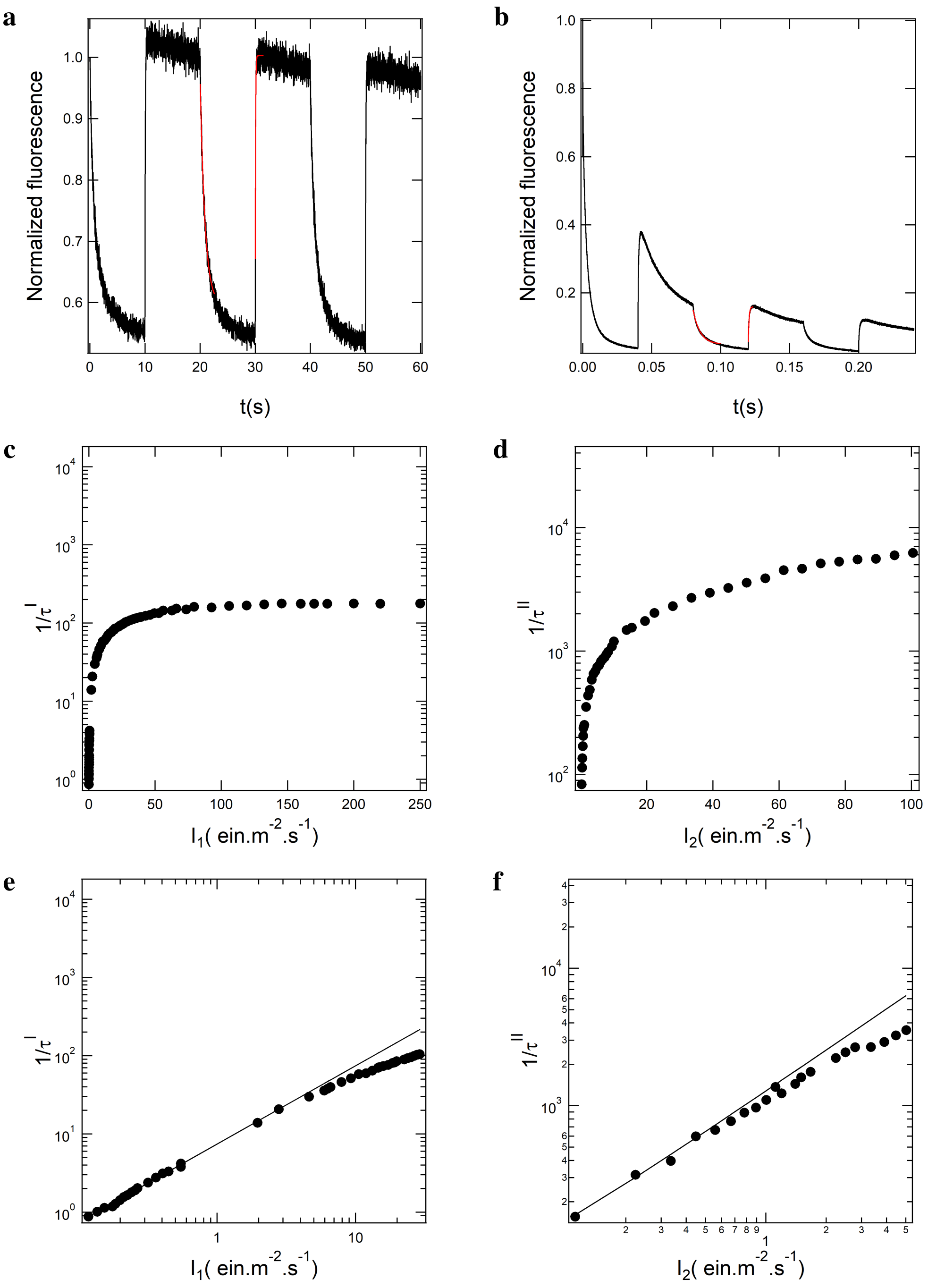

Supplementary Figure 33: Kinetic analysis of **13** photoswitching at 20 μM in pH 7.4 PBS (50 mM sodium phosphate, 150 mM NaCl); T = 298 K. **a**, **b**: Evolution of the fluorescence intensity scaled by the initial value upon continuous illumination of intensity I_1 at $\lambda_1 = 488$ nm and square-wave illumination of intensity I_2 at $\lambda_2 = 405$ nm (**a**: $I_1 = 0.05$ ein.m^{-2}.s^{-1}, $I_2 = 0.1$ ein.m^{-2}.s^{-1}; **b**: $I_1 = 200$ ein.m^{-2}.s^{-1}, $I_2 = 3$ ein.m^{-2}.s^{-1}). Black line: experimental data; red line: fitting functions according to Eq.(40) and Eq.(49); **c**: Illumination I: Inverse of the relaxation time $1/\tau^{\mathrm{I}}$ (disks) versus light intensity I_1 at $\lambda_1 = 488$ nm, **d**: Illumination II: Inverse of the relaxation time $1/\tau^{\mathrm{II}}$ (disks) versus light intensity I_2 at $\lambda_2 = 405$ nm with $I_1 = 0.2$ ein.m^{-2}.s^{-1}; **e**: Magnification of **c** at low light intensity. Solid line: linear fitting function yielding the photoswitching cross section Σ_1, **f**: Magnification of **d** at low light intensity. Solid line: linear fitting function yielding the photoswitching cross section Σ_2.

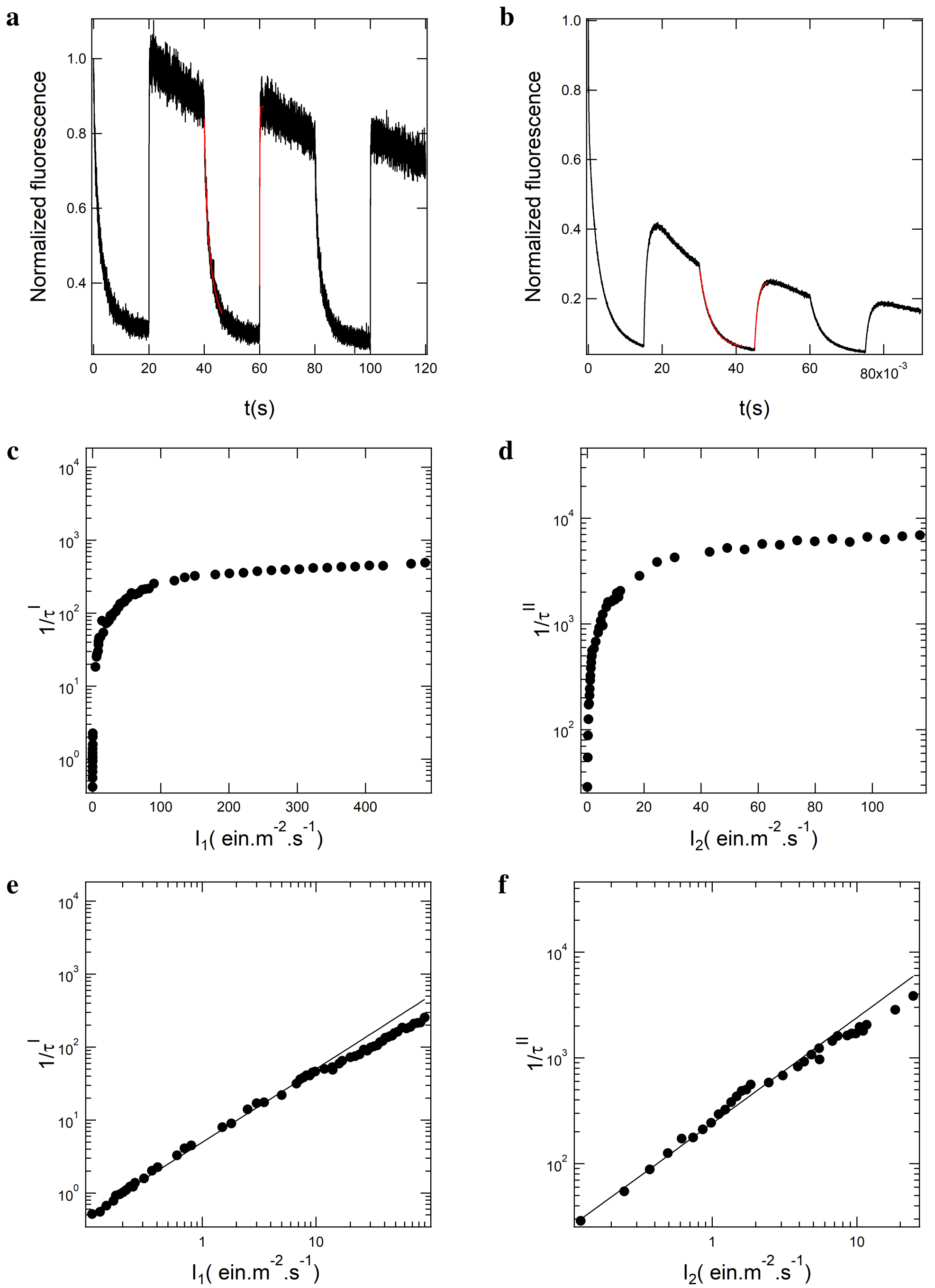

Supplementary Figure 34: Kinetic analysis of **14** photoswitching at 20 μM in pH 7.4 PBS (50 mM sodium phosphate, 150 mM NaCl); T = 298 K. **a**, **b**: Evolution of the fluorescence intensity scaled by the initial value upon continuous illumination of intensity I_1 at $\lambda_1 = 488$ nm and square-wave illumination of intensity I_2 at $\lambda_2 = 405$ nm (**a**: $I_1 = 0.05$ ein.m^{-2}.s^{-1}, $I_2 = 0.1$ ein.m^{-2}.s^{-1}; **b**: $I_1 = 200$ ein.m^{-2}.s^{-1}, $I_2 = 3$ ein.m^{-2}.s^{-1}). Black line: experimental data; red line: fitting functions according to Eq.(40) and Eq.(49); **c**: Illumination I: Inverse of the relaxation time $1/\tau^{\mathrm{I}}$ (disks) versus light intensity I_1 at $\lambda_1 = 488$ nm, **d**: Illumination II: Inverse of the relaxation time $1/\tau^{\mathrm{II}}$ (disks) versus light intensity I_2 at $\lambda_2 = 405$ nm with $I_1 = 0.2$ ein.m^{-2}.s^{-1}; **e**: Magnification of **c** at low light intensity. Solid line: linear fitting function yielding the photoswitching cross section Σ_1, **f**: Magnification of **d** at low light intensity. Solid line: linear fitting function yielding the photoswitching cross section Σ_2.

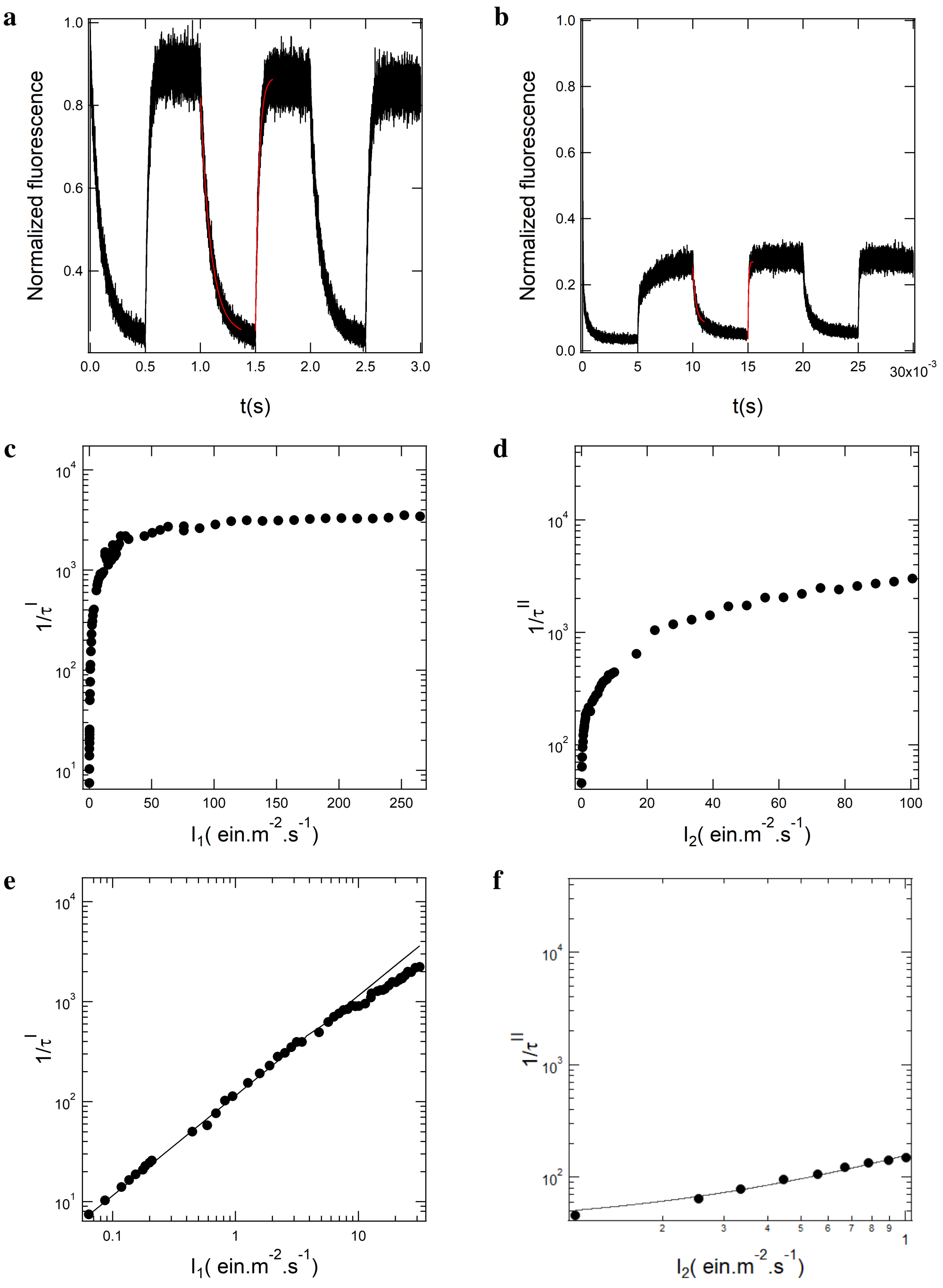

Supplementary Figure 35: Kinetic analysis of **15** photoswitching at 20 μM in pH 7.4 PBS (50 mM sodium phosphate, 150 mM NaCl); T = 298 K. **a**, **b**: Evolution of the fluorescence intensity scaled by the initial value upon continuous illumination of intensity I_1 at $\lambda_1 = 488$ nm and square-wave illumination of intensity I_2 at $\lambda_2 = 405$ nm (**a**: $I_1 = 0.05$ ein.m^{-2}.s^{-1}, $I_2 = 0.1$ ein.m^{-2}.s^{-1}; **b**: $I_1 = 200$ ein.m^{-2}.s^{-1}, $I_2 = 3$ ein.m^{-2}.s^{-1}). Black line: experimental data; red line: fitting functions according to Eq.(40) and Eq.(49); **c**: Illumination I: Inverse of the relaxation time $1/\tau^{\mathrm{I}}$ (disks) versus light intensity I_1 at $\lambda_1 = 488$ nm, **d**: Illumination II: Inverse of the relaxation time $1/\tau^{\mathrm{II}}$ (disks) versus light intensity I_2 at $\lambda_2 = 405$ nm with $I_1 = 0.2$ ein.m^{-2}.s^{-1}; **e**: Magnification of **c** at low light intensity. Solid line: linear fitting function yielding the photoswitching cross section Σ_1, **f**: Magnification of **d** at low light intensity. Solid line: linear fitting function yielding the photoswitching cross section Σ_2.

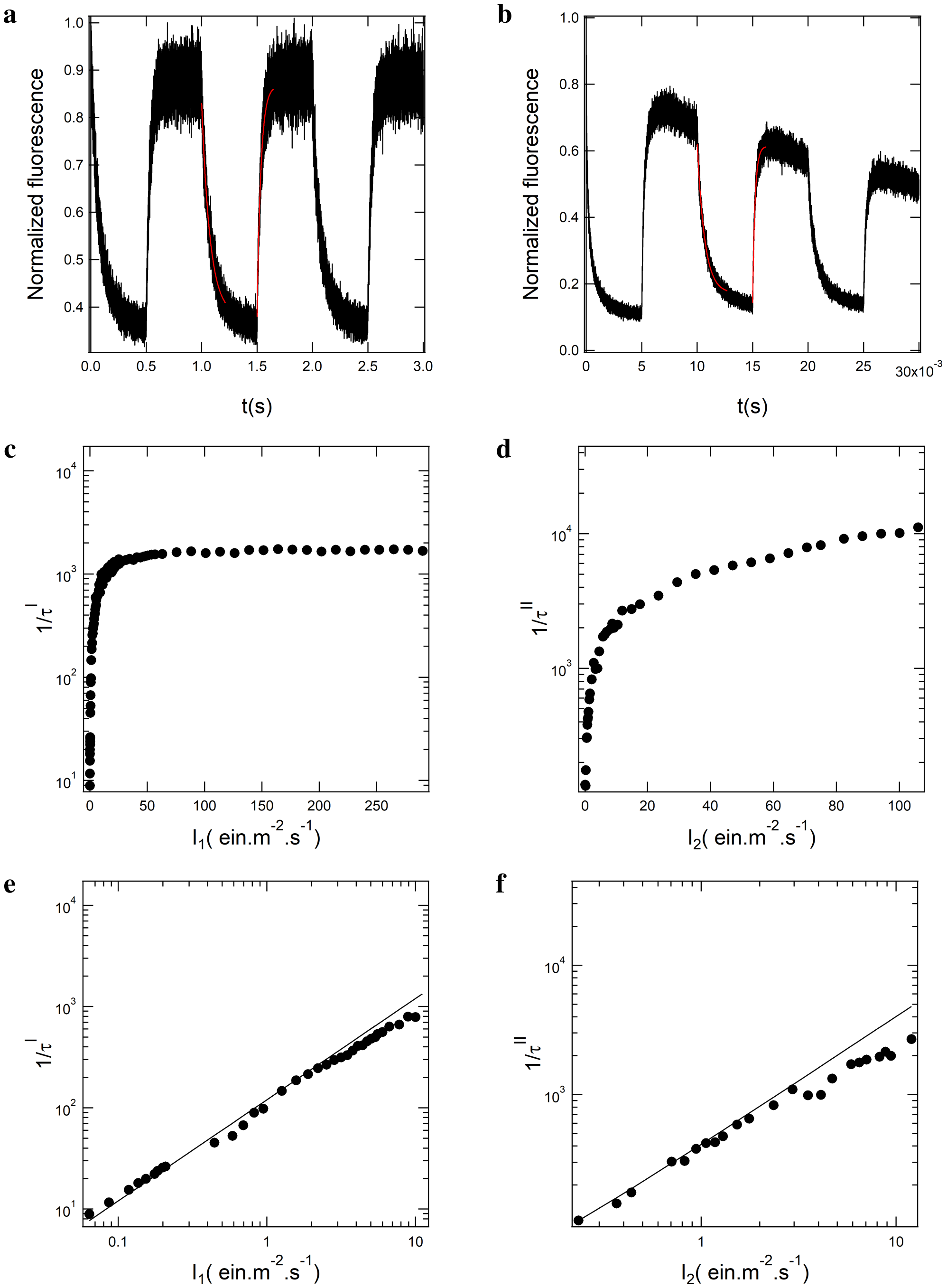

Supplementary Figure 36: Kinetic analysis of **16** photoswitching at 20 μM in pH 7.4 PBS (50 mM sodium phosphate, 150 mM NaCl); T = 298 K. **a**, **b**: Evolution of the fluorescence intensity scaled by the initial value upon continuous illumination of intensity I_1 at $\lambda_1 = 488$ nm and square-wave illumination of intensity I_2 at $\lambda_2 = 405$ nm (**a**: $I_1 = 0.05$ ein.m^{-2}.s^{-1}, $I_2 = 0.1$ ein.m^{-2}.s^{-1}; **b**: $I_1 = 200$ ein.m^{-2}.s^{-1}, $I_2 = 3$ ein.m^{-2}.s^{-1}). Black line: experimental data; red line: fitting functions according to Eq.(40) and Eq.(49); **c**: Illumination I: Inverse of the relaxation time $1/\tau^{\mathrm{I}}$ (disks) versus light intensity I_1 at $\lambda_1 = 488$ nm, **d**: Illumination II: Inverse of the relaxation time $1/\tau^{\mathrm{II}}$ (disks) versus light intensity I_2 at $\lambda_2 = 405$ nm with $I_1 = 0.2$ ein.m^{-2}.s^{-1}; **e**: Magnification of **c** at low light intensity. Solid line: linear fitting function yielding the photoswitching cross section Σ_1, **f**: Magnification of **d** at low light intensity. Solid line: linear fitting function yielding the photoswitching cross section Σ_2.

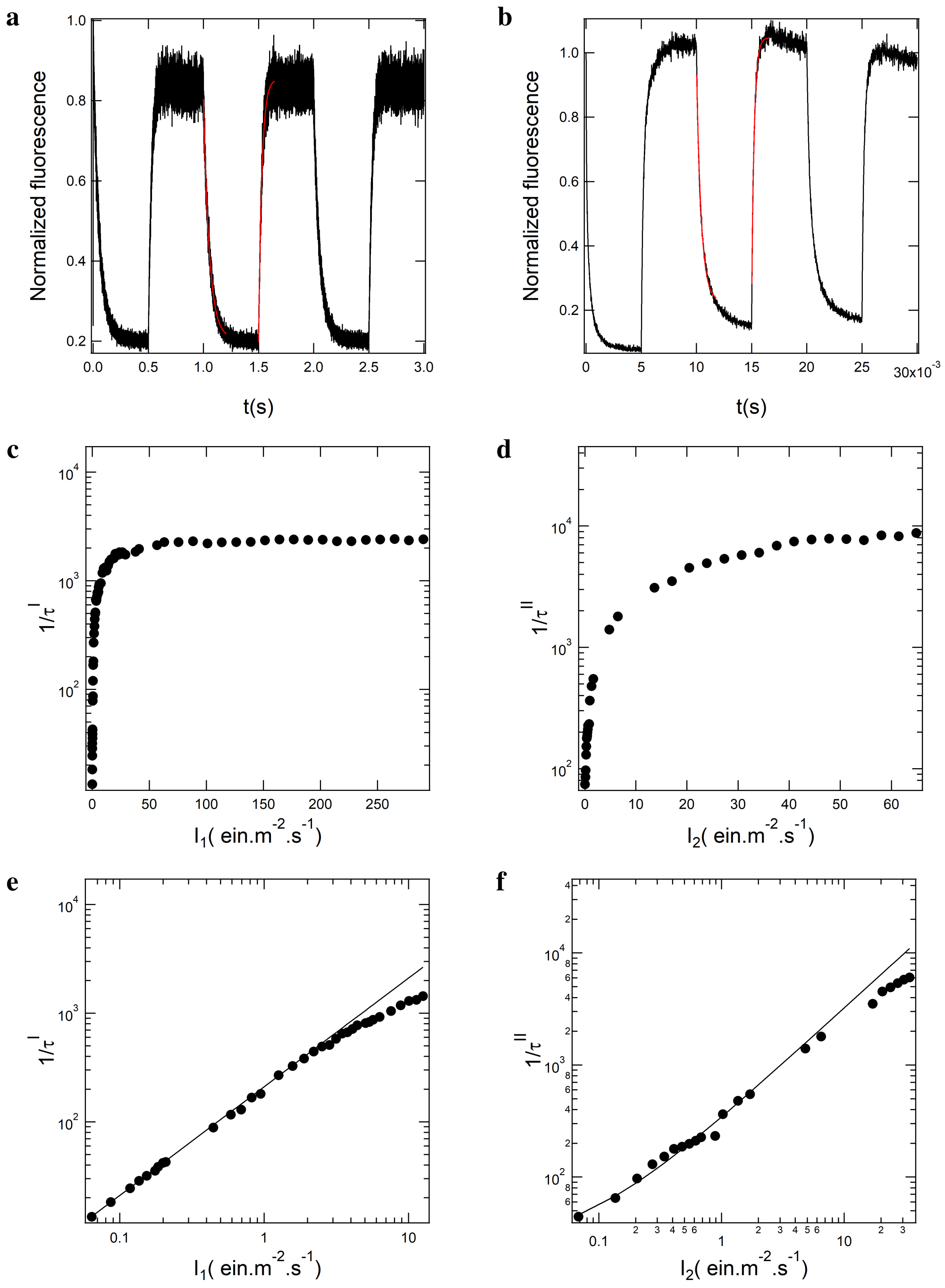

Supplementary Figure 37: Kinetic analysis of **17** photoswitching at 20 μM in pH 7.4 PBS (50 mM sodium phosphate, 150 mM NaCl); T = 298 K. **a**, **b**: Evolution of the fluorescence intensity scaled by the initial value upon continuous illumination of intensity I_1 at $\lambda_1 = 488$ nm and square-wave illumination of intensity I_2 at $\lambda_2 = 405$ nm (**a**: $I_1 = 0.05$ ein.m^{-2}.s^{-1}, $I_2 = 0.1$ ein.m^{-2}.s^{-1}; **b**: $I_1 = 200$ ein.m^{-2}.s^{-1}, $I_2 = 3$ ein.m^{-2}.s^{-1}). Black line: experimental data; red line: fitting functions according to Eq.(40) and Eq.(49); **c**: Illumination I: Inverse of the relaxation time $1/\tau^{\mathrm{I}}$ (disks) versus light intensity I_1 at $\lambda_1 = 488$ nm, **d**: Illumination II: Inverse of the relaxation time $1/\tau^{\mathrm{II}}$ (disks) versus light intensity I_2 at $\lambda_2 = 405$ nm with $I_1 = 0.2$ ein.m^{-2}.s^{-1}; **e**: Magnification of **c** at low light intensity. Solid line: linear fitting function yielding the photoswitching cross section Σ_1, **f**: Magnification of **d** at low light intensity. Solid line: linear fitting function yielding the photoswitching cross section Σ_2.

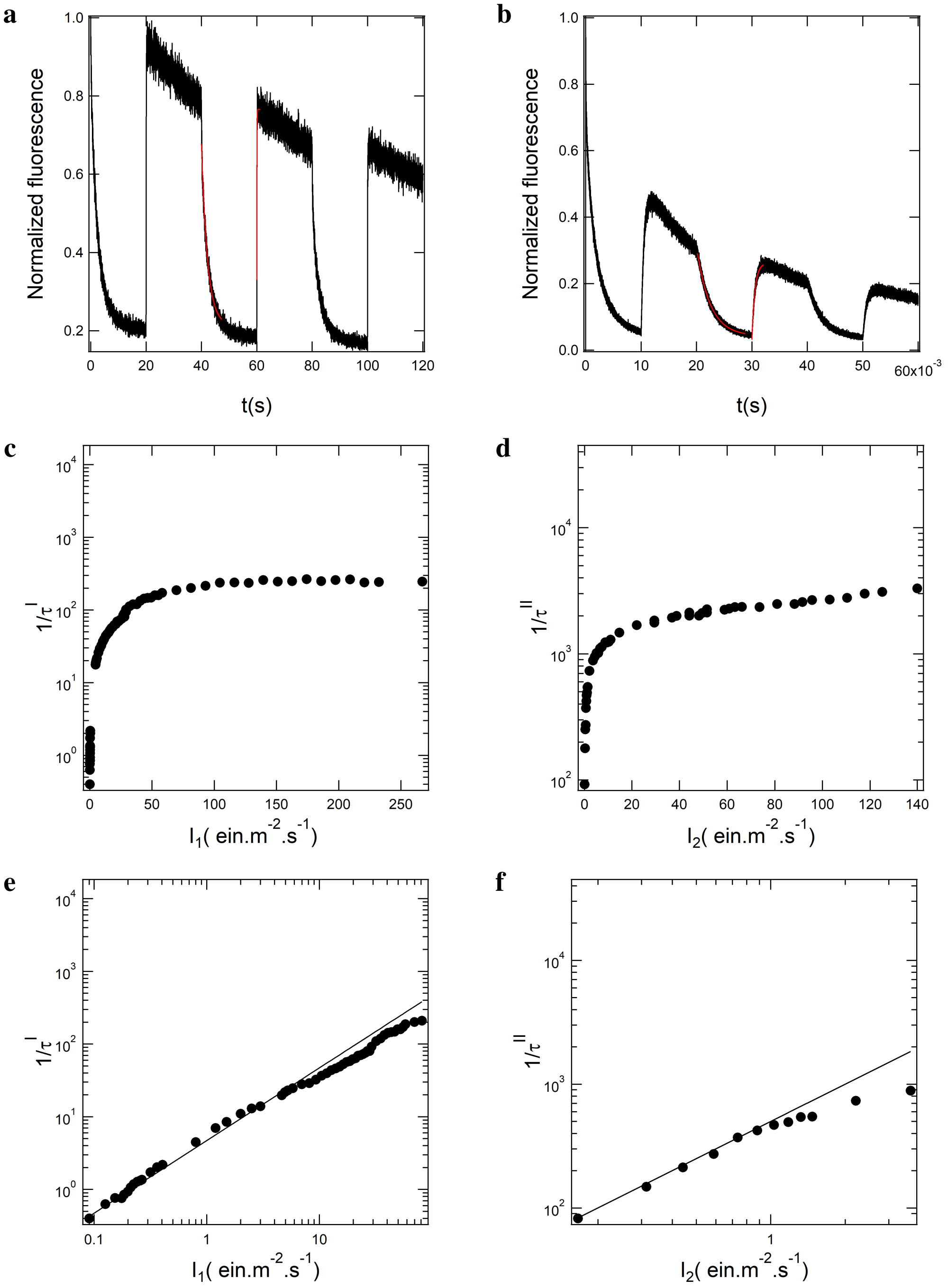

Supplementary Figure 38: Kinetic analysis of **18** photoswitching at 20 μM in pH 7.4 PBS (50 mM sodium phosphate, 150 mM NaCl); T = 298 K. **a**, **b**: Evolution of the fluorescence intensity scaled by the initial value upon continuous illumination of intensity I_1 at $\lambda_1 = 488$ nm and square-wave illumination of intensity I_2 at $\lambda_2 = 405$ nm (**a**: $I_1 = 0.05$ ein.m^{-2}.s^{-1}, $I_2 = 0.1$ ein.m^{-2}.s^{-1}; **b**: $I_1 = 200$ ein.m^{-2}.s^{-1}, $I_2 = 3$ ein.m^{-2}.s^{-1}). Black line: experimental data; red line: fitting functions according to Eq.(40) and Eq.(49); **c**: Illumination I: Inverse of the relaxation time $1/\tau^{\mathrm{I}}$ (disks) versus light intensity I_1 at $\lambda_1 = 488$ nm, **d**: Illumination II: Inverse of the relaxation time $1/\tau^{\mathrm{II}}$ (disks) versus light intensity I_2 at $\lambda_2 = 405$ nm with $I_1 = 0.2$ ein.m^{-2}.s^{-1}; **e**: Magnification of **c** at low light intensity. Solid line: linear fitting function yielding the photoswitching cross section Σ_1, **f**: Magnification of **d** at low light intensity. Solid line: linear fitting function yielding the photoswitching cross section Σ_2.

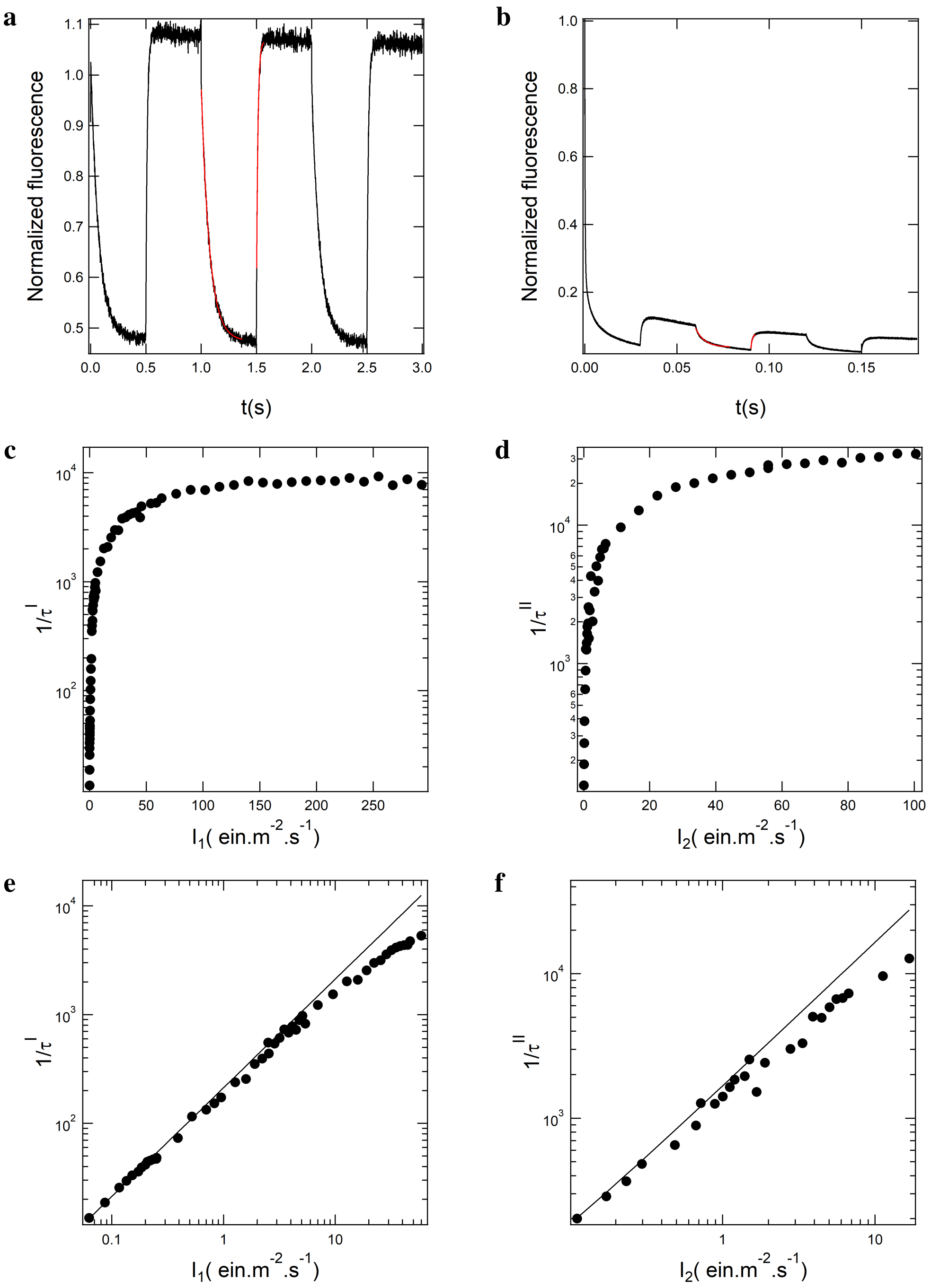

Supplementary Figure 39: Kinetic analysis of **19** photoswitching at 20 μM in pH 7.4 PBS (50 mM sodium phosphate, 150 mM NaCl); T = 298 K. **a**, **b**: Evolution of the fluorescence intensity scaled by the initial value upon continuous illumination of intensity I_1 at $\lambda_1 = 488$ nm and square-wave illumination of intensity I_2 at $\lambda_2 = 405$ nm (**a**: $I_1 = 0.05$ ein.m^{-2}.s^{-1}, $I_2 = 0.1$ ein.m^{-2}.s^{-1}; **b**: $I_1 = 200$ ein.m^{-2}.s^{-1}, $I_2 = 3$ ein.m^{-2}.s^{-1}). Black line: experimental data; red line: fitting functions according to Eq.(40) and Eq.(49); **c**: Illumination I: Inverse of the relaxation time $1/\tau^{\mathrm{I}}$ (disks) versus light intensity I_1 at $\lambda_1 = 488$ nm, **d**: Illumination II: Inverse of the relaxation time $1/\tau^{\mathrm{II}}$ (disks) versus light intensity I_2 at $\lambda_2 = 405$ nm with $I_1 = 0.2$ ein.m^{-2}.s^{-1}; **e**: Magnification of **c** at low light intensity. Solid line: linear fitting function yielding the photoswitching cross section Σ_1, **f**: Magnification of **d** at low light intensity. Solid line: linear fitting function yielding the photoswitching cross section Σ_2.

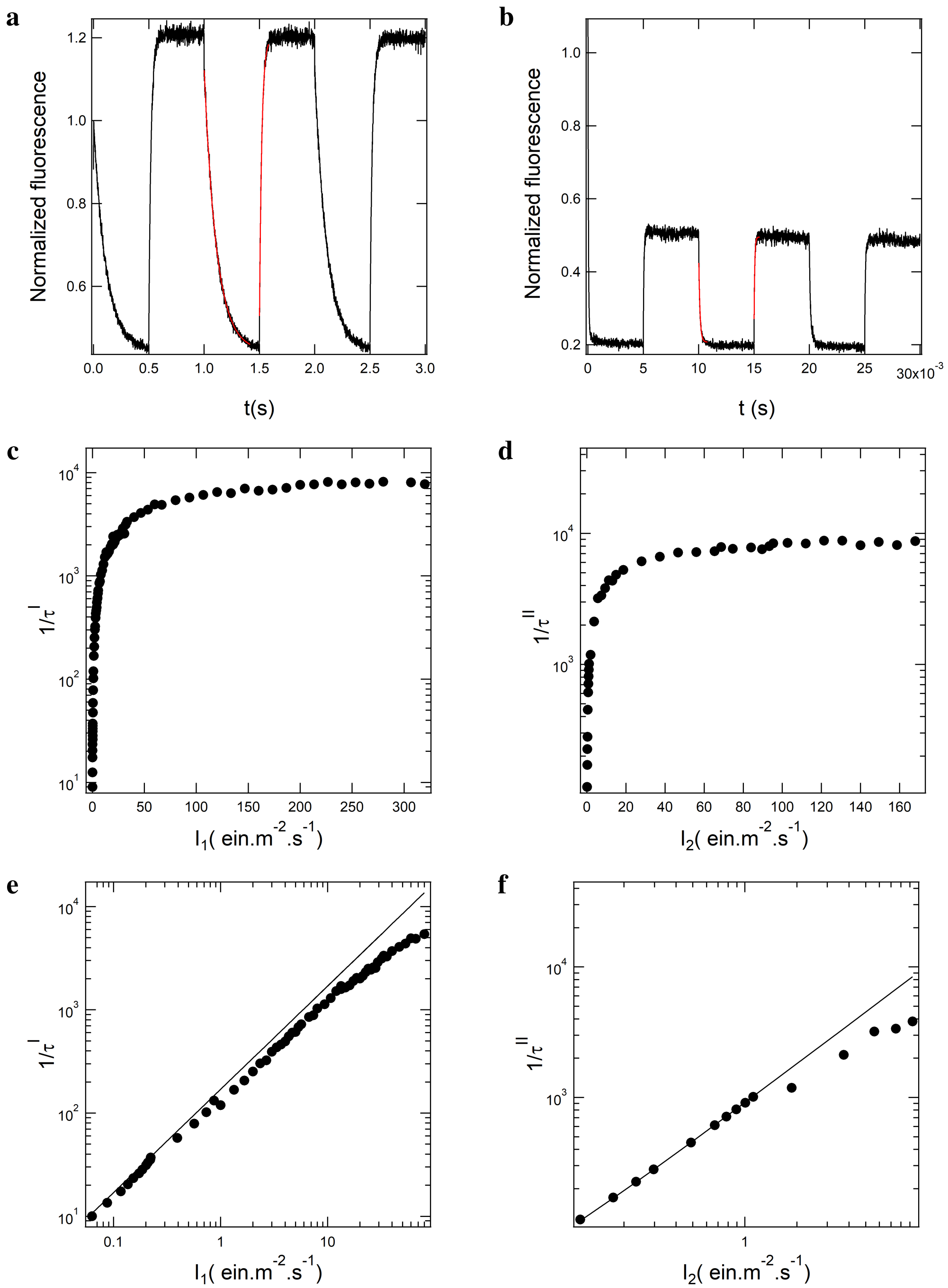

Supplementary Figure 40: Kinetic analysis of **20** photoswitching at 20 μM in pH 7.4 PBS (50 mM sodium phosphate, 150 mM NaCl); T = 298 K. **a**, **b**: Evolution of the fluorescence intensity scaled by the initial value upon continuous illumination of intensity I_1 at $\lambda_1 = 488$ nm and square-wave illumination of intensity I_2 at $\lambda_2 = 405$ nm (**a**: $I_1 = 0.05$ ein.m^{-2}.s^{-1}, $I_2 = 0.1$ ein.m^{-2}.s^{-1}; **b**: $I_1 = 200$ ein.m^{-2}.s^{-1}, $I_2 = 3$ ein.m^{-2}.s^{-1}). Black line: experimental data; red line: fitting functions according to Eq.(40) and Eq.(49); **c**: Illumination I: Inverse of the relaxation time $1/\tau^{\mathrm{I}}$ (disks) versus light intensity I_1 at $\lambda_1 = 488$ nm, **d**: Illumination II: Inverse of the relaxation time $1/\tau^{\mathrm{II}}$ (disks) versus light intensity I_2 at $\lambda_2 = 405$ nm with $I_1 = 0.2$ ein.m^{-2}.s^{-1}; **e**: Magnification of **c** at low light intensity. Solid line: linear fitting function yielding the photoswitching cross section Σ_1, **f**: Magnification of **d** at low light intensity. Solid line: linear fitting function yielding the photoswitching cross section Σ_2.

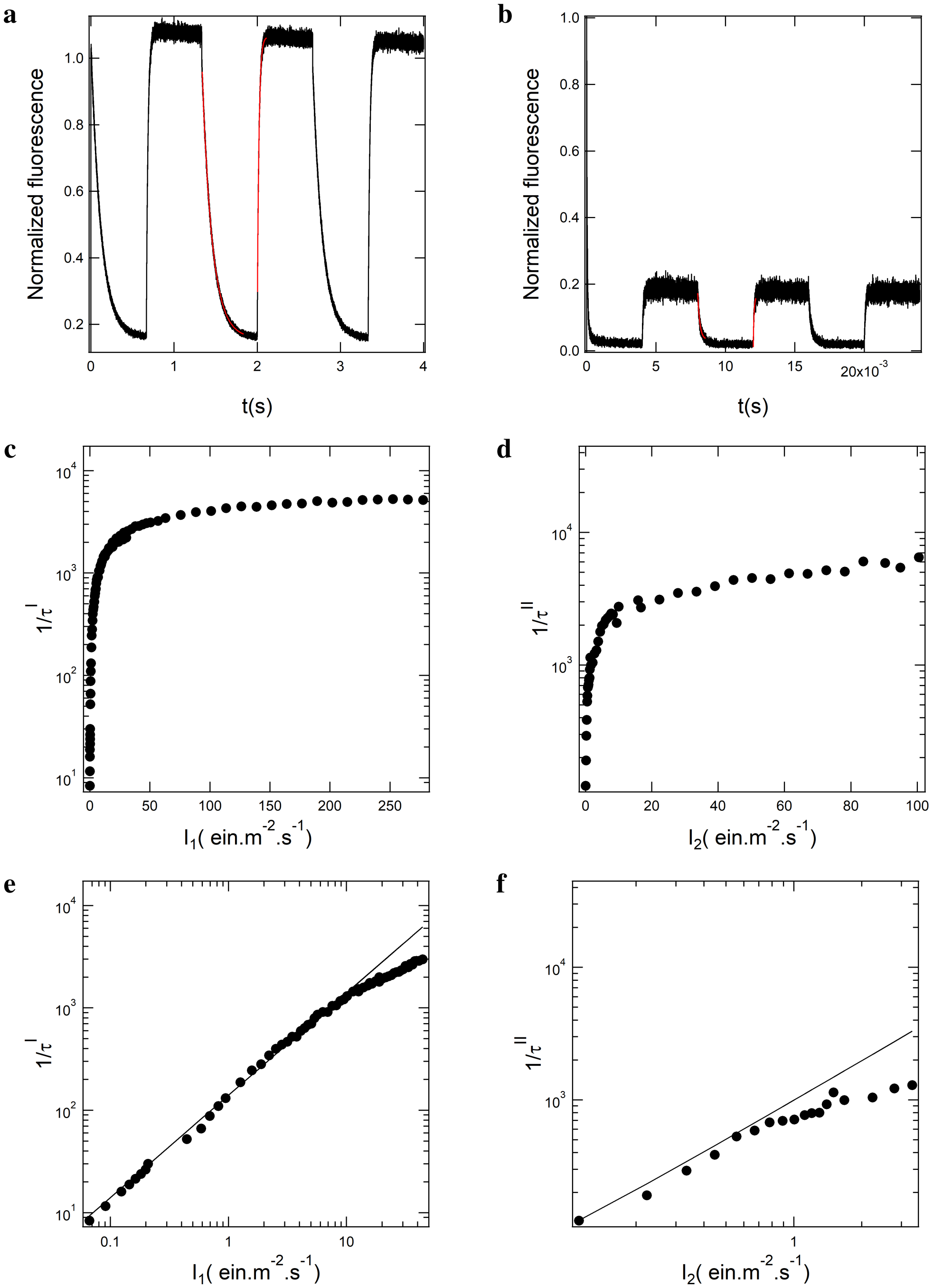

Supplementary Figure 41: Kinetic analysis of **21** photoswitching at 20 μM in pH 7.4 PBS (50 mM sodium phosphate, 150 mM NaCl); T = 298 K. **a**, **b**: Evolution of the fluorescence intensity scaled by the initial value upon continuous illumination of intensity I_1 at $\lambda_1 = 488$ nm and square-wave illumination of intensity I_2 at $\lambda_2 = 405$ nm (**a**: $I_1 = 0.05$ ein.m^{-2}.s^{-1}, $I_2 = 0.1$ ein.m^{-2}.s^{-1}; **b**: $I_1 = 200$ ein.m^{-2}.s^{-1}, $I_2 = 3$ ein.m^{-2}.s^{-1}). Black line: experimental data; red line: fitting functions according to Eq.(40) and Eq.(49); **c**: Illumination I: Inverse of the relaxation time $1/\tau^{\mathrm{I}}$ (disks) versus light intensity I_1 at $\lambda_1 = 488$ nm, **d**: Illumination II: Inverse of the relaxation time $1/\tau^{\mathrm{II}}$ (disks) versus light intensity I_2 at $\lambda_2 = 405$ nm with $I_1 = 0.2$ ein.m^{-2}.s^{-1}; **e**: Magnification of **c** at low light intensity. Solid line: linear fitting function yielding the photoswitching cross section Σ_1, **f**: Magnification of **d** at low light intensity. Solid line: linear fitting function yielding the photoswitching cross section Σ_2.

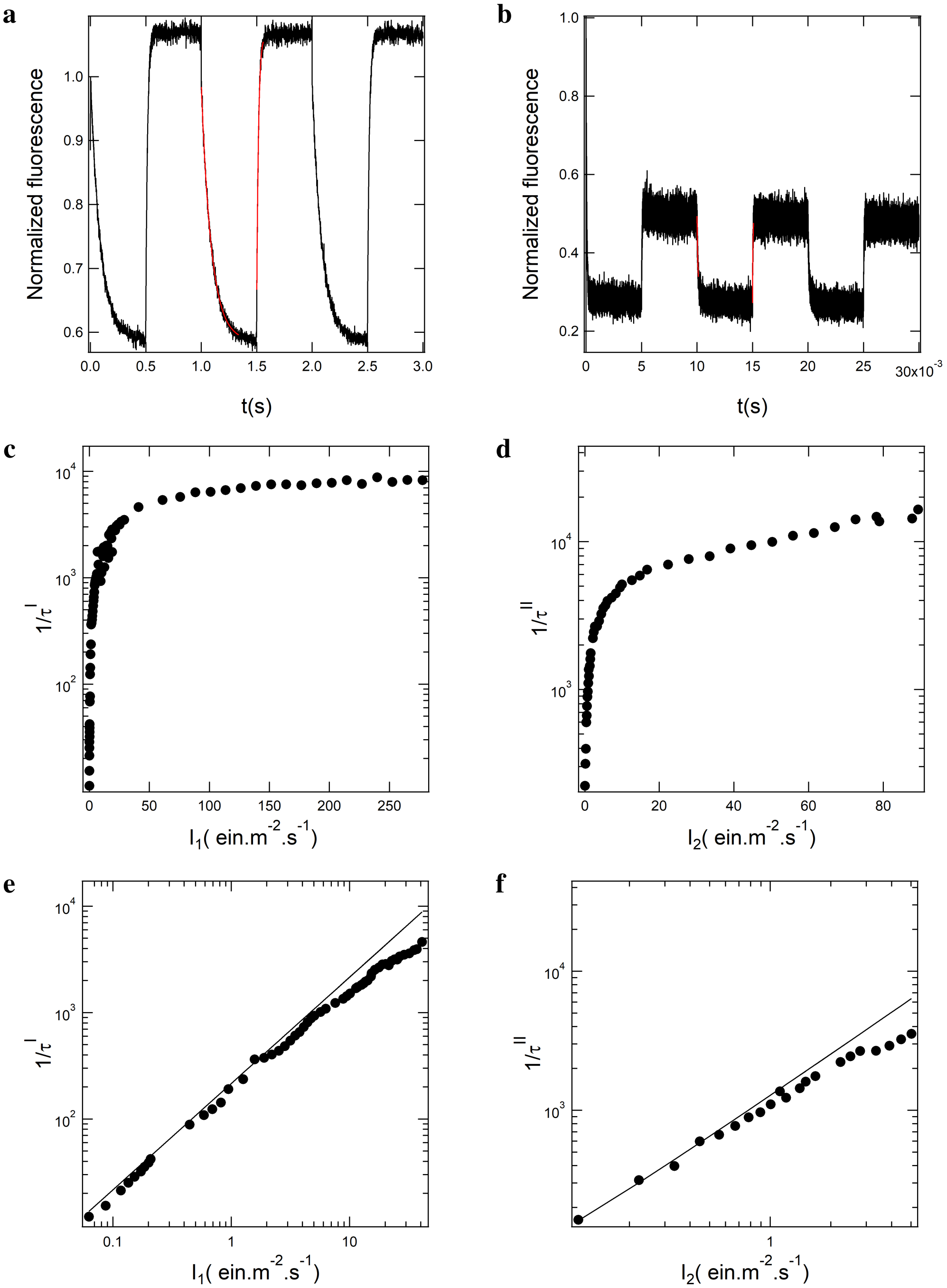

Supplementary Figure 42: Kinetic analysis of **22** photoswitching at 20 μM in pH 7.4 PBS (50 mM sodium phosphate, 150 mM NaCl); T = 298 K. **a**, **b**: Evolution of the fluorescence intensity scaled by the initial value upon continuous illumination of intensity I_1 at $\lambda_1 = 488$ nm and square-wave illumination of intensity I_2 at $\lambda_2 = 405$ nm (**a**: $I_1 = 0.05$ ein.m^{-2}.s^{-1}, $I_2 = 0.1$ ein.m^{-2}.s^{-1}; **b**: $I_1 = 200$ ein.m^{-2}.s^{-1}, $I_2 = 3$ ein.m^{-2}.s^{-1}). Black line: experimental data; red line: fitting functions according to Eq.(40) and Eq.(49); **c**: Illumination I: Inverse of the relaxation time $1/\tau^{\mathrm{I}}$ (disks) versus light intensity I_1 at $\lambda_1 = 488$ nm, **d**: Illumination II: Inverse of the relaxation time $1/\tau^{\mathrm{II}}$ (disks) versus light intensity I_2 at $\lambda_2 = 405$ nm with $I_1 = 0.2$ ein.m^{-2}.s^{-1}; **e**: Magnification of **c** at low light intensity. Solid line: linear fitting function yielding the photoswitching cross section Σ_1, **f**: Magnification of **d** at low light intensity. Solid line: linear fitting function yielding the photoswitching cross section Σ_2.

2.4 Photochemical characterization of the RSFP-labeled bacteria

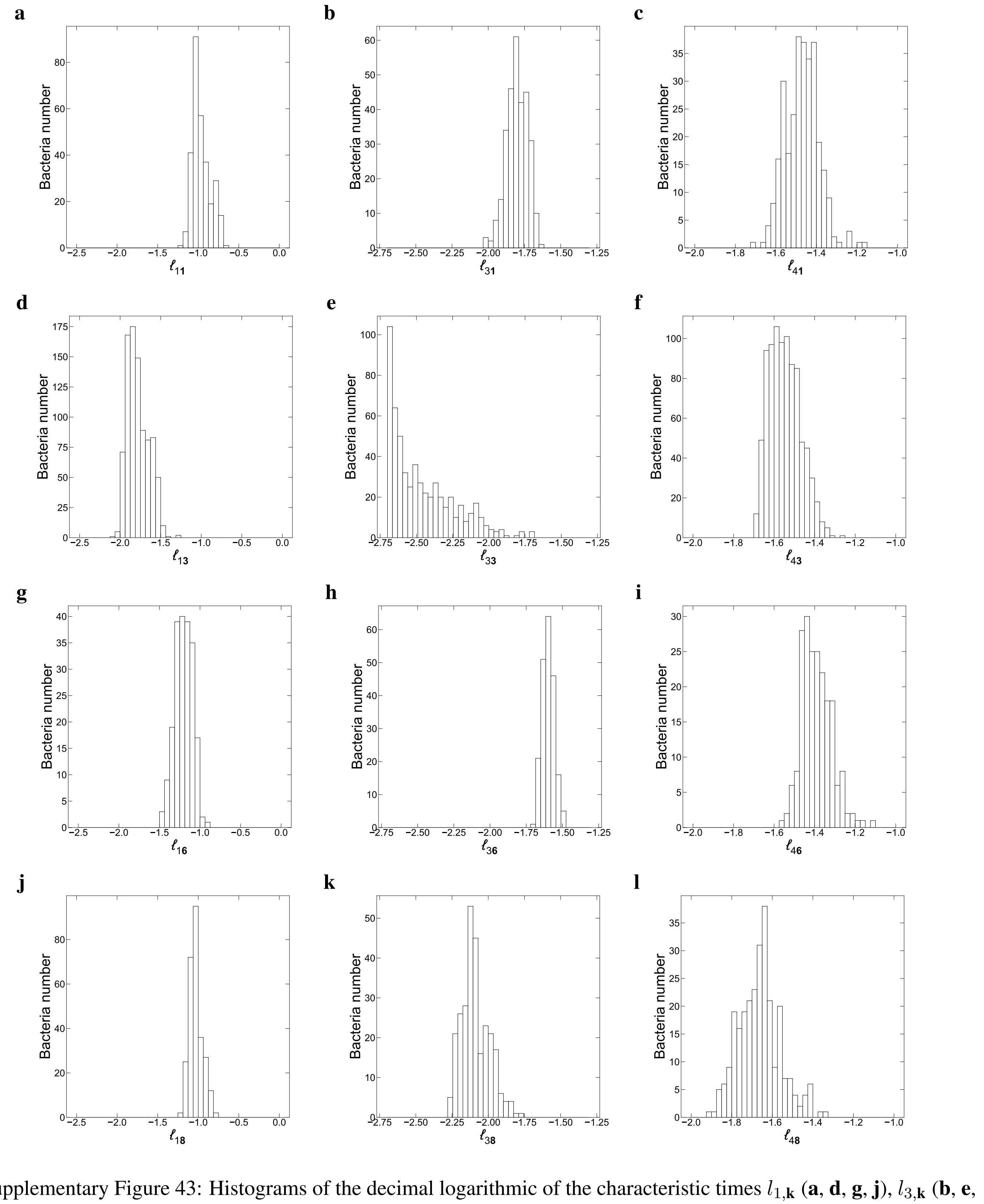

Supplementary Figure 43: Histograms of the decimal logarithmic of the characteristic times $l_{1,\mathbf{k}}$ (**a**, **d**, **g**, **j**), $l_{3,\mathbf{k}}$ (**b**, **e**, **h**, **k**), and $l_{4,\mathbf{k}}$ (**c**, **f**, **i**, **l**) for the RSFP-labeled bacteria **1**, **3**, **6**, and **8**.

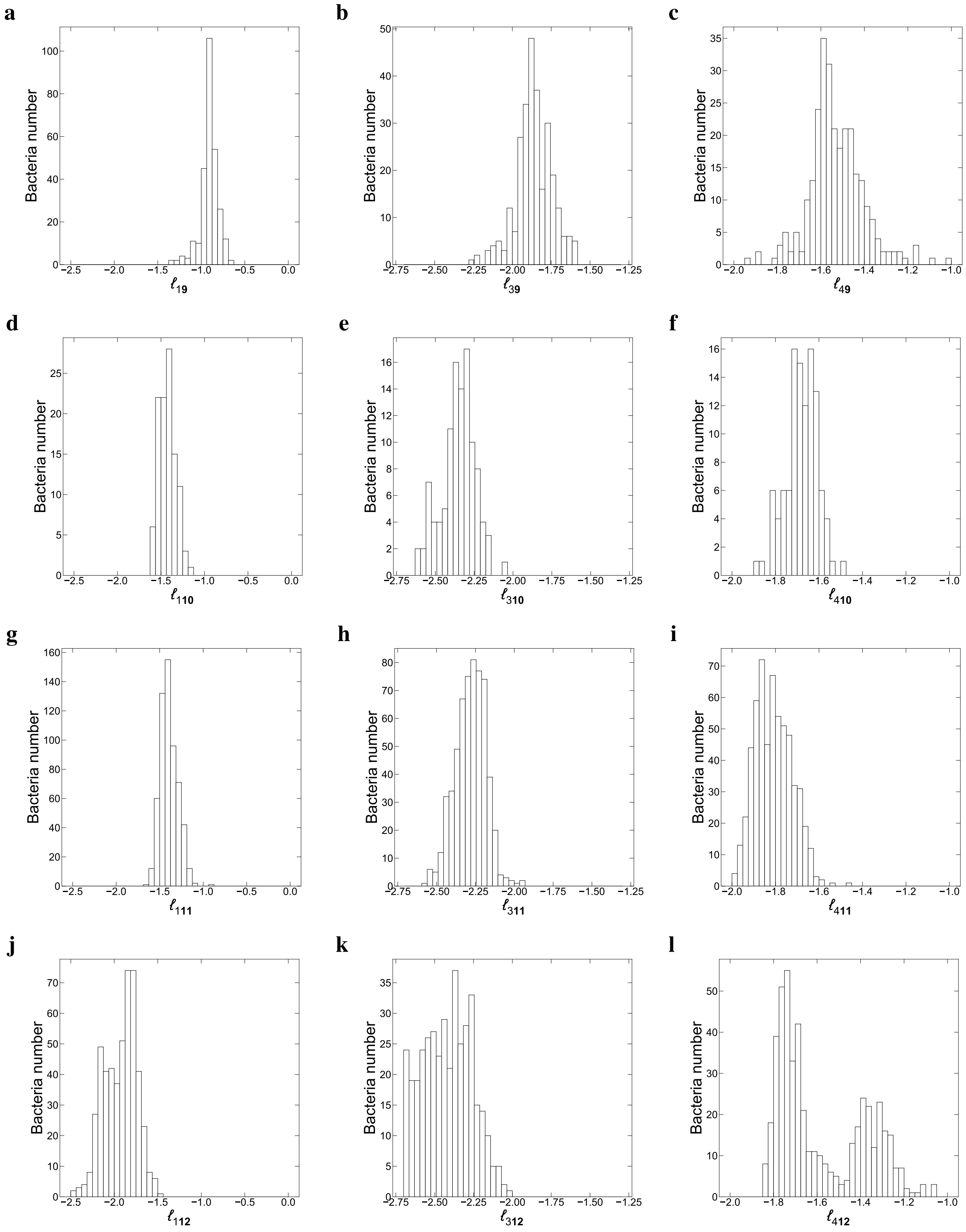

Supplementary Figure 44: Histograms of the decimal logarithmic of the characteristic times $l_{1,\mathbf{k}}$ (**a**, **d**, **g**, **j**), $l_{3,\mathbf{k}}$ (**b**, **e**, **h**, **k**), and $l_{4,\mathbf{k}}$ (**c**, **f**, **i**, **l**) for the RSFP-labeled bacteria **9**, **10**, **11**, and **12**.

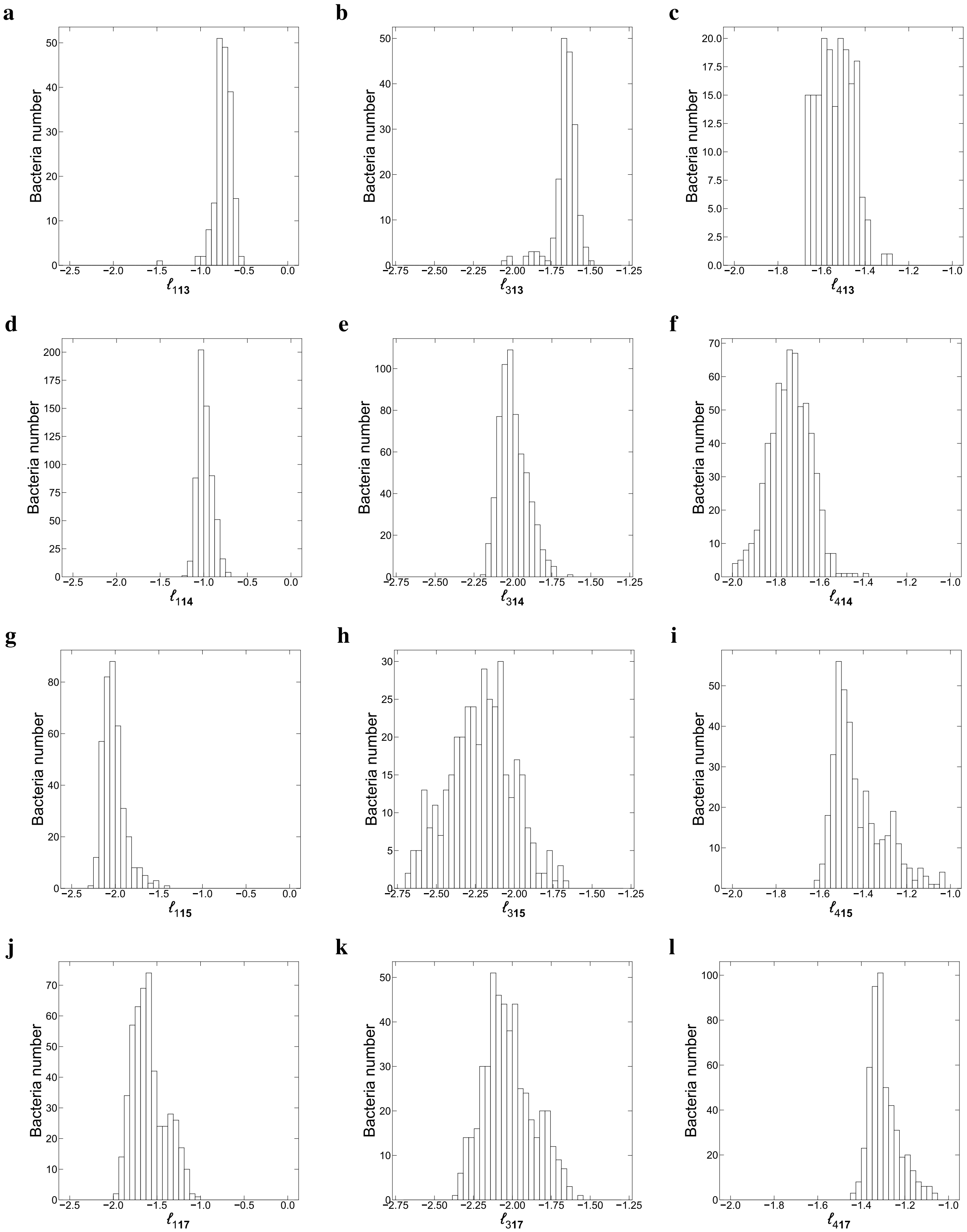

Supplementary Figure 45: Histograms of the decimal logarithmic of the characteristic times $l_{1,\mathbf{k}}$ (**a**, **d**, **g**, **j**), $l_{3,\mathbf{k}}$ (**b**, **e**, **h**, **k**), and $l_{4,\mathbf{k}}$ (**c**, **f**, **i**, **l**) for the RSFP-labeled bacteria **13**, **14**, **15**, and **17**.

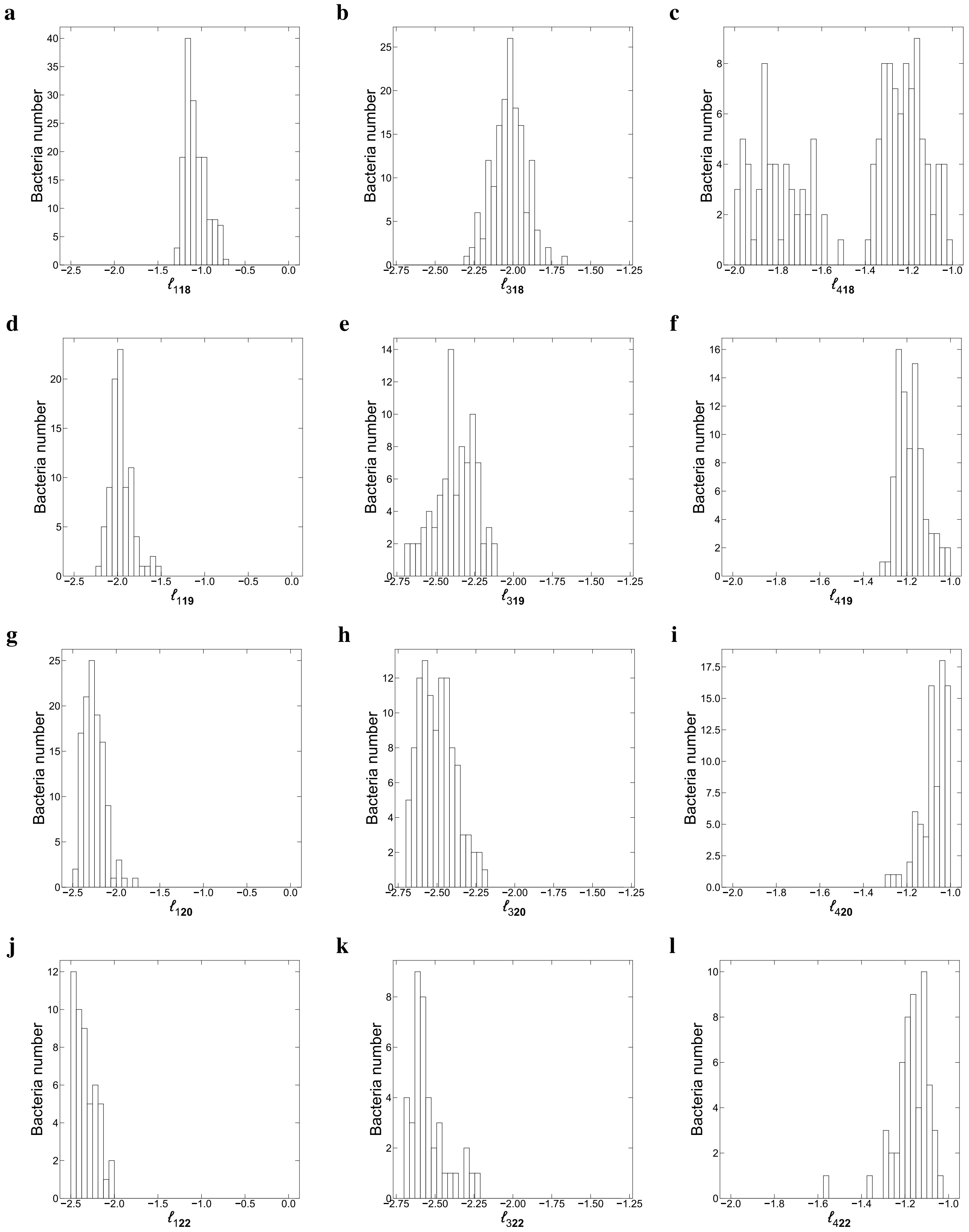

Supplementary Figure 46: Histograms of the decimal logarithmic of the characteristic times $l_{1,\mathbf{k}}$ (**a**, **d**, **g**, **j**), $l_{3,\mathbf{k}}$ (**b**, **e**, **h**, **k**), and $l_{4,\mathbf{k}}$ (**c**, **f**, **i**, **l**) for the RSFP-labeled bacteria **18**, **19**, **20**, and **22**.

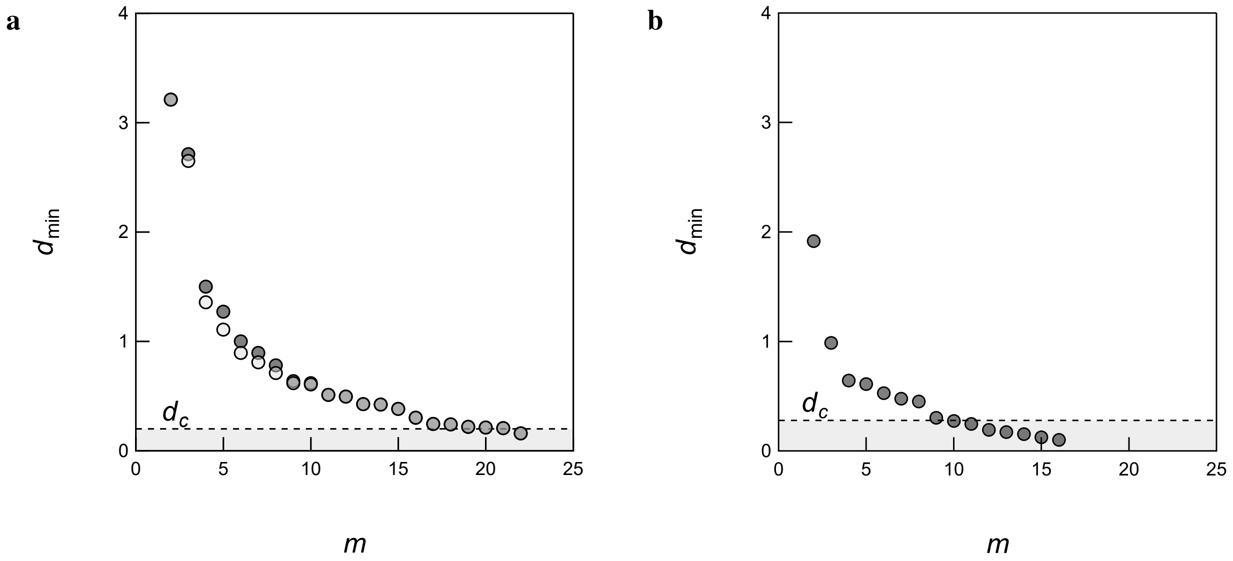

Supplementary Figure 47: *The LIGHTNING discriminatory power*. Minimum distance between pairs for optimized subsets of RSFPs versus cardinal m of the subsets. Deducing the threshold d_c from the experimental uncertainty on the determination of the characteristic times, the condition $d_{\text{min}} \geq d_c$ fixes the overall number of discriminatable representatives within a RSFP set: 20 among the 22 RSFPs **1**–**22** in RSFP solutions (**a**); 9 among the 16 RSFPs in the RSFP-labeled bacteria (**b**). In **a**, the disks and circles refer to the application of the non-inclusive and inclusive sorting algorithms respectively.

3 Supplementary Tables

3.1 Photophysical properties of the RSFPs

Supplementary Table 1: *Photophysical properties of the 22 investigated RSFPs as measured in this study.* λ_{max} and $\varepsilon(\lambda_{max})$ are the wavelength of maximal absorption and the molar absorption coefficient at λ_{max}, and λ_{em} and Φ_F are the wavelength of maximal emission and quantum yield of fluorescence. Solvent: pH=7.4 PBS (50 mM PBS, 150 mM NaCl).

RSFP	λ_{max} (nm)	$\varepsilon(\lambda_{max})$ ($M^{-1}.cm^{-1}$)	λ_{em} (nm)	Φ_F
**1**	503	80000	514	0.8
**2**	488	75000	512	0.2
**3**	487	55000	514	0.4
**4**	488	20000	513	–
**5**	505	40000	514	–
**6**	482	45000	514	0.4
**7**	496	35000	515	0.5
**8**	501	60000	514	0.7
**9**	499	95000	514	0.7
**10**	499	65000	514	0.7
**11**	500	85000	514	0.6
**12**	500	60000	513	0.6
**13**	503	105000	514	0.7
**14**	500	80000	514	0.6
**15**	486	50000	515	0.5
**16**	484	60000	515	0.2
**17**	483	50000	514	0.4
**18**	500	63000	515	0.5
**19**	479	55000	513	0.2
**20**	483	60000	515	0.3
**21**	484	50000	514	0.2
**22**	482	65000	515	0.2

3.2 Photochemical properties of the RSFPs acquired in RSFP solutions

Supplementary Table 2: *Results of the preliminary illumination experiments to evidence fast reversible fluorescence photoswitching vs slow light-driven long-term decay of fluorescence for the 22 investigated RSFPs.* $\Sigma_{j,fast}$ and $\Sigma_{j,slow}$ respectively designate the cross sections extracted from τ^{i}_{fast} and τ^{i}_{slow} using Eq.(5) as a fitting function to the fluorescence decays (the exponent $i = \mathrm{I}, \mathrm{II}$ refers to the two illuminations I and II) (see Eq.(43) for I and Eq.(52) for II). Solvent: pH=7.4 PBS (50 mM PBS, 150 mM NaCl).

RSFP	$\Sigma_{1,fast}$	$\Sigma_{1,slow}$	$\frac{\Sigma_{1,fast}}{\Sigma_{1,slow}}$	$\Sigma_{2,fast}$	$\Sigma_{2,slow}$	$\frac{\Sigma_{2,fast}}{\Sigma_{2,slow}}$
	(m^2/mol)	(m^2/mol)		(m^2/mol)	(m^2/mol)	
**1**	4.5 ± 0.7	6.10^{-4}	$(8.3 \pm 0.4)\times10^3$	315±10	$(6.3 \pm 0.1)\times10^{-4}$	$(2.2 \pm 0.4)\times10^4$
**2**	199 ± 10	7.10^{-3}	$(3.6 \pm 0.2)\times10^4$	431±20	$(7.3 \pm 0.2)\times10^{-3}$	$(7.1 \pm 0.2)\times10^4$
**3**	27 ± 3	2.10^{-3}	$(2.4 \pm 0.3)\times10^4$	326±30	$(2.2 \pm 0.1)\times10^{-3}$	$(1.4 \pm 0.3)\times10^4$
**4**	65 ± 2	6.10^{-3}	$(1.2 \pm 0.1)\times10^4$	240±15	$(7.6 \pm 0.1)\times10^{-3}$	$(1.5 \pm 0.1)\times10^3$
**5**	31 ± 3	5.10^{-4}	$(5.7 \pm 0.4)\times10^4$	75±8	$(6.4 \pm 0.3)\times10^{-4}$	$(2.4 \pm 0.2)\times10^3$
**6**	125 ± 12	9.10^{-4}	$(13.4 \pm 0.9)\times10^4$	700±20	$(1.5 \pm 0.1)\times10^{-3}$	$(3.2 \pm 0.1)\times10^4$
**7**	40 ± 5	3.10^{-3}	$(1.5 \pm 0.4)\times10^4$	680±30	$(3.4 \pm 0.2)\times10^{-3}$	$(5.3 \pm 0.1)\times10^3$
**8**	30 ± 8	4.10^{-4}	$(7.3 \pm 0.4)\times10^4$	560±20	$(4.2 \pm 0.3)\times10^{-4}$	$(1.2 \pm 0.5)\times10^4$
**9**	1.5 ± 0.3	2.10^{-4}	$(1.3 \pm 0.1)\times10^4$	490±30	$(2.2 \pm 0.4)\times10^{-4}$	$(1.8 \pm 0.7)\times10^4$
**10**	21 ± 2	2.10^{-4}	$(9.3 \pm 0.1)\times10^4$	425 ± 10	$(2.5 \pm 0.2)\times10^{-4}$	$(1.1 \pm 0.6)\times10^4$
**11**	20 ± 3	2.10^{-4}	$(12.6 \pm 0.8)\times10^4$	493±15	$(1.3 \pm 0.1)\times10^{-4}$	$(5.4 \pm 0.2)\times10^4$
**12**	45 ± 7	2.10^{-3}	$(2.2 \pm 0.4)\times10^4$	760±40	$(2.3 \pm 0.1)\times10^{-3}$	$(1.5 \pm 0.7)\times10^4$
**13**	7.3 ± 0.1	2.10^{-4}	$(3.1 \pm 0.2)\times10^4$	320±10	$(2.7 \pm 0.1)\times10^{-4}$	$(2.8 \pm 0.6)\times10^4$
**14**	5.7 ± 0.1	2.10^{-4}	$(2.8 \pm 0.3)\times10^4$	330±20	$(2.4 \pm 0.1)\times10^{-4}$	$(4.1 \pm 0.4)\times10^4$
**15**	97 ± 12	2.10^{-3}	$(4.3 \pm 0.2)\times10^4$	300±30	$(3.5 \pm 0.3)\times10^{-3}$	$(9.4 \pm 0.5)\times10^4$
**16**	110 ± 20	3.10^{-3}	$(4.6 \pm 0.3)\times10^4$	415±25	$(2.7 \pm 0.1)\times10^{-3}$	$(1.8 \pm 0.1)\times10^4$
**17**	200 ± 15	1.10^{-4}	$(10.1 \pm 0.8)\times10^4$	330±30	$(2.3 \pm 0.4)\times10^{-4}$	$(11.3 \pm 0.4)\times10^4$
**18**	5.4 ± 0.2	1.10^{-3}	$(4.2 \pm 0.4)\times10^4$	450±40	$(1.2 \pm 0.1)\times10^{-4}$	$(4.5 \pm 0.3)\times10^4$
**19**	190 ± 10	3.10^{-3}	$(7.2 \pm 0.6)\times10^4$	1400±250	$(2.6 \pm 0.2)\times10^{-3}$	$(1.4 \pm 0.2)\times10^4$
**20**	160 ± 21	3.10^{-3}	$(5.3 \pm 0.2)\times10^4$	920±20	$(3.7 \pm 0.1)\times10^{-3}$	$(7.6 \pm 0.3)\times10^3$
**21**	150 ± 8	7.10^{-3}	$(2.1 \pm 0.4)\times10^4$	1010±40	$(8.2 \pm 0.4)\times10^{-3}$	$(1.4 \pm 0.1)\times10^3$
**22**	200 ± 17	5.10^{-3}	$(4.1 \pm 0.3)\times10^4$	1250±30	$(6.3 \pm 0.2)\times10^{-3}$	$(1.4 \pm 0.2)\times10^4$

Supplementary Table 3: *Kinetic signatures of the 22 investigated RSFPs in the [1µs;1s] time windows.* Σ_1 and Σ_2 are the cross sections for reversible fluorescence photoswitching at $\lambda_1 = 488$ and $\lambda_2 = 405$ nm respectively. They have been extracted from the slopes of $1/\tau^{\mathrm{I}}$ versus I_1 and $1/\tau^{\mathrm{II}}$ versus I_2, respectively in the regimes of low light intensity. k_{21}^{Δ} is the rate constant for thermally-driven back isomerization of the photoswitched state. τ_{high}^{I} and $\tau_{high}^{\mathrm{II}}$ are the characteristic times respectively obtained at $I_1 = 200$ ein.m^{-2}.s^{-1} (illumination I$_{high}$) and $I_1 = 2$ ein.m^{-2}.s^{-1} and $I_2 = 90$ ein.m^{-2}.s^{-1} (illumination II$_{high}$). I_1^c and I_2^c are the cutoff light intensities at λ_1 and λ_2 at which the inverse of the characteristic times $1/\tau^{\mathrm{I}}$ and $1/\tau^{\mathrm{II}}$ depart from the two-states model predictions by 33% and 20%, respectively. Solvent: pH=7.4 PBS (50 mM PBS, 150 mM NaCl).

RSFP	Σ_1	Σ_2	$10^4 \times k_{21}^{\Delta}$	τ_{high}^{I}	$\tau_{high}^{\mathrm{II}}$	I_1^c	I_2^c
	(m^2/mol)	(m^2/mol)	(s^{-1})	(s)	(s)	(ein/m^2.s)	(ein/m^2.s)
**1**	5.1±0.5	350±20	5 ± 1	(4.52±0.09)×10^{-3}	(1.85±0.04)×10^{-4}	36 ± 2	4.3 ± 0.1
**2**	198±8	415±30	200 ± 50	(3.34±0.01)×10^{-4}	(7.73±0.02)×10^{-5}	8.2 ± 0.5	9.4 ± 0.2
**3**	30±5	350±18	1300 ± 300	(4.84±0.08)×10^{-3}	(1.25±0.03)×10^{-4}	4.6 ± 0.1	3.47 ± 0.05
**4**	70±10	230±22	0 ± 6	(2.56±0.07)×10^{-3}	(1.13±0.02)×10^{-2}	6.3 ± 0.5	1.23 ± 0.01
**5**	36±2	70±10	0 ± 8	(3.57±0.08)×10^{-3}	(9.25±0.02)×10^{-3}	35 ± 2	0.8 ± 0.1
**6**	112±9	670±45	6 ± 2	(1.17±0.05)×10^{-4}	(2.14±0.02)×10^{-4}	83 ± 15	3.1 ± 0.8
**7**	32±3	650±50	20 ± 10	(5.44±0.02)×10^{-4}	(6.74±0.03)×10^{-5}	36 ± 4	8.14 ± 0.02
**8**	33±2	515±30	6 ± 1	(4.23±0.03)×10^{-3}	(9.5±0.1)×10^{-5}	4.1 ± 0.2	4.2 ± 0.5
**9**	1.2 ±0.1	465±25	0.8 ± 0.2	(5.95±0.02)×10^{-3}	(1.25±0.02)×10^{-4}	2.2 ± 0.5	0.9 ± 0.1
**10**	25±4	420±30	10 ± 10	(6.8±0.1)×10^{-4}	(7.75±0.03)×10^{-5}	35 ± 6	0.27 ± 0.01
**11**	21±1	440±21	20 ± 10	(3.82±0.07)×10^{-3}	(1.12±0.01)×10^{-4}	6.3 ± 0.2	10 ± 4
**12**	50±6	730±43	4 ± 2	(3.1±0.2)×10^{-4}	(6.23±0.02)×10^{-5}	40 ± 5	3.12 ± 0.02
**13**	7.4±0.2	300±12	3 ± 1	(5.63±0.02)×10^{-3}	(1.75±0.03)×10^{-4}	35 ± 6	1.18 ± 0.03
**14**	6.1±0.3	300±31	20 ± 10	(2.92±0.05)×10^{-3}	(1.84±0.02)×10^{-4}	20 ± 3	8.42 ± 0.05
**15**	115±6	310±28	20 ± 10	(3.14±0.02)×10^{-4}	(3.64±0.03)×10^{-4}	7.1 ± 0.3	0.45 ± 0.07
**16**	120±11	400±37	20 ± 10	(6.25±0.04)×10^{-4}	(9.83±0.02)×10^{-5}	6.3 ± 0.1	3.45 ± 0.08
**17**	212±6	320±10	30 ± 10	(4.26±0.03)×10^{-4}	(1.27±0.02)×10^{-4}	3.4 ± 0.1	2.15 ± 0.07
**18**	5.1±0.3	483±24	0 ± 3	(3.82±0.01)×10^{-3}	(3.82±0.06)×10^{-4}	41 ± 3	1.32 ± 0.04
**19**	212±8	1670±120	–†	(1.21±0.06)×10^{-4}	(3.15±0.01)×10^{-5}	25 ± 5	1.15 ± 0.02
**20**	170±10	900±85	–†	(1.34±0.07)×10^{-4}	(1.35±0.04)×10^{-4}	5±2	0.83 ± 0.01
**21**	144±35	930±66	1.1 ± 0.3	(2.15±0.03)×10^{-4}	(1.73±0.01)×10^{-4}	2.5 ± 0.1	3.4 ± 0.2
**22**	212±10	1260±100	–†	(1.36±0.08)×10^{-4}	(6.17±0.02)×10^{-5}	1.7 ± 0.2	2.5 ± 0.4

† Too slow to be reliably measured.

A LIGHTNING data collection

In this section, we address several issues related to LIGHTNING acquisition. We first examine the general response of an RSF when it is submitted to a change of constant illumination. We subsequently report on the design of the measurement cell of our instrumental setup (the photoswitchometer). Eventually, we discuss the optimization of LIGHTNING acquisition.

A.1 Theoretical analysis of the time evolution of the RSF fluorescence signal upon constant illumination

In this subsection, we analyze the response of an RSF when it is submitted to a change of constant illumination. We consider an RSF engaged in a photocycle involving n_s states $\mathrm{C_i}$ and first-order reactions

$$\mathrm{C_i} \underset{k_{ji}}{\overset{k_{ij}}{\rightleftharpoons}} \mathrm{C_j} \tag{22}$$

where $i = 1, ..., n_s$, $j = 1, ..., n_s$, and $i \neq j$. The rate constants k_{ij} are associated with thermal or photochemical steps. The dynamics of the system is governed by the system of equations

$$\frac{\mathrm{d}C_1}{\mathrm{d}t} = -\left(k_{12} + k_{13} + ... + k_{1n_s}\right) C_1 + k_{21}C_2 + k_{31}C_3 + ... + k_{n_s1}C_n \tag{23}$$

$$\frac{\mathrm{d}C_2}{\mathrm{d}t} = k_{12}C_1 - \left(k_{21} + k_{23} + ... + k_{2n_s}\right) C_2 + k_{32}C_3 + ... + k_{n_s2}C_n \tag{24}$$

$$... \tag{25}$$

$$\frac{\mathrm{d}C_n}{\mathrm{d}t} = k_{1n_s}C_1 + k_{2n_s}C_2 + k_{3n_s}C_3 + ... - \left(k_{n_s1} + k_{n_s2} + ... + k_{n_s(n_s-1)}\right) C_n \tag{26}$$

which yields the conservation relation $\Sigma_{i=1}^{n_s} C_i(t) = \Sigma_{i=1}^{n_s} C_i^0$ where C_i^0 are the initial concentrations.

The linear system given in Eqs.(23–26) can be written in matrix form

$$\frac{\mathrm{d}\mathbf{C}}{\mathrm{d}t} = \mathbf{KC} \tag{27}$$

with the vector **C** and the matrix **K**

$$\mathbf{C} = \begin{pmatrix} C_1 \\ C_2 \\ . \\ . \\ C_n \end{pmatrix}, \tag{28}$$

$$\mathbf{K} = \begin{pmatrix} -\left(k_{12} + k_{13} + ... + k_{1n_s}\right) & k_{21} & k_{31} & ... & k_{n_s1} \\ k_{12} & -\left(k_{21} + k_{23} + ... + k_{2n_s}\right) & k_{32} & ... & k_{n_s2} \\ . & . & . & ... & . \\ . & . & . & ... & . \\ . & . & . & ... & . \\ k_{1n_s} & k_{2n_s} & k_{3n_s} & ... & -\left(k_{n_s1} + k_{n_s2} + ... + k_{n_s(n_s-1)}\right) \end{pmatrix} \tag{29}$$

The matrix **K** has n_s eigenvalues, one of which is zero.[13] Considering the case where the eigenvalues are real, negative and denoted by $-1/\tau_i$, they are ordered according to $0 < \tau_1 < \tau_2 < ... < \tau_{n_s-1}$. The Gerschgorin's circle theorem has been used to show that the eigenvalues are bounded and obey[13]

$$0 \leq \frac{1}{\tau_i} \leq 2 \max_i (\Sigma_{j=1, j\neq i}^{n_s} k_{ij}). \tag{30}$$

Eq.(27) is straightforwardly solved in the eigenvector basis. By using the change-of-basis matrix and the initial conditions, we find that the concentrations C_i are sums of exponential terms involving the eigenvalues

$$C_i = \Sigma_{j=1}^{n_s-1} r_{ij} \exp\left(-\frac{t}{\tau_j}\right) + s_{\rm i}. \tag{31}$$

where the preexponential factors r_{ij} and the constant $s_{\rm i}$ depend on the rate constants and the initial conditions.

The RSF fluorescence signal $I_F(t)$ originates from summing the individual contributions of the n_s RSF states associated with brightness Q_i and concentrations C_i

$$I_F(t) = \Sigma_{i=1}^{n_s} Q_i I C_i = \Sigma_{j=1}^{n_s-1} U_j \exp\left(-\frac{t}{\tau_j}\right) + W \tag{32}$$

with $U_j = \Sigma_{i=1}^{n_s} Q_i I r_{ij}$ and $W = \Sigma_{i=1}^{n_s} Q_i I s_{\rm i}$ for a light excitation of intensity I. As shown in Eq.(32), the RSF fluorescence signal exhibits a multiexponential time dependence.

A.2 Design of the measurement cell of the photoswitchometer for liquid solutions

Our home-built photoswichometer was designed to illuminate the samples with light intensities covering five orders of magnitude (from 0.5 W.cm^{-2} to 50 kW.cm^{-2}). Whereas for low light intensities we could rely on LED delivering homogeneous illumination in a large volume, we had to focus laser beams to reach the higher light intensities. In the case of a small illuminated cell, the fluorescence evolution may adopt a non-trivial decay due to the diffusion of the fluorophore outside the illuminated cell. The design of the photoswitchometer is compatible with the acquisition of the photoswitching properties of RSFs in solid samples. However, it was interesting to make it as well relevant for measurements in solution. Indeed diffusion can refresh a solution between successive experiments which avoids long-term extinction. Fluorescence evolution including the effect of diffusion could be theoretically treated to extract the photochemical information sought for. Nevertheless this computation would not be easily prone to data processing and would slow down the automatic acquisition of the photochemical information in the widest range of illumination intensities. Hence we designed experimental conditions in which the contribution of diffusion could be neglected. In this subsection, we first derive the expressions of the fluorescence evolution in a two-state system, which is relevant of the regimes of low light intensity (see section C). Then we make precise geometrical constraints on the measurement cell of the photoswitchometer for which diffusion may be neglected.

A.2.1 Fluorescence evolution in a homogeneous system

We adopt the two-state exchange

$$\mathrm{C}_1 \underset{k_{21}^{\rm i}}{\overset{k_{12}^{\rm i}}{\rightleftharpoons}} \mathrm{C}_2 \tag{33}$$

where the state C_1 is photochemically converted into the state C_2 at rate constant $k_{12}^{\rm i}$ from which it can relax back to the state C_1 at the rate constant $k_{21}^{\rm i}$ including photochemical and thermally-driven contributions. The exponent $i = \mathrm{I}, \mathrm{II}, \mathrm{III}$ refers to the illuminations I, II, and III. We further introduce $K_{12}^{\rm i} = k_{12}^{\rm i}/k_{21}^{\rm i}$.

We first assume that the system is homogeneous at any time of its evolution. The evolution of the concentrations $C_1^{\rm i}$ and $C_2^{\rm i}$ of the two species C_1 and C_2 for each illumination i is then given by

$$\frac{\mathrm{d}C_1^{\rm i}}{\mathrm{d}t} = -k_{12}^{\rm i} C_1^{\rm i} + k_{21}^{\rm i} C_2^{\rm i} \tag{34}$$

with the conservation relation $C_1^{\rm i} + C_2^{\rm i} = C_{\rm tot}$, where $C_{\rm tot}$ is the total concentration.

Illumination I For illumination I, under light intensity I_1 at the wavelength λ_1, the initial condition is $C_1^{\mathrm{I}} = C_{\mathrm{tot}}$. The concentrations C_1^{I} and C_2^{I} obey

$$C_2^{\mathrm{I}} - C_2^{\mathrm{I},\infty} = C_1^{\mathrm{I},\infty} - C_1^{\mathrm{I}} = -C_2^{\mathrm{I},\infty} \exp\left(-\frac{t}{\tau^{\mathrm{I}}}\right) \tag{35}$$

where

$$C_1^{\mathrm{I},\infty} = \frac{1}{1+K_{12}^{\mathrm{I}}} C_{\mathrm{tot}} \tag{36}$$

$$C_2^{\mathrm{I},\infty} = \frac{K_{12}^{\mathrm{I}}}{1+K_{12}^{\mathrm{I}}} C_{\mathrm{tot}} \tag{37}$$

are the steady concentrations reached at large time and

$$\tau^{\mathrm{I}} = \frac{1}{k_{12}^{\mathrm{I}} + k_{21}^{\mathrm{I}}} \tag{38}$$

designates the relaxation time of the photochemical conversion of the two species C_1 and C_2. The fluorescence emission results from the individual contributions of the species C_1 and C_2. For the sake of simplicity, we assume that only one species, supposed to be C_1, has a non-negligible brightness Q. The results can be straightforwardly extended to the general case. The fluorescence intensity can be written

$$I_F^{\mathrm{I}} = Q C_1^{\mathrm{I}} I_1 \tag{39}$$

Using Eqs.(35-37), we find

$$\frac{I_F^{\mathrm{I}}}{I_F^{\mathrm{I},0}} = 1 + \frac{Q C_{\mathrm{tot}} I_1}{I_F^{\mathrm{I},0}} \frac{K_{12}^{\mathrm{I}}}{1+K_{12}^{\mathrm{I}}} \left[\exp\left(-\frac{t}{\tau^{\mathrm{I}}}\right) - 1\right] \tag{40}$$

where $I_F^{\mathrm{I},0}$ is the initial fluorescence intensity.

In the case of RSFPs and at low light intensity I_1 the two-state model (33) satisfactorily accounts for the observed fluorescence photoswitching. Denoting by $\sigma_{12,1}$ (resp. $\sigma_{21,1}$) the molecular cross section for the photoisomerization $C_1 \rightarrow C_2$ (resp. $C_2 \rightarrow C_1$) driven at wavelength λ_1 and by k_{21}^{Δ} the thermal part of the rate constant for the backward step $C_2 \rightarrow C_1$, we have

$$k_{12}^{\mathrm{I}} = \sigma_{12,1} I_1 \tag{41}$$

$$k_{21}^{\mathrm{I}} = \sigma_{21,1} I_1 + k_{21}^{\Delta} \tag{42}$$

The relaxation time given in Eq.(38) is rewritten

$$\tau^{\mathrm{I}} = \frac{1}{\Sigma_1 I_1 + k_{21}^{\Delta}} \tag{43}$$

where $\Sigma_1 = \sigma_{12,1} + \sigma_{21,1}$ is the sum of cross sections at the wavelength λ_1. Eq.(43) shows that $1/\tau^{\mathrm{I}}$ exhibits a linear dependence on the light intensity I_1.

Illumination II For illumination II, under light intensities I_1 at the wavelength λ_1 and I_2 at the wavelength λ_2, the initial condition is the steady state reached at the end of illumination I, $C_1^{\mathrm{II}} = C_1^{\mathrm{I},\infty}$. The concentrations C_1^{II} and C_2^{II} obey

$$C_2^{\mathrm{II}} - C_2^{\mathrm{II},\infty} = C_1^{\mathrm{II},\infty} - C_1^{\mathrm{II}} = \left(C_2^{\mathrm{I},\infty} - C_2^{\mathrm{II},\infty}\right) \exp\left(-\frac{t}{\tau^{\mathrm{II}}}\right) \tag{44}$$

where

$$C_1^{\mathrm{II},\infty} = \frac{1}{1 + K_{12}^{\mathrm{II}}} C_{\mathrm{tot}} \tag{45}$$

$$C_2^{\mathrm{II},\infty} = \frac{K_{12}^{\mathrm{II}}}{1 + K_{12}^{\mathrm{II}}} C_{\mathrm{tot}} \tag{46}$$

are the steady concentrations reached at large time and

$$\tau^{\mathrm{II}} = \frac{1}{k_{12}^{\mathrm{II}} + k_{21}^{\mathrm{II}}} \tag{47}$$

designates the relaxation time of the photochemical conversion of the two species C_1 and C_2. The brightness at the wavelength λ_2 is assumed to be much smaller than the one at the wavelength λ_1.[7] Using the same assumptions as the ones made for illumination I, we obtain the fluorescence intensity

$$I_F^{\mathrm{II}} = Q C_1^{\mathrm{II}} I_1 \tag{48}$$

Using Eqs.(44-46), we find

$$\frac{I_F^{\mathrm{II}}}{I_F^{\mathrm{II},0}} = 1 + \frac{Q C_{\mathrm{tot}} I_1}{I_F^{\mathrm{II},0}} \left(\frac{K_{12}^{\mathrm{II}}}{1 + K_{12}^{\mathrm{II}}} - \frac{K_{12}^{\mathrm{I}}}{1 + K_{12}^{\mathrm{I}}}\right) \left[\exp\left(-\frac{t}{\tau^{\mathrm{II}}}\right) - 1\right] \tag{49}$$

where $I_F^{\mathrm{II},0}$ is the initial fluorescence intensity.

For RSFPs at low light intensities I_1 and I_2, the two-state model (33) can be used to account for the observed fluorescence photoswitching. Introducing $\sigma_{12,2}$ and $\sigma_{21,2}$ as the molecular cross sections for photoisomerization steps driven at wavelength λ_2, one has

$$k_{12}^{\mathrm{II}} = \sigma_{12,1} I_1 + \sigma_{12,2} I_2 \tag{50}$$

$$k_{21}^{\mathrm{II}} = \sigma_{21,1} I_1 + \sigma_{21,2} I_2 + k_{21}^{\Delta} \tag{51}$$

The relaxation time given in Eq.(47) is rewritten

$$\tau^{\mathrm{II}} = \frac{1}{\Sigma_1 I_1 + \Sigma_2 I_2 + k_{21}^{\Delta}} \tag{52}$$

where $\Sigma_2 = \sigma_{12,2} + \sigma_{21,2}$ is the sum of cross sections at the wavelength λ_2. Eq.(52) shows that $1/\tau^{\mathrm{II}}$ exhibits a linear dependence on the light intensity I_2.

Illumination III For the illumination III which has been used to measure the rate constant associated to thermal relaxation of the photoswitched RSFPs, the initial condition is the steady state reached at the end of illumination I, $C_1^{\mathrm{III}} = C_1^{\mathrm{I},\infty}$. The concentrations C_1^{III} and C_2^{III} obey

$$C_2^{\mathrm{III}} - C_2^{\mathrm{III},\infty} = C_1^{\mathrm{III},\infty} - C_1^{\mathrm{III}} = \left(C_2^{\mathrm{I},\infty} - C_2^{\mathrm{III},\infty}\right) \exp\left(-\frac{t}{\tau^{\mathrm{III}}}\right) \tag{53}$$

where

$$C_1^{\mathrm{III},\infty} = \frac{1}{1+K_{12}^{\mathrm{III}}} C_{\mathrm{tot}} \quad (54)$$

$$C_2^{\mathrm{III},\infty} = \frac{K_{12}^{\mathrm{III}}}{1+K_{12}^{\mathrm{III}}} C_{\mathrm{tot}} \quad (55)$$

are the steady concentrations reached at large time and

$$\tau^{\mathrm{III}} = \frac{1}{k_{12}^{\mathrm{III}} + k_{21}^{\mathrm{III}}} \quad (56)$$

designates the relaxation time of the photochemical conversion of the two species C_1 and C_2. Using the same assumptions as the ones made for illumination I, we obtain the fluorescence intensity

$$I_F^{\mathrm{III}} = Q C_1^{\mathrm{III}} I_1 \quad (57)$$

Using Eqs.(53–55), we find

$$\frac{I_F^{\mathrm{III}}}{I_F^{\mathrm{III},0}} = 1 + \frac{Q C_{\mathrm{tot}} I_1}{I_F^{\mathrm{III},0}} \left(\frac{K_{12}^{\mathrm{III}}}{1+K_{12}^{\mathrm{III}}} - \frac{K_{12}^{\mathrm{I}}}{1+K_{12}^{\mathrm{I}}} \right) \left[\exp\left(-\frac{t}{\tau^{\mathrm{III}}} \right) - 1 \right] \quad (58)$$

where $I_F^{\mathrm{III},0}$ is the initial fluorescence intensity.

In the presence of the weak light at the wavelength λ_1 required to read out the RSFP signal after variable delays without any light, one has

$$k_{12}^{\mathrm{III}} \simeq 0 \quad (59)$$

$$k_{21}^{\mathrm{III}} \simeq k_{21}^{\Delta} \quad (60)$$

The relaxation time given in Eq.(56) is rewritten

$$\tau^{\mathrm{III}} \simeq \frac{1}{k_{21}^{\Delta}}. \quad (61)$$

A.2.2 Impact of diffusion

We then release the assumption of system homogeneity during its evolution under illumination. Without loss of generality, we restrict the analysis of diffusion effect to illumination i=I. The rate constants in Eq.(33) are written k_{12} and k_{21}. We establish below the evolution of the concentrations of C_1 and C_2 in two different geometries of the illuminated measuring cell. In the first case, we apply a 3D-model in which illumination is restricted to a sphere. In the second situation, we apply a 2D-model in which illumination occurs within a cylinder. The most favorable geometry of the illuminated cell of the photoswitchometer is then made precise.

Spherical illumination In the first model, we assume that illumination is homogeneous within a sphere of radius r_0 and vanishes out of the illuminated sphere within the whole solution envisioned as a spherical reservoir of radius R containing the species C_1 at concentration C_{tot}. We adopt $r_0 = 0.3$ μm and $R = 2.2$ μm which are experimentally relevant. As expected for the investigated fluorescent proteins, the species C_1 and C_2 are supposed to diffuse in the reactor with the same diffusion coefficient D so that their concentrations obey the conservation relation $C_1 + C_2 = C_{\mathrm{tot}}$.

We deal with a reaction-diffusion system with spherical symmetry. In the illuminated cell, the dynamics is governed by

$$\frac{\partial C_1}{\partial t} = -(k_{12}+k_{21})C_1 + k_{21}C_{\mathrm{tot}} + D\left[\frac{\partial^2 C_1}{\partial r^2} + \frac{2}{r}\frac{\partial C_1}{\partial r}\right] \tag{62}$$

where r is the radial coordinate. The change of variable

$$u = r[C_1 - C_1^{h,\infty}], \tag{63}$$

where $C_1^{h,\infty} = \frac{k_{21}C_{\mathrm{tot}}}{k_{12}+k_{21}}$ is the steady state obtained upon homogeneous illumination, leads to[14]

$$\frac{\partial u}{\partial t} = -(k_{12}+k_{21})u + D\frac{\partial^2 u}{\partial r^2} \tag{64}$$

for which the boundary condition $u(r = 0, t) = 0$ holds for all time t. The initial condition is $u(r, t = 0) = r(C_{\mathrm{tot}} - C_1^{h,\infty})$. An analogous equation is used outside the illuminated cell where $k_{12} = 0$. Our goal is to determine the range of rate constants in which diffusion can be neglected.

In the absence of diffusion, the dynamics of the system is given by

$$\frac{\mathrm{d}C_1^h}{\mathrm{d}t} = -(k_{12}+k_{21})C_1^h + k_{21}C_{\mathrm{tot}} \tag{65}$$

leading to the exponential decay

$$C_1^h = \frac{C_{\mathrm{tot}}}{k_{12}+k_{21}}\left[k_{12}\exp(-(k_{12}+k_{21})t) + k_{21}\right] \tag{66}$$

We define the time τ^∞ at which the concentration of species C_1 reaches the stationary value $C_1^{h,\infty}$ to within 1 percent in the absence of diffusion. Following Eq.(66), we find

$$\tau^\infty = \frac{-1}{k_{12}+k_{21}}\ln\left(\frac{0.01k_{21}}{k_{12}}\right) \tag{67}$$

where we assume $k_{21} \leq k_{12}$. Eq.(64) inside the illuminated cell and the analogous equation with $k_{12} = 0$ outside the illuminated cell are discretized and numerically solved until time reaches τ^∞. We investigated the following parameter range: 1 $\mathrm{s}^{-1} \leq k_{12} \leq 10^7$ s^{-1}, 1 $\mathrm{s}^{-1} \leq k_{21} \leq 10^5$ s^{-1} with $k_{21} \leq k_{12}$, 30 $\mu\mathrm{m}^2\mathrm{s}^{-1} \leq D \leq 300$ $\mu\mathrm{m}^2\mathrm{s}^{-1}$, which is consistent with RSFs properties.

The stationary spatial profile $C_1(r, \tau^\infty)$ is compared to the prediction $C_1^{h,\infty}$ in Supplementary Figure 48 for different values of k_{12}. In the spherical geometry and for such parameter values, diffusion induces an important bias, which decreases without disappearing as the rate constant k_{12} increases in the investigated range.

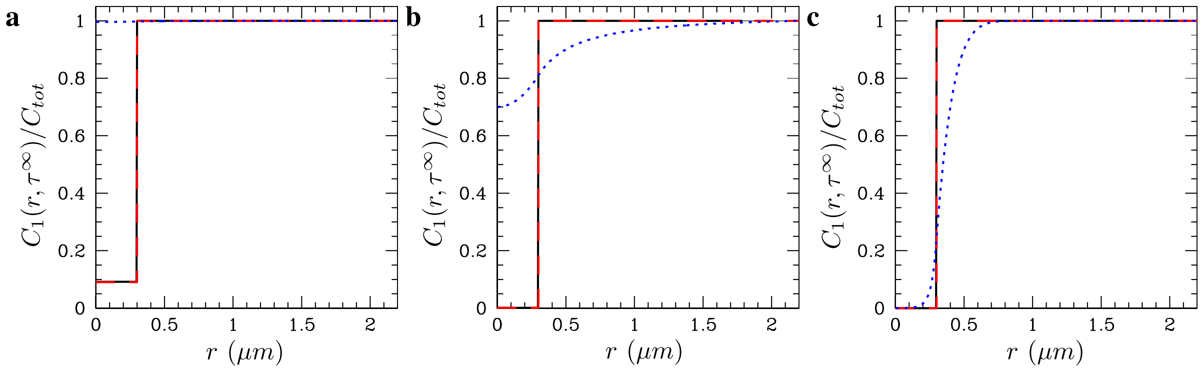

Supplementary Figure 48: Scaled stationary spatial profiles $C_1(r, \tau^\infty)/C_{\rm tot}$ for species C_1 deduced from the numerical integration of Eq.(64) in the spherical geometry (blue dotted line), the numerical integration of Eq.(71) in the cylindrical geometry (black dashed line), and the prediction $C_1^{h,\infty}$ (red dashed line) for $r_0 = 0.3$ μm, $R = 2.2$ μm, $k_{21} = 1$ s^{-1}, $D = 100$ μm^2 s^{-1} with k_{12} (s^{-1})= 10 (**a**), 10^3 (**b**), 10^5 (**c**).

Without loss of generality, we assume that only species C_1 has a non vanishing brightness Q. The fluorescence signal $I_F = QI_1I'_F$ is proportional to the integral of the concentration C_1 over the illuminated volume

$$I'_F = \int_0^{r_0} C_1 4\pi r^2 \mathrm{d}r \quad (68)$$

where $C_1 = C_{\rm tot} - C_2$ is deduced from Eqs.(63) and (64).

The evolution of fluorescence I_F is compared to the prediction without diffusion, $C_1^h V$, where C_1^h is given in Eq.(66) and $V = \frac{4}{3}\pi r_0^3$ is the volume of the illuminated cell. Supplementary Figure 49 displays the fluorescence evolution for different values of the rate constant k_{12}. The difference between the results in the presence and absence of diffusion markedly depends on k_{12}. As already mentioned, the deviation is reduced as k_{12} increases.

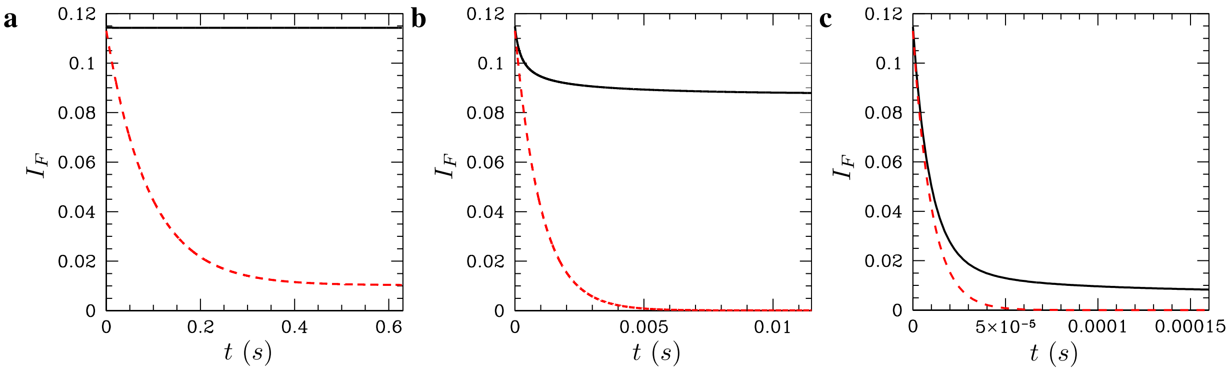

Supplementary Figure 49: Spherical geometry: Fluorescence evolution I_F for species C_1 deduced from the numerical integration of Eq.(64) and integration over the volume $V = \frac{4}{3}\pi r_0^3$ of the illuminated cell (black solid line) and from exponential decay $C_1^h V$ predicted by Eq.(66) upon homogeneous illumination (red dashed line) for the parameter values given in the caption of Supplementary Figure 48 and $Q = I_1 = 1$.

If the signal-to-noise ratio remains sufficient when integrating the concentration C_1 over a sphere of radius $r_0/2$ instead of the radius r_0 of the illuminated zone, the results are improved for $k_{12} = 10^5$ s^{-1} as shown in Supplementary Figure 50. The same scale was chosen in Supplementary Figure 49c and Supplementary Figure 50 to better highlight the loss of signal strength due to the integration of the concentration over a smaller volume. Nevertheless, this strategy

is useless for $k_{12} \leq 10^3$ s^{-1}, since the concentration at $r_0/2$ is extremely far from the prediction without diffusion, as shown in Supplementary Figure 48a and Supplementary Figure 48b.

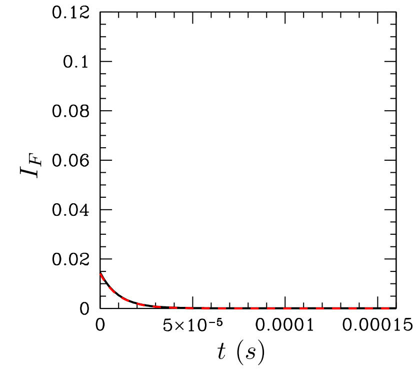

Supplementary Figure 50: Spherical geometry: Same caption as for Supplementary Figure 49c ($k_{12} = 10^5$ s^{-1}) for the fluorescence evolution obtained by integration of the concentration C_1 over a sphere of radius $r_0/2$ instead of r_0.

A more quantitative evaluation of the discrepancy between the results with and without diffusion is given in Supplementary Figure 51 for variable rate constants and two values of the diffusion coefficient. The difference between the signal and the exponentially-decaying solution obtained upon homogeneous illumination is computed according to:

$$J = \frac{\int_{t=0}^{\tau^\infty} (I'_F - C_1^h V) dt}{(C_{\text{tot}} - C_1^{h,\infty}) V \tau^\infty} \tag{69}$$

where $C_1^h(t)$ is given in Eq.(66). The deviation J tends to 1 when the rate constant k_{12} is small compared to the typical frequency $\frac{D}{r_0^2}$ and vanishes for sufficiently large values of k_{12}. As expected, J is smaller and the results are improved for a smaller value of the diffusion coefficient. Interestingly, J does not sensitively depend on the rate constant k_{21}.

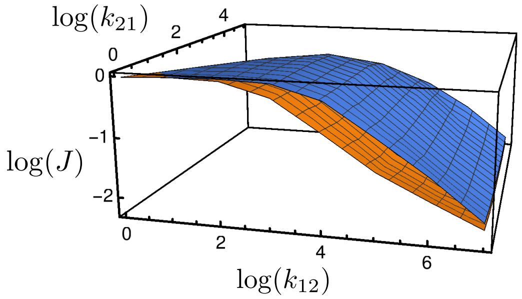

Supplementary Figure 51: Spherical geometry: Decimal logarithm $\log(J)$ of the deviation between the results with and without diffusion versus $\log(k_{12})$ and $\log(k_{21})$ for $D = 300$ μm^2 s^{-1} (blue) and $D = 30$ μm^2 s^{-1} (orange).

According to Supplementary Figure 51, the determination of the relaxation time using the exponential decay without diffusion is valid to within a few percent in a spherical reactor for $r_0 = 0.3$ μm and $D \simeq 100$ μm^2 s^{-1} if the rate constant k_{12} of the photochemical step is larger than or equal to 10^7 s^{-1}, which is very restrictive. More generally, we introduce the cutoff rate constant

$$k_{12}^c \simeq 10^4 \frac{D}{r_0^2} \tag{70}$$

where D is the diffusion coefficient of the different species and r_0, the typical length of the illuminated cell. Diffusion is negligible to within a few percent in the spherical geometry provided that $k_{12} \geq k_{12}^c$ with k_{12}^c given in Eq.(70). Even with a larger cell of radius $r_0 = 10$ μm, the condition would become $k_{12} \geq 10^4$ s^{-1}, which remains constraining.

Cylindrical illumination In the second model, we assume that illumination is homogeneous within a cylinder of radius r_0 and height h and vanishes out of the illuminated zone within the whole solution envisioned as a cylindrical reservoir of radius R containing the species C_1 at concentration $C_{\rm tot}$. With respect to the experimental conditions, we adopt $h = 80$ μm, $r_0 = 0.3$ μm, and $R = 2.2$ μm in order to establish the comparison with the results derived in the spherical geometry.

In the illuminated cell, the dynamics is governed by

$$\frac{\partial C_1}{\partial t} = -(k_{12} + k_{21})C_1 + k_{21}C_{\rm tot} + D\left[\frac{\partial^2 C_1}{\partial r^2} + \frac{1}{r}\frac{\partial C_1}{\partial r}\right] \quad (71)$$

The same equation with $k_{12} = 0$ holds outside the illuminated cell. We introduce the same change of variable $u(r,t) = r(C_1 - C_1^{h,\infty})$ as in the case of spherical symmetry, because the boundary condition $u(r = 0, t) = 0$ is convenient and makes it possible to solve 1D equations. The equations for u inside and outside the illuminated zone are solved in an infinite cylinder in contact with a reservoir of species C_1. The stationary profiles obtained in the cylindrical reactor are given in Supplementary Figure 52 and superimposed on the predictions without diffusion. The results perfectly agree with the homogeneously illuminated solution $C_1^{h,\infty}$, even for the small rate constant value $k_{12} = 10$ s^{-1}. It is to be noted that the results are obtained for an infinite cylindrical reactor which certainly overestimates their quality. However, the predictions remain valid for a cylinder of height h about 10 times larger than the radius r_0. This condition is fully obeyed for the considered parameters leading to $h/r_0 \simeq 270$.

The fluorescence evolution $I_F = QI_1I_F'$ given in Supplementary Figure 52 for $k_{12} = 10$ s^{-1} results from integrating the concentration C_1 over a cylinder of radius r_0 and height h according to

$$I_F' = h\int_0^{r_0} C_1 2\pi r \mathrm{d}r \quad (72)$$

Due to the larger volume of the cylinder compared to the sphere of the same radius, the fluorescence evolution in Supplementary Figure 52 begins with a larger amplitude than the fluorescence evolution in Supplementary Figure 49. In Supplementary Figure 52, the fluorescence series I_F taking into account or not diffusion are superimposed even for the small value $k_{12} = 10$ s^{-1} of the rate constant and the small radius $r_0 = 0.3$ μm of the illuminated cell.

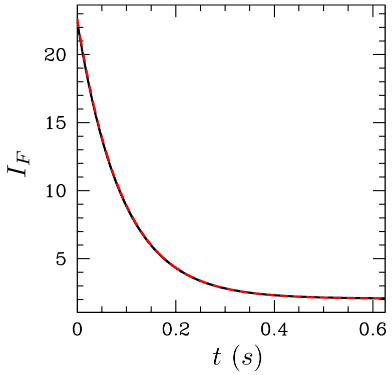

Supplementary Figure 52: Cylindrical geometry: Evolution of the signal S for species C_1 deduced from the numerical integration of Eq.(71) and integration over the volume $V = \pi(r_0)^2h$ of the illuminated zone (blue solid line) and exponential decay C_1^hV predicted by Eq.(66) upon homogeneous illumination (red dashed line) for $k_{12} = 10$ s^{-1}, $h = 80$ μm, and $Q = I_1 = 1$. The values of the other parameters are given in the caption of Supplementary Figure 48.

The difference J between the fluorescence series with and without diffusion defined in Eq.(69) is equal to 1.2% for $k_{12} = 10^3$ s^{-1}, $r_0 = 0.3$ μm, and $D = 100$ μm^2 s^{-1}. Hence, in the cylindrical geometry, we find that the cutoff

rate constant obeys

$$k_{12}^c \simeq \frac{D}{r_0^2} \tag{73}$$

Diffusion is negligible inside the illuminated cell for $k_{12} \geq k_{12}^c$ and whatever k_{21}. Comparing Eqs.(70) and (73), we conclude that the cylindrical geometry, with an illuminated cell of height larger than its radius, is much more favorable than the spherical geometry.

Conclusion Based on the above results, our experiments have been performed in a cylindrical reactor of radius $R = 70$ μm and height $h = 80$ μm. When using laser illumination, the smallest measured rate constant k_{12} for the investigated RSFPs was 1 s^{-1} and the largest was 10^5 s^{-1}. The radius of the illuminated cylinder was chosen equal to $r_0 = 10$ μm, which guarantees a cutoff rate constant $k_{12}^c \simeq 1$ s^{-1} for a diffusion coefficient $D = 100$ μm^2 s^{-1} while preserving the homogeneity of the illuminated cell. For further precautions, the fluorescence signal has been integrated over a cylinder of radius $r_0/2$ instead of r_0. Under these conditions, the effect of diffusion was entirely negligible over the full range of experimental values of the rate constants.

A.3 Optimization of LIGHTNING acquisition

In this subsection, we address the optimization of LIGHTNING acquisition.

A.3.1 Discrimination power and number of discrimination dimensions

We first determine the relationship between the number of distinguishable RSFs and the time necessary to achieve their distinction with the number n of discrimination dimensions. Along any discriminative dimension, we assume that (i) two RSFs can be distinguished if the decimal logarithm of their characteristic times differs by at least Δl along one of the considered dimensions; (ii) the two RSFs associated with the smallest characteristic times can be distinguished during a same acquisition time T. Hence, distinguishing 2^n RSFs using a single dimension requires an additional $(2^n - 2)\Delta l$ span for the logarithm of characteristic times and a total acquisition time of $T10^{(2^n-2)\Delta l}$. In contrast, achieving the same goal using n dimensions only necessitates the time nT: Using n dimensions instead of 1 reduces the acquisition time by a factor of $10^{(2^n-2)\Delta l}/n$, which exponentially increases with n and Δl.

Using the results obtained for RSFP-labeled bacteria for illustration, we adopt $n = 3$ and $\Delta l = 6M = 0.5$ (see section D.2.2). Thus 2^3 RSFs can be distinguished during the time $3T$ in 3 dimensions. In contrast, distinguishing 8 RSFs in one dimension would require at least a $7\Delta l = 3.5$ span of the logarithm of their characteristic times and the $10^{6\Delta l}T = 1000T$ time.

A.3.2 Optimization and time integration of the four illuminations

We then interrogate optimization of the four illuminations and their time integration during LIGHTNING acquisition. Two considerations have to be taken into account for optimal LIGHTNING implementation: (i) To get maximal temporal resolution, the acquisition duration should be minimal for each explored discriminative dimension (which generates an individual discriminating characteristic time); (ii) The sequential acquisitions to retrieve the overall set of characteristic times used to measure the LIGHTNING distance d_{ij} should be optimally integrated to obtain maximal fluorescence changes along each discriminative dimension in a minimal duration.

The illumination conditions in the regimes of low and high light intensities are obtained by tuning the light intensities I_1 and I_2 in order to make the changes of the fluorescence signal to be under kinetic control of the photochemical activation and of at least a thermal step, respectively. In order to reach the highest temporal resolution, the intensities

should be fixed at their highest values in their respective regimes. Hence the light intensities $I_{1,low}$ and $I_{2,low}$ should remain below the cutoff light intensities I_1^c and I_2^c for all the imaged RSFPs (see Supplementary Table 3) whereas $I_{1,high}$ and $I_{2,high}$ should be fixed at the highest accessible values in the high intensity one.

LIGHTNING implementation for fluorescence imaging of RSFPs further necessitates the integration of the four conditions of illumination yielding the set $\left(\tau_{low}^{\mathrm{I}}, \tau_{low}^{\mathrm{II}}, \tau_{high}^{\mathrm{I}}, \tau_{high}^{\mathrm{II}}\right)$. To benefit from the largest amplitudes of fluorescence changes under the four conditions of illuminations, we propose to have initially all the RSFPs in their thermodynamically stable state and to apply the sequence 1) $\{I_{1,low}\}$, 2) $\{I_{1,low}, I_{2,low}\}$, 3) $\{I_{1,high}\}$, and 4) $\{I_{1,high}, I_{2,high}\}$ upon fixing the duration of acquisition along each discriminative dimension to five times the characteristic time of the slowest RSFP.

A.3.3 Optimization of sampling

LIGHTNING exploits the time response of the RSF fluorescence to several changes of illuminations. Up to this point, we assumed instantaneous reading out of the flux of emitted fluorescence photons. However, these conditions are not met in real detecting instruments: the emitted fluorescence photons are collected over a collecting time t_c fixed by the acquisition frequency of the detecting device. Whereas fast-responding detectors are compatible with acquisition frequencies higher than 1 MHz (like the photomultipliers used for acquiring the RSFP photochemical information), cameras can typically acquire images up to 1 kHz. More precisely, they collect photons over a frame time t_c down to 1 ms and the acquired frame is then transferred in a time t_d. The time delay t_d is much shorter than the frame acquisition time t_c. With RSFPs, the fluorescence signal does not significantly change at the t_d timescale so as to be neglected in the analysis of the time series of fluorescence used to extract the characteristic times. The sequential acquisition of the fluorescence signal over t_c-wide windows yielding discrete amounts of data points for LIGHTNING processing interrogates the optimization of acquisition and processing of data (see Supplementary Figure 53).

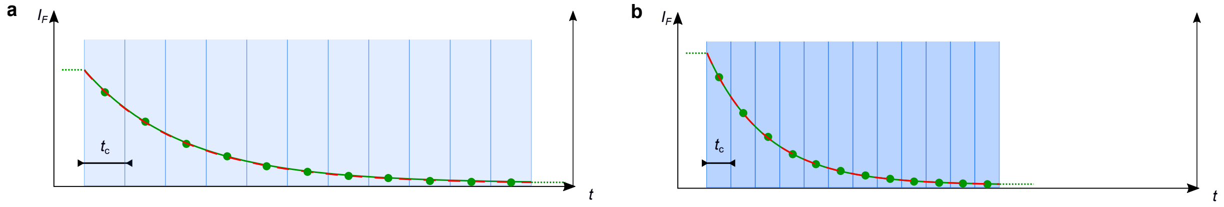

Supplementary Figure 53: Sampling of LIGHTNING acquisition. The illumination is maintained constant and the emitted photons are collected over successive time windows of width t_c. With a camera, the image built during t_c is then transferred in a time $t_d \ll t_c$ so as to be neglected in the figure. The time series of fluorescence signals (averaged over t_c) retraces the kinetics of the photoactivation step in a regime of low light intensity (**a**) whereas it integrates the kinetics of thermal steps in a regime of high light intensity (**b**). Dashed green line: Time evolution of the concentrations in the bright states of the RSFP; Green disks: Collected fluorescence signals over t_c; Dashed red line: Time profile of the fluorescence signal extracted from analyzing the fluorescence evolution upon applying illumination and used to extract the characteristic times along each discriminative dimension. Illumination I of RSFPs exhibiting negative photochromisms at $\lambda_1 = 488$ nm is used for illustration.

To address this issue, we simulated the effect of noise on a typical fluorescence evolution and studied the influence of the collecting time t_c on the determination of the characteristic time. The product νt_c, where ν is the number of analyzed fluorescence data, was constant and equal to the considered time window for extracting the characteristic time. Hence the smaller t_c, the wider the fluctuation of the number of collected photons. More precisely, noise varies as $1/\sqrt{t_c}$, i.e. $\sqrt{\nu}$. We considered a monoexponential decay $I_F(t) = \exp(-t/\tau_F)$ where $\tau_F = 1$ in arbitrary units (a. u.) with an additive Gaussian white noise $\epsilon\xi(t)$ with $\epsilon = 0.0032\sqrt{\nu}$, $\langle\xi(t)\rangle = 0$, and $\langle\xi(t)\xi(t')\rangle = \delta(t - t')$, which

was representative of the experimental noise properties for a concentration of 20 μM when using the photomultiplier detecting system. 1000 decays have been generated over a time window of 10 a. u. for each t_c value. Typically we analyzed $\nu = 1001$ data points for $t_c = 0.01$ and $\nu = 5$ data points for $t_c = 2$. The characteristic times were determined using both a monoexponential reduction over a time window of 5 a. u. and a spectrum of relaxation times over 10 a. u (see Section B). The variations of the mean value $\langle l \rangle$ and the standard deviation σ_l of the decimal logarithm of the characteristic time with respect to the number ν of analyzed data points are given in Supplementary Figure 54 for the two methods of data processing.

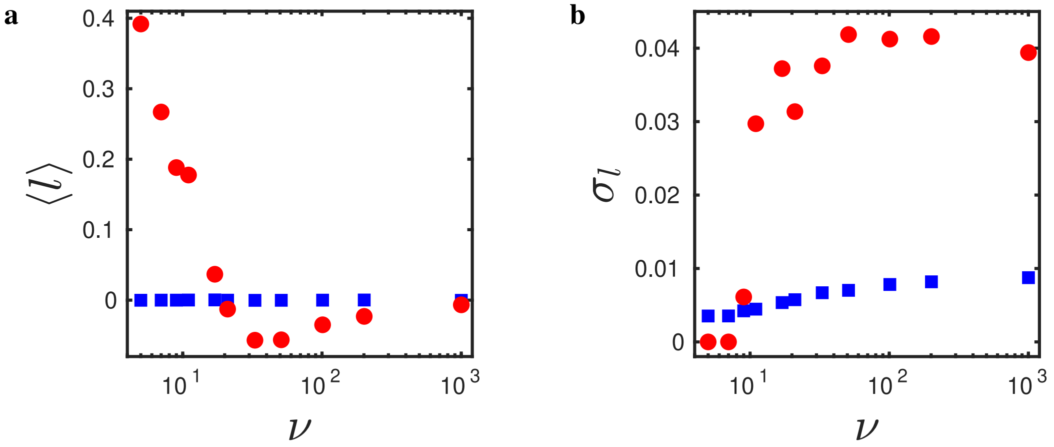

Supplementary Figure 54: Mean values $\langle l \rangle = \langle \log(\tau) \rangle$ (**a**) and standard deviations σ_l (**b**) of the decimal logarithm of the characteristic times τ versus amount ν of data points used to sample 1000 noisy exponential decays $\exp(-t/\tau_F) + \epsilon\xi(t)$ over the same time window. $\xi(t)$ is a Gaussian white noise of zero mean and unit variance, $\epsilon = 0.0032\sqrt{\nu}$, and $\tau_F = 1$ a. u. The blue squares give the results of a monoexponential reduction and the red circles are deduced from a spectrum approach.

In both cases, the effect of noise remains small and the standard deviation σ_l is smaller than 0.01 using a monoexponential reduction and smaller than 0.04 using the spectrum approach. The results provided by the fitting approach are excellent. The mean value $\langle l \rangle$ agrees with $\log(\tau_F) = 0$ to within 0.01% for all ν. The standard deviation σ_l even decreases as the amount ν of analyzed data decreases due to the decrease of noise amplitude. Whereas the fitting approach leads to continuous values of l, the maximum of the spectrum is only found in discrete values of l. For more than 11 analyzed data points, the spectrum approach provides a good evaluation of the characteristic time with a satisfying accuracy. For $\nu < 11$ searching for the maximum of the spectrum always leads to the same value as l because the time step in logarithmic units is larger than the noise-induced uncertainty about l. Hence, the standard deviation σ_l vanishes but the value of $\langle l \rangle$ is far from $\log(\tau_F) = 0$. Due to the large number of analyzed data points, analogous results are obtained when considering a logarithmic increase of t_c to sample fluorescence evolution.

From this series of simulations, we concluded that LIGHTNING should favor sampling with a small number of data points with individual low level of noise and preferentially exploit a monoexponential reduction instead of the spectrum approach to process the data (see also Section B).

A.3.4 Optimization of the photon budget and the acquisition duration

To address the photon budget and the acquisition duration of LIGHTNING which determines its signal-to-noise ratio and temporal resolution respectively, we computed the number of detected photons emitted from a pixel of volume V containing a RSF solution at a variable concentration $C(t)$ under illuminations $i =$I, II. For the sake of simplicity

we considered that only excitation of intensity I_1 at the wavelength λ_1 leads to fluorescence emission and assumed the following monoexponential decay for the concentration $C^{\mathrm{I}}(t) = C_{tot} \exp(-t/\tau^{\mathrm{I}})$ for illumination I and $C^{\mathrm{II}}(t) = C_{tot} \left(1 - \exp(-t/\tau^{\mathrm{II}})\right)$ for illumination II. The brightness can be written in the form $Q = \epsilon(\lambda_1)\Phi_F$ where $\epsilon(\lambda_1)$ is the molar absorption coefficient at the wavelength λ_1 and Φ_F the quantum yield of RSF fluorescence. The collection factor of the objective is denoted by ρ. To derive orders of magnitude, we adopted $C_{tot} = 1$ μM, a cubic pixel of length 0.3 μm and $\rho = 0.25$. We chose RSFPs **1** and **2** as typical examples of proteins associated with small and large cross sections of photoisomerization, respectively. The absorption coefficients and the fluorescence quantum yields of RSFPs **1** and **2** are given in Supplementary Table 1, the cross sections in Supplementary Table 3, and the values of the characteristic times τ^{I}_{low}, τ^{II}_{low}, τ^{I}_{high}, and $\tau^{\mathrm{II}}_{high}$ and the corresponding intensities I_1 in Supplementary Table 7.

In the case of continuous illumination, the numbers of photons $n^i_{h\nu}$ detected during five times the characteristic time τ^{i} for illuminations i =I, II are obtained by integrating the fluorescence intensity between 0 and $5\tau^{\mathrm{i}}$ leading to

$$n^{\mathrm{I}}_{h\nu} = \epsilon(\lambda_1)\Phi_F I_1 \rho V C_{tot} \tau^{\mathrm{I}} \left(1 - e^{-5}\right) \quad (74)$$

$$n^{\mathrm{II}}_{h\nu} = \epsilon(\lambda_1)\Phi_F I_1 \rho V C_{tot} \tau^{\mathrm{II}} \left(4 + e^{-5}\right) \quad (75)$$

For given illumination and detection conditions, the number of detected photons is about four times larger during a fluorescence increase than a decay. We determined the number of detected photons during fluorescence evolution using Eqs.(74,75). The results are given in Supplementary Table 4 and Supplementary Table 5.

Supplementary Table 4: Evaluation of the number of detected photons $n^i_{h\nu}$ for RSFP **1** under the continuous illumination conditions $i = \mathrm{I}_{low}, \mathrm{II}_{low}, \mathrm{I}_{high}, \mathrm{II}_{high}$ at intensity I_1 at the wavelength $\lambda_1 = 488$ nm. τ^{i} is the characteristic time associated with fluorescence evolution under illuminations i. The parameters values are $C_{tot} = 1$ μM, $V = 0.027$ $\mu\mathrm{m}^3$, $\rho = 0.25$, $\epsilon(\lambda_1) = 80000$ $\mathrm{M}^{-1}.\mathrm{cm}^{-1}$, $\Phi_F = 0.8$.

Illumination	I_1	τ^{i}	$n^i_{h\nu}$
	(ein.m^{-2}.s^{-1})	(ms)	
I_{low}	2	140	7 236
II_{low}	2	31	6 464
I_{high}	200	4.5	23 260
II_{high}	200	0.18	3 754

Supplementary Table 5: Evaluation of the number of detected photons $n^i_{h\nu}$ for RSFP **2** under the continuous illumination conditions $i = \mathrm{I}_{low}, \mathrm{II}_{low}, \mathrm{I}_{high}, \mathrm{II}_{high}$ at intensity I_1 at the wavelength $\lambda_1 = 488$ nm. τ^{i} is the characteristic time associated with fluorescence evolution under illuminations i. The parameters values are $C_{tot} = 1$ μM, $V = 0.027$ $\mu\mathrm{m}^3$, $\rho = 0.25$, $\epsilon(\lambda_1) = 75000$ $\mathrm{M}^{-1}.\mathrm{cm}^{-1}$, $\Phi_F = 0.2$.

Illumination	I_1	τ^{i}	$n^i_{h\nu}$
	(ein.m^{-2}.s^{-1})	(ms)	
I_{low}	2	2.2	27
II_{low}	2	25	1 222
I_{high}	200	0.33	400
II_{high}	200	0.077	376

Our data predict that the typical numbers of photons extracted from a (0.3 μm)^3-pixel at micromolar concentration for each characteristic time is about 500 and 10000 for the fast and slow RSFPs, respectively: More photons are detected for RSFPs associated with large values of the product $I_1\tau^{\mathrm{i}}$, i.e. small cross sections such as for RSFP **1**. These numbers of detected photons with LIGHTNING are essentially similar to the order of magnitude obtained with

non-photoswitchable fluorophores exhibiting similar photophysical features. Indeed, the number of detected photons is reduced by a factor of 5 in the case of illumination I and a factor of 5/4 in the case of illumination II. Moreover the duration of the experiments allowing us to determine the kinetic fingerprint of an RSFP for continuous illumination is equal to five times the sum of the four characteristic times. According to Supplementary Table 4 and Supplementary Table 5, we obtain 878 ms for RSFP **1** and 138 ms for RSFP **2**. Hence, in the optimization of the choice of an RSFP, the number of detected photons is balanced by the duration of the acquisition.

B LIGHTNING data processing

We have chosen to characterize the fluorescence evolution of an RSFP for given light intensities by a single time, which is not obvious in particular when the fluorescence evolution is not monotonous. In order to check the validity of the monoexponential reduction of the signal, we adapted a correlation method which leads to a spectrum of characteristic times without requiring hypotheses on the multiexponential nature of the fluorescence evolution.[15–22] For all RSFPS and investigated light intensities, the results deduced from the spectra of characteristic times legitimate the chosen data processing method based on a monoexponential reduction.

B.1 Spectrum of the characteristic times of RSF fluorescence evolution

B.1.1 Generating a spectrum

A spectrum of relaxation times has been used to characterize the kinetics of RSFPs. The spectrum is built by correlating the fluorescence evolution $I_F(t)$ with a function $f_l(t)$ where the parameter l is the decimal logarithm of a relaxation time[15–22]

$$S(l) = \frac{\int_0^{\beta 10^l} \left(I_F(t) - \langle I_F(t)\rangle\right)\left(f_l(t) - \langle f_l(t)\rangle\right) dt}{\int_0^{\beta 10^l} \left(e^{-\frac{t}{10^l}} - \langle e^{-\frac{t}{10^l}}\rangle\right)\left(f_l(t) - \langle f_l(t)\rangle\right) dt} \tag{76}$$

The average $\langle\rangle$ represents the integration between 0 and $\beta 10^l$. The value of the parameter β depends on the function $f_l(t)$. The denominator is a normalization condition.

When the fluorescence intensity is a monoexponential function associated with a relaxation time τ_F

$$I_F(t) = \left(I_F^0 - I_F^\infty\right)\exp\left(-t/\tau_F\right) + I_F^\infty \tag{77}$$

the value of β is chosen to ensure that the spectrum $S(l)$ possesses an extremum for $l_F = \log(\tau_F)$ as shown in Supplementary Figure 55. Due to the chosen normalization, the amplitude of the extremum $S(l_F)$ is equal to the preexponential factor $I_F^0 - I_F^\infty$. The sign of the preexponential factor determines the nature of the extremum, a positive factor leading to a maximum and a negative factor leading to a minimum of $S(l)$. When the fluorescence evolution $I_F(t)$ is not correctly described by a monoexponential function but behaves as a sum of $n_s - 1$ exponential terms, the spectrum of the relaxation times displays between one and $n_s - 1$ extrema.

The spectra of relaxation times can be computed for different choices of the function $f_l(t)$. The fluorescence evolution $I_F(t)$ being a linear combination of exponential functions (see section A.1), the choice $f_l(t) = e^{-\frac{t}{10^l}}$ is intuitive. The correlation with an exponential is known to produce spectra with broad peaks, i.e. a moderate time resolution but little sensitivity to the signal-to-noise ratio of the fluorescence signal.[21,22] In order to characterize the kinetics of fluorescence photoswitching of an RSF under a given illumination by a single quantity, we chose to compute the spectrum using the exponential correlator and look for the absolute extremum of the spectrum. For the exponential correlator we find $\beta = 3.25$.

The analytical expression of the spectrum $S(l)$ for the monoexponential fluorescence evolution $I_F(t)$ given in Eq.(77) is given by

$$S(l) = \left(I_F^0 - I_F^\infty\right) \frac{\exp\left(-3.25 \times 10^{l-l_F}\right)\left(1.38 + 1.20 \times 10^{l-l_F}\right) - 1.38 + 3.28 \times 10^{l-l_F}}{10^{l-l_F}\left(1 + 10^{l-l_F}\right)} \quad (78)$$

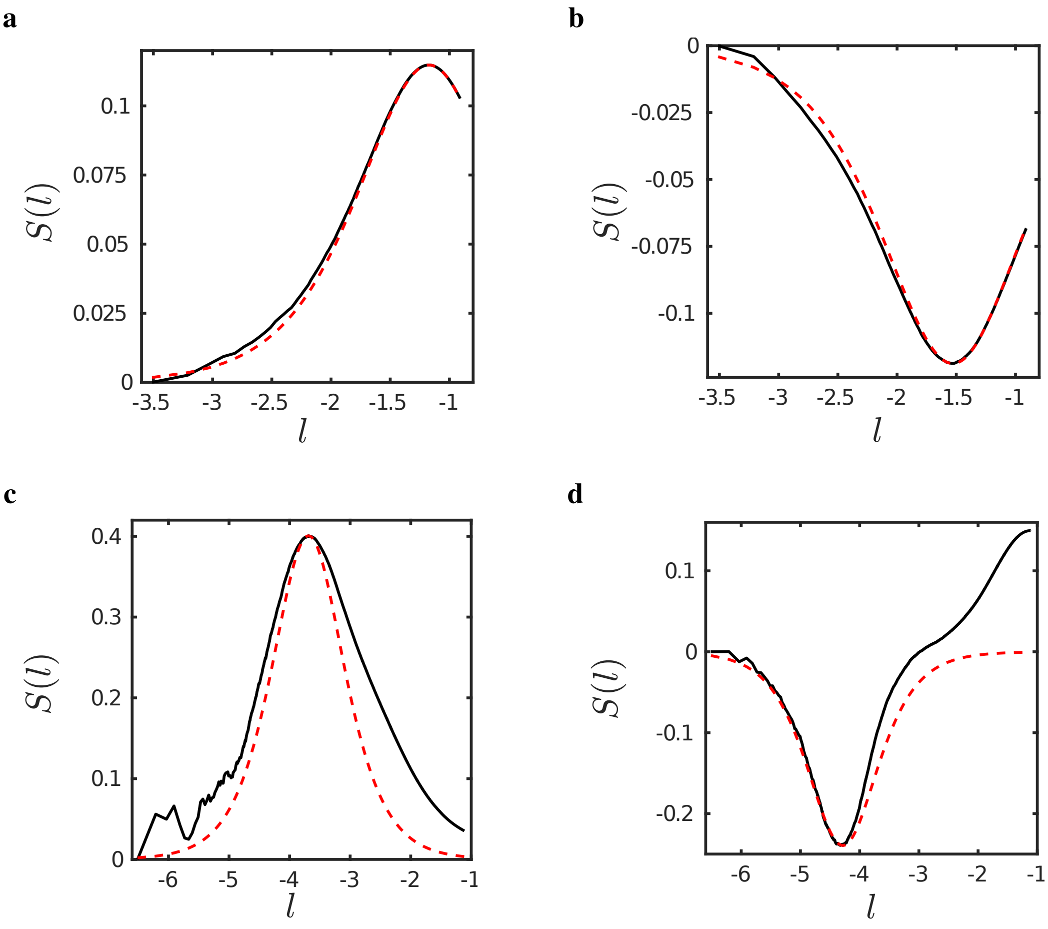

Supplementary Figure 55: Spectra of the relaxation times associated with the RSFP **2** in solution. The solid lines are obtained by computing $S(l)$ for the experimental fluorescence evolution $I_F(t)$. The dashed red lines are obtained when replacing $I_F(t)$ by a monoexponential function with the relaxation time associated with the absolute extremum of the spectrum built from the experimental fluorescence evolution. **a**: Illumination I with $I_{1,low} = 0.23$ Ein.m^{-2}.s^{-1} at the wavelength $\lambda_1 = 488$ nm; **b**: Illumination II with $I_1 = 0.07$ Ein.m^{-2}.s^{-1} at the wavelength $\lambda_1 = 488$ nm and $I_{2,low} = 0.10$ Ein.m^{-2}.s^{-1} at the wavelength $\lambda_2 = 405$ nm; **c**: Illumination I with $I_{1,high} = 128.8$ Ein.m^{-2}.s^{-1} at the wavelength $\lambda_1 = 488$ nm; **d**: Illumination II with $I_1 = 0.15$ Ein.m^{-2}.s^{-1} at the wavelength $\lambda_1 = 488$ nm and $I_{2,high} = 106$ Ein.m^{-2}.s^{-1} at the wavelength $\lambda_2 = 405$ nm.

As shown in Supplementary Figure 55a,b, the peak of the spectrum is symmetrical with respect to the extremum and its width at half height equals $\Delta l = 1.4$. The logarithm of the relaxation time l has to differ by more than 1.5 from l_F for the corresponding spectrum value $S(l)$ to be smaller than 10% of the amplitude $S(l_F)$ of the extremum.

The generalization to a linear combination of exponential functions is straightforward.

In the case where the fluorescence intensity $I_F(t)$ is a sum of two exponential terms with identical prefactors p and different characteristic times $\tau_F < \tau_{F'}$, the spectrum has either one or two extrema. The spectrum shows one or

two maxima if the prefactors are positive and one or two minima if the prefactors are negative. If $l_{F'} - l_F < 1.1$, the spectrum has a single extremum for l between l_F and $l_{F'}$. In this case, the amplitude of the extremum is between $1.3p$ and $2p$. On the contrary, if $l_{F'} - l_F > 1.1$, the spectrum has two extrema. If $l_{F'} - l_F > 1.7$, the abscissa of the extrema differ from l_F and $l_{F'}$ by less than 5%. Moreover, the values of the spectrum associated with the extrema are less than 7% larger than the prefactor p.

For a fluorescence intensity $I_F(t)$ equal to the difference of two exponential terms with identical prefactors and characteristic times $\tau_F < \tau_{F'}$, the spectrum has always one maximum and one minimum. If $l_{F'} - l_F > 1.7$, then the abscissa of the extrema differ from l_F and $l_{F'}$ by less than 4%. In addition, the amplitudes of the extrema are less than 6% smaller than the prefactor.

In the general case of sufficiently different prefactors the position of the absolute extremum is close to the characteristic time of the exponential associated with the largest prefactor.

Regardless of the multiexponential nature of the signal, it is always possible to use the spectrum of characteristic times to characterize the evolution by a single quantity. The abscissa of the absolute extremum of the spectrum provides the desired characteristic time.

B.1.2 Application to RSFPs

Supplementary Figure 55 displays the spectra of the relaxation times obtained for the RSFP **2** in four illuminations. The spectrum directly deduced from Eq.(76) for the experimental fluorescence evolution $I_F(t)$ is referred to as the experimental spectrum and the spectrum obtained when replacing $I_F(t)$ by a monoexponential function with a relaxation time matching the extremum observed in the experimental spectrum is called the predicted spectrum.

For the low light intensities used to obtain Supplementary Figure 55a and Supplementary Figure 55b, the photochemical reactions are rate-limiting steps and an excellent match between the experimental and predicted spectra is observed. This agreement proves that, in these low light intensity regimes, the characteristic times deduced by reducing $I_F(t)$ to a monoexponential function (see section B.2) are properly evaluated. Hence, for sufficiently low light intensities, a single characteristic time or equivalently a two-state model is sufficient to adequately describe the kinetics of fluorescence photoswitching.

For the high light intensities used to obtain Supplementary Figure 55c and Supplementary Figure 55d, the reaction rate depends on some thermal steps. The asymmetrical broadening of the experimental spectrum in Supplementary Figure 55c accounts for at least a biexponential behavior with close relaxation times but different preexponential factors. Nevertheless the value of the characteristic time deduced from a monoexponential reduced function satisfactorily coincides with the position of the maximum of the experimental spectrum. The experimental spectrum in Supplementary Figure 55d exhibits a minimum, an inflection point, and a maximum at the largest τ values. However the good agreement between the minima of the experimental and predicted spectra still validates the use of a monoexponential reduced function to $I_F(t)$.

More generally, only one extremum was observed for all studied RSFPs for illumination I: a minimum for **4** and **5** and a maximum for the other 20 RSFPs, as expected from their respective positive and negative photochromism. In contrast, for illumination II, we extract the abscissa of the minimum of the spectrum (maximum for **4** and **5**, resp.) observed at all light intensities and ignored the maximum (minimum for **4** and **5**, resp.) which could be observed at the highest light intensities. As mentioned above, the perturbation of the extremum by a second relaxation time of the fluorescence photoswitching remains small when the two relaxation times are sufficiently different or sufficiently close, or when the preexponential factor associated with the second relaxation time is sufficiently small. One of these conditions is always met for all considered RSFPs.

B.2 Monoexponential reduction of RSF fluorescence evolution

In this section, we propose a simple protocol for processing RSF kinetics and apply the results to the fluorescence evolution obtained for the illuminations I and II.

B.2.1 A kinetic filter

Choosing a time window around a targeted time scale plays the role of a kinetic filter for fluorescence evolution. According to the concentration evolution of RSFs engaged in first-order reactions between n_s states, the fluorescence evolution is a linear combination of exponential terms (see Eq.(32)). Focusing on the dynamics occurring at a time scale amounts to selecting the small number of exponential terms associated with relaxation times close to this time scale. Two situations are mainly encountered:

- A single relaxation time τ_a is close to the targeted time scale. For a time t close to τ_a, the exponential terms associated with the m first relaxation times obeying $\tau_i < \tau_a$ are negligible with respect to $\exp(-t/\tau_a)$. At the same time scale, the other $n_s - m - 2$ exponential terms can be considered constant. Consequently the fluorescence evolution is close to

$$I_F = U_a \exp\left(-\frac{t}{\tau_a}\right) + W_a \tag{79}$$

  with $W_a = W + \Sigma_{i=n_s-m-2}^{n_s} U_i$ where W and U_i are defined under Eq.(32). In this case, the effect of the kinetic filter legitimates using the following monoexponential reduction

$$I_F \simeq \left(I_F^0 - I_F^\infty\right) \exp\left(-\frac{t}{\tau}\right) + I_F^\infty \tag{80}$$

- If two relaxation times $\tau_a < \tau_b$ are close to the targeted time scale, the observed fluorescence evolution is biexponential

$$I_F = U_a \exp\left(-\frac{t}{\tau_a}\right) + U_b \exp\left(-\frac{t}{\tau_b}\right) + W_b \tag{81}$$

  with $W_b = W + \Sigma_{i=n_s-m-3}^{n_s} U_i$. The observed behavior depends on the sign of the product $U_a U_b$ of the preexponential factors:

  - If $U_a U_b > 0$, fluorescence evolution is monotonous. We then assume that Eq.(81) can be approximated by the monoexponential function given in Eq.(80). The initial $(t = 0)$ and final $(t \to \infty)$ conditions on I_F lead to

$$I_F^0 \simeq U_a + U_b + W_b \tag{82}$$

$$I_F^\infty \simeq W_b \tag{83}$$

    Substituting Eqs.(82,83) into Eq.(80), we perform a first-order expansion of the exponential terms of Eq.(81) around $t = 0$ and obtain

$$\tau \simeq \frac{\tau_a \tau_b (U_a + U_b)}{U_a \tau_b + U_b \tau_a} \tag{84}$$

    Hence the fluorescence evolution can be approximated by a monoexponential function with a characteristic time between τ_a and τ_b.

– If $U_a U_b < 0$, fluorescence evolution displays an extremum and the relaxation time τ_a is associated with the first part of the evolution before the extremum whereas τ_b is associated with the second part of the evolution. We consider the case where the extremum is a maximum, i.e.

$$0 < \tau_a < \tau_b \tag{85}$$

$$0 < U_b < \mid U_a \mid \tag{86}$$

$$U_a + U_b + W_b > 0 \tag{87}$$

The extension to the case of a minimum is straightforward. According to Eq.(81), the maximum of the fluorescence evolution

$$I_F^{\max} = U_a \exp\left(-\frac{t^{\max}}{\tau_a}\right) + U_b \exp\left(-\frac{t^{\max}}{\tau_b}\right) + W_b \tag{88}$$

is reached at time

$$t^{\max} = \frac{\tau_a \tau_b}{\tau_b - \tau_a} \ln\left(\frac{\mid U_a \mid \tau_b}{U_b \tau_a}\right) \tag{89}$$

Substituting Eq.(89) into Eq.(88), we perform a first-order expansion of $I_F^{\max}$ with respect to the parameter τ_a/τ_b and obtain

$$I_F^{\max} \simeq U_b \left[1 - \frac{\tau_a}{\tau_b} \ln\left(\frac{\mid U_a \mid \tau_b}{U_b \tau_a}\right)\right] + W_b \tag{90}$$

We approximate the first part of the evolution before the maximum by a monoexponential function of characteristic time τ reaching $I_F^{\max}$ for $t \to \infty$

$$I_F \simeq \left(I_F^0 - I_F^{\max}\right) \exp\left(-\frac{t}{\tau}\right) + I_F^{\max} \tag{91}$$

in which I_F^0 is given in Eq.(82) and $I_F^{\max}$ in Eq.(88). We perform a first-order expansion of the exponential terms around $t = 0$ in the biexponential expression of I_F given in Eq.(81) and in the monoexponential reduced function given in Eq.(91) and obtain the following relation between the fitting parameter τ and the relaxation times τ_a and τ_b

$$\tau \simeq \tau_a \left[1 + \frac{U_b \tau_a}{U_a \tau_b} \ln\left(\frac{\mid U_a \mid \tau_b}{U_b \tau_a}\right)\right] \tag{92}$$

The conditions given in Eqs.(85,86) ensure that $\tau < \tau_a$. The correction to τ_a is small provided that $\tau_a \ll \tau_b$ and $U_b \ll \mid U_a \mid$. This result supports the choice of a monoexponential reduced function for the increasing part of the fluorescence evolution before the maximum.

The decreasing part of the fluorescence evolution can be processed in a similar way to extract a single characteristic time different from the one extracted from the increasing part.

This subsection gives the conditions for which it is legitimate to use a monoexponential reduced function to the fluorescence evolution. It is essential to choose a time window in which the evolution is monotonous. The reduction procedure provides a characteristic time close to the relaxation times of the kinetics at the targeted time scale.

B.2.2 Application to RSFPs

In the case of RSFPs, the previously studied cases have been encountered. For illuminations I and II and for sufficiently low light intensities the kinetics of RSFPs is well-described by a two-state model and monoexponential reduction to fluorescence evolution using Eq.(80) is fully justified. The fluorescence data are processed over the entire acquisition window. For illumination I and high light intensities, the fluorescence evolution is described by a linear combination of at least two exponential functions with negative prefactors for RSFPs **4** and **5**, and positive prefactors for the 20 others. It can be approximated by the monoexponential reduced function given in Eq.(80). The estimated characteristic time is between the smallest relaxation time and the largest one selected by the kinetic filter. This assessment is sufficiently accurate for the purpose of RSFPs discrimination. For illumination II and high light intensities, the fluorescence evolution displays a minimum for $t = t^{\max}$ for RSFPs **4** and **5**, and a maximum for the 20 others. Nevertheless a monoexponential reduced function can be used to account for the first part of the curve before the maximum provided that fluorescence evolution does not too strongly depart from a monotonous behavior. In the measurements performed in RSFP solutions, the processed time window has been restricted to $[0, t^{\max}]$. Then the characteristic time deduced from the fitting protocol is close to the smallest relaxation time selected by the kinetic filter.

B.3 Comparison of the spectrum and the monoexponential reduction for extracting characteristic times

The two data processing methods proposed for extracting the LIGHTNING characteristic times yield similar behavior of the dependence of the inverse of the characteristic time versus the light intensity. As shown in Supplementary Figure 56, excellent match of the linear increase at low light intensity are observed in both cases. Less than a factor of 2 between the characteristic times extracted from the spectrum and the monoexponential reduction of the fluorescence evolution has been obtained in the high intensity regime, which is satisfactory for the purpose of discrimination.

It is worth noting that autofluorescence can be modeled by a constant term in the fluorescence evolution already present in Eqs. (77,79,81). The spectrum of characteristic times and the characteristic time deduced from a monoexponential fitting are independent of constants terms. Consequently, data processing eliminates autofluorescence. The quality of the results obviously depends on the noise and the amplitude of fluorescence variation must be at least as large as the noise level.

In conclusion to this section, the position of the extremum obtained by directly computing the spectrum of relaxation times from the experimental fluorescence evolution $I_F(t)$ is close to the position of the extremum of the spectrum obtained by reducing the data to a monoexponential function. The curve fitting based on a monoexponential function is more easily and faster implemented and less sensitive to noise than the spectrum of characteristic times approach. Therefore the monoexponential reduction has been chosen to extract the LIGHTNING kinetic signature.

C Kinetic models of fluorescence photoswitching in RSFPs

The present publication does not address the discussion of all aspects of the observed evolution of the RSFP fluorescence signal upon the illuminations I and II in the regimes of low and high light intensities, which have been observed in Supplementary Figure 21–Supplementary Figure 42a,b. This discussion is left for another publication interrogating the mechanism of the RSFP photoconversion. Below we provide general guidelines accounting for the observed behavior. Starting from the available information in the literature and from our own observations, we have built minimal kinetic models accounting for the light-intensity dependence of the characteristic time extracted from analyzing the fluorescence changes after light jumps for the RSFPs.

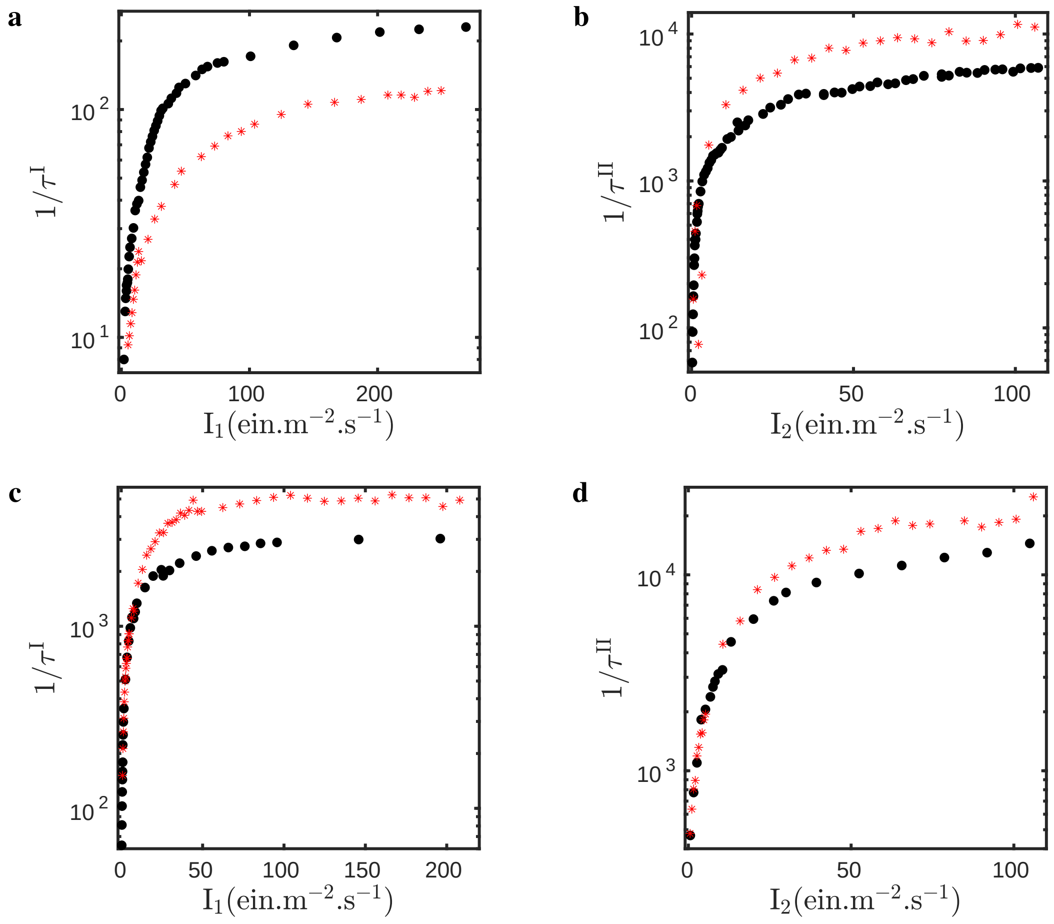

Supplementary Figure 56: Kinetic characterization of RSFP **1** (**a**,**b**) and **2** (**c**,**d**) at 20 μM in pH=7.4 PBS (50 mM sodium phosphate, 150 mM NaCl); T = 298 K. **a**: Illumination I: Inverse of the relaxation time $1/\tau^{I}$ versus light intensity I_1 at $\lambda_1 = 488$ nm, **b**: Illumination II: Inverse of the relaxation time $1/\tau^{II}$ versus light intensity I_2 at $\lambda_2 = 405$ nm. The relaxation times have been extracted using either the extremum of the spectrum of characteristic times (red star) or monoexponential fitting (black disk).

C.1 An eight-state photoswitching mechanism for RSFPs relevant in the 1 μs–1 s time window

The detailed photoswitching mechanisms of all RSFPs are not entirely known. We rely on literature to start from the photoswitching mechanism given in Supplementary Figure 57a, which focusses on reported steps of isomerization,[23–27] protonation,[23–27] and the existence of a dark state[28] in order to account for the photochemical behavior of negative RSFPs in the 1 μs–1 s time window. We use existing information on the more studied RSFPs, such as Dronpa and Dronpa-2, and admit that the other RSFPs obey a similar mechanism.

The thermodynamically stable Cis-O$^-$ state at neutral pH,[24] denoted A, absorbs light mainly at the wavelength $\lambda_1 = 480$ nm and to a lesser extent at the wavelength $\lambda_2 = 405$ nm forming the singlet excited state A* from which three reactions paths begin. The Cis-O$^-$ state is recovered (i) by singlet relaxation, (ii) through a dark state E, and (iii) by a longer reaction path described below. Isomerization of A* yields the Trans-O$^-$ state, denoted B, which presumably possesses a similar absorption spectrum and a similar order of magnitude of relaxation rate of its singlet excited state B* as the Cis-O$^-$ state. Protonation of B leads to the Trans-OH state, denoted C, which is the predominant acid-base state of the trans isomer at neutral pH.[23–27] As a consequence, we consider that the singlet excited state C*

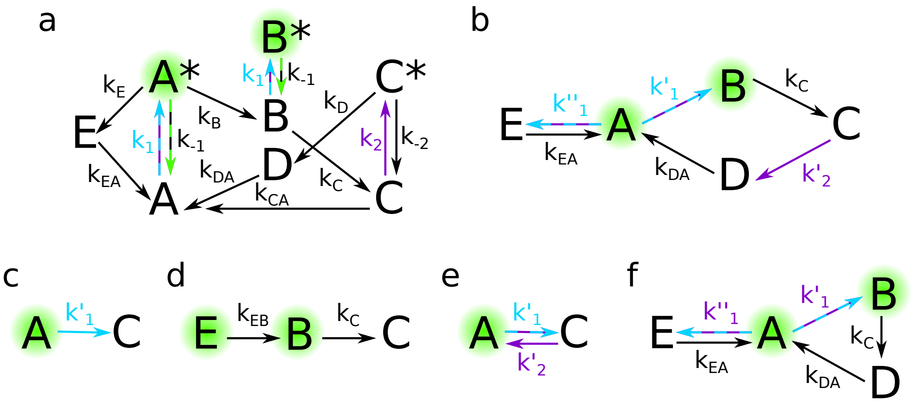

Supplementary Figure 57: Photoswitching mechanisms of negative RSFPs relevant in the 1 μs–1 s time window. **a**: Eight-state mechanism; **b**: Five-state mechanism relevant in the investigated light intensity range obtained after elimination of the fast variables A*, B*, and C*; **c**: Reduced mechanism valid for low light intensity at $\lambda_1 = 480$ nm and no light at $\lambda_2 = 405$ nm (illumination I_{low}); **d**: Reduced mechanism valid for high light intensity at $\lambda_1 = 480$ nm and no light at $\lambda_2 = 405$ nm (illumination I_{high}); **e**: Reduced mechanism valid for low light intensities at $\lambda_1 = 480$ nm and $\lambda_2 = 405$ nm (illumination II_{low}); **f**: Reduced mechanism valid for light intensity at $\lambda_1 = 480$ nm and high light intensity at $\lambda_2 = 405$ nm (illumination II_{high}). The order of magnitude of the rate constants and light intensities associated with each case are given in Supplementary Table 6. The photochemical rate constants associated with absorption at $\lambda_1 = 480$ nm ($\lambda_2 = 405$ nm, resp.) are cyan (purple, resp.). The thermal rate constants are black. Species with a green halo are bright or acquire brightness due to mechanism reduction.

of the Trans-OH state is only formed by absorption at the wavelength $\lambda_2 = 405$ nm. The Cis-O$^-$ state may be directly formed from Trans-OH by thermal isomerization or obtained by light excitation of Trans-OH, isomerization yielding the Cis-OH state denoted D, and deprotonation.[23–27]

The introduction of the state E in the RSFP mechanism is supported by the observation of a long-lived triplet state in one relaxation pathway of some green fluorescent proteins possibly involving several other dark states.[28] The introduction of other dark states associated with comparable relaxation times would lead to qualitatively similar behaviors of fluorescence with additional characteristic times that would unnecessarily complicate the description. The orders of magnitude of the rate constants involved in Supplementary Figure 57a are given in Supplementary Table 6.

The eight-state photoswitching mechanism displayed in Supplementary Figure 57a possesses both photochemical and thermal steps. Therefore it is well-adapted to evaluate the impact of changing the rate-limiting steps by tuning light intensities. We consider two different illuminations I and II. In illumination I, a light intensity I_1 at the wavelength $\lambda_1 = 488$ nm is applied to the sample initially in the bright Cis-O$^-$ state. In illumination II, a light intensity I_2 at the wavelength $\lambda_2 = 405$ nm and a fixed intensity I_1 at the wavelength $\lambda_1 = 480$ nm are applied to the sample initially in the dark Trans-OH state. The complementary illumination at $\lambda_1 = 480$ nm is added to obtain a fluorescent signal since the brightness of the states A and B at the wavelength $\lambda_2 = 405$ nm are small.

Supplementary Table 6: Order of magnitude of parameters of the photoisomerization mechanisms of RSFPs.

	parameter	order of magnitude
eight-state mechanism		
absorption cross sections[23–27]	ϵ_{11}, ϵ_{22}	10^3-10^4 $m^2.mol^{-1}$
	ϵ_{12}	10^2-10^3 $m^2.mol^{-1}$
singlet relaxation[23–27]	k_{-1}	10^9 s^{-1}
	k_{-2}	10^{11} s^{-1}
isomerization[23–27]	k_B	10^7 s^{-1}
	k_D	10^{10} s^{-1}
proton exchange[23–27]	k_C, k_{DA}	10^3-10^6 s^{-1}
thermal isomerization[23–27]	k_{CA}	10^{-4}-10^{-2} s^{-1}
formation of state E[28]	k_E	10^7 s^{-1}
relaxation of state E[28]	k_{EA}	$10 - 10^3$ s^{-1}
five-state mechanism		
light intensities	I_1, I_2	$\ll 10^5$ $ein.m^{-2}.s^{-1}$
isomerization cross sections	σ_{11}, σ_{22}	10-10^2 $m^2.mol^{-1}$
	σ_{12}	1-10 $m^2.mol^{-1}$
cross sections of state E	σ_{11}^E	10-10^2 $m^2.mol^{-1}$
	σ_{12}^E	1-10 $m^2.mol^{-1}$
two-state mechanism I_{low}		
light intensity	I_1	$\ll$ 0.1-10^2 $ein.m^{-2}.s^{-1}$
three-state mechanism I_{high}		
light intensity	I_1	$\gg$ 10-10^5 $ein.m^{-2}.s^{-1}$
rate constant of step E$\rightarrow$B	k_{EB}	1-10^3 s^{-1}
two-state mechanism II_{low}		
light intensities	I_1	$\sim$ 0.1 $ein.m^{-2}.s^{-1}$
	I_2	$\ll$ 1-10^3 $ein.m^2.s^{-1}$
four-state mechanism II_{high}		
light intensities	I_1	$2 - 50$ $ein.m^{-2}.s^{-1}$
	I_2	$\gg$ 10-10^5 $ein.m^2.s^{-1}$

The eight-state mechanism given in Supplementary Figure 57a yields the rate laws

$$\frac{\mathrm{d}A}{\mathrm{d}t} = -k_1 A + k_{-1} A^* + k_{CA} C + k_{DA} D + k_{EA} E \tag{93}$$

$$\frac{\mathrm{d}A^*}{\mathrm{d}t} = k_1 A - (k_{-1} + k_B + k_E) A^* \tag{94}$$

$$\frac{\mathrm{d}B}{\mathrm{d}t} = k_B A^* - (k_1 + k_C) B + k_{-1} B^* \tag{95}$$

$$\frac{\mathrm{d}B^*}{\mathrm{d}t} = k_1 B - k_{-1} B^* \tag{96}$$

$$\frac{\mathrm{d}C}{\mathrm{d}t} = k_C B - (k_2 + k_{CA}) C + k_{-2} C^* \tag{97}$$

$$\frac{\mathrm{d}C^*}{\mathrm{d}t} = k_2 C - (k_{-2} + k_D) C^* \tag{98}$$

$$\frac{\mathrm{d}D}{\mathrm{d}t} = k_D C^* - k_{DA} D \tag{99}$$

$$\frac{\mathrm{d}E}{\mathrm{d}t} = k_E A^* - k_{EA} E \tag{100}$$

with the law of matter conservation $C_{\mathrm{tot}} = A + A^* + B + B^* + C + C^* + D + E$. In Eqs. (93–100), the photochemical rate constants k_1 and k_2 can be written as

$$k_1 = \epsilon_{11} I_1 + \epsilon_{12} I_2 \tag{101}$$

$$k_2 = \epsilon_{22} I_2 \tag{102}$$

where ϵ_{11} and ϵ_{12} are the absorption cross sections of A and B at the wavelengths $\lambda_1 = 488$ nm and $\lambda_2 = 405$ nm respectively, and ϵ_{22} is the absorption cross section of C at the wavelength $\lambda_2 = 405$ nm. We assume that only illumination at $\lambda_1 = 488$ nm leads to fluorescence emission. The rate constant k_{-1} takes into account the radiative and non radiative de-excitation of the singlet excited states A* and B*. We neglect the fluorescence of all species except A* and B*[23,24] leading to the fluorescence intensity written as

$$I_F = k^F_{A^*} A^* + k^F_{B^*} B^* \tag{103}$$

where $k^F_{A^*}$ and $k^F_{B^*}$ are the radiative part of the rate constants k_{-1} associated with the desexcitation of A* and B*, respectively.

Varying the light intensities I_1 and I_2 allows us to explore different kinetic regimes. We exploit the very different orders of magnitude of the rate constants and use the quasi-steady-state approximation to reduce the system given in Eqs. (93-100). The mechanism being composed of first-order steps, the condition of validity for the elimination of a fast chemical species is easy to express: The sum of the rate constants of the steps in which the considered species is a reagent must be large compared to the sum of the rate constants of the steps in which the species is a product. The fastest reactive species are eliminated first and only smaller characteristic times defining the timescale of interest remain. The next fastest species is eliminated until all characteristic times are close within the chosen time range.

C.2 Elimination of the singlet excited states – The five-state mechanism

The rate constant $k_{CA} \leq 10^{-2}$ s^{-1} is associated with a slow kinetics inaccessible in the explored time window and therefore ignored in the following. According to Supplementary Table 6, the fastest species are the singlet excited states A*, B*, and C*. Their elimination is valid if the conditions $k_1 \ll k_{-1} + k_B + k_E$, $k_1 \ll k_{-1}$, and $k_2 \ll k_{-2} + k_D$ are fulfilled, i.e. if the light intensities obey $I_1 \ll k_{-1}/\epsilon_{11}$ and $I_2 \ll k_{-2}/\epsilon_{22}$. Using Supplementary Table 6, we find that light intensities smaller than 10^5 ein.m^2.s^{-1} meet these conditions. According to the steady-state approximation, we eliminate the species A*, B*, and C* using $\frac{\mathrm{d}A^*}{\mathrm{d}t} = 0$, $\frac{\mathrm{d}B^*}{\mathrm{d}t} = 0$, and $\frac{\mathrm{d}C^*}{\mathrm{d}t} = 0$ where A^*, B^*, and C^* obey Eqs. (94,96,98). The dynamics is then given by

$$\frac{\mathrm{d}A}{\mathrm{d}t} = -\left(k_1' + k_1''\right) A + k_{DA} D + k_{EA} E \tag{104}$$

$$\frac{\mathrm{d}B}{\mathrm{d}t} = k_1' A - k_C B \tag{105}$$

$$\frac{\mathrm{d}C}{\mathrm{d}t} = k_C B - k_2' C \tag{106}$$

$$\frac{\mathrm{d}D}{\mathrm{d}t} = k_2' C - k_{DA} D \tag{107}$$

$$\frac{\mathrm{d}E}{\mathrm{d}t} = k_1'' A - k_{EA} E \tag{108}$$

where $k_1' = k_1 k_B/(k_{-1} + k_B + k_E)$, $k_1'' = k_1 k_E/(k_{-1} + k_B + k_E)$, and $k_2' = k_2 k_D/(k_{-2} + k_D)$. We obtain the reduced five-state mechanism given in Supplementary Figure 57b with the apparent photochemical rate constants

$$k_1' = \sigma_{11} I_1 + \sigma_{12} I_2 \tag{109}$$

$$k_1'' = \sigma_{11}^E I_1 + \sigma_{12}^E I_2 \tag{110}$$

$$k_2' = \sigma_{22} I_2 \tag{111}$$

where $\sigma_{11} = \epsilon_{11} k_B/(k_{-1} + k_E + k_B)$ and $\sigma_{11}^E = \epsilon_{11} k_E/(k_{-1} + k_E + k_B)$ are cross sections at the wavelength $\lambda_1 = 480$ nm and $\sigma_{12} = \epsilon_{12} k_B/(k_{-1} + k_E + k_B)$, $\sigma_{12}^E = \epsilon_{12} k_E/(k_{-1} + k_E + k_B)$, and $\sigma_{22} = \epsilon_{22} k_D/(k_{-2} + k_D)$ are cross sections at the wavelength $\lambda_2 = 405$ nm.

Using Eq. (103), we write the fluorescence intensity as

$$I_F = (Q_A A + Q_B B)\, I_1 \tag{112}$$

where $Q_A = \epsilon_{11} k_{A^*}^F/(k_{-1} + k_B + k_E)$ and $Q_B = \epsilon_{11} k_{B^*}^F/k_{-1}$ are the brightnesses of species A and B, respectively. It is to be noted that the non-vanishing brightness of species B is a direct consequence of the hypothesis on the fluorescent nature of B*.

C.3 Reduced mechanisms valid for four different illuminations

The thermal rate constants k_C, k_{DA}, and k_{EA}, involved in the five-state mechanism have close orders of magnitude and are associated with reaction steps which can be observed within the investigated time range. The light intensities I_1 and I_2 set the value of the photochemical rate constants k_1', k_1'', and k_2'. Consequently the light intensities control which species are sufficiently fast to be eliminated and which reduced mechanism is relevant.

Under illumination I, $I_2 = 0$ which leads to $k_2' = 0$. Hence kinetics does not involve species D nor the rate constants k_2' and k_{DA}. The five-state mechanism becomes a four-state mechanism. In addition, since A is the thermodynamically stable state, the initial condition is assumed to be $A = C_{\text{tot}}$. We reduce then the five-state mechanism for low and high light intensities I_1.

C.3.1 Illumination $\mathbf{I}_{\text{low}}$

We first consider an illumination I_{low} of low light intensity obeying $I_1 \ll \min(k_C/\sigma_{11}, k_{EA}/\sigma_{11}^E)$. According to Supplementary Table 6, this condition is written $I_1 \ll 0.1\text{–}10^2$ Ein.m^{-2}.s^{-1}. The conditions $k_1' \ll k_C$ and $k_1'' \ll k_{EA}$ are then fulfilled enabling the elimination of the fast species B and E. We write $\frac{\mathrm{d}B}{\mathrm{d}t} = 0$ and $\frac{\mathrm{d}E}{\mathrm{d}t} = 0$ where B and E obey Eqs. (105,108). We obtain the two-state mechanism given in Supplementary Figure 57c. Using Eq. (112), we write the fluorescence intensity as

$$I_F = \left(Q_A + Q_B \frac{k_1'}{k_{DA}}\right) A I_1 \tag{113}$$

The fluorescence intensity behaves as the concentration A according to a monoexponential decrease of characteristic time

$$\tau = \frac{1}{k_1'} \tag{114}$$

The characteristic time is inversely proportional to I_1 and a photochemical step controls the fluorescence evolution.

C.3.2 Illumination $\mathrm{I}_{\mathrm{high}}$

Then we consider an illumination $\mathrm{I}_{\mathrm{high}}$ of high light intensity such that the condition $I_1 \gg k_{DA}/(\sigma_{11} + \sigma_{11}^E)$, i.e. $k_{DA} \ll k_1' + k_1''$ is fulfilled. Supplementary Table 6 is used to assess the interval of $I_1 \gg$ 10–10^5 Ein.m^{-2}.s^{-1} in which the condition is fulfilled. In this condition, species A is fast and can be eliminated using the steady-state approximation. We write $\frac{\mathrm{d}A}{\mathrm{d}t} = 0$ where A is given in Eq. (104). We obtain the three-state thermal mechanism shown in Supplementary Figure 57d. Using Eq. (112), we have

$$I_F = \left(Q_A \frac{k_{EA}}{k_1' + k_1''} E + Q_B B \right) I_1 \tag{115}$$

Exactly as the fundamental state of a fluorophore is said to be bright, due to the elimination of the fast evolving singlet excited state, some species in reduced mechanisms may be considered bright. The reduction of the eight-state mechanism results in an apparent brightness for the state E although it is not intrinsically bright.

The fluorescence intensity depends on both E and B which decrease with the two characteristic times

$$\tau_1 = \frac{1}{k_C} \tag{116}$$

$$\tau_2 = \frac{k_1' + k_1''}{k_1' k_{EA}} \tag{117}$$

Hence, for high enough intensities I_1, the experimental fluorescence evolution is at least biexponential and independent of I_1 and thermal steps control the fluorescence evolution. Considering the fluorescence emission from the Trans-O$^-$ is necessary to account for the deviation of the fluorescence emission from a monoexponential.

Regardless of the value of the light intensity I_1, illumination I yields $C = C_{\mathrm{tot}}$ at the steady state. In the case of illumination II, which occurs after illumination $\mathrm{I}_{\mathrm{low}}$, we assume that the steady state is reached when illumination II starts. Consequently we adopt $C = C_{\mathrm{tot}}$ as the initial condition of illumination II. We reduce the five-state mechanism for low and high light intensities I_2.

C.3.3 Illumination $\mathrm{II}_{\mathrm{low}}$

We consider an illumination $\mathrm{II}_{\mathrm{low}}$ of low light intensity I_2 bearing in mind that I_1 is to be fixed around 0.1 ein.m^2.s^{-1} in order to obtain a sufficient level of fluorescence. We assume that the condition $I_2 \ll \min(k_{DA}/\sigma_{22}, k_C/\sigma_{12}, k_{EA}/\sigma_{12}^E)$ is met, so that $k_1' \ll k_C$, $k_1'' \ll k_{EA}$, and $k_2' \ll k_{DA}$. Supplementary Table 6 is used to evaluate the interval of $I_2 \ll$ 1–10^3 Ein.m^{-2}.s^{-1} in which the condition is fulfilled. The two species B and D are fast and can be eliminated. We write $\frac{\mathrm{d}B}{\mathrm{d}t} = 0$ and $\frac{\mathrm{d}D}{\mathrm{d}t} = 0$ where B and D are given in Eqs. (105,107). We obtain the two-state mechanism shown in Supplementary Figure 57e. According to Eq. (113), the fluorescence intensity behaves like the concentration of A, which monoexponentially increases with the characteristic time

$$\tau = \frac{1}{k_1' + k_2'} \tag{118}$$

The characteristic time depends on I_1 and I_2 and photochemical steps control the fluorescence evolution.

C.3.4 Illumination $\mathrm{II}_{\mathrm{high}}$

Finally we consider an illumination $\mathrm{II}_{\mathrm{high}}$ associated with high light intensities I_2 and $I_1 \sim 10$ Ein.m^{-2}.s^{-1}. We suppose that the intensity I_2 obeys $I_2 \gg k_C/\sigma_{22}$, so that $k_C \ll k_2'$. Supplementary Table 6 is used to estimate the

interval of $I_2 \gg 10$–10^5 Ein.m^{-2}.s^{-1} in which the condition is fulfilled. The fast species C is eliminated. We write $\frac{\mathrm{d}C}{\mathrm{d}t} = 0$ where C is given in Eq. (106). We obtain the four-state mechanism shown in Supplementary Figure 57f. The fluorescence intensity is given in Eq. (112).

The dynamics is described by three independent variables. A 3×3 matrix has to be diagonalized and Cardano's method used to obtain the three characteristic times. According to the orders of magnitude given in Supplementary Table 6, the rate constants obey $k_1' + k_1'' + k_{EA} \ll \min(k_C, k_{DA})$. We introduce a condition of comparison between k_C and k_{DA} in order to more easily interpret the results. At the leading order of the expansion in k_{DA}/k_C, we find the characteristic times

$$\tau_1 \simeq \frac{1}{k_C} \tag{119}$$

$$\tau_2 \simeq \frac{1}{k_{DA}} \tag{120}$$

$$\tau_3 \simeq \frac{1}{k_1'' + k_{EA}} \tag{121}$$

Similarly at the leading order of the expansion in k_C/k_{DA} we find

$$\tau_1 \simeq \frac{1}{k_{DA}} \tag{122}$$

$$\tau_2 \simeq \frac{1}{k_C} \tag{123}$$

$$\tau_3 \simeq \frac{1}{k_1' + k_1'' + k_{EA}} \tag{124}$$

In both cases, the characteristic time τ_1 is smaller than τ_2 and τ_3 . Consequently fluorescence evolution is governed at short times by τ_1, then by τ_2, and eventually by τ_3.

Starting from the four-state mechanism given in Supplementary Figure 57f, we look for analytical solutions of the concentrations in the form of the sum of three exponential terms. After analytical resolution of the fluorescence evolution, we find that the amplitude associated with τ_1 is negative and the amplitudes associated with τ_2 and τ_3 are positive. Consequently fluorescence first increases and then decreases.

The shortest time τ_1 characterizing the increase of fluorescence is thermally controlled and does not depend on light intensities. Both photochemical and thermal steps control the decrease of fluorescence. A sufficiently high light intensity I_1 impacts the characteristic time τ_3.

C.4 Non-redundancy of the characteristic times acquired under the four different illuminations

Depending on the value of the light intensities, the reaction model of the RSFPs is reducible into various effective mechanisms. Exploiting the preceding theoretical analysis, the kinetics of RSFPs can be probed using four illumination regimes which grant access to four different dynamics. In the case of illumination $\mathrm{I_{low}}$, the characteristic time depends on light intensity I_1 and in the case of illumination $\mathrm{II_{low}}$, the characteristic time depends on both light intensities I_1 and I_2 which ensures the non redundancy of the two characteristic times at low intensities. At high light intensity I_1 for illumination $\mathrm{I_{high}}$ and I_2 for illumination $\mathrm{II_{high}}$, the characteristic times are imposed by different thermal steps as proven by the two different reduced mechanisms. For illumination $\mathrm{II_{high}}$, at least two characteristic times associated with the increase and decrease of fluorescence can be retrieved from the complex evolution. The characteristic time of the decrease of fluorescence may possibly depend on light intensity I_1 but differently from the characteristic times associated with illuminations $\mathrm{I_{low}}$ and $\mathrm{II_{low}}$. Hence the four illumination regimes used in LIGHTNING provide non redundant kinetic information on RSFPs.

C.5 Comparison of the experiments with the predictions of the reduced mechanisms of RSFPs

We end up this section by presenting experimental observations, which support the relevance of the reduction of the eight-state mechanism in the 1 μs–1 s time window in solution and 1 ms–1 s time window in bacteria. According to the predictions of the three-state (four-state, resp.) mechanism associated with illumination $\mathrm{I_{high}}$ ($\mathrm{II_{high}}$, resp.), two (three, resp.) characteristic times are necessary to account for the kinetics of RSFPs. Nevertheless, the data processing introduced in sections B.1 and B.2 satisfactorily associates a single characteristic time to each monotonous part of fluorescence evolution.

The fluorescence evolution from solutions of the RSFPs under various illuminations has been recorded during 0.4 s at best with a sampling interval of 10^{-6} s at best. As a consequence, only reaction steps associated with characteristic times larger than 1 μs and smaller than 1 s can be observed. In the case of illumination $\mathrm{II_{high}}$, only the short characteristic time associated with the fast fluorescence increase is extracted to reduce the acquisition time. The fluorescence evolutions of all 20 studied negative RSFPs exhibit a qualitatively similar behavior:

- Supplementary Figure 21-Supplementary Figure 42e,f show that the inverse of the characteristic time is proportional to light intensity in the regimes of low intensities. The two-state mechanisms obtained in sections C.3.1 and C.3.3 correctly predict a monoexponential behavior for which the inverse of the characteristic time linearly increases with light intensity. Kinetics is controlled by the photochemical steps in the low intensity regimes. Fluorescence experiments give access to the values of the isomerization cross sections for all investigated RSFPs. As displayed in Supplementary Table 3, the results agree with the orders of magnitude of the values tabulated in the literature.

- In the case of illuminations $\mathrm{I_{high}}$ and $\mathrm{II_{high}}$ the monoexponential approach illustrated in Supplementary Figure 21-Supplementary Figure 42c,d leads to characteristic times nearly independent of light intensity in the range of high experimentally accessible intensities.

- The three-state mechanism obtained in section C.3.2 is thermally controlled. The two characteristic times do not depend on the light intensity I_1. The fitting to the fluorescence decays reveals a departure from a monoexponential behavior in agreement with the complexity of the reduced three-state mechanism. As displayed in Supplementary Table 3, the characteristic time deduced from the monoexponential fitting is in the order of magnitude of the characteristic time of the thermal step associated with protonation which supports the idea that the Trans-$\mathrm{O^-}$ state is fluorescent.

- The fluorescence evolution under illumination $\mathrm{II_{high}}$ is not monotonous in the investigated time window. A rapid increase of large amplitude is followed by a slower decrease of smaller amplitude leading to the existence of a maximum. The four-state mechanism obtained in section C.3.4 correctly accounts for the experimental results. The characteristic time associated with the fluorescence increase and given in Supplementary Table 3 is mainly controlled by the thermal proton exchange step. The fluorescence decrease has not been analyzed in detail.

The fluorescence evolution from images of 16 RSFP-labeled bacteria under various illuminations has been recorded during 0.8 s with a sampling interval of 10^{-3} s. As a consequence, only reaction steps associated with characteristic times larger than 1 ms and smaller than 1 s can be observed. The characteristic times are displayed in Supplementary Table 13.

- The fluorescence evolution observed for illumination $\mathrm{I_{low}}$ is similar to the corresponding evolution in solution.

- In the case of illumination I_{high}, the large sampling interval leads to the determination of a different characteristic time than in solution, closer to the longest characteristic time of the reduced three-state mechanism.
- In the case of illumination II_{high}, the short characteristic time of the fluorescence increase is not accessible in the investigated 1 ms–1 s time window. The analysis of the fluorescence decrease yields a characteristic time in agreement with the longest characteristic time of the reduced four-state mechanism.

To conclude, the eight-state mechanism and the associated reduced schemes in the four illumination regimes displayed in Supplementary Figure 57 provide a satisfying description of the experimental fluorescence evolution of illuminated RSFPs in the investigated time window both in solution and bacteria.

D LIGHTNING discriminatory power

This section reports on the evaluation of the kinetic fingerprint of the RSFPs, the minimal distance among sets of RSFPs, and the cutoff distance d_c, which are reported in the Main Text .

D.1 On solutions of RSFPs

We first address the experiments on RSFPs solution, which have been made with the photoswitchometer configuration devoted to acquire the kinetic information on RSFP photoswitching.

D.1.1 LIGHTNING kinetic fingerprint of RSFPs

Supplementary Table 7 sums up the $\{\tau^{\mathrm{I}}_{low}, \tau^{\mathrm{II}}_{low}, \tau^{\mathrm{I}}_{high}, \tau^{\mathrm{II}}_{high}\}$ sets, which have been obtained with the photoswitchometer in the configuration displayed in Supplementary Figure 1 for the 22 investigated RSFPs at 10 μM concentrations.

Supplementary Table 7: *Kinetic fingerprints* $\{\tau^{\mathrm{I}}_{low}, \tau^{\mathrm{II}}_{low}, \tau^{\mathrm{I}}_{high}, \tau^{\mathrm{II}}_{high}\}$ *of the 22 RSFPs in solution.* The characteristic times τ^{I}_{low} and τ^{II}_{low} have been measured with the photoswitchometer in the configuration displayed in Supplementary Figure 1a in the regime of low light intensity with illumination I_{low}: $I_1 = 2$ ein.m^{-2}.s^{-1} and illumination II_{low}: $I_1 = 0.1$ ein.m^{-2}.s^{-1} and $I_2 = 0.1$ ein.m^{-2}.s^{-1} whereas the characteristic times τ^{I}_{high} and $\tau^{\mathrm{II}}_{high}$ were obtained at the highest light intensities accessible with the photoswitchometer in the regime of high light intensity with illumination I_{high}: $I_1 = 200$ ein.m^{-2}.s^{-1}; illumination II_{high}: $I_1 = 2$ ein.m^{-2}.s^{-1} and $I_2 = 90$ ein.m^{-2}.s^{-1}. Solvent: pH=7.4 PBS (50 mM PBS, 150 mM NaCl). T = 298 K.

RSFP	τ^{I}_{low}	τ^{II}_{low}	τ^{I}_{high}	$\tau^{\mathrm{II}}_{high}$
	(s)	(s)	(s)	(s)
**1**	$(1.4\pm0.3)\times10^{-1}$	$(3.15\pm0.01)\times10^{-2}$	$(4.52\pm0.09)\times10^{-3}$	$(1.83\pm0.04)\times10^{-4}$
**2**	$(2.2\pm0.1)\times10^{-3}$	$(2.53\pm0.05)\times10^{-2}$	$(3.34\pm0.01)\times10^{-4}$	$(7.73\pm0.02)\times10^{-5}$
**3**	$(2.57\pm0.05)\times10^{-2}$	$(2.4\pm0.03)\times10^{-2}$	$(4.84\pm0.08)\times10^{-3}$	$(1.25\pm0.03)\times10^{-4}$
**4**	$(1.12\pm0.08)\times10^{-2}$	$(4.26\pm0.03)\times10^{-2}$	$(2.56\pm0.07)\times10^{-3}$	$(1.13\pm0.02)\times10^{-2}$
**5**	$(1.42\pm0.01)\times10^{-2}$	$(1.23\pm0.02)\times10^{-1}$	$(3.57\pm0.08)\times10^{-3}$	$(9.25\pm0.02)\times10^{-3}$
**6**	$(5.1\pm0.3)\times10^{-3}$	$(1.67\pm0.01)\times10^{-2}$	$(1.17\pm0.05)\times10^{-4}$	$(2.14\pm0.02)\times10^{-4}$
**7**	$(1.7\pm0.4)\times10^{-2}$	$(1.84\pm0.02)\times10^{-2}$	$(5.44\pm0.02)\times10^{-4}$	$(6.74\pm0.03)\times10^{-5}$
**8**	$(2.11\pm0.08)\times10^{-2}$	$(1.61\pm0.04)\times10^{-2}$	$(4.23\pm0.03)\times10^{-3}$	$(9.5\pm0.1)\times10^{-5}$
**9**	$(5.8\pm0.1)\times10^{-1}$	$(1.84\pm0.02)\times10^{-2}$	$(5.95\pm0.02)\times10^{-3}$	$(1.25\pm0.02)\times10^{-4}$
**10**	$(2.53\pm0.15)\times10^{-2}$	$(2.14\pm0.02)\times10^{-2}$	$(6.8\pm0.1)\times10^{-4}$	$(7.75\pm0.03)\times10^{-5}$
**11**	$(3.16\pm0.03)\times10^{-2}$	$(2.23\pm0.03)\times10^{-2}$	$(3.82\pm0.07)\times10^{-3}$	$(1.12\pm0.01)\times10^{-4}$
**12**	$(8.6\pm0.1)\times10^{-3}$	$(1.46\pm0.05)\times10^{-2}$	$(3.1\pm0.2)\times10^{-4}$	$(6.23\pm0.02)\times10^{-5}$
**13**	$(7.1\pm0.4)\times10^{-2}$	$(2.66\pm0.03)\times10^{-2}$	$(5.63\pm0.02)\times10^{-3}$	$(1.75\pm0.03)\times10^{-4}$
**14**	$(1.36\pm0.05)\times10^{-1}$	$(2.84\pm0.01)\times10^{-2}$	$(2.92\pm0.05)\times10^{-3}$	$(1.84\pm0.02)\times10^{-4}$
**15**	$(4.2\pm0.1)\times10^{-3}$	$(2.61\pm0.03)\times10^{-2}$	$(3.14\pm0.02)\times10^{-4}$	$(3.64\pm0.03)\times10^{-4}$
**16**	$(4.2\pm0.1)\times10^{-3}$	$(1.82\pm0.06)\times10^{-2}$	$(6.25\pm0.04)\times10^{-4}$	$(9.83\pm0.01)\times10^{-5}$
**17**	$(2.45\pm0.03)\times10^{-3}$	$(2.55\pm0.01)\times10^{-2}$	$(4.26\pm0.03)\times10^{-4}$	$(1.27\pm0.02)\times10^{-4}$
**18**	$(1.37\pm0.07)\times10^{-1}$	$(2.14\pm0.04)\times10^{-2}$	$(3.82\pm0.01)\times10^{-3}$	$(3.82\pm0.06)\times10^{-4}$
**19**	$(2.6\pm0.1)\times10^{-3}$	$(7.64\pm0.03)\times10^{-3}$	$(1.21\pm0.03)\times10^{-4}$	$(3.15\pm0.04)\times10^{-5}$
**20**	$(3.6\pm0.1)\times10^{-3}$	$(9.52\pm0.02)\times10^{-3}$	$(1.31\pm0.07)\times10^{-4}$	$(1.35\pm0.04)\times10^{-4}$
**21**	$(3.25\pm0.02)\times10^{-4}$	$(8.77\pm0.02)\times10^{-3}$	$(2.15\pm0.07)\times10^{-4}$	$(1.73\pm0.01)\times10^{-4}$
**22**	$(2.7\pm0.1)\times10^{-3}$	$(7.45\pm0.01)\times10^{-3}$	$(1.36\pm0.08)\times10^{-4}$	$(6.17\pm0.02)\times10^{-5}$

D.1.2 LIGHTNING minimal distance among sets of RSFPs

The preceding results have been exploited to choose and rank RSFPs within the set of the $N = 22$ studied RSFPs by implementing the sorting algorithms reported in paragraph 1.7.1. The optimized subsets $s'(m)$ and $s(m)$ of m RSFPs for $m = 2, ..., N$ subsets are given in Supplementary Table 8.

Supplementary Table 8: Optimized subsets $s'(m)$ and $s(m)$ of m RSFPs for $m = 2, ..., 22$ using the kinetic signature given in Supplementary Table 7 and the maximum value of the minimum distance between pairs.

m	$s'(m)$	$s(m)$
2	(**5**,**19**)	(**5**,**19**)
3	(**4**,**9**,**19**)	**9**
4	(**4**,**6**,**8**,**9**)	**10**
5	(**3**,**4**,**9**,**15**,**19**)	**15**
6	(**3**,**4**,**7**,**9**,**15**,**19**)	**18**
7	(**4**,**6**,**7**,**8**,**9**,**18**,**19**)	**8**
8	(**3**,**4**,**6**,**9**,**10**,**16**,**18**,**19**)	**2**
9	(**1**,**2**,**3**,**4**,**7**,**9**,**15**,**19**,**20**)	**12**
10	(**1**,**2**,**3**,**4**,**9**,**10**,**12**,**15**,**19**,**20**)	**21**
11	(**1**,**2**,**3**,**4**,**5**,**6**,**9**,**10**,**12**,**15**,**22**)	**4**
12	(**2**,**4**,**5**,**6**,**8**,**9**,**10**,**12**,**13**,**15**,**18**,**22**)	**13**
13	(**2**,**3**,**4**,**5**,**6**,**9**,**10**,**12**,**13**,**15**,**18**,**21**,**22**)	**6**
14	(**2**,**3**,**4**,**5**,**6**,**9**,**10**,**12**,**13**,**15**,**16**,**18**,**21**,**22**)	**16**
15	(**2**,**3**,**4**,**5**,**6**,**9**,**10**,**12**,**13**,**14**,**15**,**16**,**18**,**21**,**22**)	**14**
16	(**1**,**2**,**3**,**4**,**5**,**6**,**9**,**10**,**12**,**13**,**15**,**16**,**18**,**19**,**21**,**22**)	**22**
17	(**1**,**2**,**3**,**4**,**5**,**6**,**9**,**10**,**12**,**13**,**15**,**16**,**18**,**19**,**20**,**21**,**22**)	**20**
18	(**1**,**2**,**3**,**4**,**5**,**6**,**8**,**9**,**10**,**12**,**13**,**15**,**16**,**18**,**19**,**20**,**21**,**22**)	**3**
19	(**1**,**2**,**3**,**4**,**5**,**6**,**8**,**9**,**10**,**12**,**13**,**14**,**15**,**16**,**18**,**19**,**20**,**21**,**22**)	**1**
20	(**1**,**2**,**3**,**4**,**5**,**6**,**7**,**8**,**9**,**10**,**12**,**13**,**14**,**15**,**16**,**18**,**19**,**20**,**21**,**22**)	**7**
21	(**1**,**2**,**3**,**4**,**5**,**6**,**7**,**8**,**9**,**10**,**12**,**13**,**14**,**15**,**16**,**17**,**18**,**19**,**20**,**21**,**22**)	**17**
22	(**1**,**2**,**3**,**4**,**5**,**6**,**7**,**8**,**9**,**10**,**11**,**12**,**13**,**14**,**15**,**16**,**17**,**18**,**19**,**20**,**21**,**22**)	**11**

As shown in Supplementary Table 8, the relation $s'(m) \subset s'(m+1)$ is not necessarily observed for $s'(m)$ and $s'(m+1)$. This observation has motivated to introduce the alternative sorting algorithm including three-body interactions, which does not suffer from this limitation. The two sorting algorithms lead to similar results even if the relation $s(m) \subset s(m+1)$ is not necessarily observed for $s'(m)$ and $s'(m+1)$. The set $s(m)$ is obtained from $s'(m)$ by replacing some RSFs with close ones.

In a purpose of comparison with the results we previously obtained with Speed OPIOM,[7] we use as well the sorting algorithms to choose and rank RSFPs within the reduced set of the $N = 7$ RSFPs **1**–**7**. The optimized subsets $s'(m)$ and $s(m)$ of m RSFPs for $m = 2, ..., N$ subsets are given in Supplementary Table 9.

The comparison of the data provided in Supplementary Table 8 and Supplementary Table 9 shows that the ranking of the RSFPs may be slightly affected by the removal of an RSFP in the initial set.

D.1.3 LIGHTNING cutoff distance d_c

d_c evaluation The paragraph 1.7.2 has shown that the cutoff distance d_c depends on the maximum uncertainty M of the experimental measurements. d_c has been evaluated with the photoswitchometer in the optical configuration dis-

Supplementary Table 9: Optimized subsets $s'(m)$ and $s(m)$ of m RSFPs for $m = 2, ..., 7$ using the kinetic signature given in Supplementary Table 7 and the maximum value of the minimum distance between pairs.

m	$s'(m)$	$s(m)$
2	(**2**,**5**)	(**5**,**6**)
3	(**1**,**2**,**4**)	**3**
4	(**3**,**5**,**6**,**7**)	**1**
5	(**1**,**3**,**5**,**6**,**7**)	**7**
6	(**1**,**2**,**3**,**4**,**6**,**7**)	**2**
7	(**1**,**2**,**3**,**4**,**5**,**6**,**7**)	**4**

played in Supplementary Figure 1 by recording with the photomultiplier the time series of the fluorescence originating from a cylindrical volume (80 μm-high and 10 μm-radius) of 15–20 μM RSFP solutions upon illuminations I_{low}, II_{low}, I_{high}, and II_{high}. Fluorescence evolution has been recorded 500 times for each discriminative dimension k for the RSFPs i =**2**,**6**, which is sufficient to reliably estimate the mean and the standard deviation of the distribution of the characteristic times. The characteristic times have been extracted using the spectrum approach and a monoexponential reduction. The corresponding distributions of l_{ki} are given in Supplementary Figure 58 and Supplementary Figure 59.

Both methods provide similar mean values $\langle l_{ki} \rangle$ and standard deviations σ_{ki} of the distributions as shown in Supplementary Table 10 and Supplementary Table 11. When the mean characteristic time is smaller than ten times the sampling time of the fluorescence evolution, the standard deviations deduced from the spectra are slightly larger than the values obtained using the monoexponential reduction.

Supplementary Table 10: RSFP **2**: Properties of the distribution functions of the decimal logarithm of the characteristic times deduced from two methods of data processing. The mean value $\langle l_{k\mathbf{2}} \rangle$ of the decimal logarithm of the characteristic time and the standard deviation $\sigma_{k\mathbf{2}}$ have been computed using 500 experiments by recording with the photomultiplier the changes of the fluorescence signal originating from a cylindrical volume (80 μm-high and 10 μm-radius) of 15 μM solution of RSFP **2** in pH 7.4 PBS (50 mM sodium phosphate, 150 mM NaCl) for the four discrimination dimensions $k = 1, 2, 3, 4$. The characteristic times are expressed in seconds. The values of the light intensities are given in the caption of Supplementary Figure 58.

	Spectrum		Monoexponential reduction	
	$\langle l_{k\mathbf{2}} \rangle$	$\sigma_{k\mathbf{2}}$	$\langle l_{k\mathbf{2}} \rangle$	$\sigma_{k\mathbf{2}}$
$l_{1\mathbf{2}}$	-1.4454	0.0050	-1.4343	0.0065
$l_{2\mathbf{2}}$	-1.8806	0.0063	-1.8693	0.0035
$l_{3\mathbf{2}}$	-3.4977	0.0652	-3.4910	0.0677
$l_{4\mathbf{2}}$	-4.2090	0.1534	-4.2851	0.0267

Supplementary Table 11: Same caption as in Supplementary Table 10 for 20 μM solution of RSFP **6**. The values of the light intensities are given in the caption of Supplementary Figure 59.

	Spectrum		Monoexponential reduction	
	$\langle l_{k\mathbf{6}} \rangle$	$\sigma_{k\mathbf{6}}$	$\langle l_{k\mathbf{6}} \rangle$	$\sigma_{k\mathbf{6}}$
$l_{1\mathbf{6}}$	-1.2905	0.0055	-1.2844	0.0016
$l_{2\mathbf{6}}$	-2.1295	0.0906	-2.1862	0.0066
$l_{3\mathbf{6}}$	-3.9803	0.0385	-3.9595	0.0286
$l_{4\mathbf{6}}$	-3.3176	0.0500	-3.2987	0.0254

For illuminations I_{low} and II_{low} and the two investigated RSFPs i =**2**,**6**, we found $\sigma_{1i} \simeq \sigma_{2i} \simeq 0.007$ whereas $\sigma_{3i} \simeq \sigma_{4i} \simeq 0.05$ for illuminations I_{high} and II_{high}. Based on the order of magnitude of the standard deviations of the distributions of l_{ki}, we assigned the value $M = 0.05$ to the uncertainty. Then we used Eq.(19) to derive $d_c = 0.2$ since we used $n = 4$ in the case of solutions of RSFPs.

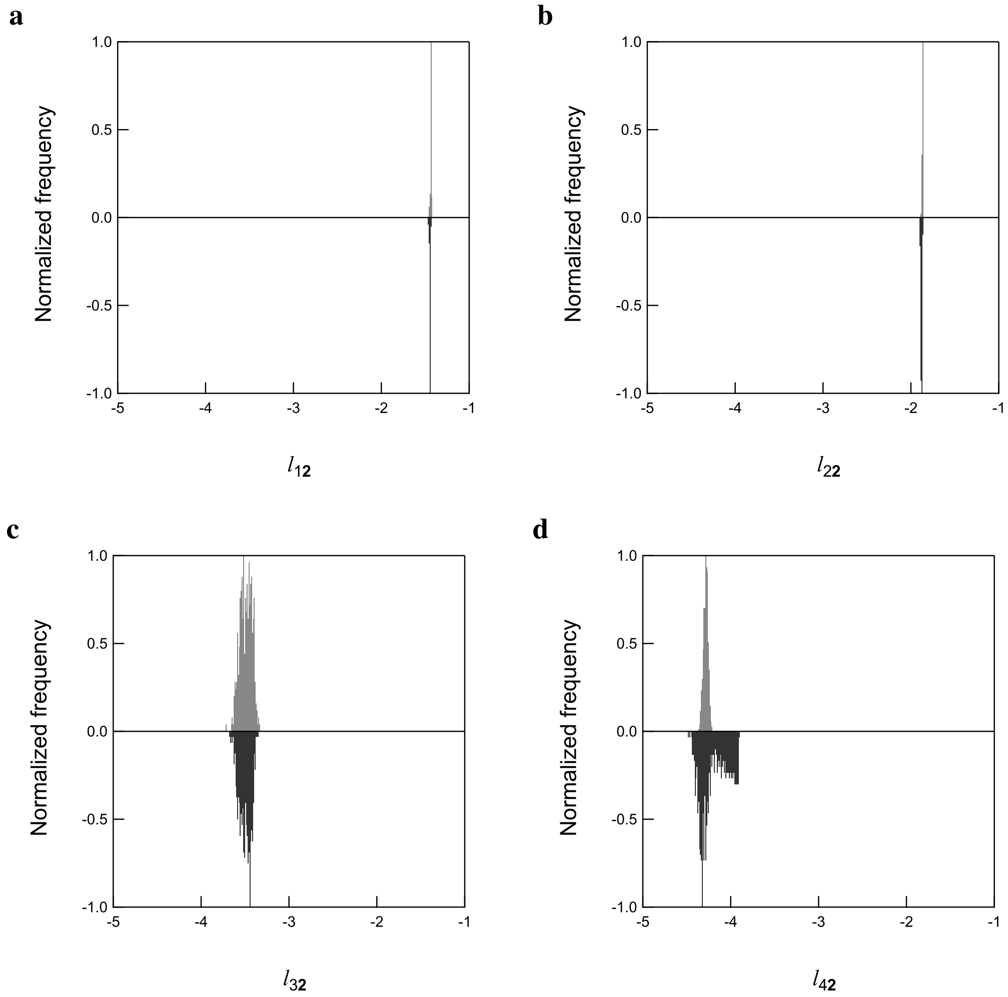

Supplementary Figure 58: Histograms of the decimal logarithm of the characteristic times l_{12} (**a**, $I_1 = 0.16$ ein.m^{-2}.s^{-1}), l_{22} (**b**, $I_1 = 0.18$ ein.m^{-2}.s^{-1}, $I_2 = 0.18$ ein.m^{-2}.s^{-1}), l_{32} (**c**, $I_1 = 228.8$ ein.m^{-2}.s^{-1}), and l_{42} (**d**, $I_1 = 0.275$ ein.m^{-2}.s^{-1}, $I_2 = 100.7$ ein.m^{-2}.s^{-1}) of RSFP **2** deduced from 500 experiments by recording with the photomultiplier the changes of the fluorescence signal originating from a cylindrical volume (80 μm-high and 10 μm-radius) of 15 μM solution of RSFP **2** in pH 7.4 PBS (50 mM sodium phosphate, 150 mM NaCl) for each discriminative dimension using the spectrum approach (black, down) and a monoexponential reduction (grey, up). The characteristic times are expressed in seconds.

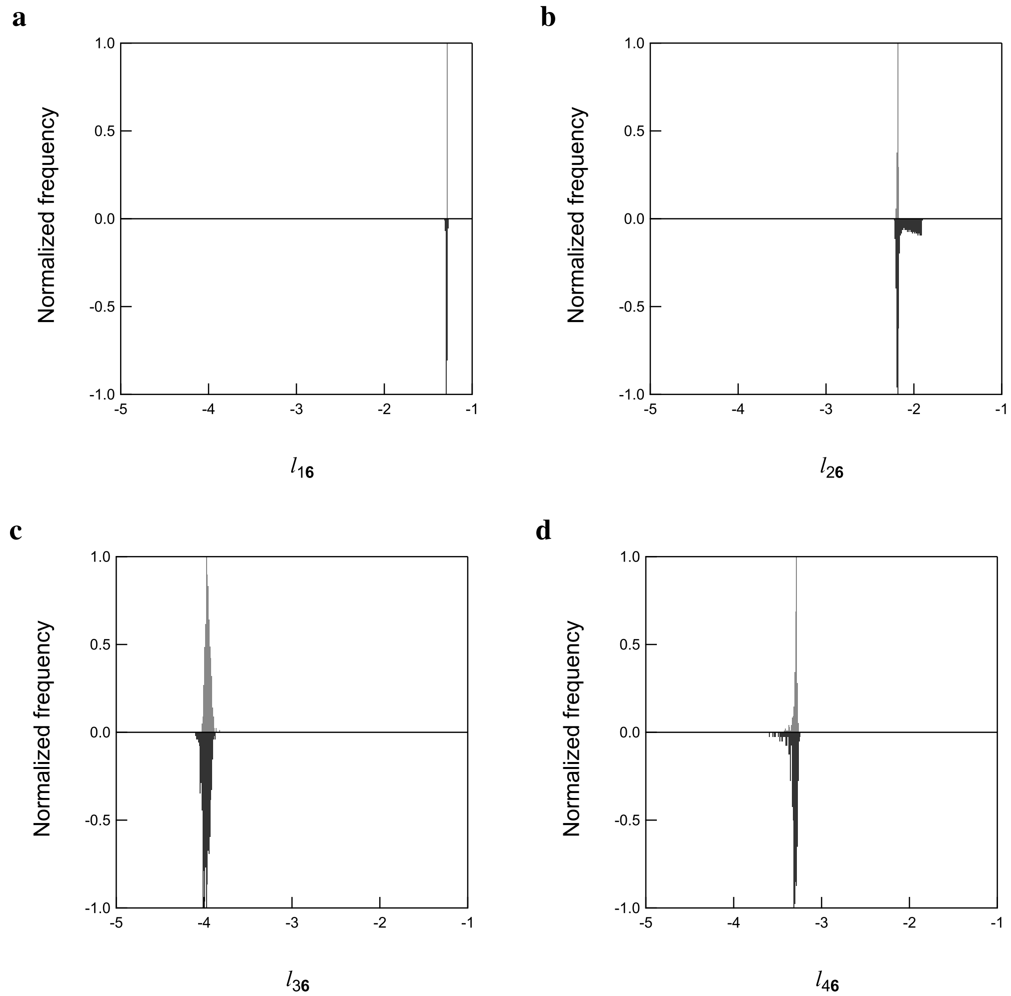

Supplementary Figure 59: Histograms of the decimal logarithm of the characteristic times $l_{\mathbf{16}}$ (**a**, $I_1 = 0.18$ ein.m^{-2}.s^{-1}), $l_{\mathbf{26}}$ (**b**, $I_1 = 0.18$ ein.m^{-2}.s^{-1}, $I_2 = 0.18$ ein.m^{-2}.s^{-1}), $l_{\mathbf{36}}$ (**c**, $I_1 = 249.6$ ein.m^{-2}.s^{-1}), and $l_{\mathbf{46}}$ (**d**, $I_1 = 0.175$ ein.m^{-2}.s^{-1}, $I_2 = 95.4$ ein.m^{-2}.s^{-1}) of RSFP **6** deduced from 500 experiments by recording with the photomultiplier the changes of the fluorescence signal originating from a cylindrical volume (80 μm-high and 10 μm-radius) of 20 μM solution of RSFP **6** in pH 7.4 PBS (50 mM sodium phosphate, 150 mM NaCl) for each discriminative dimension using the spectrum approach (black, down) and a monoexponential reduction (grey, up). The characteristic times are expressed in seconds.

Discussion Under the present experimental conditions involving a large number of RSFP molecules in a same environment contained in the observed volume (so as to give rise to an excellent signal-to-noise-ratio) as well as the high frequency of the acquisition of the fluorescence signal (so as to provide many data points), we questioned whether the standard deviations of the distributions $l_{k\mathbf{i}}$ reported in Supplementary Table 10 and Supplementary Table 11 could originate from some instabilities of our instrumental setup. To evaluate the latter possibility, we collected a fraction of the light from the LEDs at 480 and 405 nm, and from the lasers at 488 and 405 nm and measured their intensity over 500 successive acquisitions (which is sufficient to reliably estimate the mean and the standard deviation of the distribution of intensity) under the conditions, which had been used to build the histograms in Supplementary Figure 58 and Supplementary Figure 59. Supplementary Figure 60 and Supplementary Figure 61 display the results.

Supplementary Figure 60a,b and Supplementary Figure 61a,b demonstrate that light intensity at the focal plane encounters evolution over the acquisition window, which had been used to build the histograms in Supplementary

Figure 58 and Supplementary Figure 59. The observed drift is more pronounced with the lasers than with the LEDs, which interestingly correlates with the higher standard deviations observed for the distributions of $l_{3\mathbf{i}}$ and $l_{4\mathbf{i}}$ than for the distributions of $l_{1\mathbf{i}}$ and $l_{2\mathbf{i}}$ (see the data extracted from monoexponential reduction in Supplementary Table 10 and Supplementary Table 11). To quantitatively evaluate this correlation, we computed the average values and the standard deviations observed for the distributions of the decimal logarithm of the light intensity of the LEDs and the lasers displayed in Supplementary Figure 60c,d and Supplementary Figure 61c,d. The results are shown in Supplementary Table 12.

Supplementary Table 12: Average values $< I >$ and standard deviations σ_I observed for the distributions of the decimal logarithm of the light intensity of the LEDs and the lasers displayed in Supplementary Figure 60c,d and Supplementary Figure 61c,d. The light intensities have been experimentally extracted in Volt and are in arbitrary units.

Light source	$< I >$	σ_I
LED 480 nm	-2.3072	0.0035
LED 405 nm	-2.1461	0.0023
Laser 480 nm	-0.8301	0.0115
Laser 405 nm	-1.6386	0.0224

The standard deviations of the distributions of the decimal logarithm of the light intensity of the LEDs and the lasers are in fair agreement with the standard deviations extracted from the distribution functions of the decimal logarithm of the characteristic times for **2** and **6** (see the data extracted from monoexponential reduction in Supplementary Table 10 and Supplementary Table 11). This observation led us to conclude that the $M = 0.05$ uncertainty value extracted from our measurements (and correspondingly the $d_c = 0.2$ value) is an upper value, which may be limited by our instrumental setup.

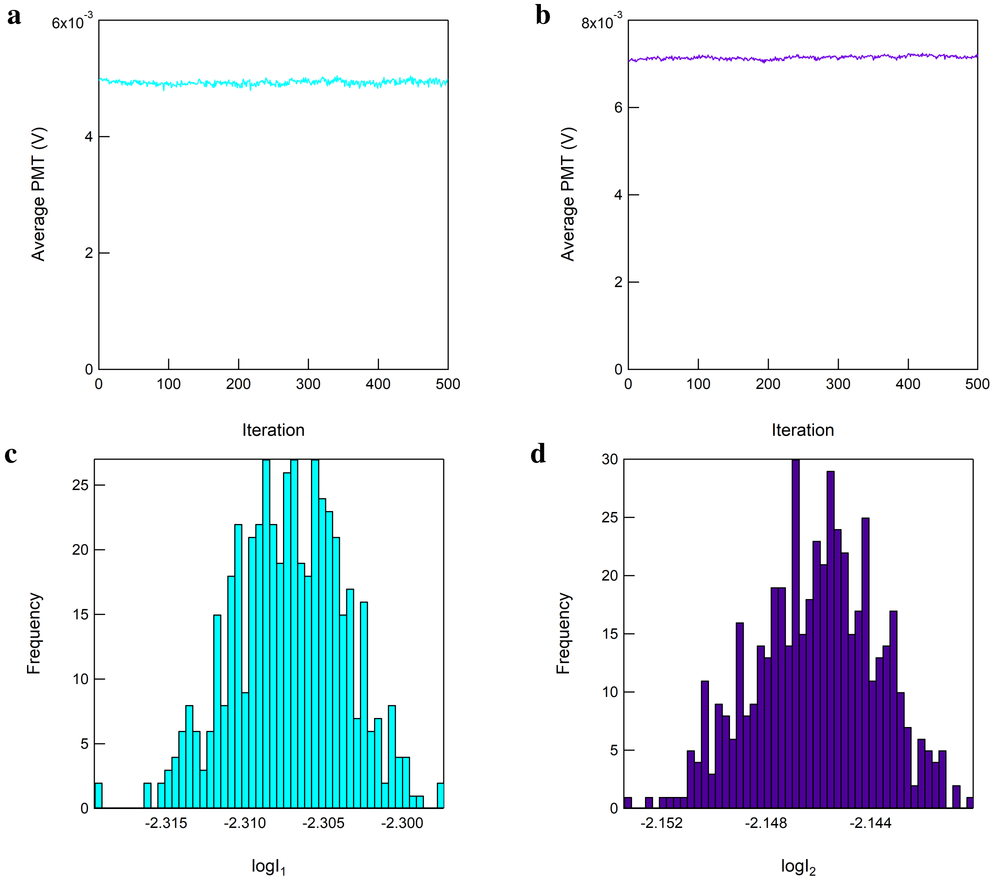

Supplementary Figure 60: *Characterization of the stability of the LED light sources over 500 successive acquisitions*. **a**,**b**: Evolution of the light intensity of the LEDs at 480 (**a**) and 405 (**b**) nm with the number of acquisitions; **c**,**d**: Histograms of the decimal logarithm of the light intensity of the LEDs at 480 (**c**) and 405 (**d**) nm.

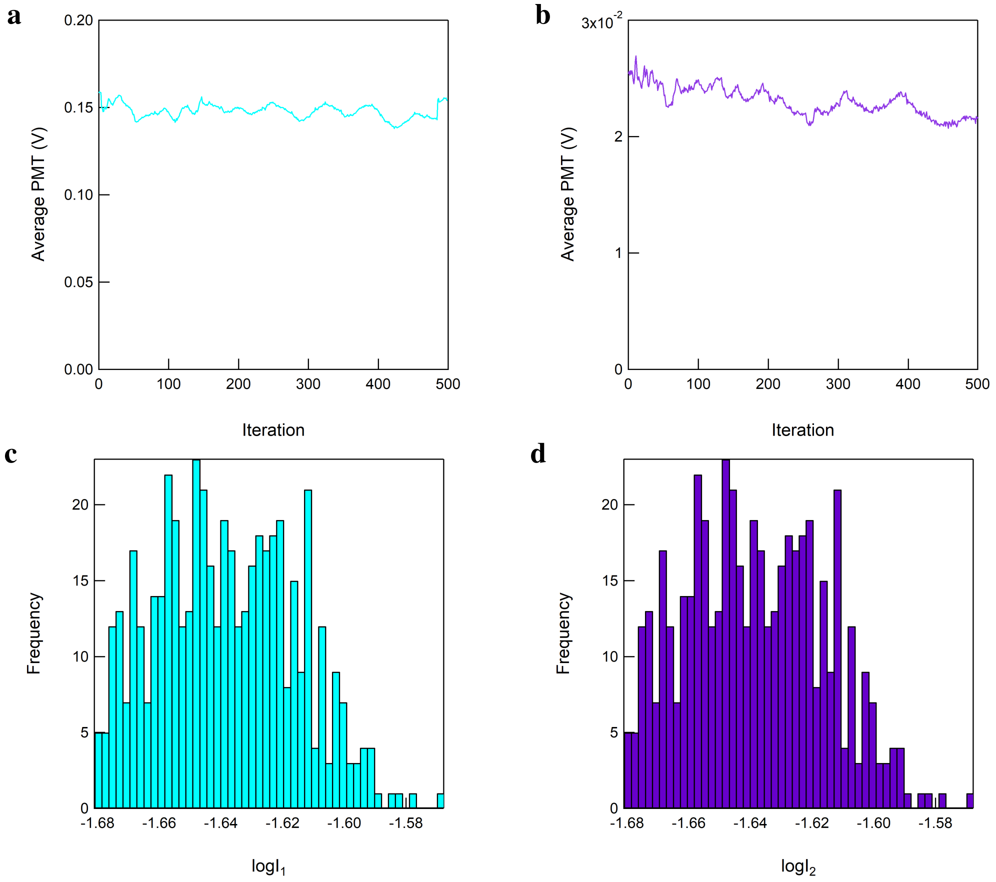

Supplementary Figure 61: *Characterization of the stability of the laser light sources over 500 successive acquisitions*. **a**,**b**: Evolution of the light intensity of the lasers at 488 (**a**) and 405 (**b**) nm with the number of acquisitions; **c**,**d**: Histograms of the decimal logarithm of the light intensity of the lasers at 488 (**c**) and 405 (**d**) nm.

D.2 On images of RSFP-labeled bacteria

We then address the experiments on RSFP-labeled bacteria, which have been made with the photoswitchometer configuration devoted to LIGHTNING image acquisition.

D.2.1 LIGHTNING kinetic fingerprint of RSFP-labeled bacteria

The collection of the RSFPs has been restricted to implement LIGHTNING imaging on RSFP-labeled bacteria. Among the 22 investigated RSFPs, we first eliminated the RSFPs **4** and **5**, which provide weak amplitudes of fluorescence changes under the regimes of strong lights (see Supplementary Figure 24b and Supplementary Figure 25b) and exhibit positive photochromism so as to facilitate their discrimination by dynamic contrast.[7] Then we further discarded the RSFPs **2**, **7**, **16**, and **21**, which were found to be associated with low expression level and/or too short time response of the fluorescence signal to fulfil the selection criteria given in Eqs. (9–11) in bacteria samples.

The images of RSFP-labeled bacteria have been processed as described in subsection 1.6. The illumination II_{low} yielded non exploitable, similar characteristic times for all RSFPs. This result is in line with the prediction of the two-state reduced chemical scheme given in section C.3.3: the characteristic times τ_{low}^{II} have the same order of magnitude as the collecting time $t_c = 10^{-3}$ s of the camera and are too small to be correctly retrieved from the fluorescence evolution recorded by the camera. Consequently, the LIGHTNING kinetic fingerprint of RSFP-labeled bacteria was

reduced to the $\{l^{\mathrm{I}}_{low,\mathbf{i}}, l^{\mathrm{I}}_{high,\mathbf{i}}, l^{\mathrm{II}}_{high,\mathbf{i}}\}$ set. Supplementary Table 13 sums up the kinetic fingerprints of the 16 RSFP-labeled bacteria, which have been obtained with the photoswitchometer in the LIGHTNING imaging configuration.

Supplementary Table 13: *Kinetic fingerprints* $\{\tau^{\mathrm{I}}_{low}, \tau^{\mathrm{I}}_{high}, \tau^{\mathrm{II}}_{high}\}$ *of the 16 RSFP-labeled bacteria.* The characteristic times have been measured with the photoswitchometer in the configuration displayed in Supplementary Figure 1b. Illumination I_{low}: $I_1 = 2$ ein.m^{-2}.s^{-1}; illumination I_{high}: $I_1 = 50$ ein.m^{-2}.s^{-1}; illumination II_{high}: $I_1 = 50$ ein.m^{-2}.s^{-1} and $I_2 = 20$ ein.m^{-2}.s^{-1}. T = 298 K. The values associated with RSFP i are deduced from a statistics on all imaged RSFP-labeled bacteria of type i.

RSFP	τ^{I}_{low} (s)	τ^{I}_{high} (s)	$\tau^{\mathrm{II}}_{high}$ (s)
**1**	$(1.1\pm0.3)\times10^{-1}$	$(1.6\pm0.3)\times10^{-2}$	$(3.4\pm0.6)\times10^{-2}$
**3**	$(1.7\pm0.5)\times10^{-2}$	$(2.7\pm1.5)\times10^{-3}$	$(2.8\pm0.5)\times10^{-2}$
**6**	$(6.3\pm1.6)\times10^{-2}$	$(2.5\pm0.2)\times10^{-2}$	$(4.1\pm0.7)\times10^{-2}$
**8**	$(9.3\pm1.8)\times10^{-2}$	$(8\pm2)\times10^{-3}$	$(2.2\pm0.5)\times10^{-2}$
**9**	$(1.2\pm0.3)\times10^{-1}$	$(1.4\pm0.4)\times10^{-2}$	$(3.0\pm0.9)\times10^{-2}$
**10**	$(3.7\pm0.8)\times10^{-2}$	$(4\pm1)\times10^{-3}$	$(2.1\pm0.3)\times10^{-2}$
**11**	$(4.1\pm0.9)\times10^{-2}$	$(5\pm1)\times10^{-3}$	$(1.5\pm0.3)\times10^{-2}$
**12**	$(1.2\pm0.5)\times10^{-2}$	$(3.2\pm1.5)\times10^{-3}$	$(3\pm1)\times10^{-2}$
**13**	$(1.8\pm0.4)\times10^{-1}$	$(2.2\pm0.4)\times10^{-2}$	$(2.9\pm0.5)\times10^{-2}$
**14**	$(1.0\pm0.2)\times10^{-1}$	$(1.0\pm0.2)\times10^{-2}$	$(1.8\pm0.4)\times10^{-2}$
**15**	$(1.0\pm0.3)\times10^{-2}$	$(6\pm3)\times10^{-3}$	$(4\pm1)\times10^{-2}$
**17**	$(3\pm1)\times10^{-2}$	$(1.0\pm0.3)\times10^{-2}$	$(5.1\pm0.8)\times10^{-2}$
**18**	$(9\pm2)\times10^{-2}$	$(9\pm2)\times10^{-3}$	$(3.2\pm2.8)\times10^{-2}$
**19**	$(1.1 \pm 0.3)\times10^{-2}$	$(4\pm1)\times10^{-3}$	$(7\pm1)\times10^{-2}$
**20**	$(6\pm2)\times10^{-3}$	$(3.0\pm0.9)\times10^{-3}$	$(9\pm2)\times10^{-2}$
**22**	$(4\pm1)\times10^{-3}$	$(2.4\pm0.9)\times10^{-3}$	$(7\pm1)\times10^{-2}$

D.2.2 LIGHTNING sorting of an optimized set of RSFP-labeled bacteria

The variances and the covariances of the kinetic fingerprint $\{l_{1\mathbf{i}}, l_{3\mathbf{i}}, l_{4\mathbf{i}}\}$ associated with the characteristic times $\{\tau^{\mathrm{I}}_{low,i}, \tau^{\mathrm{I}}_{high,i}, \tau^{\mathrm{II}}_{high,i}\}$ of RSFP i-labeled bacteria are given in Supplementary Table 14.

According to Supplementary Table 14, the typical value $M = 0.08$ is assigned to the uncertainty on the decimal logarithm of the characteristic times. Then Eq.(19) is used to derive $d_c = 0.28$ for $n = 3$. As displayed in Supplementary Figure 5, the standard deviations of the distributions of the decimal logarithm of light intensity at $\lambda_1 = 488$ nm and $\lambda_2 = 405$ nm have been found equal to 0.07 and 0.04, respectively, which is smaller than the standard deviations of the distributions of $l_{k\mathbf{i}}$. Hence we concluded that the standard deviation of the distributions of $l_{k\mathbf{i}}$ is dominated by the intrinsic dispersion of the characteristic times within the bacteria population and not by the dispersion of the light intensities of the instrumental setup as it was observed in RSFP solutions.

Supplementary Table 14: Elements of the symmetrical covariance matrix Σ^i defined in Eq. (21) retrieved from the distributions of the decimal logarithms of the characteristic times $\{\tau^{\mathrm{I}}_{low}, \tau^{\mathrm{I}}_{high}, \tau^{\mathrm{II}}_{high}\}$ measured over all imaged RSFP-labeled bacteria of type i. Illumination I$_{low}$: $I_1 = 2$ ein.m^{-2}.s^{-1}, illumination I$_{high}$: $I_1 = 50$ ein.m^{-2}.s^{-1}, and illumination II$_{high}$: $I_1 = 50$ ein.m^{-2}.s^{-1} and $I_2 = 20$ ein.m^{-2}.s^{-1}. T = 298 K.

RSFP i	$\Sigma^i_{l_{1i},l_{1i}}$	$\Sigma^i_{l_{3i},l_{3i}}$	$\Sigma^i_{l_{4i},l_{4i}}$	$\Sigma^i_{l_{1i},l_{3i}}$	$\Sigma^i_{l_{1i},l_{4i}}$	$\Sigma^i_{l_{3i},l_{4i}}$
**1**	1.24×10^{-2}	5.03×10^{-3}	6.61×10^{-3}	4.06×10^{-3}	2.74×10^{-3}	3.37×10^{-3}
**3**	1.69×10^{-2}	5.20×10^{-2}	5.83×10^{-3}	7.40×10^{-4}	7.54×10^{-3}	4.44×10^{-3}
**6**	1.17×10^{-2}	1.64×10^{-3}	5.44×10^{-3}	1.86×10^{-3}	-6.83×10^{-4}	-2.64×10^{-4}
**8**	6.73×10^{-3}	9.20×10^{-3}	1.08×10^{-2}	6.91×10^{-3}	1.04×10^{-3}	3.04×10^{-3}
**9**	1.11×10^{-2}	1.34×10^{-2}	1.69×10^{-2}	9.76×10^{-3}	5.41×10^{-3}	1.04×10^{-2}
**10**	9.14×10^{-3}	1.16×10^{-2}	5.13×10^{-3}	3.49×10^{-3}	4.94×10^{-4}	-7.53×10^{-4}
**11**	1.01×10^{-2}	9.66×10^{-3}	7.38×10^{-3}	2.14×10^{-3}	4.00×10^{-3}	-7.24×10^{-4}
**12**	3.46×10^{-2}	3.94×10^{-2}	3.97×10^{-2}	6.50×10^{-3}	-2.57×10^{-2}	-1.44×10^{-2}
**13**	1.04×10^{-2}	6.82×10^{-3}	6.36×10^{-3}	3.66×10^{-3}	-2.02×10^{-3}	2.30×10^{-3}
**14**	6.83×10^{-3}	7.99×10^{-3}	8.89×10^{-3}	6.70×10^{-3}	3.93×10^{-3}	3.97×10^{-3}
**15**	1.70×10^{-2}	4.26×10^{-2}	1.46×10^{-2}	2.03×10^{-3}	6.82×10^{-3}	-7.32×10^{-4}
**17**	3.63×10^{-2}	2.54×10^{-2}	4.91×10^{-3}	2.17×10^{-2}	-1.39×10^{-3}	-4.54×10^{-3}
**18**	1.54×10^{-2}	1.12×10^{-2}	1.18×10^{-1}	3.95×10^{-3}	-2.82×10^{-2}	5.56×10^{-3}
**19**	1.68×10^{-2}	2.06×10^{-2}	4.79×10^{-3}	2.17×10^{-3}	7.76×10^{-4}	-2.82×10^{-3}
**20**	1.61×10^{-2}	1.69×10^{-2}	8.34×10^{-3}	4.06×10^{-3}	-2.57×10^{-3}	-4.94×10^{-3}
**22**	1.91×10^{-2}	2.45×10^{-2}	8.37×10^{-3}	1.02×10^{-4}	-2.83×10^{-3}	-3.09×10^{-4}

The sorting algorithm reported in paragraph 1.7.1 and the distance between pairs given in Eq. (15) were used for $n = 3$ to obtain optimized subsets $s'(m)$ of m RSFP-labeled bacteria and the associated minimum distance $d_{\min}$. We found that several subsets of $m = 10$ RSFP-labeled bacteria are associated with $d_{\min} = 0.27$ of the order of d_c. The results are given in Supplementary Table 15. The optimized subset $J = \{\mathbf{1}, \mathbf{3}, \mathbf{6}, \mathbf{11}, \mathbf{13}, \mathbf{14}, \mathbf{15}, \mathbf{17}, \mathbf{20}\}$ was chosen to illustrate the discrimination power of LIGHTNING using RSFP-labeled bacteria.

D.2.3 LIGHTNING discrimination of 9 RSFP-labeled bacteria

The data used to determine the kinetic fingerprints of the 9 selected RSFP-labeled bacteria were considered again. Knowing the characteristic times and the associated variances and covariances given in Supplementary Table 13 and Supplementary Table 14, we computed the probability P_{ij} defined in Eq. (20) that a bacterium i is identical to one of the 9 tabulated RSFP-labeled bacteria j. The results are illustrated in Supplementary Figure 62. The small square at the top left of each Supplementary Figure 62a–i provides an image of the same sample containing bacteria labeled by RSFP **1** only. For each bacterium i the probability P_{i1} was computed and the results are represented in shades of gray in the top left square of Supplementary Figure 62a. A bacterium i is lighter as P_{i1} tends to 1, i.e. the kinetic fingerprint of i is closer to the tabulated kinetic fingerprint of the bacteria labeled by **1**. The top left squares of Supplementary Figure 62b–i give analogous images based on the values of P_{ij} for $j =$ **3**,**6**,**11**,**13**,**14**,**15**,**17**,**20**. Among the top left squares, light images of bacteria mainly appear in Supplementary Figure 62a, revealing that only P_{i1} values are significant. The top left squares of Supplementary Figure 62b–i reveal very few images of bacteria due to the very small values of P_{ij} for $j \neq \mathbf{1}$. Analogous results are observed in the 8 remaining squares of each subfigure of Supplementary Figure 62 for samples containing bacteria labeled with one of the 8 other RSFPs.

The probabilities P_{ij} that a bacterium i is identical to a tabulated RSFP-labeled bacterium j (see Eq. (20)) have been computed for all RSFP-labeled bacteria of all 9 types in the set $J = \{\mathbf{1}, \mathbf{3}, \mathbf{6}, \mathbf{11}, \mathbf{13}, \mathbf{14}, \mathbf{15}, \mathbf{17}, \mathbf{20}\}$. They were used to assign a single type to a supposedly unknown bacterium: A bacterium i of a sample is predicted to be of

Supplementary Table 15: Optimized subsets $s'(10)$ of 10 RSFP-labeled bacteria associated with a minimum distance $d_{\min} = 0.27$ obtained using the kinetic fingerprints given in Supplementary Table 13.

(**1**,**6**,**11**,**12**,**13**,**14**,**15**,**17**,**19**,**22**)
(**1**,**6**,**11**,**12**,**13**,**14**,**15**,**17**,**19**,**20**)
(**1**,**6**,**10**,**12**,**13**,**14**,**15**,**17**,**19**,**22**)
(**1**,**6**,**10**,**12**,**13**,**14**,**15**,**17**,**19**,**20**)
(**1**,**6**,**8**,**11**,**12**,**13**,**15**,**17**,**19**,**22**)
(**1**,**6**,**8**,**11**,**12**,**13**,**15**,**17**,**19**,**20**)
(**1**,**6**,**8**,**10**,**12**,**13**,**15**,**17**,**19**,**22**)
(**1**,**6**,**8**,**10**,**12**,**13**,**15**,**17**,**19**,**20**)
(**1**,**3**,**6**,**11**,**13**,**14**,**15**,**17**,**19**,**22**)
(**1**,**3**,**6**,**11**,**13**,**14**,**15**,**17**,**19**,**20**)
(**1**,**3**,**6**,**10**,**13**,**14**,**15**,**17**,**19**,**22**)
(**1**,**3**,**6**,**10**,**13**,**14**,**15**,**17**,**19**,**20**)
(**1**,**3**,**6**,**8**,**11**,**13**,**15**,**17**,**19**,**22**)
(**1**,**3**,**6**,**8**,**11**,**13**,**15**,**17**,**19**,**20**)
(**1**,**3**,**6**,**8**,**10**,**13**,**15**,**17**,**19**,**22**)
(**1**,**3**,**6**,**8**,**10**,**13**,**15**,**17**,**19**,**20**)

type l when the probability P_{il} is the maximum value of the P_{ij} set for $j \in J$. The accuracy defined as the ratio of the number of correctly identified bacteria and the total number of RSFP-labeled bacteria is equal to 0.93. Figure 4c illustrates the results. Each sample contains one type k of RSFP-labeled bacteria. A false color is assigned to each of the 9 predicted RSFP types l and the lightness of each RSFP-labeled bacterium i is set by the P_{il} value. If one excepts rare mislabeling, the sample containing bacteria of actual type k are correctly colored according to the predicted type $l = k$. The performance of the recognition of RSFP-labeled bacteria was further evaluated using a confusion matrix of elements C_{kl} defined as the scaled number of RSFP-labeled bacteria of true label $k \in J$ and predicted label $l \in J$. The scaling factor is the total number of RSFP-labeled bacteria of true label k. The diagonal elements C_{kk} of the confusion matrix give the proportion of true positives whereas the nondiagonal elements give the proportion of false positives i.e. of assigned type l different from the actual type k. The confusion matrix associated with the set J of 9 RSFP-labeled bacteria is given in Figure 4d. The confusion matrix is very close to the identity matrix, revealing the LIGHTNING discriminative power.

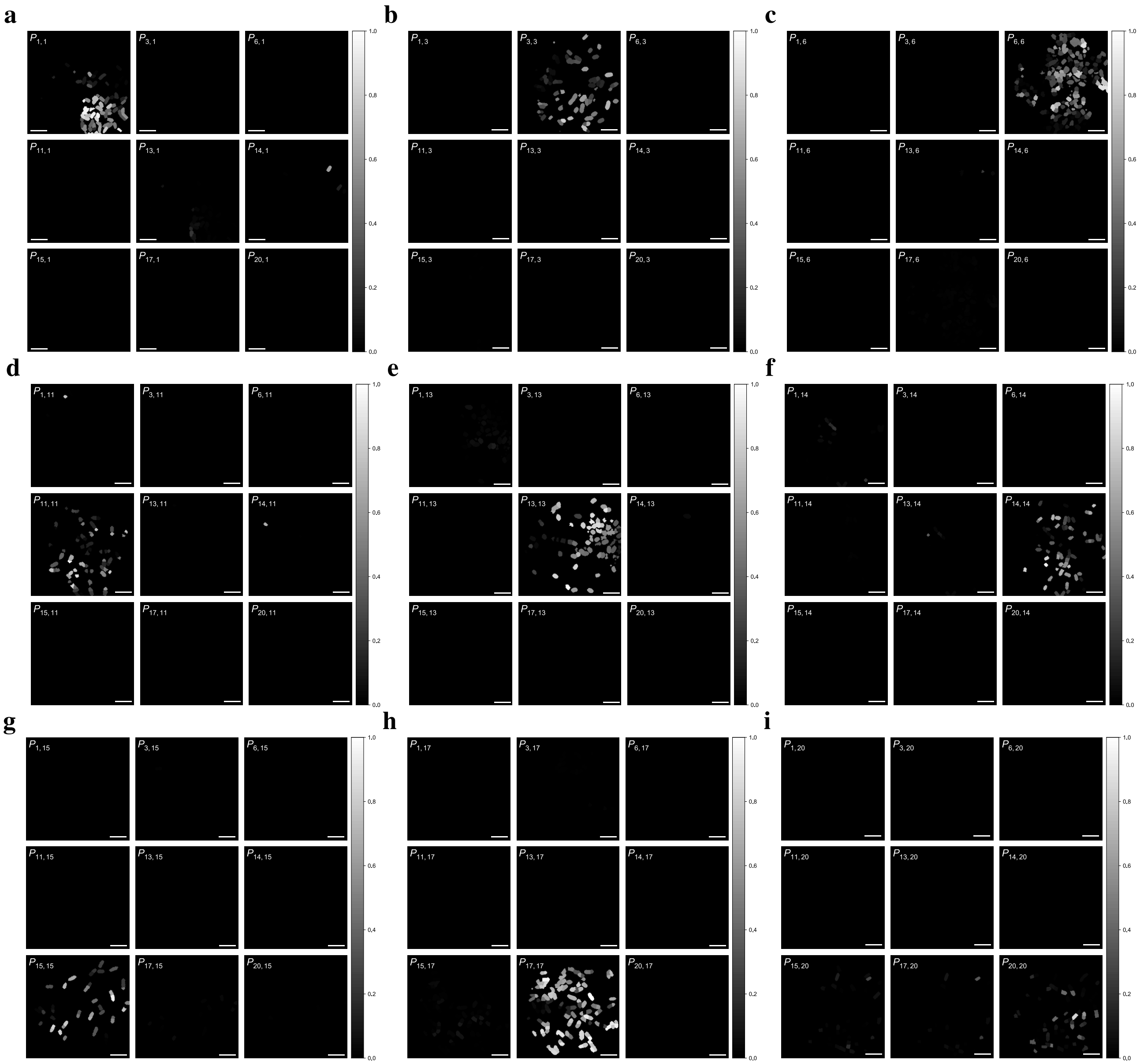

Supplementary Figure 62: *Images of RSFP-labeled bacteria based on the probability* P_{ij}. Each subfigure is divided into 9 squares. Each square is the image of a sample containing bacteria i of one of the 9 selected types k. From left to right, first line: $k = \mathbf{1}, \mathbf{3}, \mathbf{6}$, second line: $k = \mathbf{11}, \mathbf{13}, \mathbf{14}$, third line: $k = \mathbf{15}, \mathbf{17}, \mathbf{20}$. The subfigures **a-i** give different images of the same 9 samples using the values of the probability P_{ij} for the different tabulated RSFP-labeled bacteria j. **a**: $j = \mathbf{1}$, **b**: $j = \mathbf{3}$, **c**: $j = \mathbf{6}$, **d**: $j = \mathbf{11}$, **e**: $j = \mathbf{13}$, **f**: $j = \mathbf{15}$. For each bacterium i, the lightness is set by the value of probability P_{ij} according to the linear grey scale between 0 and 1. Scaling bar = 5 μm.

D.3 The LIGHTNING robustness

The kinetic fingerprints of the RSFPs reported in Supplementary Table 7 and Supplementary Table 13, and in the Main Text have been determined in solution and in fixed bacteria under different conditions of acquisition due to experimental constraints. To evaluate the robustness of LIGHTNING, we retrieved the kinetic fingerprints of two representatives of slow and fast photoswitching RSFPs, **1** (Dronpa) and **2** (Dronpa-2), in different environments – solution, fixed and living bacteria, which have been acquired at identical frequency and time window of acquisition and for different light intensities.

D.3.1 Results

The solutions of **1** and **2** as well as the associated fixed and living labeled bacteria have been submitted to illuminations I and II with various combinations of light intensities I_1 and I_2. More precisely, we explored four combinations covering the four regimes $\{\mathrm{I_{low}}, \mathrm{II_{low}}, \mathrm{I_{high}}, \mathrm{II_{high}}\}$. We used the photoswitchometer in the configuration displayed in Supplementary Figure 1a with a high sampling frequency (100 kHz) and a long acquisition time (0.5 s) in order to bridge the conditions of acquisition of both series of experiments reported in Supplementary Table 7 and Supplementary Table 13, and in the Main Text.

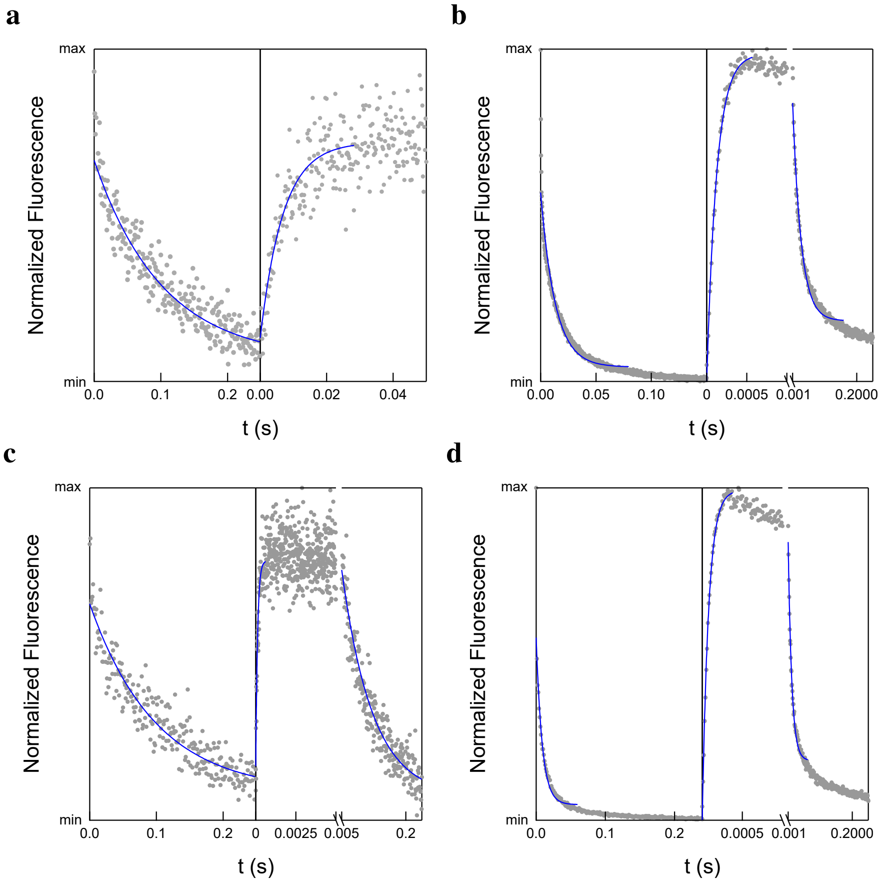

Supplementary Figure 63: Kinetic analysis of **1** photoswitching at 4 μM in pH 7.4 PBS (50 mM sodium phosphate, 150 mM NaCl); T = 298 K. **a–d**: Evolution of the fluorescence intensity scaled by the initial value upon continuous illumination of intensity I_1 at $\lambda_1 = 488$ nm and square-wave illumination of intensity I_2 at $\lambda_2 = 405$ nm (**a**: $I_1 = 2$ ein.m^{-2}.s^{-1}, $I_2 = 0.2$ ein.m^{-2}.s^{-1}; **b**: $I_1 = 50$ ein.m^{-2}.s^{-1}, $I_2 = 20$ ein.m^{-2}.s^{-1}; **c**: $I_1 = 2$ ein.m^{-2}.s^{-1}, $I_2 = 50$ ein.m^{-2}.s^{-1}; **d**: $I_1 = 100$ ein.m^{-2}.s^{-1}, $I_2 = 50$ ein.m^{-2}.s^{-1}). Black dots: experimental data; red line: monoexponential fitting functions.

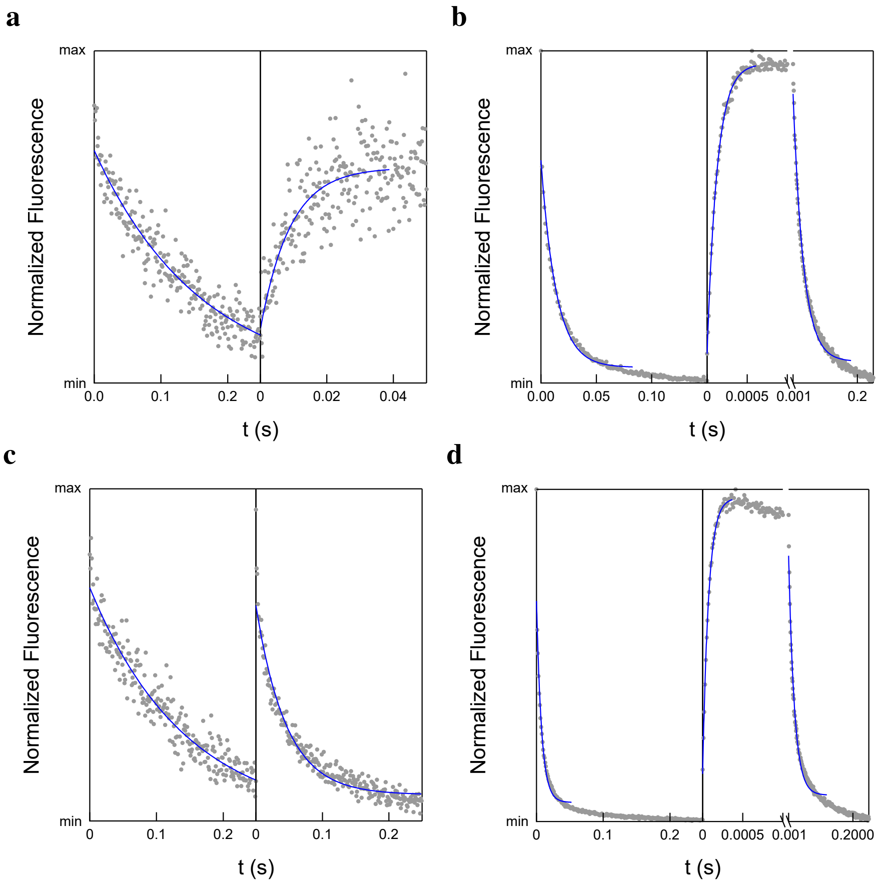

Supplementary Figure 64: Kinetic analysis of **1** photoswitching in **1**-labeled living bacteria; T = 298 K. **a**–**d**: Evolution of the fluorescence intensity scaled by the initial value upon continuous illumination of intensity I_1 at $\lambda_1 = 488$ nm and square-wave illumination of intensity I_2 at $\lambda_2 = 405$ nm (**a**: $I_1 = 2$ ein.m^{-2}.s^{-1}, $I_2 = 0.2$ ein.m^{-2}.s^{-1}; **b**: $I_1 = 50$ ein.m^{-2}.s^{-1}, $I_2 = 20$ ein.m^{-2}.s^{-1}; **c**: $I_1 = 2$ ein.m^{-2}.s^{-1}, $I_2 = 50$ ein.m^{-2}.s^{-1}; **d**: $I_1 = 100$ ein.m^{-2}.s^{-1}, $I_2 = 50$ ein.m^{-2}.s^{-1}). Black dots: experimental data; red line: monoexponential fitting functions.

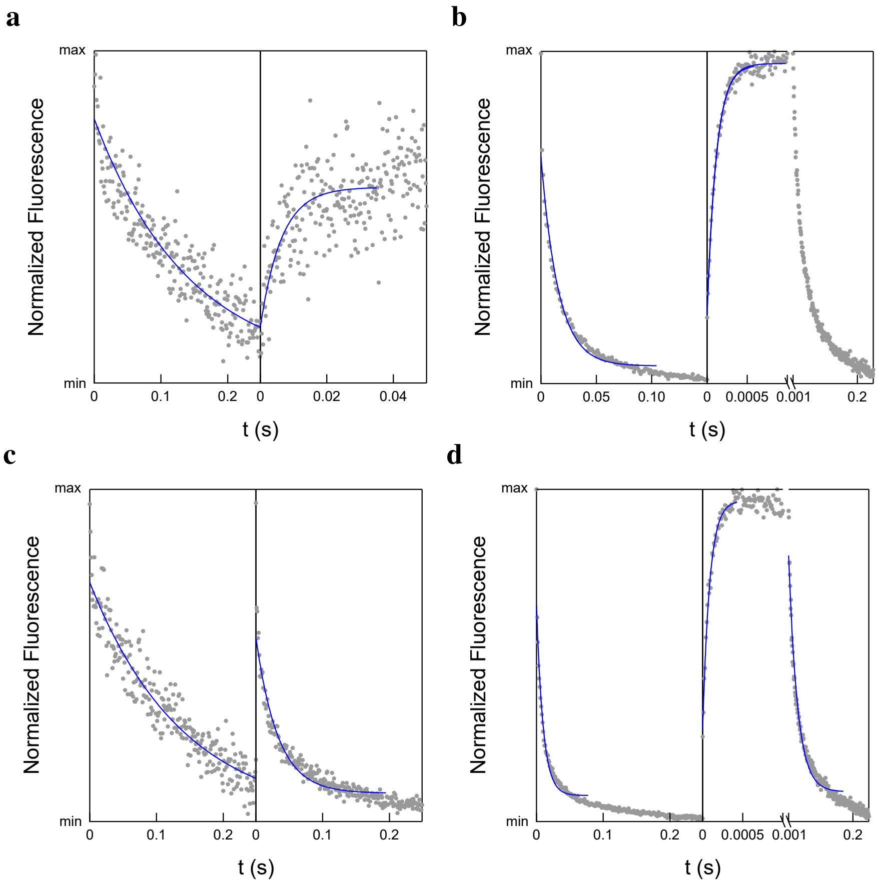

Supplementary Figure 65: Kinetic analysis of **1** photoswitching in **1**-labeled fixed bacteria; T = 298 K. **a**–**d**: Evolution of the fluorescence intensity scaled by the initial value upon continuous illumination of intensity I_1 at $\lambda_1 = 488$ nm and square-wave illumination of intensity I_2 at $\lambda_2 = 405$ nm (**a**: $I_1 = 2$ ein.m^{-2}.s^{-1}, $I_2 = 0.2$ ein.m^{-2}.s^{-1}; **b**: $I_1 = 50$ ein.m^{-2}.s^{-1}, $I_2 = 20$ ein.m^{-2}.s^{-1}; **c**: $I_1 = 2$ ein.m^{-2}.s^{-1}, $I_2 = 50$ ein.m^{-2}.s^{-1}; **d**: $I_1 = 100$ ein.m^{-2}.s^{-1}, $I_2 = 50$ ein.m^{-2}.s^{-1}). Black dots: experimental data; red line: monoexponential fitting functions.

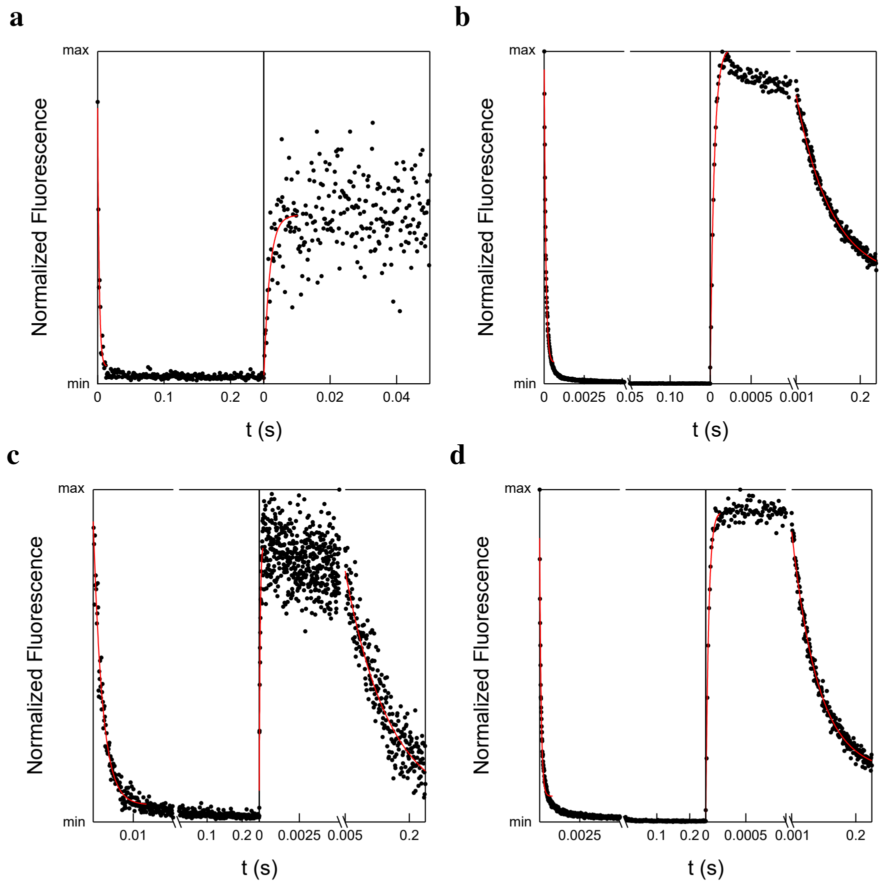

Supplementary Figure 66: Kinetic analysis of **2** photoswitching at 10 μM in pH 7.4 PBS (50 mM sodium phosphate, 150 mM NaCl); T = 298 K. **a**–**d**: Evolution of the fluorescence intensity scaled by the initial value upon continuous illumination of intensity I_1 at $\lambda_1 = 488$ nm and square-wave illumination of intensity I_2 at $\lambda_2 = 405$ nm (**a**: $I_1 = 2$ ein.m^{-2}.s^{-1}, $I_2 = 0.2$ ein.m^{-2}.s^{-1}; **b**: $I_1 = 50$ ein.m^{-2}.s^{-1}, $I_2 = 20$ ein.m^{-2}.s^{-1}; **c**: $I_1 = 2$ ein.m^{-2}.s^{-1}, $I_2 = 50$ ein.m^{-2}.s^{-1}; **d**: $I_1 = 100$ ein.m^{-2}.s^{-1}, $I_2 = 50$ ein.m^{-2}.s^{-1}). Black dots: experimental data; red line: monoexponential fitting functions.

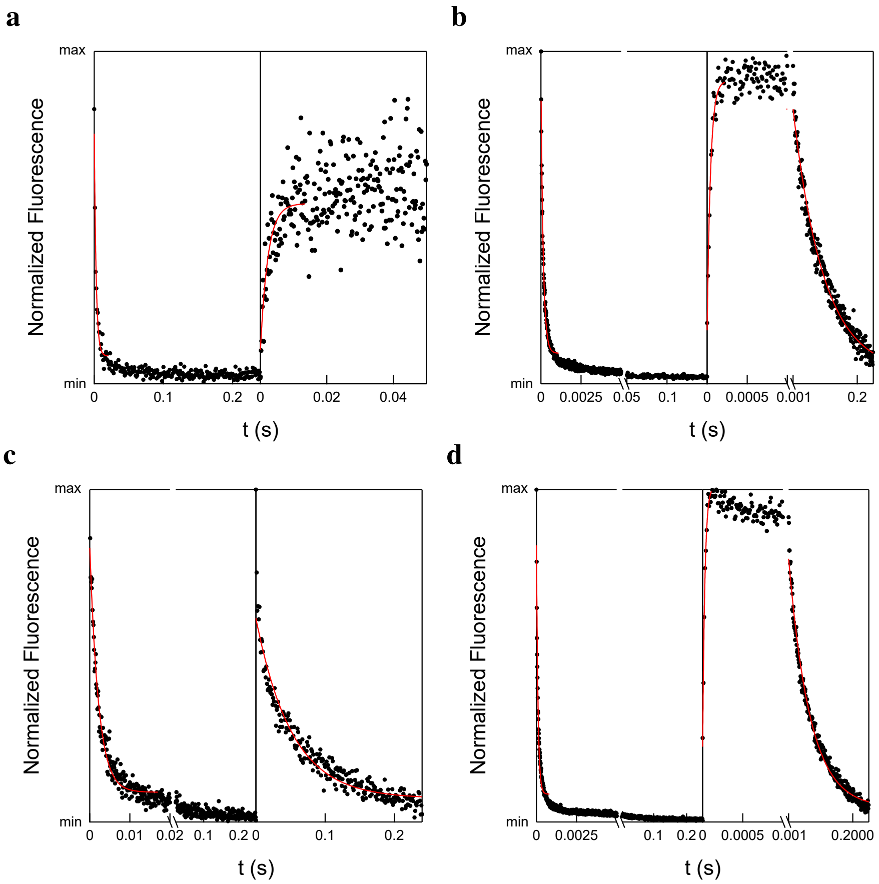

Supplementary Figure 67: Kinetic analysis of **2** photoswitching in **2**-labeled living bacteria; T = 298 K. **a**–**d**: Evolution of the fluorescence intensity scaled by the initial value upon continuous illumination of intensity I_1 at $\lambda_1 = 488$ nm and square-wave illumination of intensity I_2 at $\lambda_2 = 405$ nm (**a**: $I_1 = 2$ ein.m^{-2}.s^{-1}, $I_2 = 0.2$ ein.m^{-2}.s^{-1}; **b**: $I_1 = 50$ ein.m^{-2}.s^{-1}, $I_2 = 20$ ein.m^{-2}.s^{-1}; **c**: $I_1 = 2$ ein.m^{-2}.s^{-1}, $I_2 = 50$ ein.m^{-2}.s^{-1}; **d**: $I_1 = 100$ ein.m^{-2}.s^{-1}, $I_2 = 50$ ein.m^{-2}.s^{-1}). Black dots: experimental data; red line: monoexponential fitting functions.

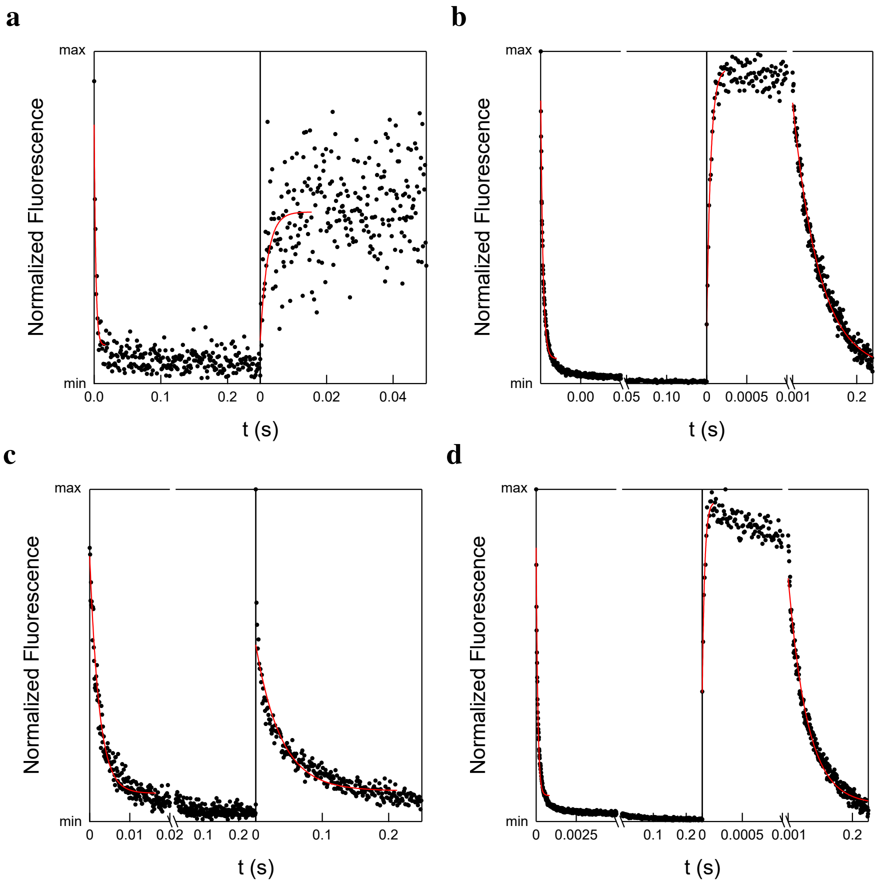

Supplementary Figure 68: Kinetic analysis of **2** photoswitching in **2**-labeled fixed bacteria; T = 298 K. **a**–**d**: Evolution of the fluorescence intensity scaled by the initial value upon continuous illumination of intensity I_1 at $\lambda_1 = 488$ nm and square-wave illumination of intensity I_2 at $\lambda_2 = 405$ nm (**a**: $I_1 = 2$ ein.m^{-2}.s^{-1}, $I_2 = 0.2$ ein.m^{-2}.s^{-1}; **b**: $I_1 = 50$ ein.m^{-2}.s^{-1}, $I_2 = 20$ ein.m^{-2}.s^{-1}; **c**: $I_1 = 2$ ein.m^{-2}.s^{-1}, $I_2 = 50$ ein.m^{-2}.s^{-1}; **d**: $I_1 = 100$ ein.m^{-2}.s^{-1}, $I_2 = 50$ ein.m^{-2}.s^{-1}). Black dots: experimental data; red line: monoexponential fitting functions.

As displayed in Supplementary Figure 63–Supplementary Figure 68, similar fluorescence evolutions are observed in the three samples containing a same RSFP in aqueous solution, living and fixed bacteria. Interestingly, the kinetic models introduced in section C account well for the fluorescence evolutions observed with both **1** and **2**:

- The kinetic regime corresponding to illumination $\mathrm{I}_{\mathrm{low}}$ is observed for $I_1 = 2$ ein.m^{-2}.s^{-1}, smaller than the cutoff intensities I_1^c of **1** and **2** (see Supplementary Table 3). The fluorescence intensity monoexponentially decreases as predicted by the two-state mechanism given in subsection C.3.1;

- The kinetic regime corresponding to illumination $\mathrm{II}_{\mathrm{low}}$ is observed for $I_1 = 2$ ein.m^{-2}.s^{-1} and $I_2 = 0.2$ ein.m^{-2}.s^{-1}, smaller than the cutoff intensities I_2^c of **1** and **2** (see Supplementary Table 3). The fluorescence intensity monoexponentially increases as expected from the two-state mechanism given in subsection C.3.3;

- The kinetic regime corresponding to illumination $\mathrm{I}_{\mathrm{high}}$ is observed for $I_1 = 50$ and 100 ein.m^{-2}.s^{-1} larger than the cutoff intensities I_1^c of **1** and **2** (see Supplementary Table 3). The fluorescence intensity exhibits at least a biexponential decay as expected from the three-state mechanism given in subsection C.3.2;

- The kinetic regime corresponding to illumination $\mathrm{II}_{\mathrm{high}}$ is observed for I_1=2, 50, and 100 ein.m^{-2}.s^{-1} and I_2=20 and 50 ein.m^{-2}.s^{-1} larger than the cutoff intensities I_2^c of **1** and **2** (see Supplementary Table 3). The

fluorescence intensity first increases and then slowly decreases as expected from the four-state mechanism given in subsection C.3.4.

The time evolution of the fluorescence signal displayed in Supplementary Figure 63–Supplementary Figure 68 has been processed using a monoexponential curve fitting as explained in subsection 1.4 in the case of illuminations $\mathrm{I}_{\mathrm{low}}$, $\mathrm{II}_{\mathrm{low}}$, and $\mathrm{I}_{\mathrm{high}}$ in order to extract the characteristic times τ^{I}_{low}, τ^{II}_{low}, and τ^{I}_{high}. For illumination $\mathrm{II}_{\mathrm{high}}$, the fluorescence evolution displays a maximum of argument t_{max}. We first defined two time windows, $[0, t_{\mathrm{max}}]$ and $[t_{\mathrm{max}}, 0.5\ \mathrm{s}]$. Then, we fitted a monoexponential function to the fluorescence evolution in each time window and extracted two characteristic times, $\tau^{\mathrm{II}}_{high,\uparrow}$ and $\tau^{\mathrm{II}}_{high,\downarrow}$, associated with the increase and the decrease of fluorescence, respectively. The results are displayed in Supplementary Table 16 and Supplementary Table 17.

D.3.2 Discussion

The preceding results have been analyzed along different perspectives aiming at evaluating the robustness of the LIGHTNING kinetic fingerprint of a given RSFP.

Dependence of the LIGHTNING kinetic fingerprint on the RSFP environment We analyzed the impact of the RSFP environment on the characteristic times acquired under similar conditions of illumination, time window, and acquisition frequency in aqueous solution, fixed and living bacteria. Supplementary Table 16 and Supplementary Table 17 show that the five characteristic times τ^{I}_{low}, τ^{II}_{low}, τ^{I}_{high}, $\tau^{\mathrm{II}}_{high,\uparrow}$, and $\tau^{\mathrm{II}}_{high,\downarrow}$ measured in the three different environments are in fair agreement. For the sake of simplicity, the five characteristic times are denoted τ in the remaining part of the paragraph. Using Supplementary Table 16 and Supplementary Table 17, we computed (i) the mean characteristic time $\bar{\tau}^i_j$ averaged over the three environments for each illumination condition, (ii) the maximum ratio $r = \max(\tau/\bar{\tau}, \bar{\tau}/\tau)$ for each illumination condition and each environment, (iii) the mean ratio $\langle \bar{r} \rangle$ averaged over the three environments and all illumination conditions. The kinetic fingerprint of RSFP **1** is especially robust since we obtained $\langle \bar{r} \rangle = 1.2$ with r always smaller than 1.5. This result leads to $\log_{10}\langle \bar{r} \rangle = 0.07$ which is consistent with the uncertainty $M = 0.08$ on the determination of the 16 kinetic fingerprints of the fixed RSFP-labeled bacteria (see section D.2.2). RSFP **2** is slightly more sensitive to its environment. We found the satisfying result $\langle \bar{r} \rangle = 1.4$ but three r values are between 2 and 4.2. Moreover, $\log_{10}\langle \bar{r} \rangle = 0.15$ which is larger than the uncertainty M and one of the reasons for which RSFP **2** has been excluded from the 16 selected fixed RSFP-labeled bacteria. These results are in line with a previous report on the weak sensitivity of RSFP photoswitching to environmental changes[29] and with our preceding observations where similar characteristic times in the regimes of low illumination have been observed in aqueous solutions, colonies of bacteria, and living and fixed eucaryotic animal and plant cells.[7,30–32]

Supplementary Table 16: *Kinetic fingerprints* $\{\tau^{\mathrm{I}}_{low}, \tau^{\mathrm{II}}_{low}, \tau^{\mathrm{I}}_{high}, \tau^{\mathrm{II}}_{high,\uparrow}, \tau^{\mathrm{II}}_{high,\downarrow}\}$ *of **1** in solution (S) and **1**-labeled living (L) and fixed (F) bacteria.* The characteristic times result from five independent acquisitions, which have been measured with the photoswitchometer in the configuration displayed in Supplementary Figure 1a. T = 298 K.

Sample	I_1 (ein/m^2.s)	$10^2 \times \tau^{\mathrm{I},}_{low}$ [a] (s)	$10^3 \times \tau^{\mathrm{I}}_{high}$ [c] (s)	I_2 (ein/m^2.s)	$10^3 \times \tau^{\mathrm{II}}_{low}$ [b] (s)	$10^5 \times \tau^{\mathrm{II}}_{high,\uparrow}$ [d] (s)	$10^3 \times \tau^{\mathrm{II}}_{high,\downarrow}$ [e] (s)
S	2	10.1±0.2	–	0.2	7.0±0.3	–	–
L	2	15.7±0.4	–	0.2	8.7±0.7	–	–
F	2	14±1	–	0.2	5.1±0.3	–	–
S	50	–	14.0±0.1	20	–	12.5±0.2	27.2±0.7
L	50	–	15.8±0.1	20	–	12.7±0.6	36.0±0.9
F	50	–	16.0±0.3	20	–	12.0±0.7	36±1
S	2	9.96 ±0.03	–	50	–	11.5±0.4	92.8±0.7
L	2	14.3 ±0.5	–	50	–	–[f]	53±2
F	2	14.2 ±0.2	–	50	–	–[f]	48±2
S	100	–	8.9±0.1	50	–	7.9±0.2	14.1±0.5
L	100	–	7.4±0.3	50	–	7.2±0.2	16.8±0.8
F	100	–	10.6±0.5	50	–	8.6±0.2	29±2

[a] $10^2 \times \tau^{\mathrm{I},}_{low} = 9.8$ s was anticipated from Supplementary Table 3 by using the value of $\Sigma_1 = 5.1$ m^2.mol^{-1} and $I_1 = 2$ ein.m^{-2}.s^{-1}. Note that the excellent agreement between the τ^{I}_{low} values retrieved from the two series experiments at the same light intensity $I_1 = 2$ ein.m^{-2}.s^{-1} evidences the reproducibility of the experiments;

[b] $10^3 \times \tau^{\mathrm{II}}_{low} = 12.5$ s was anticipated from Supplementary Table 3 by using the value of $\Sigma_1 = 5.1$ m^2.mol^{-1}, $\Sigma_2 = 350$ m^2.mol^{-1}, $I_1 = 2$ ein.m^{-2}.s^{-1}, and $I_2 = 0.2$ ein.m^{-2}.s^{-1};

[c] $10^3 \times \tau^{\mathrm{I}}_{high} = 4.5$ s and $10^3 \times \tau^{\mathrm{I}}_{high} = 16$ s were anticipated from Supplementary Table 3 and Supplementary Table 13 respectively;

[d] $10^5 \times \tau^{\mathrm{II}}_{high} = 18.5$ s was anticipated from Supplementary Table 3;

[e] $10^3 \times \tau^{\mathrm{I}}_{high} = 34$ s was anticipated from Supplementary Table 13;

[f] No signal rise could be recorded under our conditions of acquisition.

Dependence of the LIGHTNING kinetic fingerprint on the acquisition parameters We evaluated the robustness of the LIGHTNING kinetic fingerprint to the acquisition parameters using the experimental results and the kinetic mechanisms introduced in section C. We analyzed the impact of the light intensities I_1 and I_2, the frequency and the time window of fluorescence acquisition on the LIGHTNING kinetic fingerprint reported in Supplementary Table 7, Supplementary Table 13, Supplementary Table 16, and Supplementary Table 17.

Supplementary Table 17: *Kinetic fingerprints* $\{\tau^{\mathrm{I}}_{low}, \tau^{\mathrm{II}}_{low}, \tau^{\mathrm{I}}_{high}, \tau^{\mathrm{II}}_{high,\uparrow}, \tau^{\mathrm{II}}_{high,\downarrow}\}$ *of **2** in solution (S) and **2**-labeled living (L) and fixed (F) bacteria.* The characteristic times result from five independent acquisitions, which have been measured with the photoswitchometer in the configuration displayed in Supplementary Figure 1a. T = 298 K.

Sample	I_1 (ein/m^2.s)	$10^3 \times \tau^{\mathrm{I},}_{low}$ [a] (s)	$10^4 \times \tau^{\mathrm{I}}_{high}$ [c] (s)	I_2 (ein/m^2.s)	$10^3 \times \tau^{\mathrm{II}}_{low}$ [b] (s)	$10^5 \times \tau^{\mathrm{II}}_{high,\uparrow}$ [d] (s)	$10^3 \times \tau^{\mathrm{II}}_{high,\downarrow}$ (s)
S	2	2.1±0.2	–	0.2	1.88±0.05	–	–
L	2	1.9±0.2	–	0.2	1.9±0.3	–	–
F	2	2.6±0.4	–	0.2	4±1	–	–
S	50	–	1.88±0.07	20	–	5.8±0.3	161±17
L	50	–	5±1	20	–	9±1	195±54
F	50	–	9±4	20	–	12±7	106±18
S	2	2.1±0.1	–	50	–	3.8±0.5	134 ± 3
L	2	3.6±0.7	–	50	–	–[e]	78 ± 3
F	2	2.6±0.3	–	50	–	–[e]	39 ± 3
S	100	–	1.45±0.04	50	–	–[e]	86±3
L	100	–	10±3	50	–	5.4±1.8	122±20
F	100	–	7.0±0.6	50	–	6.1±1.6	81±5

[a] $10^3 \times \tau^{\mathrm{I},}_{low} = 2.5$ s was anticipated from Supplementary Table 3 by using the value of $\Sigma_1 = 198$ m^2.mol^{-1} and $I_1 = 2$ ein.m^{-2}.s^{-1}. Note that the excellent agreement between the τ^{I}_{low} values retrieved from the two series experiments at the same light intensity $I_1 = 2$ ein.m^{-2}.s^{-1} evidences the reproducibility of the experiments;
[b] $10^3 \times \tau^{\mathrm{II}}_{low} = 2.1$ s was anticipated from Supplementary Table 3 by using the value of $\Sigma_1 = 198$ m^2.mol^{-1}, $\Sigma_2 = 415$ m^2.mol^{-1}, $I_1 = 2$ ein.m^{-2}.s^{-1}, and $I_2 = 0.2$ ein.m^{-2}.s^{-1};
[c] $10^4 \times \tau^{\mathrm{I}}_{high} = 3.3$ s was anticipated from Supplementary Table 3;
[d] $10^5 \times \tau^{\mathrm{II}}_{high} = 7.7$ s was anticipated from Supplementary Table 3;
[e] Too fast to be reliably evaluated.

Impact of the light intensities I_1 and I_2 Both the experimental results shown in Supplementary Figure 21–Supplementary Figure 42c–f and the kinetic models of section C highlight the dependence of the characteristic times τ^{I} and τ^{II} on the light intensities I_1 and I_2:

- In the kinetic regimes corresponding to illuminations $\mathrm{I_{low}}$ and $\mathrm{II_{low}}$, kinetics is controlled by a photochemical step as evidenced by the linear dependence of $1/\tau^{\mathrm{I}}_{low}$ and $1/\tau^{\mathrm{II}}_{low}$ on I_1 and I_2, respectively. As shown in Supplementary Table 16, Supplementary Table 17, and Supplementary Table 18, the characteristic times τ^{I}_{low} and τ^{II}_{low} are reliably predicted using Eqs.(114,118) and the isomerization cross sections given Supplementary Table 3. Supplementary Table 18 further demonstrates the good agreement between the values of τ^{I}_{low} and τ^{II}_{low} measured in solution and fixed bacteria;

- In the kinetic regimes corresponding to illuminations $\mathrm{I_{high}}$ and $\mathrm{II_{high}}$, thermal steps intervene in the photoswitching kinetics and τ^{I}_{high}, $\tau^{\mathrm{II}}_{high,\uparrow}$, and $\tau^{\mathrm{II}}_{high,\uparrow}$ exhibit no significant or a weak dependence on light intensities as evidenced by the data shown in Supplementary Table 16 and Supplementary Table 17.

Supplementary Table 18: Comparison of the characteristic times $\tau^{\text{I,sol}}_{low,exp}$ and $\tau^{\text{II,sol}}_{low,exp}$ measured in RSFP solutions (see Supplementary Table 7), $\tau^{\text{I,bact}}_{low,exp}$ in fixed RSFP-labeled bacteria (see Supplementary Table 13), and $\tau^{\text{I,sol}}_{low,th}$ and $\tau^{\text{II,sol}}_{low,th}$ computed using the isomerization cross sections (see Supplementary Table 3) for illumination I_{low}: $I_1 = 2$ ein.m^{-2}.s^{-1} and illumination II_{low}: $I_1 = 0.1$ ein.m^{-2}.s^{-1} and $I_2 = 0.1$ ein.m^{-2}.s^{-1}.

RSFP	$10^3 \times \tau^{\text{I,sol}}_{low,exp}$ [a]	$10^3 \times \tau^{\text{I,bact}}_{low,exp}$ [a]	$10^3 \times \tau^{\text{I,sol}}_{low,th}$ [a]	$10^3 \times \tau^{\text{II,sol}}_{low,exp}$ [b]	$10^3 \times \tau^{\text{II,sol}}_{low,th}$ [b]
	(s)	(s)	(s)	(s)	(s)
**1**	140[c]	110[d]	98	31.5	28.2
**2**	2.2[c]	–	2.5	25.3	16.3
**3**	25.7	17	16.7	24	26.3
**4**	11.2	–	7.14	42.6	33.3
**5**	14.2	–	13.9	123	94.3
**6**	5.1	63	4.46	16.7	12.8
**7**	17	–	15.6	18.4	14.7
**8**	21.1	93	15.2	16.1	18.2
**9**	580	120	417	18.4	21.5
**10**	25.3	37	20	21.4	22.5
**11**	31.6	41	20	22.3	22.5
**12**	8.6	12	10	14.4	12.8
**13**	71	18	67.6	26.6	32.5
**14**	136	100	82	28.4	32.7
**15**	4.2	10	4.35	26.1	23.5
**16**	4.2	–	4.17	18.2	19.2
**17**	2.45	30	2.36	25.5	18.8
**18**	137	90	98	21.4	20.5
**19**	2.6	11	2.36	7.64	5.31
**20**	3.6	6	2.94	9.52	9.35
**21**	0.32	–	3.47	8.77	9.31
**22**	2.7	4	2.36	7.45	6.79

[a] This characteristic time is associated with a fluorescence decrease (except for **4** and **5**);
[b] This characteristic time is associated with a fluorescence increase (except for **4** and **5**);
[c] $10^3 \times \tau^{\text{I,sol}}_{low,exp} = 101$ s for **1** and $10^3 \times \tau^{\text{I,sol}}_{low,exp} = 2.1$ s for **2** with $I_1 = 2$ ein.m^{-2}.s^{-1} (see Supplementary Table 16 and Supplementary Table 17);
[d] $10^3 \times \tau^{\text{I,bact}}_{low,exp} = 140$ s in fixed bacteria with $I_1 = 2$ ein.m^{-2}.s^{-1} (see Supplementary Table 16);

Impact of the frequency and time window of fluorescence acquisition The conditions of acquisition of the kinetic fingerprints obtained in solution and in labeled bacteria significantly differ by the frequency and the time window of fluorescence acquisition. In solution, we used a 1 MHz acquisition frequency and time windows with upper limit ranging from a few seconds (for illuminations I_{low} and II_{low}) down to some milliseconds (for illuminations I_{high} and II_{high}). In contrast, in imaging bacteria, we used a 1 kHz acquisition frequency and a fixed [1ms–1s] time window.

The acquisition frequency determines the lowest characteristic times that can be retrieved from fluorescence evolution. Hence, whereas characteristic times as small as a few tens of microseconds appear in the kinetic fingerprints obtained in solution (see Supplementary Table 7), only characteristic times above a few milliseconds are found in the kinetic fingerprint of the RSFP-labeled bacteria (see Supplementary Table 13). As a consequence, the too low characteristic times τ^{II}_{low} obtained at appropriate I_2 values (see Supplementary Table 16 and Supplementary Table 17) cannot be extracted for the acquisition frequency used for RSFP-labeled bacteria. In addition, the τ^{II}_{high} values reported in the

kinetic fingerprints recorded in solution and in bacteria significantly differ. Whereas the high acquisition frequency used solution gave access to the characteristic time $\tau^{\mathrm{II}}_{high,\uparrow}$ of the fast fluorescence increase, the lower acquisition frequency used in bacteria led us to characterize the slower fluorescence decrease by the time $\tau^{\mathrm{II}}_{high,\downarrow}$. For the sake of simplicity, $\tau^{\mathrm{II}}_{high}$ is often used instead of $\tau^{\mathrm{II}}_{high,\uparrow}$ or $\tau^{\mathrm{II}}_{high,\downarrow}$.

The results obtained for τ^{I}_{high}, $\tau^{\mathrm{II}}_{high,\uparrow}$, and $\tau^{\mathrm{II}}_{high,\downarrow}$ in solution and bacteria are summed up in Supplementary Table 19. The light intensities I_1 and I_2 used in solution and bacteria differ. However, the impact of intensities has been shown to be too small to explain the discrepancies between the τ^{I}_{high} values. The upper limit of the time window used in solution is smaller than in bacteria. The contribution of the shortest characteristic time of an at least biexponential fluorescence evolution (see subsection C.3.2) to τ^{I}_{high} obtained from a fitted monoexponential function is stronger for a smaller upper limit of the time window. The role of the time window explains the shorter τ^{I}_{high} values obtained in solution than in bacteria.

Supplementary Table 19: Comparison of the characteristic times $\tau^{\mathrm{I,sol}}_{high}$ and $\tau^{\mathrm{II,sol}}_{high,\uparrow}$ measured in RSFP solutions (see Supplementary Table 7) and $\tau^{\mathrm{I,bact}}_{high}$ and $\tau^{\mathrm{II,bact}}_{high,\downarrow}$ measured in fixed RSFP-labeled bacteria (see Supplementary Table 13). Conditions of illumination in solution (in bacteria, resp.): Illumination $\mathrm{I_{high}}$: $I_1 = 200$ ein.m^{-2}.s^{-1} ($I_1 = 50$ ein.m^{-2}.s^{-1}, resp.) and illumination $\mathrm{II_{high}}$: $I_1 = 2$ ein.m^{-2}.s^{-1} and $I_2 = 90$ ein.m^{-2}.s^{-1} ($I_1 = 50$ ein.m^{-2}.s^{-1} and $I_2 = 20$ ein.m^{-2}.s^{-1},resp.).

RSFP	$10^4 \times \tau^{\mathrm{I,sol}}_{high}$ [a]	$10^4 \times \tau^{\mathrm{I,bact}}_{high}$ [a]	$10^5 \times \tau^{\mathrm{II,sol}}_{high,\uparrow}$ [b]	$10^2 \times \tau^{\mathrm{II,bact}}_{high,\downarrow}$ [a]
	(s)	(s)	(s)	(s)
**1**	45.2[c]	160 [d]	18.3[e]	3.4 [f]
**2**	3.34[c]	–	7.73[e]	–
**3**	48.4	27	12.5	2.8
**4**	256	–	1130	–
**5**	35.7	–	925	–
**6**	1.17	250	21.4	4.1
**7**	5.44	–	6.74	–
**8**	42.3	80	9.5	2.2
**9**	59.5	140	12.5	3.0
**10**	6.8	40	7.75	2.1
**11**	38.2	50	11.2	1.5
**12**	3.1	32	6.23	3
**13**	56.3	220	17.5	2.9
**14**	29.2	100	18.4	1.8
**15**	3.14	60	36.4	4
**16**	6.25	–	9.83	–
**17**	4.26	100	12.7	5.1
**18**	38.2	90	38.2	3.2
**19**	1.21	40	3.15	7
**20**	1.31	30	13.5	9
**21**	2.15	–	17.3	–
**22**	1.36	24	6.17	7

[a] This characteristic time is associated with a fluorescence decrease (except for **4** and **5**);
[b] This characteristic time is associated with a fluorescence increase (except for **4** and **5**);
[c] $10^4 \times \tau^{\mathrm{I,sol}}_{high} = 89$ s for **1** and $10^4 \times \tau^{\mathrm{I,sol}}_{high} = 1.45$ s for **2** with $I_1 = 100$ ein.m^{-2}.s^{-1} (see Supplementary Table 16 and Supplementary Table 17);
[d] $10^4 \times \tau^{\mathrm{I,bact}}_{high} = 160$ s in fixed bacteria with $I_1 = 50$ ein.m^{-2}.s^{-1} (see Supplementary Table 16);
[e] $10^5 \times \tau^{\mathrm{II,sol}}_{high,\uparrow} = 11.5$ s for **1** and $10^5 \times \tau^{\mathrm{II,sol}}_{high,\uparrow} = 3.8$ s for **2** with $I_1 = 2$ ein.m^{-2}.s^{-1} and $I_2 = 50$ ein.m^{-2}.s^{-1} (see Supplementary Table 16);
[f] $10^2 \times \tau^{\mathrm{II,bact}}_{high,\downarrow} = 3.6$ s in fixed bacteria with $I_1 = 50$ ein.m^{-2}.s^{-1} and $I_2 = 20$ ein.m^{-2}.s^{-1} (see Supplementary Table 16).

D.4 Colocalization of RSFPs

LIGHTNING can discriminate and recognize not only an RSFP but several co-localized RSFPs as well. In the following, we assume that the sample contains m RSFPs and illustrate the discrimination and recognition of two RSFPs, which can be colocalized. Our approach can be generalized to a larger number of colocalized RSFPs.

The fluorescence properties are compared to the one of pairs (i, j) of RSFPs to determine whether i, j, or both i

and j are present. For a given pair (i, j), we use the kinetic fingerprints given in Supplementary Table 7 to determine the most discriminating dimension k for which the 1-dimensional distance $d_{ij}^k = \max_{k'}(| l_{k'i} - l_{k'j} |)$ is maximum, where $l_{k'i}$ is the decimal logarithm of the characteristic time of the RSFP i under illumination k'. We also rank the corresponding characteristic times leading for example to $\tau_{ki} < \tau_{kj}$. Then we use the monoexpoxential fitting function $U \exp\left(-\frac{t}{\tau}\right) + W$ to the experimental fluorescence evolution I_F^k under illumination k in the interval $[\tau_{kj}, 5\tau_{kj}]$ to evaluate whether RSFP j is present. The lower bound of the interval is found to be the smallest value minimizing the possible impact of exponential functions associated with the shorter characteristic time τ_{ki} without significant detrimental effect on the determination of the longer characteristic time τ_{kj}. The procedure provides the prefactor U_{ka} and the characteristic time τ_{ka}, i.e. l_{ka}. The presence of RSFP j is asserted if the 1-dimensional distance $d_{ja}^k =| l_{kj} - l_{ka} |$ is smaller than a well-defined cutoff distance d_c. The biexponential fitting function $U \exp\left(-\frac{t}{\tau}\right) + U_{ka} \exp\left(-\frac{t}{\tau_{ka}}\right) + W$ to I_F^k, with U_{ka} and τ_{ka} set at the values previously deduced, is used in the interval $[0, 5\tau_{ki}]$ to evaluate whether RSFP i is present. The procedure provides the prefactor U_{kb} and the characteristic time τ_{kb}, i.e. l_{kb}. Similarly the presence of RSFP i is asserted if $d_{ib}^k < d_c$. The fluorescence evolution is analyzed $m(m-1)/2$ times to conclude for all possible pairs, which is sufficient and may be redundant to determine if each RSFP is present.

The cutoff distance d_c does not anymore depend only on the uncertainty M on the determination of the kinetic fingerprint as for non colocalized RSFPs but it also involves an error e on the determination of the characteristic times from applying the preceding fitting protocol. It becomes $d_c = M + e$. We previously estimated M in subsection D.1.3 for experimentally relevant noise levels. When checking if the pair (i, j) is present, the error e on the determination of the characteristic times τ_{ka} and τ_{kb} increases as the distance d_{ij}^k decreases. The method is as accurate as for non colocalized RSFPs when the characteristic times of the two checked RSFPs typically differ by an order of magnitude. An upper bound of e can be obtained by taking the lower bound $d_{\min}$ of d_{ij}^k in an optimized subset $s'(m)$. Hence the error e depends on the number m of considered RSFPs in $s'(m)$.

To evaluate the dependence of e on m, we first implemented an analogous sorting algorithm as in section D.1.2 to generate subsets of RSFPs as distant as possible by computing the minimum distances $d_{\min} = \max_{s'(m)}(\min_{i,j}(d_{ij}^k))$ between pairs (i, j) for all subsets $s'(m)$ of m RSFPs. This sorting protocol has been applied to RSFPs. Among the 22 studied RSFPs, only two RSFPs, **4** and **5**, are positive photoswitchers. The colocalization of positive and negative photoswitchers may blur the fluorescence signal. We therefore considered the 20 negative photoswitchers. The sorting results are given in Supplementary Table 20 and Supplementary Figure 69.

In order to estimate e for the considered sorting of RSFPs, we analyzed a designed signal accounting for the fluorescence evolution of a system containing RSFP i of characteristic time τ_{ki} and RSFP j of characteristic time τ_{kj} under illumination k. We further assumed that the contribution of an RSFP to the fluorescence intensity is adequately described by a monoexponential function and the two preexponential factors are equal. The fluorescence evolution was generated with an additive Gaussian white noise $\epsilon\xi(t)$

$$I_F^k = \exp\left(-\frac{t}{\tau_{ki}}\right) + \exp\left(-\frac{t}{\tau_{kj}}\right) + \epsilon\xi(t) \tag{125}$$

with $\tau_{ki} < \tau_{kj}$, $\langle\xi(t)\rangle = 0$, $\langle\xi(t)\xi(t')\rangle = \delta(t - t')$, and the value $\epsilon = 0.05$ is experimentally relevant.

We determined the mean values $\langle l_{ka}\rangle$, $\langle l_{kb}\rangle$ and the standard deviations σ_{ka}, σ_{kb} from a statistics over 1000 realizations of the noisy signal I_F^k for $l_{ki} = 0$ and $l_{kj} = d_{\min}$ where $d_{\min}$ is given in Supplementary Figure 69. The longer characteristic time τ_{kb} is more precisely determined than the shorter characteristic time τ_{ka} and the error is estimated by $e =| l_{ki} - \langle l_{ka}\rangle | + \sigma_{ka}$. Knowing that the minimum distance $d_{\min}$ depends on the optimized subset $s'(m)$, the error e depends on m. As shown in Supplementary Figure 69a, we found $e = 0.037m$ in the considered conditions. Using $M = 0.05$ as in subsection D.1.3, we retrieved $d_c = 0.037m + 0.05$.

Supplementary Table 20: Optimized subsets $s'(m)$ of m RSFPs for $m = 2, ..., 20$ using the kinetic signature given in Supplementary Table 7 and the minimum distance $d_{\min}$ between pairs.

m	$s'(m)$
2	(**2**,**9**)
3	(**6**,**8**,**9**)
4	(**3**,**9**,**15**,**19**)
5	(**3**,**9**,**10**,**15**,**19**)
6	(**1**,**3**,**6**,**10**,**16**,**19**)
7	(**3**,**6**,**9**,**10**,**14**,**16**,**19**)
8	(**1**,**3**,**6**,**9**,**10**,**12**,**17**,**22**)
9	(**1**,**2**,**3**,**6**,**9**,**10**,**12**,**15**,**19**)
10	(**2**,**3**,**6**,**9**,**10**,**12**,**14**,**15**,**18**,**22**)
11	(**1**,**2**,**3**,**6**,**9**,**10**,**12**,**15**,**18**,**19**,**22**)
12	(**2**,**3**,**6**,**9**,**10**,**12**,**13**,**14**,**15**,**18**,**19**,**22**)
13	(**2**,**3**,**6**,**9**,**10**,**12**,**13**,**14**,**15**,**16**,**18**,**19**,**22**)
14	(**1**,**2**,**3**,**6**,**9**,**10**,**12**,**13**,**15**,**16**,**18**,**19**,**21**,**22**)
15	(**1**,**2**,**3**,**6**,**9**,**10**,**12**,**13**,**14**,**15**,**16**,**18**,**19**,**21**,**22**)
16	(**1**,**2**,**3**,**6**,**9**,**10**,**12**,**13**,**14**,**15**,**16**,**18**,**19**,**20**,**21**,**22**)
17	(**1**,**2**,**3**,**6**,**7**,**9**,**10**,**12**,**13**,**14**,**15**,**16**,**18**,**19**,**20**,**21**,**22**)
18	(**1**,**2**,**6**,**7**,**8**,**9**,**10**,**11**,**12**,**13**,**14**,**15**,**16**,**18**,**19**,**20**,**21**,**22**)
19	(**1**,**2**,**6**,**7**,**8**,**9**,**10**,**11**,**12**,**13**,**14**,**15**,**16**,**17**,**18**,**19**,**20**,**21**,**22**)
20	(**1**,**2**,**3**,**6**,**7**,**8**,**9**,**10**,**11**,**12**,**13**,**14**,**15**,**16**,**17**,**18**,**19**,**20**,**21**,**22**)

For all checked pairs (i', j') of an optimized subset $s'(m)$ and any j', the presence of a given RSFP i is unambiguously asserted if $d^k_{i'a} > d_c$ and $d^k_{i'b} > d_c$, $\forall i' \neq i$, and $d^k_{ia} < d_c$ and $d^k_{ib} < d_c$. Similarly the presence of a given colocalized pair (i, j) such that $\tau_{ki} < \tau_{kj}$ is asserted if $d^k_{i'b} > d_c$ and $d^k_{j'a} > d_c$, $\forall i' \neq i, j' \neq j$, and $d^k_{ib} < d_c$ and $d^k_{ja} < d_c$. Since the presence of a given RSFP i is checked in all trials involving it in a pair, the procedure may lead to distance values leading to an inconsistent conclusion. The conclusion of the presence of an RSFP is more likely to be undecided for a shorter characteristic time.

The presence of any pair of colocalized RSFPs in a sample containing m RSFPs can be asserted if $d_{\min}$ is larger than the cutoff distance d_c. As shown in Supplementary Figure 69b up to 9 RSFPs can be discriminated in these conditions.

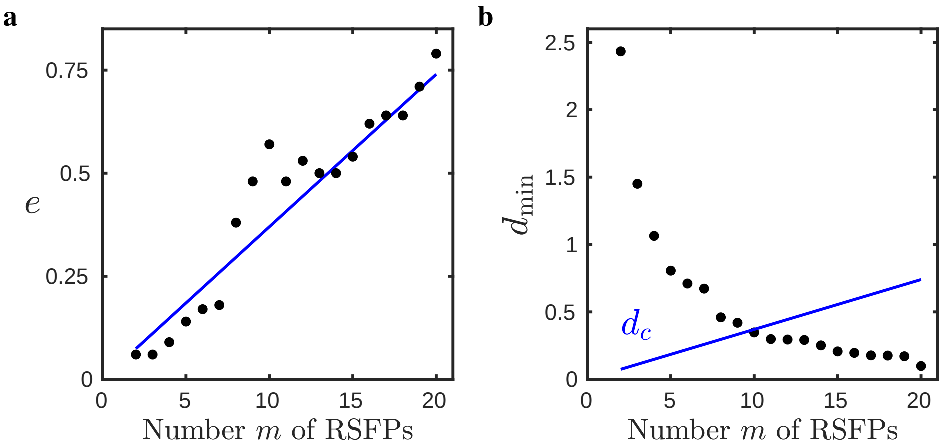

Supplementary Figure 69: *The LIGHTNING discriminatory power of colocalized RSFPs.* **a**: Error e on the determination of each characteristic time of two colocalized RSFPs deduced from a designed noisy fluorescence evolution versus number m of possible RSFPs in the sample. The blue line $e = 0.037m$ fits the results. **b**: Minimum distance $d_{\min}$ between pairs for optimized subsets $s'(m)$ of RSFPs versus cardinal m of the subsets. The threshold $d_c = e + 0.05$ is deduced from the uncertainty on the determination of the characteristic time of a colocalized RSFP and a non colocalized RSFP. The condition $d_{\min} \geq d_c$ fixes the overall number of discriminatable colocalized RSFPs.

Supplementary References

[1] R. Ando, H. Mizuno, and A. Miyawaki. Regulated fast nucleocytoplasmic shuttling observed by reversible protein highlighting. *Science*, 306:1370–1373, 2004.

[2] A. C. Stiel, S. Trowitzsch, G. Weber, M. Andresen, C. Eggeling, S. W. Hell, S. Jakobs, and M. C. Wahl. 1.8 å bright-state structure of the reversibly switchable fluorescent protein Dronpa guides the generation of fast switching variants. *Biochem. J.*, 402:35–42, 2007.

[3] R. Ando, C. Flors, H. Mizuno, J. Hofkens, and A. Miyawaki. Highlighted generation of fluorescence signals using simultaneous two-color irradiation on Dronpa mutants. *Biophys. J.*, 92:L97 – L99, 2007.

[4] D. K. Tiwari, Y. Arai, M. Yamanaka, T. Matsuda, M. Agetsuma, M. Nakano, K. Fujita, and T. Nagai. A fast- and positively photoswitchable fluorescent protein for ultralow-laser-power RESOLFT nanoscopy. *Nat. Meth.*, 12:515–518, 2015.

[5] M. Andresen, A. C. Stiel, J. Follig, D. Wenzel, A. Schoenle, A. Egner, C. Eggeling, S. W. Hell, and S. Jakobs. Photoswitchable fluorescent proteins enable monochromatic multilabel imaging and dual color fluorescence nanoscopy. *Nat. Biotech.*, 26:1035–1040, 2008.

[6] T. Grotjohann, I. Testa, M. Reuss, T. Brakemann, C. Eggeling, S. W. Hell, and S. Jakobs. rsEGFP2 enables fast RESOLFT nanoscopy of living cells. *ELife*, 1, 2012.

[7] J. Quérard, R. Zhang, Z. Kelemen, M.-A. Plamont, X. Xie, R. Chouket, I. Roemgens, Y. Korepina, S. Albright, E. Ipendey, M. Volovitch, H. L. Sladitschek, P. Neveu, L. Gissot, A. Gautier, J.-D. Faure, V. Croquette, T. Le Saux, and L. Jullien. Resonant out-of-phase fluorescence microscopy and remote imaging overcome spectral limitations. *Nat. Comm.*, 8:969, 2017.

[8] C. Duan, V. Adam, M. Byrdin, J. Ridard, S. Kieffer-Jaquinod, C. Morlot, D. Arcizet, I. Demachy, and D. Bourgeois. Structural evidence for a two-regime photobleaching mechanism in a reversibly switchable fluorescent protein. *J. Am. Chem. Soc.*, 135:15841–15850, 2013.

[9] Paula J Cranfill, Brittney R Sell, Michelle A Baird, John R Allen, Zeno Lavagnino, H Martijn De Gruiter, Gert-Jan Kremers, Michael W Davidson, Alessandro Ustione, and David W Piston. Quantitative assessment of fluorescent proteins. *Nat. Meth.*, 13(7):557, 2016.

[10] Stéfan van der Walt, Johannes L. Schönberger, Juan Nunez-Iglesias, Francois Boulogne, Joshua D. Warner, Neil Yager, Emmanuelle Gouillart, and Tony Yu. scikit-image: image processing in python. 2:e453. Publisher: PeerJ Inc.

[11] Edward Dougherty. *Mathematical Morphology in Image Processing*. CRC Press.

[12] Xiangyang Xu, Shengzhou Xu, Lianghai Jin, and Enmin Song. Characteristic analysis of otsu threshold and its applications. 32(7):956–961, 2011.

[13] D. Summers and J. M. W. Scott. Systems of first-order chemical reactions. *Mathl. Comput. Modelling*, 10:901–909, 1988.

[14] J. Crank. *The Mathematics of Diffusion (2nd edn)*. Oxford University Press, Oxford, 1975.

[15] A. A. Istratov and O. F. Vyvenko. Exponential analysis in physical phenomena. *Rev. Sci. Instrum.*, 70:1233–1257, 1999.

[16] G. L. Miller, J. V. Ramirez, and D. A. H. Robinson. A correlation method for semiconductor transient signal measurements. *J. Appl. Phys.*, 46:2638–2644, 1975.

[17] S. W. Provencher. An eigenfunction expansion method for the analysis of exponential decay curves. *J. Chem. Phys.*, 64:2772–2777, 1976.

[18] P. M. Henry, J. M. Meese, J. W. Farmer, and C. D. Lamp. Frequency-scanned deep-level transient spectroscopy. *J. Appl. Phys.*, 57:628–630, 1985.

[19] J. Ferenczi, J. Boda, and T. Pavelka. Isothermal frequency scan dlts. *Phys. Status Solidi A*, 94:K119–K124, 1986.

[20] L. Stuchlikova, L. Harmatha, V. Nagl, and M. Gazi. Determination of deep-level parameters by isothermal deep-level transient spectroscopy with optical excitation. *Phys. Status Solidi A*, 138:K241–K248, 1993.

[21] A. A. Istratov. The resolution limit of traditional correlation functions for deep-level transient spectroscopy. *Rev. Sci. Instrum.*, 68:3861–3865, 1997.

[22] A. A. Istratov. New correlation procedure for rthe improvement of resolution of deep-level transient spectroscopy of semiconductors. *J. Appl. Phys.*, 82:2965–2968, 1997.

[23] M. M. Warren, M. Kaucikas, A. Fitzpatrick, P. Champion, J. T. Sage, and J. J. van Thor. Ground-state proton transfer in the photoswitching reactions of the fluorescent protein dronpa. *Nat. Comm.*, 4:1461, 2013.

[24] D. Yadav, F. Lacombat, N. Dozova, F. Rappaport, P. Plaza, and A. Espagne. Real-time monitoring of chromophore isomerization and deprotonation during the photoactivation of the fluorescent protein dronpa. *J. Phys. Chem. B*, 119:2404–2414, 2015.

[25] J. P. Colletier, M. Sliwa, F. X. Gallat, M. Sugahara, V. Guillon, G. Schiro, N. Coquelle, J. Woodhouse, L. Roux, G. Gotthard, A. Royant, L. M. Uriarte, C. Ruckebusch, Y. Joti, M. Byrdin, E. Mizohata, E. Nango, T. Tanaka, K. Tono, M. Yabashi, V. Adam, M. Cammarata, I. Schlichting, D. Bourgeois, and M. Weik. Serial femtosecond crystallography and ultrafast absorption spectroscopy of the photoswitchable fluorescent protein irisfp. *J. Phys. Chem. Lett.*, 7:882–887, 2016.

[26] N. Coquelle, M. Sliwa, J. Woodhouse, G. Schiro, V. Adam, A. Aquila, T. R. M. Barends, S. Boutet, M. Byrdin, S. Carbajo, E. De la Mora, R. B. Doak, M. Feliks, F. Fieschi, L. Foucar, V Guillon, M. Hilpert, M. S. Hunter, S. Jakobs, J. E. Koglin, G. Kovacsova, T. J. Lane, B. Lévy, M. Liang, K. Nass, J. Ridard, J. S. Robinson, C. M. Roome, Ruckebusch C., M. Seaberg, M. Thepaut, M. Cammarata, I. Demachy, M. Field, R. L. Shoeman, D. Bourgeois, J. P. Colletier, I. Schlichting, and M. Weik. Chromophore twisting in the excited state of a photoswitchable fluorescent protein captured by time-resolved serial femtosecond crystallography. *Nat. Chem.*, 10:31–37, 2017.

[27] Sergey P Laptenok, Agnieszka A Gil, Christopher R Hall, Andras Lukacs, James N Iuliano, Garth A Jones, Gregory M Greetham, Paul Donaldson, Atsushi Miyawaki, Peter J Tonge, et al. Infrared spectroscopy reveals multi-step multi-timescale photoactivation in the photoconvertible protein archetype dronpa. *Nat. Chem.*, 10(8):845, 2018.

[28] Martin Byrdin, Chenxi Duan, Dominique Bourgeois, and Klaus Brettel. A long-lived triplet state is the entrance gateway to oxidative photochemistry in green fluorescent proteins. *J. Am. Chem. Soc.*, 140(8):2897–2905, 2018.

[29] Y.-T. Kao, X. Zhu, and W. Min. Protein-flexibility mediated coupling between photoswitching kinetics and surrounding viscosity of a photochromic fluorescent protein. *Proc. Natl. Acad. Sci. U. S. A.*, 109:3220–3225, 2012.

[30] J. Quérard, T.-Z. Markus, M.-A. Plamont, C. Gauron, P. Wang, A. Espagne, M. Volovitch, S. Vriz, V. Croquette, A. Gautier, T. Le Saux, and L. Jullien. Photoswitching kinetics and phase-sensitive detection add discriminative dimensions for selective fluorescence imaging. *Angew. Chem. Int. Ed.*, 127:2671–2675, 2015.

[31] R. Zhang, R. Chouket, M.-A. Plamont, Z. Kelemen, A. Espagne, A. G. Tebo, A. Gautier, L. Gissot, J.-D. Faure, L. Jullien, V. Croquette, and T. Le Saux. Macroscale fluorescence imaging against autofluorescence under ambient light. *Light: Science & Applications*, 7:97, 2018.

[32] Ruikang Zhang, Raja Chouket, Alison G Tebo, Marie-Aude Plamont, Zsolt Kelemen, Lionel Gissot, Jean-Denis Faure, Arnaud Gautier, Vincent Croquette, Ludovic Jullien, and Thomas Le Saux. Simple imaging protocol for autofluorescence elimination and optical sectioning in fluorescence endomicroscopy. *Optica*, 6(8):972–980, 2019.
