# Supplementary material for: Extra kinetic dimensions for label discrimination
Source: Nat Commun. 2022 Mar 18;13:1482. doi: 10.1038/s41467-022-29172-0 (PMC8933551; doi:10.1038/s41467-022-29172-0)
Supplement: Supplementary file 3 — Reporting Summary [file 41467_2022_29172_MOESM3_ESM.pdf]

nature portfolio

Corresponding author(s): JULLIEN Ludovic

Last updated by author(s): Feb 15 2022

Reporting Summary

Nature Portfolio wishes to improve the reproducibility of the work that we publish. This form provides structure for consistency and transparency in reporting. For further information on Nature Portfolio policies, see our Editorial Policies and the Editorial Policy Checklist.

Statistics

For all statistical analyses, confirm that the following items are present in the figure legend, table legend, main text, or Methods section.

n/a	Confirmed	
☐	☒	The exact sample size (n) for each experimental group/condition, given as a discrete number and unit of measurement
☐	☒	A statement on whether measurements were taken from distinct samples or whether the same sample was measured repeatedly
☒	☐	The statistical test(s) used AND whether they are one- or two-sided<br>*Only common tests should be described solely by name; describe more complex techniques in the Methods section.*
☒	☐	A description of all covariates tested
☐	☒	A description of any assumptions or corrections, such as tests of normality and adjustment for multiple comparisons
☐	☒	A full description of the statistical parameters including central tendency (e.g. means) or other basic estimates (e.g. regression coefficient) AND variation (e.g. standard deviation) or associated estimates of uncertainty (e.g. confidence intervals)
☒	☐	For null hypothesis testing, the test statistic (e.g. F, t, r) with confidence intervals, effect sizes, degrees of freedom and P value noted<br>*Give P values as exact values whenever suitable.*
☒	☐	For Bayesian analysis, information on the choice of priors and Markov chain Monte Carlo settings
☒	☐	For hierarchical and complex designs, identification of the appropriate level for tests and full reporting of outcomes
☒	☐	Estimates of effect sizes (e.g. Cohen's d, Pearson's r), indicating how they were calculated

Our web collection on statistics for biologists contains articles on many of the points above.

Software and code

Policy information about availability of computer code

Data collection	Labview 2010 (photoswitchometer) and HCImage 4.4.2.7 (Hamamatsu Corporation) for imaging.
Data analysis	Igor Pro 8; Mathematica 12.3.1.0; Inkscape 1.1; Matlab 2019b, Python 3.6; The code for processing the images is available at https://github.com/Alienor134/image_segmentation (https://doi.org/10.5281/zenodo.5684342).

For manuscripts utilizing custom algorithms or software that are central to the research but not yet described in published literature, software must be made available to editors and reviewers. We strongly encourage code deposition in a community repository (e.g. GitHub). See the Nature Portfolio guidelines for submitting code & software for further information.

Data

Policy information about availability of data

All manuscripts must include a data availability statement. This statement should provide the following information, where applicable:
- Accession codes, unique identifiers, or web links for publicly available datasets
- A description of any restrictions on data availability
- For clinical datasets or third party data, please ensure that the statement adheres to our policy

All data generated or analysed during this study are included in this published article (and its supplementary information files).

Field-specific reporting

Please select the one below that is the best fit for your research. If you are not sure, read the appropriate sections before making your selection.

☒ Life sciences ☐ Behavioural & social sciences ☐ Ecological, evolutionary & environmental sciences

For a reference copy of the document with all sections, see nature.com/documents/nr-reporting-summary-flat.pdf

Life sciences study design

All studies must disclose on these points even when the disclosure is negative.

Sample size	In the case of the series of experiments dealing with LIGHTNING imaging of bacteria, at least 50 bacteria were analyzed to build the plots displayed in Figures S43-S46 of the Supporting Information. This size is sufficient to estimate the mean and the standard deviation of the distribution of the characteristic times and retrieve the LIGHTNING kinetic fingerprint. The other reported distributions (RSFP characteristic times in solution or light intensities) have been obtained from an even larger number of individual experiments.
Data exclusions	In the series of experiments dealing with LIGHTNING imaging of bacteria, we introduced a series of criteria led to discard background objects that were misidentified as bacteria because of their size and bacteria, which were exhibiting a low signal-to-noise ratio (such as 2-, 16- and 21-labeled bacteria) or dividing (resulting in highly dispersed values among their pixels and abnormal mean value of the characteristic times). The selection criteria for the recognition of bacteria were pre-established before image analysis and they are detailed in § 1.6.3 of the Supporting Information.
Replication	All the experiments reported in this manuscript have been reproduced at least twice.
Randomization	The samples investigated in this manuscript have been randomly produced and examined. Their data have been acquired and processed without any anticipation of the results to be obtained.
Blinding	Blinding was not involved in our study. Indeed most experiments have necessitated to adjust the acquisition conditions to the samples to be measured. Yet, all the investigated samples have been randomly produced and examined and the reported results have been obtained without any specific anticipation.

Reporting for specific materials, systems and methods

We require information from authors about some types of materials, experimental systems and methods used in many studies. Here, indicate whether each material, system or method listed is relevant to your study. If you are not sure if a list item applies to your research, read the appropriate section before selecting a response.

Materials & experimental systems

n/a	Involved in the study	
☒	☐	Antibodies
☒	☐	Eukaryotic cell lines
☒	☐	Palaeontology and archaeology
☒	☐	Animals and other organisms
☒	☐	Human research participants
☒	☐	Clinical data
☒	☐	Dual use research of concern

Methods

n/a	Involved in the study	
☒	☐	ChIP-seq
☒	☐	Flow cytometry
☒	☐	MRI-based neuroimaging
